# A Review of Microwave Synthesis of Zinc Oxide Nanomaterials: Reactants, Process Parameters and Morphologies

**DOI:** 10.3390/nano10061086

**Published:** 2020-05-31

**Authors:** Jacek Wojnarowicz, Tadeusz Chudoba, Witold Lojkowski

**Affiliations:** Institute of High Pressure Physics, Polish Academy of Sciences, Sokolowska 29/37, 01-142 Warsaw, Poland; chudoba@unipress.waw.pl (T.C.); w.lojkowski@labnano.pl (W.L.)

**Keywords:** zinc oxide (ZnO), nanostructures (NSs), nanomaterials (NMs), nanoparticles (NPs), microwave heating, microwave assisted synthesis, microwave synthesis, microwave reactors

## Abstract

Zinc oxide (ZnO) is a multifunctional material due to its exceptional physicochemical properties and broad usefulness. The special properties resulting from the reduction of the material size from the macro scale to the nano scale has made the application of ZnO nanomaterials (ZnO NMs) more popular in numerous consumer products. In recent years, particular attention has been drawn to the development of various methods of ZnO NMs synthesis, which above all meet the requirements of the green chemistry approach. The application of the microwave heating technology when obtaining ZnO NMs enables the development of new methods of syntheses, which are characterised by, among others, the possibility to control the properties, repeatability, reproducibility, short synthesis duration, low price, purity, and fulfilment of the eco-friendly approach criterion. The dynamic development of materials engineering is the reason why it is necessary to obtain ZnO NMs with strictly defined properties. The present review aims to discuss the state of the art regarding the microwave synthesis of undoped and doped ZnO NMs. The first part of the review presents the properties of ZnO and new applications of ZnO NMs. Subsequently, the properties of microwave heating are discussed and compared with conventional heating and areas of application are presented. The final part of the paper presents reactants, parameters of processes, and the morphology of products, with a division of the microwave synthesis of ZnO NMs into three primary groups, namely hydrothermal, solvothermal, and hybrid methods.

## 1. Introduction

### 1.1. Nanotechnology

Nanotechnology is one of the most rapidly developing disciplines of science and technology. It was the reason for the global industrial revolution of the 21st century [1,2,3,4,5,6,7,8,9,10]. At present, this is a leading technology within various research areas, such as: physics, biology, chemistry, biochemistry, biotechnology, medicine, materials and biomedical engineering, electronics, optoelectronics, mechatronics, spintronics, energy generation, food and agriculture, environmental protection, and interdisciplinary fields [11,12,13,14,15,16,17,18,19,20,21,22,23,24,25,26,27,28,29,30,31,32,33,34,35,36,37,38]. This technology enables testing, controlling, regulating, modifying, processing, producing, and using structures in which at least one of the dimensions does not exceed 100 nanometres (1 nm = 10^−9^ m) [39]. Materials in the nano-scale are characterised by new specific properties and phenomena, which sometimes considerably deviate from the characteristic properties of the same materials appearing in the micro-scale [40,41,42,43,44,45,46,47,48,49,50]. The observed changes to the physicochemical properties are caused primarily by two factors, namely the quantum confinement of electrons and increased share of surface atoms/ions in relation to atoms/ions present inside the particle. The big development of the specific surface area of the nanomaterial leads to an increase in the number of unsaturated coordination centres, defects and stresses in the crystal lattice, and in the chemical reactivity of particles [51,52]. 

Nanotechnology enables the application of nanomaterials to create innovative products, devices, and complex systems that make use of material properties in the nano-scale [11]. Products developed and created thanks to the use of nanotechnology are present in all aspects related to the human activity on Earth and in the outer space [2,3,4,5,6,7,8,9,10,11,53,54,55,56,57,58,59,60,61,62,63,64,65,66,67]. The current research concerning the applications of nanotechnology in the broadly understood catalysis, optoelectronics, microelectronics, biomedicine and pharmacy concentrate on the control of the physicochemical properties of nanostructures (NSs) [68,69], including of the nano zinc oxide (ZnO) [70]. Numerous tests related to the use of nano ZnO for application purposes are in progress and attempts are made to produce them on an industrial scale. The dynamic development of methods of repeatable production of nano ZnO, characterised by high purity, controlled properties, high efficiency, and shortest synthesis duration possible, is desirable [71].

### 1.2. Bulk ZnO: Properties and Application

**Figure 1:**
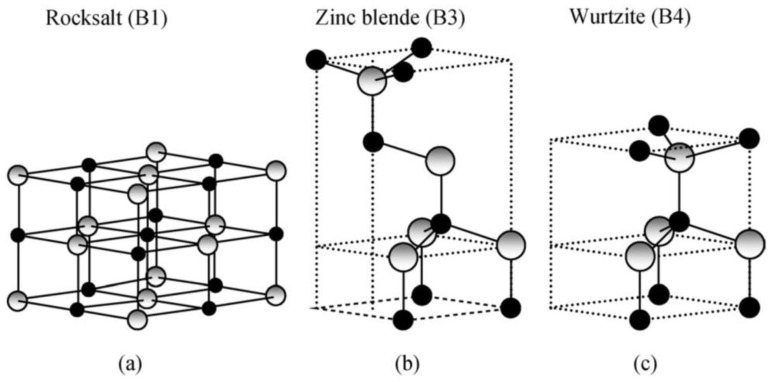
Stick and ball representation of ZnO crystal structures: (**a**) cubic rocksalt (B1), (**b**) cubic zinc blende (B3), and (**c**) hexagonal wurtzite (B4). The shaded grey and black spheres denote Zn and O atoms, respectively. Reprinted from [97], with the permission of John Wiley & Sons, Inc. Copyright 2009. All rights reserved. In order to re-use permission must be obtained from the rightsholder.

**Figure 2:**
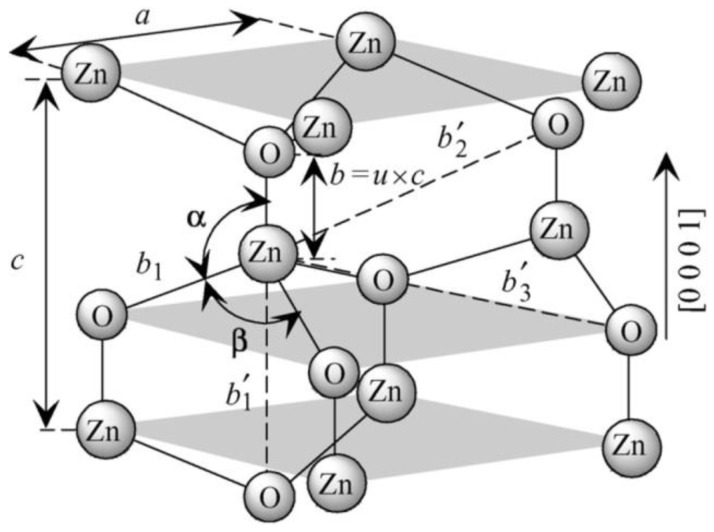
Schematic representation of a wurtzitic ZnO structure with lattice constants a in the basal plane and c in the basal direction, u parameter, which is expressed as the bond length or the nearest-neighbour distance **b** divided by **c** (0.375 in an ideal crystal), **a** and **b** (109.47 in an ideal crystal) bond angles, and three types of second-nearest-neighbour distances **b**′_1_, **b**′_2_, and **b**′_3_. Reprinted from [97], with the permission of John Wiley & Sons, Inc. Copyright 2009. All rights reserved. In order to re-use permission must be obtained from the rightsholder.

Owing to its exceptional physical (Table 1) and chemical properties, ZnO counts as a multifunctional material [72,73,74,75]. Its properties include high electrochemical coupling coefficient, broad range of radiation absorption, high photostability, low toxicity, biocompatibility, and biodegradability [76]. It occurs naturally in Earth’s crust in the form of a mineral called zincite (Zn_1−*x*_M*_x_*O). Zincite rarely occurs in a pure form; most often it includes dopants of other bivalent metals (among others: Mn(II) and Fe(II)), which is manifested in various colours of the mineral, among others, red, red and yellow, orange, and brown [77]. ZnO is insoluble in water and its powdered form is white. Due to its amphoteric properties it reacts both with acids and bases. ZnO can crystallise in three primary crystal systems: hexagonal wurtzite, cubic zinc blende, and cubic rocksalt (Figure 1). In normal conditions, the thermodynamically stable crystal structure of ZnO is wurtzite (Figure 2) [78]. Hexagonal ZnO is an II–VI semiconductor characterised by a wide band gap. A wide band gap (3.37 eV) and a high bond energy (60 meV) make ZnO a subject of enormous interest as regards applications in optoelectronics and electronics (among others, voltage dependent resistors) [79,80]. Owing to its antibacterial (disinfecting) properties, ZnO is widely applied as an ingredient of medications for various purposes [81,82]. The earliest information about the use of ZnO as an ingredient of therapeutic ointments for treating skin boils and ulcers comes already from 2000 BC [83,84,85,86]. It is common knowledge that ZnO was widely produced and used in the 13th century in Persia, where it was applied in treatment of eye inflammation and production of brass [86]. ZnO accelerates wound healing, which is why it is used as an ingredient of pastes (e.g., zinc paste) for treating skin inflammations (among others, acme, skin eruptions) and against itching. It is also commonly used in dentistry, e.g., as temporary tooth dressing [87] and root canal filling materials for deciduous teeth [88]. Moreover, ZnO is used as an additive in feed for pigs, where it helps protect piglets from diarrhoea by inhibiting the growth of pathogenic flora [89,90]. In the past, ZnO was also an ingredient of a medication for treating diarrhoea in humans, but at present, it is used in the oral form, most often as an ingredient of food products and dietary supplements, where it acts as a source of zinc (Zn^2+^). Zinc is the second most important trace element in the human body after iron, as it fulfils the catalytic, structural and regulatory function. The average zinc (Zn^2+^) content in the organism of an adult is 2–3 g [91], while the recommended daily average dose for a healthy adult is 8–11 mg [92]. ZnO is used as an ultraviolet (UV) radiation filter in sunscreen cosmetics (creams, balms, lotions). Nowadays ZnO is commonly used as an additive in such products as pigments (zinc white, zinc green), flame retardants, cements, ceramics, porcelain, glass, plastics, sealants, adhesives, lubricating oils and paints [70,76,89,90,93,94]. The presence of ZnO in paint, apart from changing the colour, contributes also to providing the paint coat with anti-corrosion and antifouling properties. The most important area of ZnO application is the rubber industry, where it is widely used as an activator of the conventional process of sulphur vulcanisation of natural rubber [95]. At present, as much as ca. 50% of the produced ZnO is used for the vulcanisation of rubber, which is a material for manufacturing tyres, sports equipment, garden and industrial hoses, shoe soles, belts, and other rubber products [96]. The presence of ZnO in rubber enhances its strength, hardness, dynamic stability, capacity to absorb heat, and provides important pigmenting properties.

### 1.3. Nano ZnO: Properties and Application

**Figure 3:**
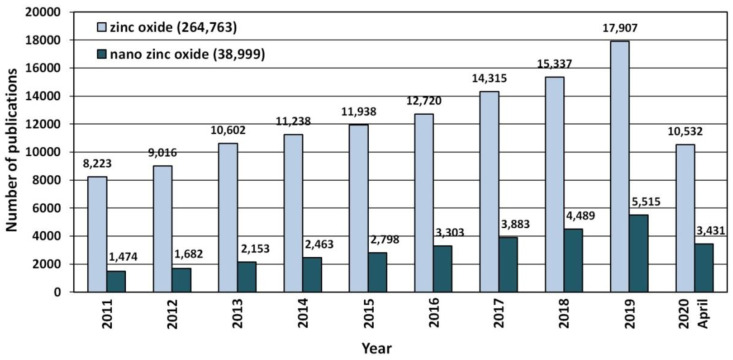
The number of scientific publications referring to the search of “zinc oxide” and “nano zinc oxide” phrases published in the period of 2011–2020. Source: ScienceDirect (accessed on 19 April 2020).

**Figure 4:**
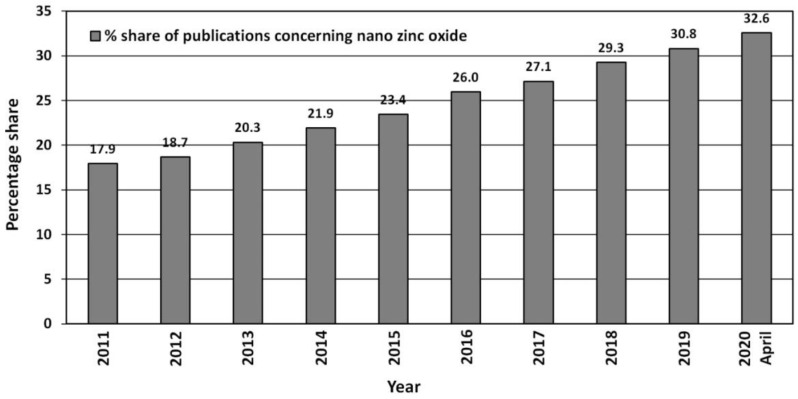
Percentage share of publications concerning “nano zinc oxide” among all publications concerning “zinc oxide” published in the period 2011–2020. Source: ScienceDirect (accessed on 19 April 2020).

Due to its unique properties ZnO enjoys unfading popularity among research units worldwide (Figure 3). Increasingly often, researchers focus on ZnO in the nano-form, which is proven by the search results in the ScienceDirect scientific paper search engine (Figure 3). The number of hits was as follows: “zinc oxide”—264,763 hits, and “nano zinc oxide”—38,999 hits. The overall share of publications concerning “nano zinc oxide” among all the hitherto published papers about ZnO was ≈14.7%. The number of publications concerning nano ZnO have been growing dynamically year by year (Figure 4), while the annual share of publications concerning nano ZnO in 2020 was as high as 32.6% (Figure 4). Over the past 10 years, the number of scientific publications related to nano ZnO increased by ca. 400% (Figure 3). The growing popularity of nano ZnO results among others from the technological development of production of ZnO nanostructures, which are characterised by novel physical properties allowing hitherto unknown applications and possibilities [71,108,109,110].

The main limitation related to the synthesis and application of various types of nanopowders, obviously including nano ZnO, is the problem of repeatability of parameters of nanopowders. Most “wet methods” [73,76,86,111] used for producing metal oxide nanoparticles lead to obtaining powders with a broad particle size distribution, insufficient crystallinity degree, variable morphology and insufficient purity. This causes a range of problems with respect to their application in the broadly understood industry, where properties of raw materials are the crucial and decisive parameter for their choice and guarantee the performance characteristics of the product being manufactured. Research on ZnO nanomaterials (ZnO NMs) can be intensified in line with the development of new methods of production and control. The evolution of nanoparticle characterisation techniques [112] and the standardisation of nanotechnology [113] currently permit determining whether changes to the ZnO NMs properties are, e.g., caused by unrepeatability of methods of obtaining, actual changes to NMs features (among others, material ageing, etc.) or unrepeatability of research procedures. An important role in research on ZnO NMs is played by a network of Accredited Research Laboratories, where increasingly more accurate and appropriately validated measurement techniques are introduced. The large number of published scientific papers devoted to the issues of nano ZnO prove that the topic of synthesis, properties, and application of such powders is researched intensively. This indicates an enormous development potential of nano ZnO, which was noticed and appreciated by numerous research groups worldwide.

Nano ZnO is regarded as a safe material [114], which excellently fulfils the function of an ultraviolet (UV) radiation filter, because it permits creation of protective layers which are invisible to the human eye. Other advantages of the use of ZnO nanoparticles (NPs) as a UV filter in the area of personal hygiene and sun protection are, among others, long-term protection and broadband protection (UV-A (315–380 nm) and UV-B (280–315 nm)) [115]. The impact of contact between ZnO NPs and human skin is still being monitored and tested [116,117,118,119,120,121]. ZnO NPs are also applied in deodorants, medical and sanitary materials, glass, ceramic, as well as self-cleaning materials [115,122]. In optoelectronics, various ZnO nanostructures are used for producing e.g., refractive index sensors [123], surface-enhanced Raman scattering (SERS) sensors [124], lasers, UV detectors, and UV diodes [74,125,126,127,128]. ZnO nanostructures may serve for producing sensors of gases [129,130,131,132,133,134,135,136,137,138,139,140,141,142,143,144,145,146,147,148,149,150,151,152] such as steam (humidity, H_2_O) [133], ammonia (NH_3_) [129,130,131,143], nitrogen (N_2_) [131], nitrogen monoxide (NO) [129,130,139], nitrogen dioxide (NO_2_) [129,131,134,135,136,151,152]_,_ hydrogen (H_2_) [129,130,131,132,149]_,_ ozone (O_3_) [131,137]_,_ hydrogen sulfide (H_2_S) [129,130,131,138,139], carbon monoxide (CO) [129,130,131,135,152], carbon dioxide (CO_2_) [140], methane (CH_4_) [141], acetylene (C_2_H_2_) [131], ethylene (C_2_H_4_) [147], ethane (C_2_H_6_) [129], 1,2-dichloroethane (C_2_H_4_Cl_2_) [129], p-xylene (C_8_H_10_) [129], phenol (C_6_H_5_OH) [130], chlorobenzene (C_6_H_5_Cl) [130], methanol (CH_3_OH) [131], ethanol (C_2_H_5_OH) [129,130,131,148,150], formaldehyde (HCHO) [129,130,131,151], acetaldehyde (CH_3_CHO) [131], acetone ((CH_3_)_2_CO) [129,130,131,144,145,146], mixture of propane (C_3_H_8_) and butane (C_4_H_10_) [130,142], and triethylamine (C_6_H_15_N) [129]. The application of ZnO nanostructures in the commercial development of new gas sensors is quite strongly limited for the time being, which is a consequence above all of the ageing effect [153]. Thin films of nano ZnO are most often used as inorganic conductors in flexible and transparent devices [154,155], e.g., transparent electrodes, transparent windows, flat panel displays, and components of devices with the surface acoustic wave [156,157,158,159,160,161].

The antibacterial and antifungal properties of ZnO NPs have become the focus of interest of pharmacy, biomedicine and dentistry due to their low toxicity to the human organism [162,163,164,165,166,167,168,169,170,171,172,173,174,175,176,177,178,179,180,181]. It has been proved that ZnO NPs applied in textiles effectively fight bacteria, e.g., *Staphylococcus aureus* or *Klebsiella pneumoniae* [182]. In medicine, research is in progress on the application of ZnO NMs as a potential contrast agent (imaging), drug carrier, iron delivery, gene carrier, biosensor, a potential anti-cancer agent, in a photodynamic therapy, for prophylactic and therapeutic vaccines, support for antifungal treatments, in photocatalytic antibiotics, inhibition of influenza virus infection, diagnostic-therapeutic functions, wound dressing and tissue engineering [70,166,168,183,184,185,186,187,188,189,190,191,192,193,194,195,196,197,198,199,200,201,202,203,204,205,206,207,208,209,210,211,212,213]. However, before implementing ZnO NMs in biomedical applications on a commercial scale, the toxicity of ZnO NMs must be carefully learnt and their toxicity mechanisms must be explained [121,162,171,174,178,179,214,215,216,217,218,219]. Once doped, they display new properties, e.g., electrical conductivity, magnetic, magneto-optical, photocatalytic, antibacterial and optical [74,161,220,221,222,223,224,225,226,227,228,229,230,231,232]. At present, various ZnO nanostructures are being used in attempts to produce a new generation of light-emitting diodes [233,234], lasers [235], field emission devices [236,237], memory carriers [238,239], solar cells [240,241,242,243,244,245,246], liquid crystals [247], polymer nanocomposites [248,249,250,251], food packaging materials [252,253,254,255,256,257,258], transparent ultraviolet light absorbers in unplasticised polymers [259], catalysts [260], photoluminescent NPs [261], photocatalysts [262,263,264,265,266,267,268,269,270,271,272,273], hybrid materials [272,273,274], chemical sensors [275,276], optical biosensors [277], nanofibrous materials [278,279], piezoelectric nanogenerators [280,281], nano-piezotronics [282], supercapacitors [155], flexible and transparent thin-film transistors [154,155], batteries [283,284], photoelectrochemical applications [285], water treatment [286,287,288,289,290,291], water filters [292], solar water splitting [266,293,294,295], functional coatings [296,297,298,299,300,301,302,303,304,305], and in crop cultivation [306,307]. ZnO nanostructures are investigated intensively for the purpose of their application production of chemical and biological sensors [190,308,309]. 

In agriculture, research is in progress on the application of the antifungal properties of ZnO NPs to protect crops [307,310]. ZnO NPs are used in a broad range of commercial applications, thus it is important to understand their fate and behaviour in soil, water, their manner of absorption and spreading/accumulation in plants, animals and microorganisms, but also interactions with impurities [311,312,313,314,315,316,317,318,319,320,321,322,323,324,325,326,327,328,329,330,331,332,333,334]. It must be borne in mind that the dynamically increasing the commercial application of ZnO NPs without appropriate supervision and tests may contribute to their adverse impact on land and water plants and animals [335,336].

The properties of nanostructures depend strongly on their size and shape [40,41,42,43,44,45,46,47,48,49,50]. The size-dependent effect of ZnO NPs has been observed on the: photocatalytic activity [337,338,339,340,341,342], catalyst activity [343], dielectric properties [344], piezoelectric property [345,346], breakdown voltage varistors [347], visible emission property of quantum dots displays [348], equilibrium constant of chemical reactions [349], gas sensing properties [350,351,352], thermal diffusivity of water nanofluid [353], photoluminescence [354], UV absorption [355,356], biomedical potential [357], toxicity [312,328,357,358,359,360,361,362], bioavailability [363], and interactions with biomatrices [364]. A nano ZnO material may be characterised by piezoelectric parameters of even a few times higher values than a bulk ZnO material [365]. The photoluminescence, band gap width, conductivity, or magnetic properties, in turn, may be controlled by doping ZnO NPs with transition metal ions (dopant quantity and type): e.g., Co, Mn, Cr, Ni, Fe, V) [74,223,224,225,226,227,228,229,230,231,232]. It was found that the UV sensing performance of doped ZnO improved in term of sensitivity and photoresponse properties compared to non-doped ZnO [366]. One of the goals of research concerning doped ZnO (diluted magnetic semiconductor—DMS) is to enable a combination of communication, memory, and data processing in a single device. They will serve for creating a new generation of smaller, faster and less energy-consuming devices than the present ones, acting based on a combination of conventional micro-electronics and spin-dependent effects [367,368].

In order that ZnO could be more willingly used in products on the industrial scale, the development of ZnO NPs synthesis methods is necessary to enable simultaneous precise control of the average particle size, obtaining narrow particle size distributions and their doping with transition ions. For the purpose of achieving the required performance characteristics, e.g., in pharmaceutical applications, it is also necessary that the material is homogeneous, fully crystalline, and characterised by high purity.

### 1.4. ZnO Market

**Figure 5:**
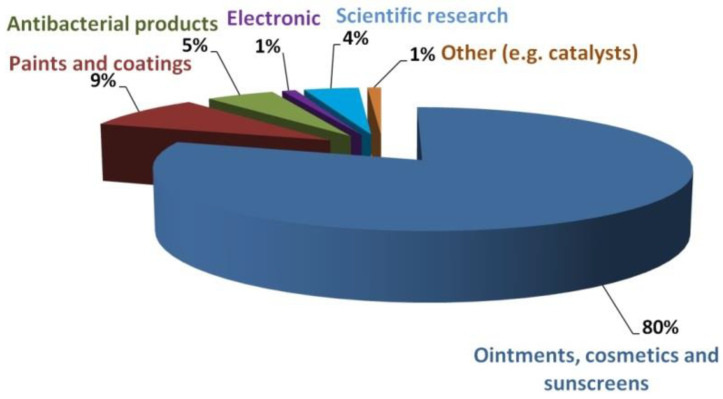
Estimated demand for ZnO NPs on the market in terms of their applications. The chart was prepared based on data from [115].

It is estimated that the production of NPs of metal oxides in 2020 will be ca. 660 thousand tons, with the estimated production of ZnO NPs alone being between 45 and 56 thousand tons, i.e., ca. 7–8% of the market [115,122]. At present, ZnO NPs are used mainly for producing the following (Figure 5):

- pharmaceuticals,
- cosmetics,
- paints,
- various coatings,
- antibacterial products,
- electronics,
- and in scientific research.

### 1.5. Obtaining ZnO Nanomaterials

The synthesis of ZnO NMs with repeatable properties is a quite difficult topic because some of the ZnO nanopowders available on the market are characterised by an insufficient degree of crystallinity, variable morphology, wide particle size distribution, presence of impurities and unrepeatability of properties. In addition, available ZnO nanopowders are mostly composed of a mixture of NPs agglomerates and aggregates, which can be confirmed by a test of average particle size for a dry powder using the laser diffraction method (ISO 13320:2020), while for suspension samples - using the dynamic light scattering method (ISO 22412:2017) [369].

The literature provides numerous chemical, physical and biological methods of producing ZnO nanostructures [73,76,86,154,169,170,171,172,173,174,270,307,370,371,372,373,374,375,376,377,378,379,380,381,382,383,384,385,386,387,388,389,390]. The following are most frequently enumerated methods of obtaining ZnO NMs [76]: co-precipitation with subsequent calcination, sol-gel method with subsequent calcination, mechanical synthesis combined with high-energy milling, hydrothermal synthesis and solvothermal synthesis. The unrepeatability of ZnO NMs properties arises above all from the limitations of the synthesis methods, unrepeatability of the reactants used and complexity of chemical reactions. The main obstacle in obtaining NMs with intended physicochemical properties is the lack of technologies that ensure the simultaneous control of, among others, average particle size, particle size distribution, shape, phase purity, dopant content, and particle agglomeration and aggregation. The synthesis of nanomaterials is currently at a new stage of development [391,392], at which the main criterion of the approach to obtaining ZnO NMs is understanding the synthesis mechanisms [393,394,395,396,397,398,399,400,401,402]. The familiarity with mechanisms of ZnO NMs synthesis allows obtaining repeatable and reproducible ZnO nanostructures.

**Figure 6:**
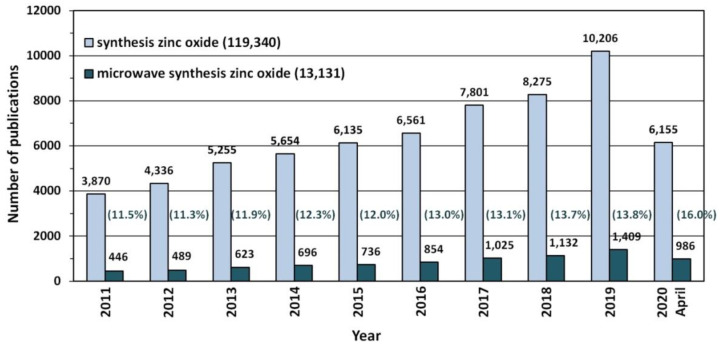
The number of scientific publications referring to the search of “synthesis zinc oxide” and “microwave synthesis zinc oxide” phrases published in the period of 2011–2020. Source: ScienceDirect ScienceDirect (accessed on 19 April 2020).

Nowadays, when the humans are fully aware of their harmful environmental impact [403,404] and of the climate change on the planet [405], particular attention is drawn to technologies of obtaining such nanomaterials that are environment friendly. One of the most interesting dynamically developing “green technologies” of ZnO nanostructure syntheses is the “microwave assisted synthesis”, which is proved, e.g., by the constant trend of the growing number of publications concerning microwave synthesis of ZnO (Figure 6). At present, the number of scientific papers describing the microwave synthesis of ZnO accounts for ca. 10.9% of the number of all hitherto published papers describing the synthesis of ZnO, while the annual percentage share in 2020 was as high as ca. 16.0%. It must be noted that microwave synthesis is also commonly and willingly employed for obtaining different nanomaterials, e.g., gold (Au) [406,407], silver (Ag) [406,407,408], rhodium (Rh) [406,407], copper (Cu) [406,407], hydroxyapatite (HAp) [409], zirconium dioxide (ZrO_2_) [410,411,412], titanium dioxide (TiO_2_) [413], silicon oxide (SiO_2_) [414], cerium dioxide (CeO_2_) [415], tin oxides (SnO and SnO_2_) [416], and nanocomposites (i.a. ZrO_2_-AlO(OH) [417,418], MoS_2_- polydopamine-Ag [419]).

## 2. Microwave Heating

**Figure 7:**
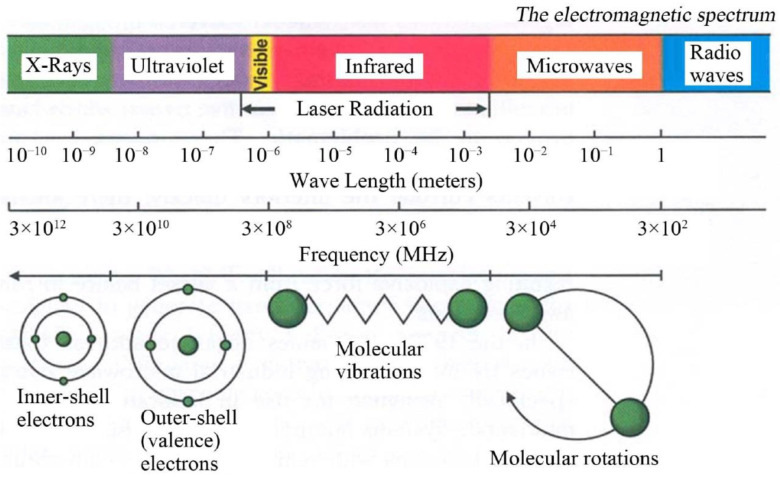
The electromagnetic spectrum showing characteristics of microwaves. Reprinted with permission from [428], Copyright © 2014 Rana et al. and Open Access Library Journal, Article licensed under a CC BY 4.0, https://creativecommons.org/licenses/by/4.0/.

Microwaves constitute the part of the electromagnetic spectrum with the wavelength (*λ*) between 1 mm and 1 m (Figure 7), which corresponds to the frequency range between 300 MHz (*λ* = 1 m) and 300 GHz (*λ* = 1 mm) [420,421]. Generally, microwaves are commonly used for commercial and military purposes, namely in cellular, Internet, and satellite communications, in radar technologies (radiolocation and radio navigation) and in the food industry (heating and drying). The International Telecommunication Union (ITU) [422] introduced the official division of the microwave range by frequency for the first time in 1953 [423,424]. ITU periodically organises World Radiocommunication Conferences (WRC) [425], during which legal radio regulations, i.e., international agreements controlling the use of the radio frequency spectra, are reviewed and corrected as necessary. In accordance with international agreements, some frequencies were assigned for industrial, scientific and medical purposes to avoid interferences in telecommunication. In each country, a dedicated governmental department deals with the assignment of frequencies and the frequency range as well as the terms and conditions of their use; in Poland, this role is fulfilled by the Ministry of Infrastructure [426]. The division of frequencies for industrial and scientific purposes is presented, e.g., by Torgovnikov [427]. Given the fact of the frequent use of microwave radiation in telecommunication, its individual frequencies were assigned to specific fields, e.g., microwave ovens and equipment employed at a laboratory were assigned the frequency of 2.45 GHz, which corresponds to the wavelength of ca. 12.25 cm. 

**Figure 8:**
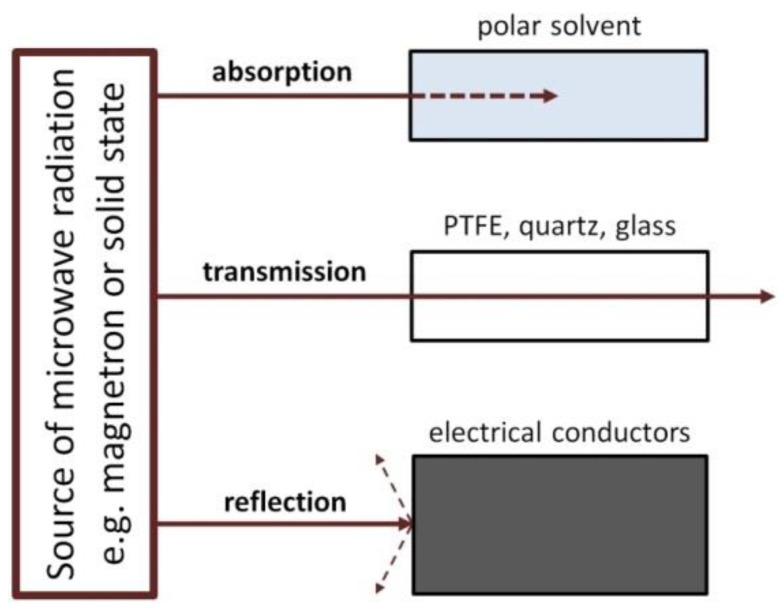
Interaction of different substances with microwaves: absorbing materials (e.g., polar solvents), insulation materials (e.g., PTFE, quartz, glass) and electrical conductors (e.g., metals).

The interaction of the microwave radiation with a substance may be divided into three types (Figure 8): absorption, transmission and reflection. Polar substances (e.g., solvents: H_2_O, ethylene glycol) absorb microwave radiation, as a result of which they are heated. Non-polar substances may display low interactions with microwave radiation, for which they are a microwave transparent medium. The third type includes substances (electrical conductors, e.g., metals) which reflect the microwave radiation from their surface. 

**Figure 9:**
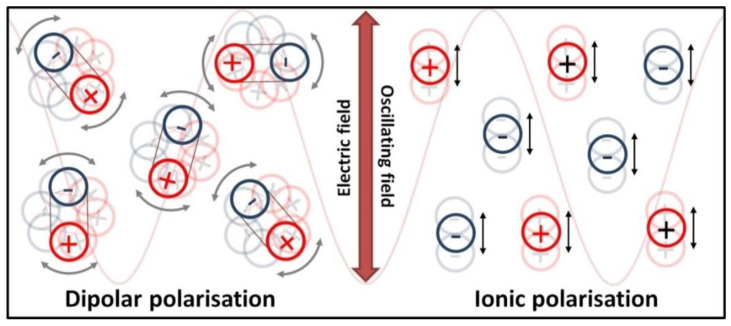
Dielectric heating schematic illustration of dipolar polarisation mechanism (dipoles align in the microwave field) and ionic conduction mechanism (ions move in the microwave field).

**Figure 10:**
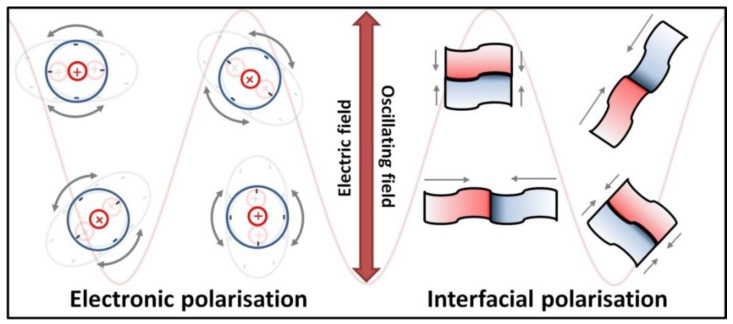
Dielectric heating schematic illustration of the electronic polarisation mechanism (movement of charges in the microwave field) and the interfacial mechanism (accumulation of charges on the interface between two different surfaces).

The heating of substances by microwave radiation has already been thoroughly described in the literature by numerous scholars [428,429,430,431,432,433,434,435,436,437,438,439,440,441,442,443,444,445,446,447,448,449,450,451,452,453,454,455,456,457]. Microwave heating (MH) is based on the transformation of electromagnetic field energy stemming from microwave radiation into kinetic energy (heat) by interacting with the polar particles of the material. MH arising from the electric component of electromagnetic radiation may occur in four ways (Figure 9 and Figure 10): by rotation of dipoles (dipolar polarisation), ionic conduction (ionic polarisation), electronic polarisation (atomic polarisation), and interfacial polarisation (surface polarisation).

The first mechanism of MH consists of the rotation of dipoles having a dipole moment, which try to position themselves in line with the direction and sense of the variable electromagnetic field, which imposes their motion. During the rotation, the microwave radiation energy is converted into kinetic energy, which is transferred between the particles that collide and rub against each other. The result is a uniform spreading of heat in the heated material and a rapid increase in temperature. The second mechanism of MH based on ionic conduction concerns systems (solutions, suspensions) containing ions. Ions present in the microwave field move in line with the direction of the variable electric field. The collision of migrating ions with those moving in the opposite direction causes a heating effect, which is the stronger the higher the concentration and mobility of ions are. The third mechanism of MH constitutes the induction of a dipole moment by shifting the centre of the electron charge in relation to the nucleus. The fourth mechanism of MH is based on the polarisation of a material in the microwave field by accumulation of charges on the interface surface (particle boundaries, phase boundaries), i.e., partial charging of the surface (induced surface charges) through the action of the microwave radiation.

The capability of a substance (solvent) in the conditions of being radiated with microwaves at a given frequency and temperature of converting the microwave radiation energy is determined by the so-called “loss factor” or “loss angle” or “loss tangent”, tanδ. Loss factor is expressed as $\tan\delta =\frac{\varepsilon^{\prime \prime }}{\varepsilon^{\prime }}$, where $\varepsilon^{\prime \prime }$ is the dielectric loss (F·m^−1^), which is indicative of the efficiency with which electromagnetic radiation is converted into heat and ε′ is the dielectric constant (F·m^−1^), which is indicative of the polarisability of molecules in the electric field [428]. The higher the tanδ value, the more efficient the heating of a given substance by microwave radiation will be. The tanδ and ε values depend on the electromagnetic radiation frequency and temperature. The tanδ values of popular pure solvents are presented in Table 2. The heating capability (tanδ) of solvents also depends on the substances it contains. The following classification of solvents was adopted depending on tanδ value:

- low microwave absorbing, where tanδ value <0.1
- medium microwave absorbing, where tanδ value ranges from 0.1 to 0.5
- high microwave absorbing, where tanδ value is higher than 0.5.

Generally, microwaves are capable of heating only such solvents with particles being dipoles, e.g., water (H_2_O), methanol (CH_3_OH), ethanol (C_2_H_5_OH), ethylene glycol (C_2_H_4_(OH)_2_), diethylene glycol ((C_2_H_4_OH)_2_O), dimethylformamide ((CH_3_)_2_NCHO), dimethylsulfoxide ((CH_3_)_2_SO), ethyl acetate (CH_3_COOC_2_H_5_), chloroform (CHCl_3_), methyl chloride (CH_2_Cl_2_), acetic acid (CH_3_COOH), acetonitrile (CH_3_CN), and acetone ((CH_3_)_2_CO). Liquid (and solid) substances which do not have particles with the dipole moment and electric charge carriers do not absorb microwave radiation and do not heat. Therefore, such solvents as hexane (C_6_H_14_), benzene (C_6_H_6_), toluene (C_6_H_5_CH_3_), diethyl ether (C_2_H_5_)_2_O, tetrachloromethane (CCl_4_), tetrahydrofuran (C_4_H_8_O), or polytetrafluoroethylene (PTFE) oil virtually do not heat under the influence of microwave radiation or heat insufficiently. Our experience shows that even a small quantity of an impurity that has a dipole moment and is present in a non-polar solvent may contribute to a considerable improvement of the solvent heating under the influence of microwave radiation.

It must be remembered that during the interaction of microwave radiation with unearthed metals, electric charge carriers (electrons) move under the influence of the electric component of radiation, which leads to metal polarisation. Therefore, unearthed metal subject to microwave radiation will heat intensively and the charges generated on its surface will cause electric discharges and sparking.

### 2.1. Comparison of Conventional Heating with Microwave Heating

**Figure 11:**
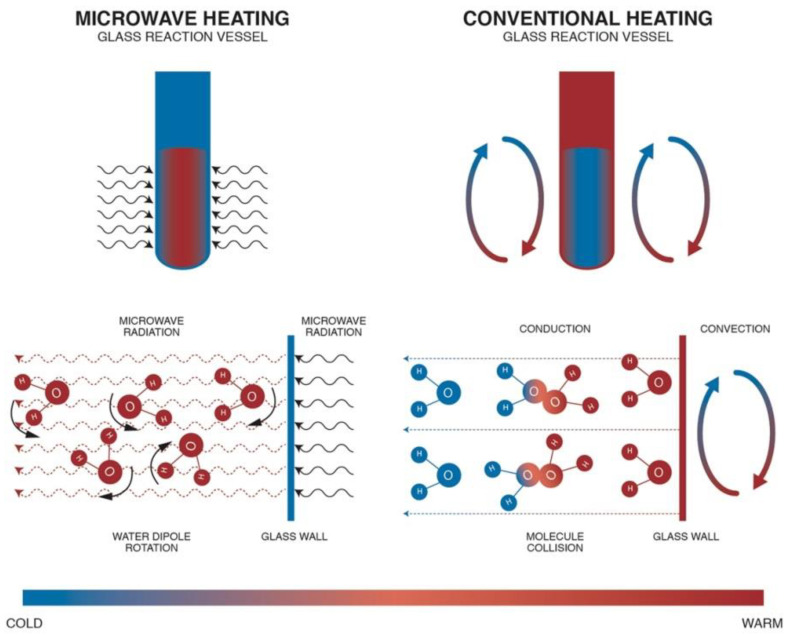
Comparison of conventional heating with microwave heating on the example of heating a water sample. Reprinted with permission from [460], Copyright ©2018 Sweygers et al., Article licensed under a CC BY 4.0, https://creativecommons.org/licenses/by/4.0/.

Conventional methods of obtaining nanomaterials employ direct resistance heating and indirect resistance heating. In direct resistance heating, current flows directly through the feedstock itself, causing its heating. This type of heating is not popular because it mostly requires a high current to flow through the feedstock, which flows through the contacts connecting the power source with the feedstock but this causes a range of complications. Indirect resistance heating uses a heat source, i.e., a heating element (e.g., an electric heater). There are two possibilities of applying the heating element: at the direct contact with the feedstock (e.g., electric kettle) or at the indirect contact, where the heating element is in contact with the vessel walls and with the feedstock (e.g., heating mantle or heating jacket). The application of a heating element directly in the feedstock is not popular, which results mainly from heavy contamination caused by corrosion of heater walls and from accumulation of solid synthesis products on the heater surface. Heating jackets are used in the majority of conventional chemical reactors. It must be borne in mind that during conventional heating the heating jacket first heats the walls of a reaction chamber/vessel and subsequently the reaction feedstock, which is shown in Figure 11. 

Conventional heating of the reactor feedstock involves the following disadvantages:

(a) long heating time, which depends on the thermal conduction of the material of which the reaction chamber walls are made;
(b) temperature maximums occur on the reaction vessel/chamber wall surface, which is one of the direct causes of the heterogeneity of the obtained products (so-called wall effect);
(c) limited reaction control caused by a high thermal inertia of the system, which results from the heating of the heating jacket and the reaction chamber walls;
(d) difficulties involved in the speed of the feedstock cooling process;
(e) high heat losses.

**Figure 12:**
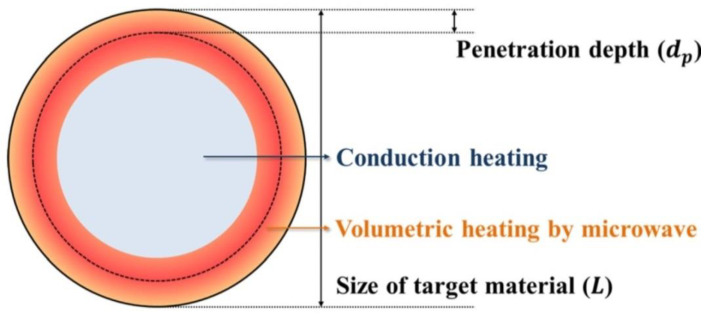
Schematic illustration of microwave heating phenomenon when the size of the target material is much larger than the microwave penetration depth. Reprinted with permission from [456], Copyright ©2014 Korean Carbon Society, Article licensed under a CC BY-NC 3.0, http://creativecommons.org/licenses/ by-nc/3.0/.

MH is characterised by shorter heating times in relation to the conventional method. With optimum parameters (MH power, resonance structure), feedstock may be heated rapidly and uniformly due to the direct molecular heating by the energy of microwaves. MH is defined as endogenous or volumetric heating [461], which means that heat may be generated within the whole precursor mass (sample inside) rather than transferred from an external heat source (Figure 11). However, it must be borne in mind that microwave radiation does not always have to heat the whole sample volume. When electromagnetic radiation is cast on the surface of a given substance, a part of the radiation may reflect from the surface while the remaining part may penetrate inside the (volume of the) substance. Electromagnetic radiation which penetrated inside interacts with substance molecules and ions. Depending on the properties of a given substance, electromagnetic radiation may penetrate a given substance at various depths, which is illustrated in Figure 12. Penetration depth is the key parameter of each substance in the process of microwave radiation heating. The penetration depth of a field is defined as the distance from the surface to a certain internal point where the magnitude of field strength decreases to 1/e (=36.8%) of the original magnitude at the surface [462]. This parameter is described by the following formula [437]:

$$d_{p}=\frac{\lambda_{0}}{2\pi }{\left(\frac{1}{2\mu \varepsilon_{0}\varepsilon^{\prime }}\right)}^{1/2}[{\left(1+{\left(\tan\delta \right)}^{2}{)}^{\frac{1}{2}}-1\right]}^{-1/2}$$

where $d_{p}$—penetration depth (cm), $\lambda_{0}$—wavelength at vacuum conditions (cm), $\mu_{0}$—magnetic permeability of free space (H·m^−1^), $\mu^{\prime }$—magnetic relative permeability (H·m^−1^), $\varepsilon_{0}$—permittivity in free space, (F·m^−1^), $\varepsilon^{\prime }$—relative permeability (F·m^−1^), and tanδ—loss angle. If considering, e.g., water, the penetration depth of the electromagnetic radiation with the frequency of 2.45 GHz at the temperature of 25 °C for deionised water is 2.88 cm, while for water with an addition of NaCl (0.5 M)—merely 0.45 cm [456] (Table 3). When the size of the heated substance is greater than the microwave penetration depth, only a part of the volume of this substance is heated, which is illustrated in Figure 12. 

Reaction vessels of microwave reactors are mostly made of polytetrafluoroethylene (PTFE, one of the trade names is Teflon®), which is characterised by a low thermal conductivity (0.25 W/(m·K)) and acts as a thermal insulator, thanks to which a low temperature gradient is maintained in the reaction chamber [461,466,467]. Compared with conventional heating, MH has the following advantages:

(a) No direct contact of heat source with heated material (contactless method).
(b) Minimisation of the “wall effect” because the wall of the vessel (reaction chamber) is not heated directly.
(c) Volumetric heating of the feedstock.
(d) Instantaneous and precise electronic control. Quick heating switching on and off, e.g., heating process can be controlled with the accuracy of 1 s, namely after switching off the magnetron power unit, the heat source supply is interrupted immediately.
(e) Rapid heating with preservation of low thermal gradients (rapid energy transfer) [468].
(f) Heating uniformity (which is translated into the quality of obtained products, e.g., homogeneity and narrow size distribution of obtained nanomaterials [437,438,439,440,441,469,470]).
(g) Shorter duration of syntheses/processes. Microwave synthesis, depending on type of organic compound obtaining, may be 5–2500 times quicker than in the case of the same syntheses carried out by conventional thermal methods [429,433,471].
(h) Fewer side reactions [472,473].
(i) Selectivity and purity of generated products [432,474].
(j) Easy to conduct under solvent-free conditions [451].
(k) Very high power densities developed in the processing zone [452].
(l) Superior moisture levelling [452].
(m) Energy saving [467].
(n) Higher production efficiency (faster throughputs) [452].
(o) Lower apparatus size (compact equipment) [452].
(p) Shorter time of apparatus start-up.
(q) A green chemistry approach [475,476,477,478,479,480].

### 2.2. Application of Microwave Heating, Chemical Microwave Apparatus

Microwave heating is used for various applications, among others:

(a) food processing (np. home cooking, pasteurisation, drying) [445,446,447];
(b) industrial application (np. drying, wood curing, rubber curing and vulcanisation, disinfection, coal pre-treatment and processing, ceramic processing, polymer processing, polymeric composites, ceramic composites, melting of glasses, melting of metallic materials, roasting of tea/coffee beans, plant extraction processes) [442,447,448,449,450,451,481,482,483],
(c) waste treatment (np. medical waste, garbage, sludge) [447];
(d) medical applications (np. sterilisation, drying, diagnosis, novel methods for treating inoperable tumours) [447,484];
(e) analytical chemistry (laboratory sample processing, ashing, digestion, extraction, moisture analysis) [432,485];
(f) chemical reactions: organic and inorganic synthesis (microwave assisted synthesis of e.g., medications, polymers and nanomaterials) [406,407,429,430,431,432,433,434,435,436,437,438,439,440,441,442,443,444,447,455,475,476,477,478,479,480,486,487,488,489,490,491,492,493,494,495].

**Figure 13:**
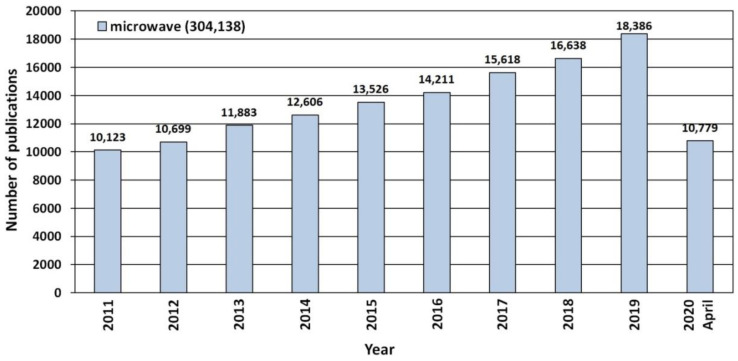
The number of scientific publications referring to the search of “microwave” phrases published in the period of 2011–2020. Source: ScienceDirect (accessed on 19 April 2020).

Recent years have seen a noticeable constant trend of growing popularity of microwave technologies, which is proved e.g., by the increase in the number of publications that were published in the period 2011–2020 (Figure 13). According to the scientific search engine ScienceDirect, 304,138 scientific publications have been released so far which contain a reference to the keyword “microwave” (Figure 13).

The main reason for the increased interest in microwave technologies, which is visible particularly in chemical laboratories, is the dynamic development of microwave apparatus, in particular chemical reactors [428,461,496,497,498,499,500,501], and the decrease in apparatus prices. The use of microwave reactors involves a range of advantages, which are already discussed in the previous Section 2.1 describing the advantages of microwave heating. Control and measurement technologies have reached a very high development level over the past 25 years. Very difficult to control at the beginning of the 21st century, microwave devices are currently equipped with advanced control systems and their operation parameter repeatability matches older and well developed technologies. Microwave chemical reactors of a new generation are characterised by a high repeatability of processes thanks to, among others, the optimisation of reactor structure (batch or flow type) in terms of applied materials, shape and size of the reaction chamber/vessel, recess structure (single synthesis or parallel synthesis), classes of microwave applicators (monomode or multimode) and automation. The capability of monitoring of the real course of reaction (temperature (*T*), pressure (*P*)) in microwave chemical reactors is crucial for meeting the requirements of chemical product generation, e.g., in the pharmaceutical industry. New reactor designs enable the rapid cooling of products, e.g., through adiabatic opening of the reactor chamber, which enables freezing the course of reaction over just a few seconds after the finish of the heating process [461,502]. Microwave reactors can always experience a failure, just like other ones [503]. It must be emphasised that microwave ovens, which are often used for conducting chemical reactions, are not professional equipment. Processes performed in microwave ovens are characterised by unrepeatability, which is often related to:

- random setting of the reaction vessel,
- random geometry of the reaction vessel (shape and size),
- impossibility to monitor the course of the process (temperature (*T*), pressure (*P*)).

**Figure 14:**
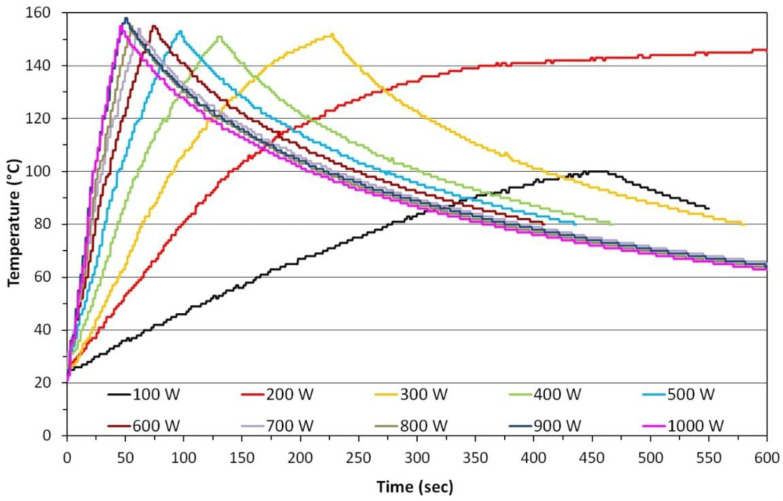
Kinetics of heating of 70 mL of distilled water, depending on the power emitted by the magnetron. Source: Experimental data achieved in the MSS3 reactor (IHPP PAS).

One of the main reasons why microwave chemical reactors are popular is their heating speed. Figure 14 shows the kinetics of distilled water heating in an experimental reactor, MSS3, which was designed and constructed at the Laboratory of Nanostructures, Institute of High Pressure Physics, Polish Academy of Sciences (IHPP PAS). The MSS3 is a reactor intended exclusively for research applications with a reaction vessel made of quartz, which is cooled continuously. The selection of the appropriate microwave power (Figure 14) enables the precise control of kinetics of feedstock heating, which, as was proven later, permits the application of unique features to the synthesised nanomaterials (ZnO NPs) [502]. Differences between the dynamics of heating and cooling are perfectly visible in the chart (Figure 14). It is easier to heat a material because energy is supplied by the power of the microwave field, while cooling takes place through thermal conduction and this process is limited by the thermal conduction of the applied materials. Table 4 presents times after which the water sample reached the temperature of 100 °C and 140 °C depending on the applied microwave power. 

## 3. Microwave Hydrothermal Synthesis of ZnO

The hydrothermal synthesis was defined in the literature [504] as a process in a closed system, in which chemical reactions take place exclusively in a water solvent at increased temperatures, at a pressure that is higher than atmospheric pressure (*P* > 101,325 Pa). This definition is often modified by scholars, e.g., Byrappa and Yoshimura [505] proposed a definition of the “hydrothermal reaction” as any heterogeneous chemical reaction in the presence of a solvent (whether aqueous or non-aqueous) above the room temperature and at a pressure greater than 1 atm in a closed system. For the purposes of this review, we assume that the “hydrothermal synthesis” is a process occurring in an aqueous environment (where mH_2_O > 50%), with the pressure equal to or higher than atmospheric pressure. In hydrothermal processes, reaction products are mainly oxides and salts due to the properties of water as a solvent. The results of the literature review concerning microwave hydrothermal synthesis of ZnO are divided into the following subgroups:

(1) Microwave hydrothermal synthesis of ZnO nanostructures without any additional heat treatment, where the literature review results [506,507,508,509,510,511,512,513,514,515,516,517,518,519,520,521,522,523,524,525,526,527,528,529,530,531,532,533,534,535,536,537,538,539,540,541,542,543,544,545,546,547,548,549,550,551,552,553,554,555,556,557,558,559,560,561,562,563,564,565,566,567,568,569,570,571,572,573,574,575,576,577,578,579,580,581,582,583,584,585,586,587,588,589,590,591,592,593,594,595,596,597,598,599,600,601,602,603,604,605,606,607,608,609,610,611,612,613,614,615,616,617,618,619,620,621,622,623,624,625,626,627,628,629,630,631,632,633,634,635,636,637,638,639] are summarised in Table 5.
(2) Microwave hydrothermal synthesis of ZnO nanostructures with additional heat treatment, where the literature review results [585,640,641,642,643,644,645,646,647,648,649,650,651,652,653,654,655,656,657,658,659,660,661,662,663,664,665,666,667,668,669,670,671,672,673] are summarised in Table 6.
(3) Microwave hydrothermal synthesis of ZnO nanocomposites or ZnO hybrid nanostructures without any additional heat treatment, where the literature review results [674,675,676,677,678,679,680,681,682,683,684,685,686,687,688,689,690,691,692,693,694,695,696,697,698,699,700,701,702,703,704,705,706,707,708,709,710,711,712,713,714,715,716,717,718,719,720,721,722,723] are summarised in Table 7.
(4) Microwave hydrothermal synthesis of ZnO nanocomposites or ZnO hybrid nanostructures with additional heat treatment, where the literature review results [724,725,726,727,728,729,730,731,732,733,734,735,736,737,738] are summarised in Table 8.

### 3.1. Reactants

**Figure 15:**
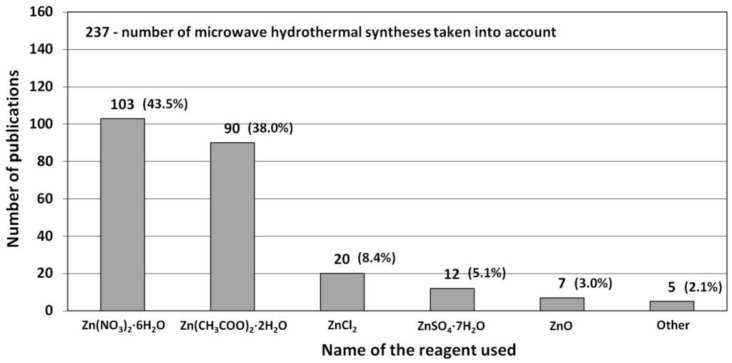
Statistics concerning the use of reactants (Zn^2+^ salts) in the microwave hydrothermal synthesis of ZnO. Source: [506,507,508,509,510,511,512,513,514,515,516,517,518,519,520,521,522,523,524,525,526,527,528,529,530,531,532,533,534,535,536,537,538,539,540,541,542,543,544,545,546,547,548,549,550,551,552,553,554,555,556,557,558,559,560,561,562,563,564,565,566,567,568,569,570,571,572,573,574,575,576,577,578,579,580,581,582,583,584,585,586,587,588,589,590,591,592,593,594,595,596,597,598,599,600,601,602,603,604,605,606,607,608,609,610,611,612,613,614,615,616,617,618,619,620,621,622,623,624,625,626,627,628,629,630,631,632,633,634,635,636,637,638,639,640,641,642,643,644,645,646,647,648,649,650,651,652,653,654,655,656,657,658,659,660,661,662,663,664,665,666,667,668,669,670,671,672,673,674,675,676,677,678,679,680,681,682,683,684,685,686,687,688,689,690,691,692,693,694,695,696,697,698,699,700,701,702,703,704,705,706,707,708,709,710,711,712,713,714,715,716,717,718,719,720,721,722,723,724,725,726,727,728,729,730,731,732,733,734,735,736,737,738].

The most popular reactants of the “Zn^2+^” zinc cation precursor used for ZnO synthesis according to the data derived from the literature review [506,507,508,509,510,511,512,513,514,515,516,517,518,519,520,521,522,523,524,525,526,527,528,529,530,531,532,533,534,535,536,537,538,539,540,541,542,543,544,545,546,547,548,549,550,551,552,553,554,555,556,557,558,559,560,561,562,563,564,565,566,567,568,569,570,571,572,573,574,575,576,577,578,579,580,581,582,583,584,585,586,587,588,589,590,591,592,593,594,595,596,597,598,599,600,601,602,603,604,605,606,607,608,609,610,611,612,613,614,615,616,617,618,619,620,621,622,623,624,625,626,627,628,629,630,631,632,633,634,635,636,637,638,639,640,641,642,643,644,645,646,647,648,649,650,651,652,653,654,655,656,657,658,659,660,661,662,663,664,665,666,667,668,669,670,671,672,673,674,675,676,677,678,679,680,681,682,683,684,685,686,687,688,689,690,691,692,693,694,695,696,697,698,699,700,701,702,703,704,705,706,707,708,709,710,711,712,713,714,715,716,717,718,719,720,721,722,723,724,725,726,727,728,729,730,731,732,733,734,735,736,737,738] (Table 5, Table 6, Table 7 and Table 8) are Zn(NO_3_)_2_·6H_2_O, Zn(CH_3_COO)_2_·2H_2_O, ZnCl_2_, ZnSO_4_·7H_2_O and ZnO (Figure 15), respectively. The popularity of these reactants results from their low price and easy availability. Reactants being a source of “Zn^2+^” ions are commonly produced at a large scale mostly by several producers in each developed country. It should be emphasised that ZnCl_2_ is one of the problematic reactants, which arises from the possibility that during the ZnO synthesis a stable by-product (impurity), called simonkolleite (Zn_5_(OH)_8_Cl_2_·H_2_O), is formed. If Zn(NO_3_)_2_·6H_2_O is used as the reactant and if the temperature of the ZnO synthesis is too low (below 100 °C), one of the synthesis by-products (impurity) may be the salt Zn_5_(OH)_8_(NO_3_)_2_·2H_2_O [515]. It must be borne in mind that ZnO obtained by the hydrothermal method, depending on the parameters employed (*T*, *P*, pH), may contain impurities being hydroxides, oxide hydroxides or basic salts. Depending on the quantity and size of crystallites of “impurities”, they are often invisible in the results of the X-ray powder diffraction analysis (XRD) because they are below the method’s detection limit. When discussing the results, it must be remembered that the XRD analysis is capable of indicating only crystalline impurities of ZnO.

**Figure 16:**
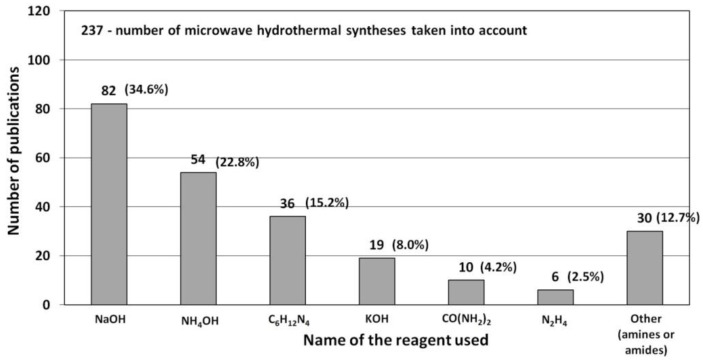
Statistics concerning the use of reactants (OH^−^) in the microwave hydrothermal synthesis of ZnO. Source: [506,507,508,509,510,511,512,513,514,515,516,517,518,519,520,521,522,523,524,525,526,527,528,529,530,531,532,533,534,535,536,537,538,539,540,541,542,543,544,545,546,547,548,549,550,551,552,553,554,555,556,557,558,559,560,561,562,563,564,565,566,567,568,569,570,571,572,573,574,575,576,577,578,579,580,581,582,583,584,585,586,587,588,589,590,591,592,593,594,595,596,597,598,599,600,601,602,603,604,605,606,607,608,609,610,611,612,613,614,615,616,617,618,619,620,621,622,623,624,625,626,627,628,629,630,631,632,633,634,635,636,637,638,639,640,641,642,643,644,645,646,647,648,649,650,651,652,653,654,655,656,657,658,659,660,661,662,663,664,665,666,667,668,669,670,671,672,673,674,675,676,677,678,679,680,681,682,683,684,685,686,687,688,689,690,691,692,693,694,695,696,697,698,699,700,701,702,703,704,705,706,707,708,709,710,711,712,713,714,715,716,717,718,719,720,721,722,723,724,725,726,727,728,729,730,731,732,733,734,735,736,737,738].

The most popular reactants being hydroxide anion precursor chemicals “OH^−^” used for the ZnO synthesis according to the data derived from the literature review [506,507,508,509,510,511,512,513,514,515,516,517,518,519,520,521,522,523,524,525,526,527,528,529,530,531,532,533,534,535,536,537,538,539,540,541,542,543,544,545,546,547,548,549,550,551,552,553,554,555,556,557,558,559,560,561,562,563,564,565,566,567,568,569,570,571,572,573,574,575,576,577,578,579,580,581,582,583,584,585,586,587,588,589,590,591,592,593,594,595,596,597,598,599,600,601,602,603,604,605,606,607,608,609,610,611,612,613,614,615,616,617,618,619,620,621,622,623,624,625,626,627,628,629,630,631,632,633,634,635,636,637,638,639,640,641,642,643,644,645,646,647,648,649,650,651,652,653,654,655,656,657,658,659,660,661,662,663,664,665,666,667,668,669,670,671,672,673,674,675,676,677,678,679,680,681,682,683,684,685,686,687,688,689,690,691,692,693,694,695,696,697,698,699,700,701,702,703,704,705,706,707,708,709,710,711,712,713,714,715,716,717,718,719,720,721,722,723,724,725,726,727,728,729,730,731,732,733,734,735,736,737,738] (Table 5, Table 6, Table 7 and Table 8) (Figure 16) are sodium hydroxide (NaOH), ammonia water (NH_3_·H_2_O, NH_4_(OH)), hexamethylenetetramine (urotropin, HMTA, C_6_H_12_N_4_), potassium hydroxide (KOH), urea (CH₄N₂O, CO(NH_2_)_2_), hydrazine (N_2_H_4_·H_2_O) as well as other amides and amines, e.g., 1-n-butyl-3-methyl imidazolium tetrafluoroborate (C_8_H_15_BF_4_N_2_), N,N dimethylformamide (C_3_H_7_NO), pyridine (C_5_H_5_N), aniline (C_6_H_5_NH_2_), arginine (C_6_H14N_4_O_2_), tris (hydroxymethyl) aminomethane (C_4_H_11_NO_3_), bis(triaminomethyl) carbonate (C_3_H_12_N_6_O_3_), butyl-3-ethyl imidazolium tetrafluoroborate (C_8_H_15_BF_4_N_2_), dodecylamine (C_12_H_27_N), and 1-hexyl-2-ethyl-3-methylimidazoliumtetrafluoroborate (C_6_H_11_BF_4_N_2_).

The chemical properties of amines are similar to those of ammonia. These are compounds with alkaline properties, which form salts with acids and produce an alkaline reaction in aqueous solutions. The alkalinity of amines depends on substituents by the nitrogen atom. Aliphatic amines are, as a rule, more alkaline than ammonia while the alkaline properties of aromatic amines are weaker than those of ammonia. Amides undergo a slow hydrolysis, which leads to the formation of OH^−^ and the formation of an intermediate, Zn(OH)_2_.

### 3.2. Surfactants

Surfactants are commonly used in nanotechnology for passivation of surface, control of shape, and giving of photophysical properties [740]. Similar impact on properties of nanomaterials, apart from various types of surfactants (anionic, nonionic, cationic and amphoteric), is exerted also by surface active polymers, amines, amino acids, proteins, and carbohydrates. One of the primary purposes of the use of surfactants in obtaining nanoparticles is their strong contribution to obtaining dispersoids (emulsions, suspensions, colloids), in which they counteract the agglomeration of NPs [741,742,743]. Surfactants enable achieving a dispersion of NPs in non-polar solvents, e.g., in lubricating oils [744]. Surfactants are also used for controlling the shape of nanomaterials [740,745]. The shape control mechanism is related above all to the adsorption of the surfactant particles on various planes of crystalline nucleation centres of crystallites; these particles selectively promote the growth of these centres or to the contrary: inhibit their growth. The following surfactants were used in microwave hydrothermal syntheses of ZnO NMs for the purpose of controlling the morphology:

- Ethylenediamine (EDA, C_2_H_8_N_2_) for obtaining nanoneedles [525].
- Hexamethylenetetramine (HMT, (C_6_H_12_N_4_)) for obtaining nanorods [525].
- Triethyl citrate (C_12_H_20_O_7_) for obtaining hexagonal disks [525].
- Triethanolamine (TEA, C_6_H_15_NO_3_) for obtaining nanosheets [521], pompon-like spheres [554], peach nut-like spheres [554], misshapen spheres [554], rugby-like nanostructures [565], raspberry-like nanostructures [566], hollow nanospheres [566], dumbbell-like [626], football-like shape [626], and spherical nanoparticles [565,566,625,645].
- 1,2,4,5-benzenetetracarboxylic acid (BTCA, C_6_H_2_(CO_2_H)_4_) for obtaining nanosheet [554] and elongated triangles [554].
- Cetyltrimethylammonium bromide (CTAB, (C_19_H_42_BrN)) for obtaining heterogeneous shapes [531], wires [547], nanorods [576], wire-like architecture [597], flower-like microstructures composed of nanorods [597], and spike-like nanostructures [638].
- Sodium di-2-ethylhexyl-sulfosuccinate (C_20_H_36_Na_2_O_7_S) for obtaining hexagonal rods [581], hexagonal prismatic [581], bihexagonal rod-like structures [581], wire-like architecture [597], and flower-like microstructures composed of nanorods [597] and rods [597].
- Pluronic F127 (polyoxypropylene polyoxyethylene block copolymer) for obtaining heterogeneous shapes [531].
- Polyethylene glycol 400 (PEG400, C_2n_H_4n+2_O_n+1_) for obtaining nanorods [533], flowers [533], rod-like nanostructures [574], star-like nanostructures [574], particles with an irregular shape (plate and rod-like particles) [596], quasi-spherical shapes [620], flower-like structures [620], flower-like hierarchical structures [655], rod-like structures [673], and needle-like structures [673].
- Acetyl acetate (ACAC, (CH_3_CO)_2_O) for obtaining rod-like structures [644].
- Guanidinium carbonate ((CH_5_N_3_)_2_·H_2_CO_3_) for obtaining spherical nanoparticles [644] and flower-like ZnO [644].
- Polyvinyl alcohol 2000 (PVA2000, (C_2_H_4_O)_n_) for obtaining spherical nanoparticles [578].
- Polyvinyl Pyrrolidine (PVP, (C_6_H_9_NO)_n_) for obtaining star-like nanostructures [526,558].
- Triethyl citrate (C_12_H_20_O_7_) for obtaining disk- and nut-like structures [525].
- Tripotassium citrate for obtaining UFOs and balls-like structures [525].
- Arginine (C_6_H_14_N_4_O_2_) for obtaining rods and flowers [543].
- Albumen for obtaining whisker-like and rod-like nanostructures [585].
- Triton X-100 (C_14_H_22_O(C_2_H_4_O)_n_ (n = 9–10)) for obtaining rods (400–800 nm) and flower structures [594].

### 3.3. Morphology

The morphologies of ZnO NMs reported in the literature are presented in Table 5, Table 6, Table 7 and Table 8 [506,507,508,509,510,511,512,513,514,515,516,517,518,519,520,521,522,523,524,525,526,527,528,529,530,531,532,533,534,535,536,537,538,539,540,541,542,543,544,545,546,547,548,549,550,551,552,553,554,555,556,557,558,559,560,561,562,563,564,565,566,567,568,569,570,571,572,573,574,575,576,577,578,579,580,581,582,583,584,585,586,587,588,589,590,591,592,593,594,595,596,597,598,599,600,601,602,603,604,605,606,607,608,609,610,611,612,613,614,615,616,617,618,619,620,621,622,623,624,625,626,627,628,629,630,631,632,633,634,635,636,637,638,639,640,641,642,643,644,645,646,647,648,649,650,651,652,653,654,655,656,657,658,659,660,661,662,663,664,665,666,667,668,669,670,671,672,673,674,675,676,677,678,679,680,681,682,683,684,685,686,687,688,689,690,691,692,693,694,695,696,697,698,699,700,701,702,703,704,705,706,707,708,709,710,711,712,713,714,715,716,717,718,719,720,721,722,723,724,725,726,727,728,729,730,731,732,733,734,735,736,737,738] along with a description of their properties. ZnO NMs synthesised with the use of microwave hydrothermal synthesis were characterised by the following shapes: belts-like [671], bundle-like [655], brush-like [572], calthrop-like framework [561], candles [525], dandelion-like [518,612], disks [525], dumbbell-like [626,641], football-like structure [626], hexagonal column [521,629], hexagonal nanoring [521], hexagonal bi-pyramidal [626], hexagonal prisms [513,694] hexagonally shaped prismatic [528], hexagonal tubular [664], hollow structures [521], javelins [540], lamellar-like [692,699], misshapen spheres [554], peach nut-like spheres [554], petals [644], nuts-like [525], pompon-like spheres [554], raspberry-like nanostructures [566], rhombic [663], rugby-like nanostructures [565], spindles [518], sponge-like [580], spike-like [638], thruster vanes [518], tetrapod-like [631,670], tubes [514,515,571], whiskers [585], wires [518,523,534,539,547,646,711], flakes [520,586,587,592,622,648,663,664], plates [511,532,572,579,596,629,651,658,694,736], star-like [506,526,535,536,558,574,631,679,703], heterogeneous structures [507,524,528,531,630,637,665,666,668,672,720,730,731], flower-like [508,510,512,513,519,520,527,528,529,530,532,533,535,538,541,543,545,550,553,555,556,557,558,559,560,561,562,563,564,565,566,571,572,579,582,593,594,597,598,599,620,621,622,631,639,649,654,655,663,670,704,734,737,738], needle-like [510,521,525,551,556,588,588,629,655,663,673,716,729], sheets [521,544,551,552,575,577,583,585,588,627,650,686,707,714], spherical particles [511,535,536,551,560,565,566,578,585,599,614,615,623,625,628,634,635,636,638,639,645,648,649,674,680,681,682,683,684,685,702,706,712,718,721,727,735], and rods [473,509,511,517,518,519,525,527,529,533,538,541,542,543,548,549,553,556,557,559,562,563,567,568,570,573,574,576,581,583,584,585,589,590,591,592,593,594,595,596,597,599,600,601,602,603,604,605,606,607,608,609,610,611,612,613,616,617,618,619,624,629,631,632,633,640,642,643,644,649,653,655,658,659,661,663,664,670,673,678,693,696,697,700,701,705,709,710,723,725,726,732]. 

**Figure 17:**
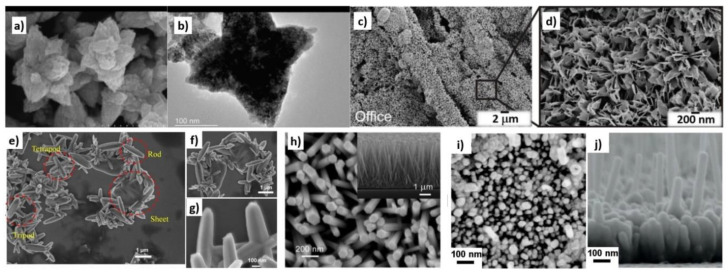
Examples of various ZnO structures obtained by the microwave hydrothermal synthesis: (**a**,**b**) Scanning electron microscopy (SEM) image and Transmission electron microscopy (TEM) image of Fe doped ZnO nanostars, respectively (Reprinted with permission from [679], Copyright ©2015 Kwong et al., Article licensed under a CC BY 3.0, https://creativecommons.org/licenses/by/3.0/); (**c**,**d**) SEM images of ZnO nanoplates grown on office papers (Reprinted with permission from [607], Copyright ©2019 Matias et al. Article licensed under a CC BY 4.0, https://creativecommons.org/licenses/by/4.0/); (**e**–**g**) SEM images of ZnO microstructures Reprinted with permission from [583], Copyright ©2013 Majithia et al. licensed under CC BY-NC-SA 3.0, https://creativecommons.org/licenses/by-nc-sa/3.0/); (**h**) SEM images of ZnO nanorods grown on a glass substrate (Reprinted with permission from [659], Copyright ©2016 Al-Sabahi et al., Article licensed under a CC BY 4.0, https://creativecommons.org/licenses/by/4.0/); (**i**,**j**) SEM image of ZnO nanorod arrays and SEM cross section image of ZnO nanorod arrays (Reprinted with permission from [617], Copyright ©2016 Pimentel, Article licensed under a CC BY 4.0, https://creativecommons.org/licenses/by/4.0).

**Figure 18:**
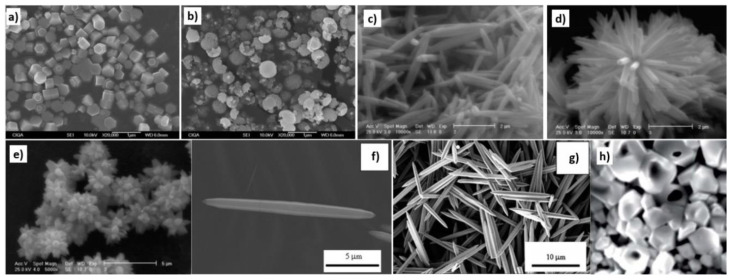
Examples of various ZnO structures obtained by the microwave hydrothermal synthesis: (**a**) SEM image of ZnO hexagonal columns; (**b**) SEM image of ZnO cauliflower-like morphology. ((**a**,**b**) Reprinted from [598], Copyright ©2015 The Authors, with permission from Elsevier [OR APPLICABLE SOCIETY COPYRIGHT OWNER]. Article licensed under a CC BY-NC-ND 4.0, https://creativecommons.org/licenses/by-nc-nd/4.0/); (**c**–**e**) SEM images of ZnO rods (Reprinted from [599], Copyright ©2015 The Authors, with permission from Elsevier [OR APPLICABLE SOCIETY COPYRIGHT OWNER]. Article licensed under a CC BY-NC-ND 4.0, https://creativecommons.org/licenses/by-nc-nd/4.0/); (**f**,**g**) SEM images of ZnO micro-javelin(s) (Republished with permission of Royal Society of Chemistry, from [540]; permission conveyed through Copyright Clearance Center, Inc. All rights reserved. In order to re-use permission must be obtained from the rightsholder.; (**h**) SEM image of hollow particles (Reprinted with permission from [672], Copyright ©The Royal Society of Chemistry, Article licensed under a CC BY-NC 3.0, https://creativecommons.org/licenses/by-nc/3.0/).

**Figure 19:**
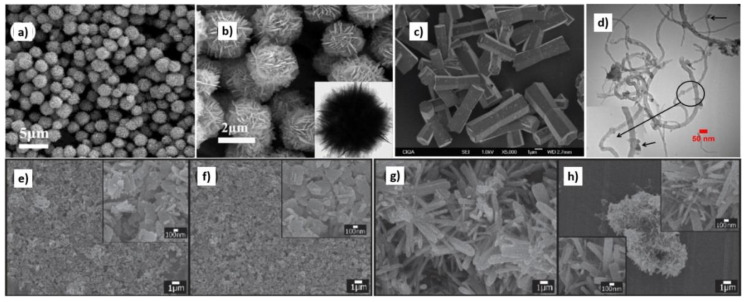
Examples of various ZnO structures obtained by the microwave hydrothermal synthesis: (**a**,**b**) SEM and TEM images of N-ZnO microflowers (Reprinted with permission from [615], Copyright ©2016 Ou et al., Article licensed under a CC BY 4.0, http://creativecommons.org/licenses/by/4.0/); (**c**) SEM image of ZnO bihexagonal rod-like (Reprinted with permission from [581], Copyright ©2013 Barreto et al., Article licensed under a CC BY 3.0, https://creativecommons.org/licenses/by/3.0/); (**d**) TEM image of Cd doped ZnO/CNT carbon nanotubes nanocomposites (Reprinted from [698], Copyright 2017 The Authors, with permission from Elsevier [OR APPLICABLE SOCIETY COPYRIGHT OWNER]. Article licensed under a CC BY-NC-ND 4.0, https://creativecommons.org/licenses/by-nc-nd/4.0/.); (**e**–**h**) SEM images of ZnO nanoplates and SEM images of nanostructure flowers, respectively (Reprinted with permission from [532], ©2019 The Ceramic Society of Japan, Article licensed under a CC BY-ND 4.0, https://creativecommons.org/licenses/by-nd/4.0/deed.en).

**Figure 20:**
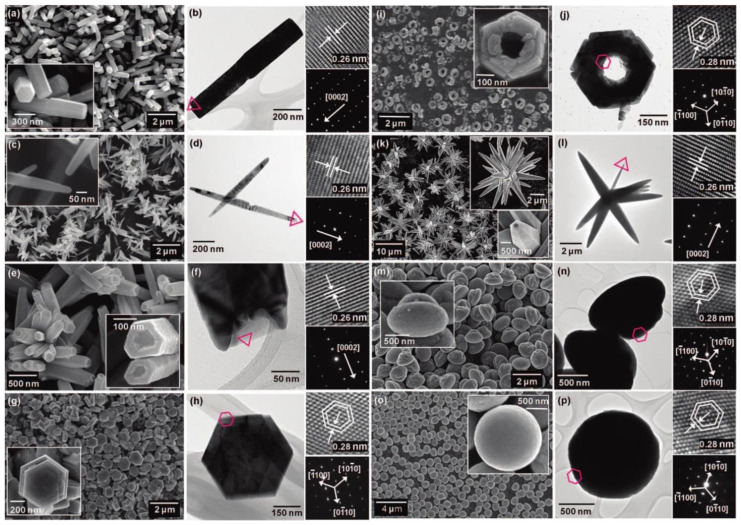
SEM (**left**) and TEM (**right**) images of the basic ZnO structures synthesised by the microwave irradiation: (**a**,**b**) nanorods, (**c**,**d**) nanoneedles, (**e**,**f**) nanocandles, (**g**,**h**) nanodisks, (**i**,**j**) nanonuts, (**k**,**l**) microstars, (**m**,**n**) microUFOs, (**o**,**p**) microballs. HRTEM images and SAED patterns were inserted as upper and lower insets in TEM images, respectively. The HRTEM images were obtained at the centre of the triangles or the hexagons in the TEM images. Reprinted (adapted) with permission from [525]. Copyright © 2008, American Chemical Society. All rights reserved. In order to re-use permission must be obtained from the rightsholder.

Examples of various morphologies of ZnO are presented in Figure 17, Figure 18, Figure 19 and Figure 20. The modification of morphology of ZnO NMs with a 3D architecture is extremely important e.g., for producing sensors with high sensing capabilities [739]. The 3D architecture of ZnO NMs, characterised by a small particle size, a high specific surface area and porosity, enables the improvement of the sensing response of gas sensors [152].

### 3.4. Microwave Hydrothermal Synthesis of ZnO without Any Additional Heat Treatment

The microwave hydrothermal synthesis of ZnO NMs owes its widespread use and popularity to water (H_2_O). This solvent is cheap, non-toxic and easy to dispose of. The main advantage of this solvent is that it can be easily separated from the product by e.g., centrifuging, drying with the use of a filter, in a vacuum dryer, or freeze-drying. Another advantage of water is that the majority of Zn^2+^ salts are soluble in it (among others: Zn(NO_3_)_2_·6H_2_O, Zn(CH_3_COO)_2_·*x*H_2_O, ZnCl_2_·*x*H_2_O, ZnSO_4_·7H_2_O). 

Considering dispersoids of the reaction mixture in the microwave hydrothermal synthesis of ZnO NMs, it is possible to perform the synthesis in a solution or in a suspension. It must be underlined that currently the synthesis of ZnO NMs has reached a new stage of development, where the common requirement is to control of properties of the obtained ZnO NMs, their repeatability and reproducibility. One of examples of papers which report, e.g., control of the ZnO shape is the publication by Wang et al. [510]. They demonstrate an example of obtaining ZnO with different morphologies by using an imidazolium salt 1-n-butyl-3-methyl imidazolium tetrafluoroborate ([BMIM]BF_4_) (an ionic liquid). The aqueous mixture of Zn(NO_3_)_2_·6H_2_O, NaOH and [BMIM]BF_4_ was used as the precursor solution. They obtained a strongly alkaline pH of the solution, where Zn^2+^ existed mainly as Zn(OH)_4_^2−^. The mechanism of the ZnO synthesis reaction proposed by Wang et al. [510] is as follows in Equations (1)–(4):

(1) $$\mathrm{Zn}{\left({\mathrm{NO}}_{3}\right)}_{2}\overset{H_{2}O\;}{↔}{\mathrm{Zn}}^{2+}+2{\mathrm{NO}}_{3}{}^{-}$$

(2) $$\mathrm{NaOH}\overset{H_{2}O\;}{↔}{\mathrm{Na}}^{+}+{\mathrm{OH}}^{-}$$

(3) $${\mathrm{Zn}}^{2+}+4{\mathrm{OH}}^{-}\overset{H_{2}O\;}{↔}\mathrm{Zn}{\left(\mathrm{OH}\right)}_{4}{}^{2-}$$

(4) $$\mathrm{Zn}{\left(\mathrm{OH}\right)}_{4}{}^{2-}\overset{MH\;}{\to }\mathrm{ZnO}↓+H_{2}O+2{\mathrm{OH}}^{-}$$

Wang et al. [510] controlled the change in the morphology from flower-like, to flower-like + needle-like and to needle-like by changing such parameters as concentration of reactants, temperature and synthesis duration.

Pulit-Prociak et al. [507] report the effect of process parameters on the size and shape of ZnO in their paper. They used Zn(NO_3_)_2_·6H_2_O and NaOH for the synthesis. The mechanism of the ZnO synthesis reaction proposed by Pulit-Prociak et al. [507] was as follows in Equations (5)–(8):

(5) $$\mathrm{Zn}{\left({\mathrm{NO}}_{3}\right)}_{2}\cdot 6H_{2}O\overset{H_{2}O\;}{↔}{\mathrm{Zn}}^{2+}+2{\mathrm{NO}}_{3}{}^{-}$$

(6) $$\mathrm{NaOH}\overset{H_{2}O\;}{↔}{\mathrm{Na}}^{+}+{\mathrm{OH}}^{-}$$

(7) $${\mathrm{Zn}}^{2+}+2{\mathrm{OH}}^{-}\overset{H_{2}O\;}{\to }\mathrm{Zn}{\left(\mathrm{OH}\right)}_{2}↓$$

(8) $$\mathrm{Zn}{\left(\mathrm{OH}\right)}_{2}\overset{MH\;}{\to }\mathrm{ZnO}↓+H_{2}O$$

Pulit-Prociak et al. [507] contributed to a change in the shape and size of aggregates of ZnO particles by changing the microwave power, synthesis duration and pressure.

Hu et al. [509] report obtaining ZnO rods using a water suspension composed of Zn(NO_3_)_2_·6H_2_O and (CH_2_)_6_N_4_ (hexamethylenetetramine, HMT) in their paper. The mechanism of the ZnO synthesis reaction proposed by Hu et al. [509] was as follows in Equations (9)–(12):

(9) $$C_{6}H_{12}N_{4}+6H_{2}O\overset{H_{2}O\;}{↔}4{\mathrm{NH}}_{3}+4\mathrm{HCHO}$$

(10) $${\mathrm{NH}}_{3}+H_{2}O\overset{H_{2}O\;}{↔}{\mathrm{NH}}_{4}{}^{+}+{\mathrm{OH}}^{-}$$

(11) $${\mathrm{Zn}}^{2+}+2{\mathrm{OH}}^{-}\overset{H_{2}O\;}{\to }\mathrm{Zn}{\left(\mathrm{OH}\right)}_{2}↓$$

(12) $$\mathrm{Zn}{\left(\mathrm{OH}\right)}_{2}↓\overset{MH\;}{\to }\mathrm{ZnO}↓+H_{2}O$$

Hu et al. [509] changed the morphology from flower-like, to flower-like + needle-like, to needle-like by changing such parameters as concentration of reactants, temperature and synthesis duration.

**Figure 21:**
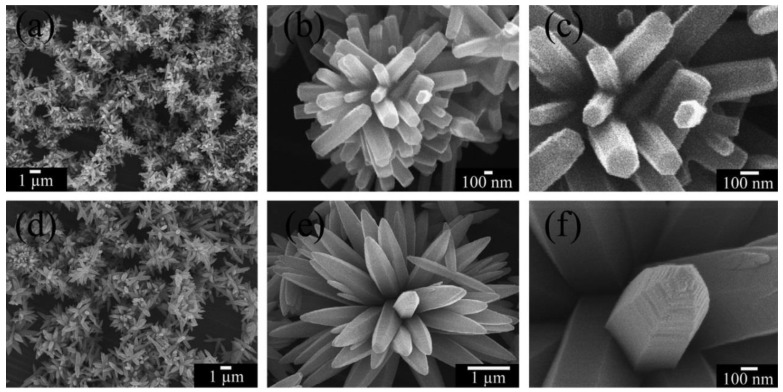
SEM images of ZnO synthesised in solutions containing Zn(NO_3_)_2_·6H_2_O and hexamethylenetetramine (HMT) at pH (**a**–**c**) 9 and (**d**–**f**) 13. Reprinted from [513], Copyright (2014), with permission from Elsevier [OR APPLICABLE SOCIETY COPYRIGHT OWNER]. All rights reserved. In order to re-use permission must be obtained from the rightsholder.

Phuruangrat et al. [513] report the control of morphologies and a growth mechanism of hexagonal prisms with planar and pyramid tips of ZnO microflowers. They used Zn(NO_3_)_2_·6H_2_O, NaOH, and hexamethylenetetramine (HMT) for the synthesis. Figure 21 presents the as-synthesised products which were pure hexagonal wurtzite ZnO microstructured flowers of hexagonal prisms with planar tips at pH 9 and of hexagonal prisms with hexagonal pyramid tips at pH 13.

Phuruangrat et al. [513] explained the formation of ZnO microflowers composed of petals in the shapes of hexagonal prisms with planar and pyramid as follows: the size and morphology of particles is controlled by the chemical state of Zn^2+^ ions in the solution. Thermodynamic and kinetic factors affect the process of precipitation of compounds containing Zn^2+^ in various ways. The chemical state of Zn^2+^ ions (dissociated form, dissociated complex form, sediment) strongly depends on the pH of the medium and may be strongly controlled by the pH of the solution and anionic type. By adding the NaOH solution gradually to the Zn(II) salt solution with the pH of 13, the Zn(OH)_4_^2−^ complex is formed, which can be denoted by in Equations (13)–(16):

(13) $$\mathrm{Zn}{\left({\mathrm{NO}}_{3}\right)}_{2}\cdot 6H_{2}O\overset{H_{2}O\;}{↔}{\mathrm{Zn}}^{2+}+2{\mathrm{NO}}_{3}{}^{-}$$

(14) $$\mathrm{NaOH}\overset{H_{2}O\;}{↔}{\mathrm{Na}}^{+}+{\mathrm{OH}}^{-}$$

(15) $${\mathrm{Zn}}^{2+}+4{\mathrm{OH}}^{-}\overset{H_{2}O\;}{↔}\mathrm{Zn}{\left(\mathrm{OH}\right)}_{4}{}^{2-}$$

(16) $$\mathrm{Zn}{\left(\mathrm{OH}\right)}_{4}{}^{2-}\overset{MH\;}{\to }\mathrm{ZnO}↓+H_{2}O+2{\mathrm{OH}}^{-}$$

Due to a large number of growth units, [Zn(OH)_4_]^2−^ complexes are able to easily adsorb on different sites of ZnO nanorods. The positively charged Zn-(0001) surfaces are the most reactive. Thus OH^−^ ions may stabilise the positive charge of the Zn-(0001) surfaces to some extent, allowing rapid growth along the [1] direction zone axis of the hexagonal phase, leading to the formation of ZnO hexagonal prisms with rod-like crystal. ZnO microflowers of hexagonal pyramid tips were obtained at a higher concentration of NaOH, where the pH of the solution was 13. It must be emphasised that higher concentrations of OH^−^ result in an increase in the solubility of Zn(OH)_2_ and ZnO at the room temperature, which is related to the formation of an ionic complex, above all Zn(OH)_4_^2−^. The solubility of Zn^2+^ complexes decreases in line with the increase in temperature during the microwave synthesis, which results in the precipitation of solid ZnO. The growth mechanism of ZnO microflowers with the petals of hexagonal pyramids may be explained as follows: when the pH value of the solution is high, a strong electrostatic interaction emerges between ions and polar surfaces, which leads to an increase in surface energies of polar surfaces (0001) and (1011) in comparison with the energy of the other surfaces of the hexagonal crystal. The growth rate of these polar surfaces decelerates and the polar surfaces appear as external surfaces with sharp edges. When the pH value is high, a strong electrostatic interaction emerges between ions and polar surfaces, which leads to an increase in surface energies of polar surfaces (0001) and (1011) in comparison with the energy of the other crystalline surfaces. The growth rate of these polar surfaces decelerates and the polar surfaces appear as external surfaces with sharp tips of the hexagonal prisms with pyramid tips.

Klofac et al. [574] report obtaining ZnO rods using a water suspension composed of Zn(CH_3_COO)_2_·2H_2_O, aqueous ammonia, polyethylene glycol (PEG; Mr = 400), and cetyltrimethylammonium bromide (CTAB, C_19_H_42_BrN) in their paper. The mechanism of the ZnO synthesis reaction proposed by Klofac et al. [574] was as follows in Equations (17)–(21):

(17) $${\mathrm{NH}}_{3}+H_{2}O\overset{H_{2}O\;}{↔}{\mathrm{NH}}_{4}{}^{+}+{\mathrm{OH}}^{-}$$

(18) $${\mathrm{Zn}}^{2+}+4{\mathrm{OH}}^{-}\overset{H_{2}O\;}{↔}{\left[\mathrm{Zn}{\left(\mathrm{OH}\right)}_{4}\right]}^{2-}$$

(19) $${\mathrm{Zn}}^{2+}+4{\mathrm{NH}}_{3}\overset{H_{2}O\;}{↔}{\left[\mathrm{Zn}{\left({\mathrm{NH}}_{3}\right)}_{4}\right]}^{2+}$$

(20) $${\left[\mathrm{Zn}{\left(\mathrm{OH}\right)}_{4}\right]}^{2-}\;\overset{H_{2}O\;}{\to }\mathrm{ZnO}↓+H_{2}O+2{\mathrm{OH}}^{-}$$

(21) $${\left[\mathrm{Zn}{\left({\mathrm{NH}}_{3}\right)}_{4}\right]}^{2+}+2{\mathrm{OH}}^{-}\overset{MH\;}{\to }\mathrm{ZnO}↓+4{\mathrm{NH}}_{3}+H_{2}O$$

Klofac et al. [574] obtained star- or flower-like ZnO particles aggregated from rod-like components united at a common centre depending on the surfactant used.

Based on these papers, a general conclusion may be drawn that depending on pH and NH_3_ derivatives the key role in reactions is played by stages related to the formation of complex compounds of zinc ions (Zn^2+^).

Details of other research papers concerning the microwave hydrothermal synthesis of ZnO without any additional heat treatment are presented in Table 5.

### 3.5. Microwave Hydrothermal Synthesis of ZnO Nanostructures with Additional Heat Treatment

The additional heat treatment process at the temperature from 150 °C to 900 °C (Table 6) of the microwave hydrothermal synthesis products is applied in order to remove organic impurities and to cause thermal decomposition of unreacted substrates and intermediates, e.g., Zn(OH)_2_, Zn_5_(OH)_8_(NO_3_)_2_·2H_2_O, Zn_5_(OH)_8_Cl_2_·H_2_O. The additional heat treatment process is applied mainly when reaction products are obtained in an open vessel in a microwave oven, which results in reaching a low reaction temperature (max. ca. 100 °C) (Table 6). The main disadvantage of the additional heat treatment in terms of structure of the obtained ZnO NMs is the sintering of particles and the increase in their size (recrystallisation process), which results in formation of large agglomerates/aggregates/conglomerates. Thus obtained ZnO NMs, despite the use of efficient homogenisation methods (e.g., ultrasounds), are characterised by a large average particle/agglomerate/aggregate size and high polydispersity in water suspensions.

**Figure 22:**
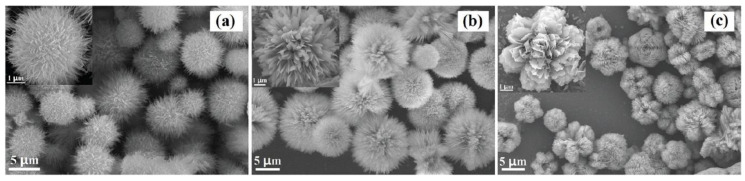
SEM images of the precursors of ZnO nanostructures synthesised with different dosages of urea: (**a**) 0.3 g, (**b**) 3.0 g, (**c**) 6.0 g. Reprinted from [654], Copyright (2014), with permission from Elsevier [OR APPLICABLE SOCIETY COPYRIGHT OWNER]. All rights reserved. In order to re-use permission must be obtained from the rightsholder.

**Figure 23:**
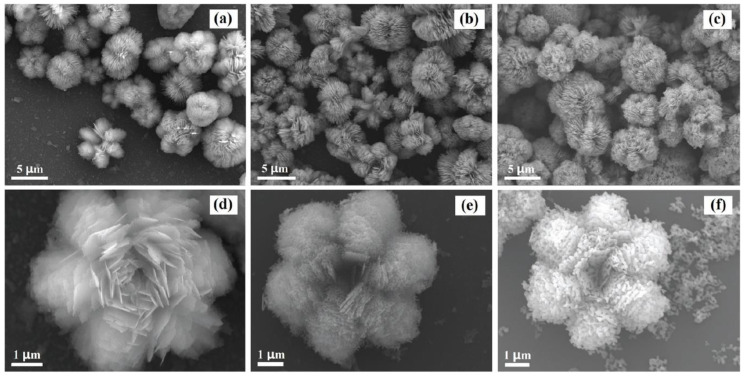
SEM images of the flower-like ZnO soaked at different temperatures: 400 °C (**a**,**d**), 500 °C (**b**,**e**), 600 °C (**c**,**f**). Reprinted from [654], Copyright (2014), with permission from Elsevier [OR APPLICABLE SOCIETY COPYRIGHT OWNER]. All rights reserved. In order to re-use permission must be obtained from the rightsholder.

When discussing aspects of application of ZnO NMs in a dry form, the additional heat treatment of samples improves the stability of nanostructures and gives unique properties by soaking ZnO in various gaseous atmospheres, which may result in the formation of oxygen vacancies in their structure. The controlled introduction of oxygen vacancies into ZnO NMs can manipulate their optical, electronic and surface properties [746]. An example that confirms the advantages of heat treatment is the paper by Gu et al. [654], where the authors used the obtained ZnO NMs for detecting gases. They used the aqueous solution of Zn(NO_3_)_2_ with varied urea contents for the synthesis. At the first stage, precursors were heated by microwaves for 40 min at the temperature of 90 °C, while at the second stage, the samples, after rinsing and drying, were soaked in various gaseous atmospheres (O_2_, N_2_, H_2_ and air) for 3 h at the temperature of 400, 500, and 600 °C. Gu et al. [654] obtained ZnO in the form of 3D nanostructures. Figure 22 shows the impact of the change in the urea content on the shape of the obtained 3D structures. With the lowest urea content (0.3 g) urchin-like structures were obtained (Figure 22a), while as a result of adding a higher quantity of urea (3.0 g and 6.0 g) flower-like structures were obtained (Figure 22b,c). The change in the soaking temperature from 400 °C to 500 °C caused a change in the shape of the flower structure and a change in the morphology of the nanoflakes themselves (Figure 23a,b). Structures soaked at 600 °C were characterised by a ragged and porous structure of the nanoflakes (Figure 23c).

**Figure 24:**
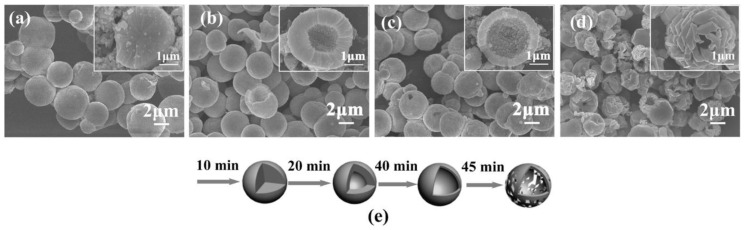
SEM images of morphology evolution of ZnO microspheres prepared with 2 mmol trisodium citrate dihydrate at 90 °C for 70 different microwave irradiation durations: (**a**) 10 min, (**b**) 20 min, (**c**) 40 min, and (**d**) 45 min. (**e**) Schematic illustration of the formation process of a hollow ZnO microsphere. Republished with permission of ©Royal Society of Chemistry from [652], Copyright (2013), permission conveyed through Copyright Clearance Center, INC. All rights reserved. In order to re-use permission must be obtained from the rightsholder.

**Figure 25:**
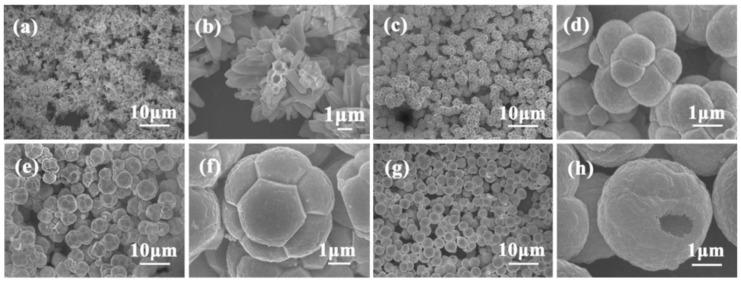
Products obtained with different amounts of trisodium citrate dihydrate at 90 °C for 40 min: (**a**–**b**) 0 mmol, (**c**–**d**) 0.08 mmol, (**e**–**f**) 0.2 mmol, and (**g**–**h**) 2 mmol. Republished with permission of ©Royal Society of Chemistry from [652], Copyright (2013), permission conveyed through Copyright Clearance Center, INC. All rights reserved. In order to re-use permission must be obtained from the rightsholder.

The paper by Li et al. [652] is another example of application of 3D structures of ZnO obtained by the microwave hydrothermal synthesis with an additional soaking process in applications related to gas detection. For the synthesis of ZnO hollow microspheres, Li et al. [652] used a solution created by mixing zinc acetate dihydrate (Zn(CH_3_COO)_2_·2H_2_O), trisodium citrate dihydrate (Na_3_C_6_H_5_O_7_·2H_2_O) with aqueous ammonia (NH_3_·H_2_O). At the first stage, precursors were heated by microwaves for 40 min at the temperature of 90 °C, while at the second stage the samples, after rinsing and drying, were soaked in an air environment for 4 h at the temperature of 400 °C. The mechanism of the ZnO synthesis reaction proposed by Li et al. [652] was identical with the mechanism proposed by Klofac et al. [574] per Equations (12)–(16). Figure 24 reveals how the authors controlled the morphology of the change to the microsphere filling by changing the synthesis duration. Figure 25 presents changes to the morphology caused by the change in the amounts of trisodium citrate dihydrate in the precursor solution.

**Figure 26:**
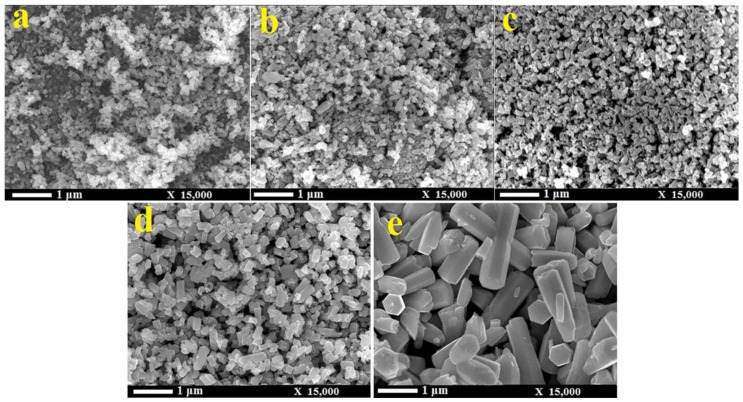
SEM images at the same magnifications of ZnO nanostructures produced with different molar ratios of Zn(NO_3_)_2_·6H_2_O and C_6_H_12_N_4_: (**a**) 3:20, (**b**) 5:20, (**c**) 12:20, (**d**) 20:20 and (**e**) 30:20. Reprinted from [660], Copyright (2019), with permission from Elsevier [OR APPLICABLE SOCIETY COPYRIGHT OWNER]. All rights reserved. In order to re-use permission must be obtained from the rightsholder.

Salah et al. [660] report developing a method of controlling the ZnO nanostructure size. They used an aqueous mixture of zinc nitrate dehydrate (Zn(NO_3_)_2_·6H_2_O) with hexamethylenetetramine (C_6_H_12_N_4_) as the reaction precursor. The first stage of microwave heating at the temperature of 120 °C lasted 10 min, and subsequently after rinsing and drying the synthesis products were soaked at 400 °C for 1 h. By applying various proportions of zinc nitrate dehydrate and hexamethylenetetramine in the precursor mixture, the authors [660] controlled the size of the structures within a range from several dozen nm to several µm (Figure 26). The change in the size of the ZnO structures was accompanied by the change in their morphology from spherical nanoparticles (Figure 26a) in micro sized hexagonal nanorods.

Details of other research papers concerning the microwave hydrothermal synthesis of ZnO with additional heat treatment are presented in Table 6.

### 3.6. Types of ZnO Nanocomposites or ZnO Hybrid Nanostructures Obtained by the Microwave Hydrothermal Synthesis

The microwave hydrothermal synthesis enables obtaining ZnO doped with the following ions: Cd^2+^ [698], Ce^4+^ [686,687,693], Co^2+^ [688,689,690,691,728,729], Cu^2+^ [695], Cr^3+^ [727,728], Eu^3+^ [694,731], Fe^2+^ [678,679,680,681,682,683,684,685], Ga^3+^ [696,697], K^+^ [692], Mn^2+^ [674,675], Sn^4+^ [733], and Sr^2+^ [699].

The microwave hydrothermal synthesis enables obtaining the following composite and hybrid materials: ZnO/ZnMn_2_O [676,677], ZnO/ZnFe_2_O_4_ [680,681,682,683,684,685], ZnO/ZnS [700], Au-decorated ZnO [701,702,738], Ag-decorated ZnO [638,703,704,705,706,735], reduced graphene oxide [707,708,709,710,711,712,713], ZrO_2_ coated ZnO [717], Fe_3_O_4_/ZnO/AgBr [719], MoS_2_/ZnO [720], ZnO doped CeO_2_ [721], mesoporous Si@ZnO [722], ZnO/zinc aluminium hydroxide [723], ZnO/TiO_2_ [724,725], ZnO/CuO [726], CdO-ZnO [730], and In_2_O_3_-ZnO [732].

### 3.7. ZnO Nanocomposites or ZnO Hybrid Nanostructures Obtained by the Microwave Hydrothermal Synthesis without Any Additional Heat Treatment

**Figure 27:**
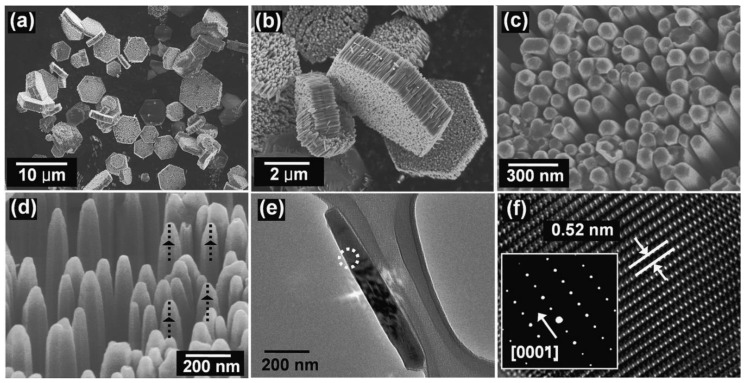
SEM images of hexagonal ZnO nanorod/zinc aluminium layered double hydroxide heterostructures: (**a**) low magnification; (**b**) medium magnification; (**c**) high magnification; (**d**) high-magnification oblique view image of ZnO nanorods grown on a zinc aluminium layered double hydroxide; (**e**) TEM image of a ZnO nanorod detached from a heterostructure; (**f**) High-resolution transmission electron microscopy (HRTEM) image of the point marked by the centre of the circle in (**e**). The inset is the SAED pattern. Republished with permission of ©The Royal Society of Chemistry, from [723], Copyright (2009), permission conveyed through Copyright Clearance Center, INC. All rights reserved. In order to re-use permission must be obtained from the rightsholder.

**Figure 28:**
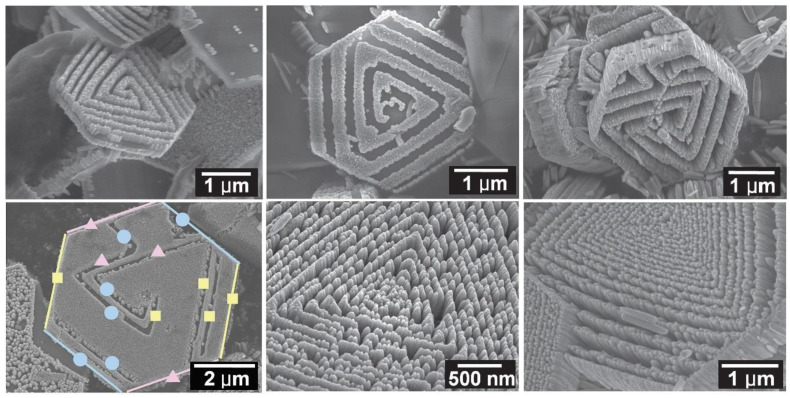
SEM images of the ZnO nanorod/ZnAl:LDH heterostructures with ZnO nanorod patterns on ZnAl:LDHs. Republished with permission of ©The Royal Society of Chemistry, from [723], Copyright (2009), permission conveyed through Copyright Clearance Center, INC. All rights reserved. In order to re-use permission must be obtained from the rightsholder.

**Figure 29:**
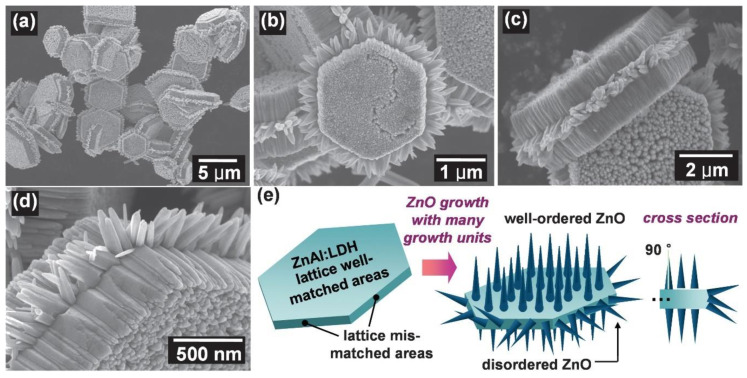
SEM images of sunflower-like ZnO nanorods/ZnAl:LDH heterostructures: (**a**) low magnification; (**b**,**c**) medium magnification; (**d**) high magnification; (**e**) schematic illustration of the formation of the sunflower-like ZnO nanorods/ZnAl:LDH heterostructures. Republished with permission of ©The Royal Society of Chemistry, from [723], Copyright (2009), permission conveyed through Copyright Clearance Center, INC. All rights reserved. In order to re-use permission must be obtained from the rightsholder.

**Figure 30:**
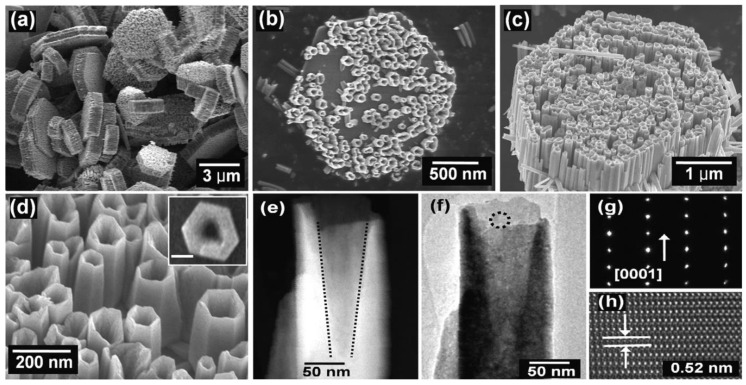
SEM images of hexagonal ZnO nanotubes/ZnAl:LDH heterostructures: (**a**) low magnification; (**b**) medium magnification (plan view); (**c**) medium magnification (oblique view); (**d**) high-magnification oblique view image of ZnO nanotubes grown on ZnAl:LDH; (**e**) HAADFSTEM image of a ZnO nanotube detached from a heterostructure; (**f**) TEM image of the ZnO nanotube; (**g**) SAED pattern; (**h**) HRTEM image of the point marked by the centre of the circle in (**f**). Republished with permission of ©The Royal Society of Chemistry, from [723], Copyright (2009), permission conveyed through Copyright Clearance Center, INC. All rights reserved. In order to re-use permission must be obtained from the rightsholder.

**Figure 31:**
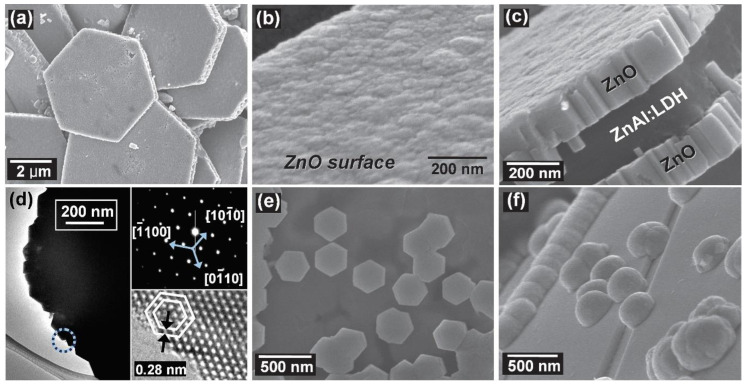
SEM images of hexagonal ZnO film/ZnAl:LDH heterostructures: (**a**) low magnification; (**b**) high-magnification image of the surface of the ZnO film; (**c**) high-magnification image of the side of the ZnO film/ZnAl:LDH heterostructures; (**d**) TEM image of a hexagonal ZnO film/ZnAl:LDH heterostructure. A corresponding electron diffraction (SAED) pattern and a HRTEM image of the point marked by a circle are inserted as the upper and lower insets, respectively; SEM images of the less dense ZnO nucleation areas of the heterostructures: (**e**) plan view; (**f**) oblique view. Republished with permission of ©The Royal Society of Chemistry, from [723], Copyright (2009), permission conveyed through Copyright Clearance Center, INC. All rights reserved. In order to re-use permission must be obtained from the rightsholder.

The potential of the microwave hydrothermal synthesis in obtaining ZnO hybrid nanostructures is proved by the results achieved by Cho et al. [723]. The paper by Cho et al. [723] is one of the more interesting publications reporting a synthesis of hierarchical hexagonal zinc oxide/zinc aluminium hydroxide heterostructures through epitaxial growth using microwave irradiation. For obtaining a substrate being zinc aluminium layered double hydroxide heterostructures (ZnAl:LDH), the authors used the solution of zinc acetate dihydrate (Zn(CH_3_COO)_2_·2H_2_O), aluminium chloride (AlCl_3_) and ammonia water (NH_3_·H_2_O). The microwave hydrothermal synthesis of ZnAl:LDH lasted 20 min at the temperature of 95 °C. The product, ZnAl:LDH substrate, was thoroughly rinsed and dried after the synthesis. Depending on the selected synthesis parameters and modifications of the precursor composition, the authors obtained various structures that grew on the surface of the ZnAl:LDH substrate, namely: hexagonal ZnO nanorods (Figure 27 and Figure 28), sunflower-like ZnO nanorods (Figure 29), ZnO nanotubes (Figure 30) and ZnO film (Figure 31). The process of ZnO nanorod growth on the substrate surface was as follows: the substrate (ZnAl:LDH) was introduced to the solution of the mixture of Zn(CH_3_COO)_2_·2H_2_O and NH_4_OH and was subjected to microwave heating (20 min, 95 °C), and subsequently the product was rinsed with deionised water and dried. On the surface of some of the substrates, the growing ZnO nanorods formed repeatable patterns, which can be seen in Figure 28. The formation mechanism of these regular patterns was not tested by the authors [723], but the occurring regular patterns indicate that the nucleation of ZnO on the surface of the ZnAl:LDH strongly depends on the atomic arrangements of the ZnAl:LDH surface. The process of sunflower-like ZnO nanorod growth on the substrate surface (Figure 29) was virtually identical to the growth of ZnO nanorods apart from the applied Zn(CH_3_COO)_2_·2H_2_O concentration, which was increased twice relative to the original solution used for the growth of ZnO nanorods. The growth of ZnO nanotubes (Figure 30) on the ZnAl:LDH surface, in turn, consisted in introducing the substrate to the solution of the mixture of Zn(CH_3_COO)_2_·2H_2_O with NH_4_OH and subjected to microwave heating (20 min, 95 °C), and subsequently the suspension was aged at the room temperature for 10 h, after which the product was rinsed with deionised water and dried. For the synthesis of a 2D ZnO film on the surface of ZnAl:LDH heterostructures, the composition of the precursor solution was modified by adding tripotassium citrate monohydrate (HOC(COOK)(CH_2_COOK)_2_·H_2_O). As a result of microwave heating, sandwich-like heterostructures were obtained (Figure 31). In our opinion, the results of that paper indicate that the microwaves could have potentially acted as a stimulator of a stereochemical reaction (i.e., spatial orientation of the structure). The reaction temperature was not high, merely 95 °C, where an identical temperature could have been reached by pre-soaking of substrates on a heating plate, but the clear spatial organisation of the structure (Figure 27, Figure 28, Figure 29, Figure 30 and Figure 31) suggests the participation of additional mechanisms, e.g., an electromagnetic field.

Details of other research papers concerning the microwave hydrothermal synthesis of ZnO with additional heat treatment are presented in Table 7.

### 3.8. ZnO Nanocomposites or ZnO Hybrid Nanostructures Obtained by the Microwave Hydrothermal Synthesis with Additional Heat Treatment

**Figure 32:**
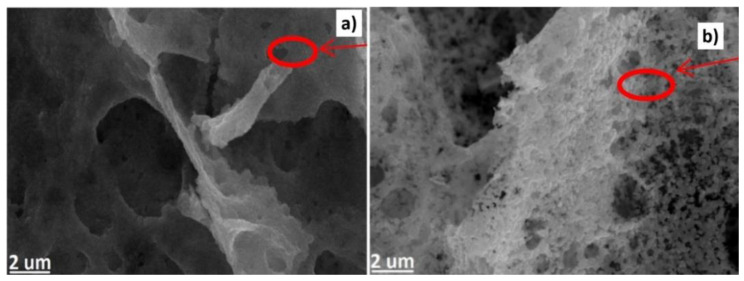
SEM images of doped ZnO: (**a**) 15% Co doped ZnO, (**b**) 15% Cr doped ZnO. Reprinted from [728], Copyright (2017), with permission from Elsevier [OR APPLICABLE SOCIETY COPYRIGHT OWNER]. All rights reserved. In order to re-use permission must be obtained from the rightsholder.

As already mentioned, the additional soaking process of the microwave hydrothermal synthesis product aims, above all, to complete the conversion of the substrates or intermediates. In the case of a synthesis of doped ZnO, additional heat treatment mostly leads to the precipitation of foreign phases, e.g., Cr_2_O_3_ [727,728], Eu_2_O_3_ [731], and SnO_2_ [733]. Depending on the type of gaseous atmosphere, dopants may also undergo a partial oxidation or reduction process. Synthesis products subjected to an additional soaking process may be also characterised by a compact structure, where the particles are sintered or strongly agglomerated, which is indicated, e.g., by the results of the research by Bhattia et al. [728] in Figure 32.

**Figure 33:**
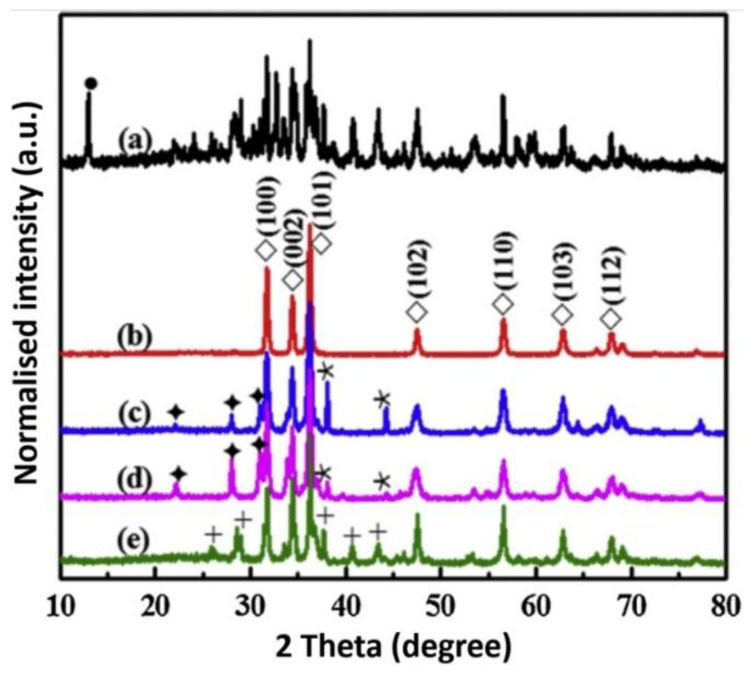
Normalised XRD patterns of Ag/Ag_2_SO_4_/ZnO nanostructures: (**a**) as-synthesised for molar ratio of thiourea and Ag^+^ kept at 1:1 before calcination, (**b**) pure ZnO, (**c**) synthesised for molar ratio of thiourea and Ag^+^ kept at 1:2, (**d**) synthesised for molar ratio of thiourea and Ag^+^ kept at 1:1 and (**e**) catalysts after calcination synthesised for molar ratio of thiourea and Ag^+^ kept at 2:1 (◊—ZnO, ♦—Ag_2_SO_4_, *—Ag, +—Ag_2_S, ●—Zn_5_(CO_3_)_2_(OH)_6_). Reprinted from [736], Copyright (2015), with permission from Elsevier [OR APPLICABLE SOCIETY COPYRIGHT OWNER]. In order to re-use permission must be obtained from the rightsholder.

One of the purposes of applying an additional process of synthesis product soaking is to give unique features both to the doped ZnO materials and composite ZnO materials, which results in the possibility to apply them e.g., as photocatalysts [724,732,734], gas sensors [725], dilute magnetic semiconductors (DMS) for spintronics applications [728]. A good example of obtaining a hybrid material with the use of an additional soaking process is the paper by Cao et al. [736], which describes obtaining Ag/Ag_2_SO_4_/ZnO nanostructures. The authors [736] used the aqueous mixture of Zn(CH_3_COO)_2_·2H_2_O, CO(NH_2_), CS(NH_2_)_2_ and AgNO_3_ as the precursor. The composition of the mixture was modified by changing the molar ratio of thiourea and Ag^+^, which was kept at 1:2, 1:1, 2:1, respectively. The samples were heated by microwaves for 30 min at the temperature of 170 °C, subsequently rinsed (water, ethanol) and were subjected to calcination for 4 h at the temperature of 500 °C in the air atmosphere. Figure 33 contains XRD results of an example as-synthesised sample (only after the microwave synthesis) and the XRD results of samples after an additional soaking process.

The as-synthesised sample reveals a considerable quantity of foreign inclusions, above all Zn_5_(CO_3_)_2_(OH)_6_ product (intermediate, by-product). The authors [736] proposed a mechanism of the formation of (Zn_5_(CO_3_)_2_(OH)_6_) by-product and main products (ZnO, Ag_2_S), which is described by the following Equations (22)–(30):

(22) $$\mathrm{CO}{\left({\mathrm{NH}}_{3}\right)}_{2}+H_{2}O\overset{T\;>\;60\;{}^{\circ}C\;}{\to }2{\mathrm{NH}}_{3}{}^{+}+{\mathrm{CO}}_{2}$$

(23) $$\mathrm{CS}{\left({\mathrm{NH}}_{3}\right)}_{2}+2H_{2}O\overset{T\;>\;60\;{}^{\circ}C\;}{\to }2{\mathrm{NH}}_{3}{}^{+}+H_{2}S+{\mathrm{CO}}_{2}$$

(24) $${\mathrm{NH}}_{3}+H_{2}O\overset{H_{2}O\;}{↔}{\mathrm{NH}}_{4}{}^{+}+{\mathrm{OH}}^{-}$$

(25) $${\mathrm{CO}}_{2}+2{\mathrm{OH}}^{-}\overset{H_{2}O\;}{↔}{\mathrm{CO}}_{3}{}^{2-}+H_{2}O\;$$

(26) $$5{\mathrm{Zn}}^{2+}+2{\mathrm{CO}}_{3}{}^{2-}+6{\mathrm{OH}}^{-}\overset{\mathrm{MH}\;}{\to }{\mathrm{Zn}}_{5}{\left({\mathrm{CO}}_{3}\right)}_{2}{\left(\mathrm{OH}\right)}_{6}↓$$

(27) $${\mathrm{Zn}}^{2+}+2{\mathrm{OH}}^{-}\overset{}{\to }\mathrm{Zn}{\left(\mathrm{OH}\right)}_{2}↓$$

(28) $$\mathrm{Zn}{\left(\mathrm{OH}\right)}_{2}\overset{\;\mathrm{MH}}{\to }\mathrm{ZnO}↓+H_{2}O$$

(29) $${\mathrm{Zn}}_{5}{\left({\mathrm{CO}}_{3}\right)}_{2}{\left(\mathrm{OH}\right)}_{6}\;↓\overset{\mathrm{MH}\;}{\to }5\mathrm{ZnO}↓+2{\mathrm{CO}}_{2}+3H_{2}O$$

(30) $$H_{2}S+2{\mathrm{Ag}}^{+}\overset{\;\mathrm{MH}}{\to }{\mathrm{Ag}}_{2}S↓+2H^{+}$$

**Figure 34:**
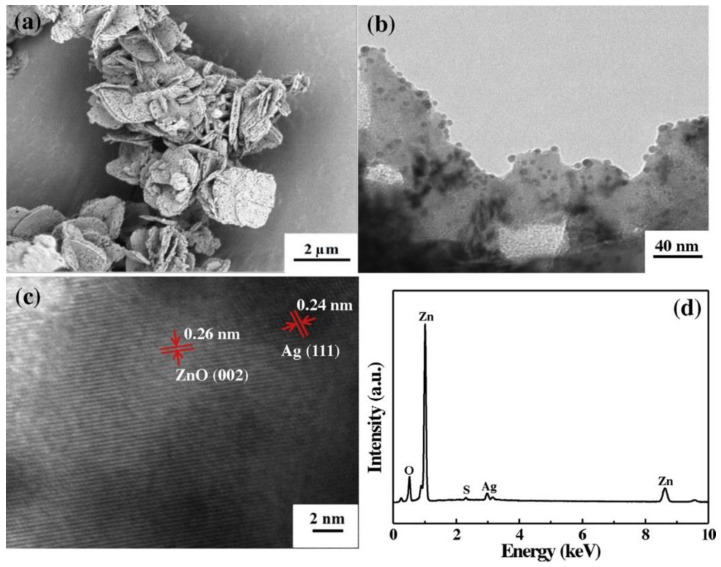
Figures should be placed in the main text near to the first time they are cited. A caption on a single line should be centered. (**a**) SEM image, (**b**) TEM image, (**c**) TEM image and (**d**) Energy dispersive spectroscopy (EDS) spectrum of Ag/Ag_2_SO_4_/ZnO nanostructures synthesised for ratio of thiourea and Ag^+^ of 1:1. Reprinted from [736], Copyright (2015), with permission from Elsevier [OR APPLICABLE SOCIETY COPYRIGHT OWNER]. All rights reserved. In order to re-use permission must be obtained from the rightsholder.

It must be underlined that ZnO, Ag_2_S and Zn_5_(CO_3_)_2_(OH)_6_ compounds were present in samples obtained only as a result of the microwave synthesis. However, after the calcination (Figure 34), the presence of such compounds as Ag_2_SO_4_ and metallic Ag was observed in the samples, which was related to the soaking of samples in an oxidising atmosphere (air). During the soaking, Ag_2_S and Zn_5_(CO_3_)_2_(OH)_6_ decomposed. In the sample with the highest addition of thiourea also ZnS formed. The course of the oxidation process was explained by the following Equations (31)–(32):

(31) $${\mathrm{Ag}}_{2}S+O_{2}\overset{500\;{}^{\circ}C,4\;h,\;\mathrm{air}\;}{\to }2\mathrm{Ag}↓+{\mathrm{SO}}_{2}$$

(32) $${\mathrm{Ag}}_{2}S+2O_{2}\overset{500\;{}^{\circ}C,4\;h,\;\mathrm{air}\;}{\to }2{\mathrm{AgSO}}_{4}↓$$

An example morphology of the Ag/Ag_2_SO_4_/ZnO composite sample is shown in Figure 34. The obtained plates structure was composed of multiple nanoparticles and had rough and irregularly porous surfaces. Obtaining a plates structure was explained by Cao et al. [736] as follows: due to the hydrolysis reactions of urea and thiourea, Ag_2_S/Zn_5_(CO_3_)_2_(OH)_6_/ZnO nanoparticles are obtained at the first stage of microwave heating. At the second stage, the existing nanoparticles rapidly formed into plates aggregates for the purpose of decreasing their surface energy. The plates shown in Figure 34 had the diameter of ca. 1.5–2 µm and the thickness of ca. 100–200 nm. The TEM image in Figure 34b indicates monodispersity of Ag/Ag_2_SO_4_ NPs with the diameters of ca. 4–7 nm, which were uniformly anchored on the surface of ZnO plates. Figure 34c presents two different kinds of the lattice fringes with 0.24 nm and 0.26 nm, corresponding to the distance of Ag (1 1 1) and ZnO (0 0 2) planes, respectively. Figure 34d confirms the qualitative composition of the Ag_2_S/Ag_2_SO_4_/ZnO composite obtained by Cao et al. [736].

Details of other research papers concerning the microwave synthesis of ZnO nanocomposites or ZnO hybrid nanostructures with additional heat treatment are presented in Table 8.

## 4. Microwave Solvothermal Synthesis of ZnO

According to the literature [747], a solvothermal synthesis is a process in a closed system, in which chemical reactions take place exclusively in non-aqueous solvents at increased temperatures, at a pressure that is higher than atmospheric pressure (*P* > 101,325 Pa). The literature [748,749] provides a different definition of the solvothermal synthesis, e.g., it is a chemical reaction based on non-aqueous solvents or their mixtures. The application of water mixtures and organic solvents in syntheses of metal oxides made the definitions of the hydrothermal synthesis and the solvothermal synthesis often used interchangeably. However, this is not reasonable and there is no need to use these definitions interchangeably. The main reason for developing the solvothermal synthesis was attempts to obtain non-oxide materials (in particular metal particles) [747,748,749,750,751,752]. In syntheses with the use of organic solvents, the critical parameter is the temperature of the onset of initial decomposition, because after exceeding this temperature solvents begin undergoing the thermal decomposition, polymerisation or oxidation process. Solvothermal synthesis is much more expensive than hydrothermal synthesis, which results from the costs of application of organic solvents, their disposal and the need to adapt the laboratory (ventilation, laboratory fume hood, etc.). However, the costs related to the maintenance and safe use of reactors operating in milder conditions of solvothermal synthesis are considerably lower than in the case of high temperatures and pressures dedicated to hydrothermal syntheses. As a result, the solvothermal technology is becoming cheaper, safer, and more popular year by year. 

For the purposes of this review, we assume that the “solvothermal synthesis” is a process occurring in an inorganic solvent environment (where m_s_ > 50%), with the pressure equal to or higher than atmospheric pressure. The results of the literature review concerning microwave solvothermal synthesis of ZnO are divided into the following subgroups:

(1) Microwave solvothermal synthesis of ZnO nanostructures without any additional heat treatment, where the literature review results [402,573,758,759,760,761,762,763,764,765,766,767,768,769,770,771,772,773,774,775,776,777,778,779,780,781,782,783,784,785,786,787,788,789,790,791,792,793,794,795,796,797] are summarised in Table 10.
(2) Microwave solvothermal synthesis of ZnO nanocomposites or ZnO hybrid nanostructures without any additional heat treatment, where the literature review results [798,799,800,801,802,803,804,805,806,807,808,809,810,811,812,813,814,815,816,817,818,819,820,821,822] are summarised in Table 11.
(3) Microwave solvothermal synthesis of undoped ZnO, ZnO nanocomposites or ZnO hybrid nanostructures with additional heat treatment, where the literature review results [823,824,825,826,827,828,829,830,831,832,833,834,835,836,837,838] are summarised in Table 12.

### 4.1. Reactants

**Figure 35:**
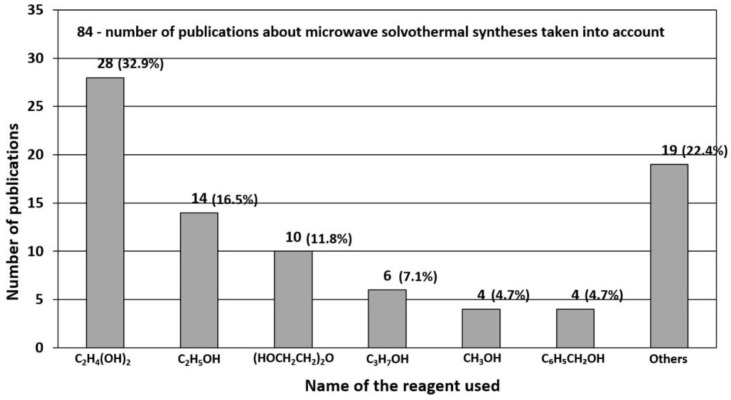
Statistics of use of various organic solvents in the microwave solvothermal synthesis. Source: [402,573,758,759,760,761,762,763,764,765,766,767,768,769,770,771,772,773,774,775,776,777,778,779,780,781,782,783,784,785,786,787,788,789,790,791,792,793,794,795,796,797,798,799,800,801,802,803,804,805,806,807,808,809,810,811,812,813,814,815,816,817,818,819,820,821,822,823,824,825,826,827,828,829,830,831,832,833,834,835,836,837,838].

The most popular organic solvents used for the microwave solvothermal synthesis of ZnO according to the data derived from the literature review [758,759,760,761,762,763,764,765,766,767,768,769,770,771,772,773,774,775,776,777,778,779,780,781,782,783,784,785,786,787,788,789,790,791,792,793,794,795,796,797,798,799,800,801,802,803,804,805,806,807,808,809,810,811,812,813,814,815,816,817,818,819,820,821,822,823,824,825,826,827,828,829,830,831,832,833,834,835,836,837,838] (Tables 10–12) are ethylene glycol (EG, C_2_H_4_(OH)_2_), ethanol (C_2_H_5_OH), diethylene glycol (DEG, (HOCH_2_CH_2_)_2_O), propanol (C_3_H_7_OH), methanol (CH_3_OH), benzyl alcohol (C₆H₅CH₂OH) and other solvents (Figure 35). The following were used sporadically as a solvent in the microwave solvothermal synthesis: acetone (C_3_H_6_O) [778], acetonitrile (C_2_H_3_N, [789]), alkoxyethanol [772], butanediol (C_4_H_8_(OH)_2_) [769], butoxyethanol (CH_3_C_3_H_6_OC_2_H_4_OH) [772], ethoxyethanol (C_2_H_5_OC_2_H_4_OH) [772], glycerol ((CH_2_OH)_2_CHOH) [822], hexanol (C_6_H_13_OH) [766], methoxyethanol (CH_3_OC_2_H_4_OH) [772], N,N-dimethylacetamide (CH_3_CON(CH_3_)_2_) [792], mixture of oleic acid (C_8_H_17_CH=CH(CH_2_)_7_COOH) with oleylamine [807], propanediol (C_3_H_6_(OH)_2_) [776], tetraethylene glycol (TTEG, C_8_H_18_O_5_) [402], triethylene glycol (TEG, C_6_H_14_O_4_) [402], tetrahydrofuran (C_4_H_8_O, THF) [795], and polyethylene glycol (PEG400) [768].

The popularity of solvents in microwave solvothermal syntheses is affected above all by such factors as availability, price, toxicity and boiling point (Table 9). Ethylene glycol and diethylene glycol are relatively cheap solvents, which are produced on an industrial scale and can be applied above all as components of coolants and as components for production of polyester resins. Ethanol and methanol are two most popular alcohols, which are applied on a global scale as primary solvents in organic syntheses. The application of organic solvents enables a considerable decrease in the pressure, temperature, and duration of the process in relation to a synthesis of the same product in water alone [839]. For example, the reaction of ZnO synthesis at the temperature of 230 °C in water is characterised by the equilibrium pressure of ca. ~28 bar [839], while in ethylene glycol for the same temperature the pressure is merely ~4 bar [502]. In a chemical reaction in the liquid state, only temperature is important, while pressure is only a derivative of the process conditions.

**Figure 36:**
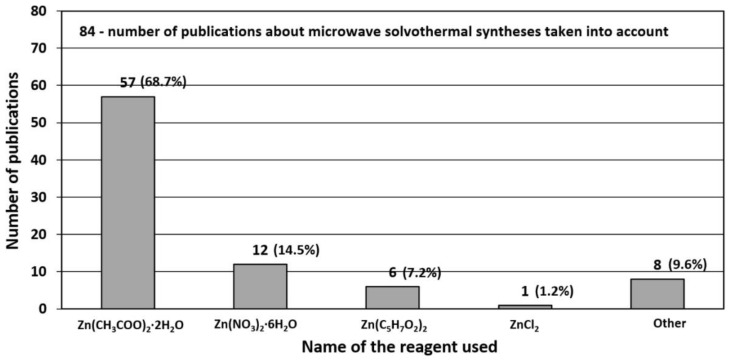
Statistics of use of reactants (Zn^2+^ salts) in the microwave solvothermal synthesis. Source: [402,573,758,759,760,761,762,763,764,765,766,767,768,769,770,771,772,773,774,775,776,777,778,779,780,781,782,783,784,785,786,787,788,789,790,791,792,793,794,795,796,797,798,799,800,801,802,803,804,805,806,807,808,809,810,811,812,813,814,815,816,817,818,819,820,821,822,823,824,825,826,827,828,829,830,831,832,833,834,835,836,837,838].

The most popular reactants of the “Zn^2+^” zinc cation precursor used for ZnO synthesis according to the data derived from the literature review [758,759,760,761,762,763,764,765,766,767,768,769,770,771,772,773,774,775,776,777,778,779,780,781,782,783,784,785,786,787,788,789,790,791,792,793,794,795,796,797,798,799,800,801,802,803,804,805,806,807,808,809,810,811,812,813,814,815,816,817,818,819,820,821,822,823,824,825,826,827,828,829,830,831,832,833,834,835,836,837,838] (Table 10, Table 11 and Table 12) are zinc acetate (Zn(CH_3_COO)_2_·*x*H_2_O), zinc nitrate (Zn(NO_3_)_2_·6H_2_O), zinc acetylacetonate (Zn(C_5_H_7_O_2_)_2_), zinc chloride (ZnCl_2_), and other (Figure 36). The following were used sporadically as the “Zn^2+^” zinc cation precursor in the microwave solvothermal synthesis: ZnO [775,816,819,822], bis(acetylacetonato)zinc [789], bis(methylacetato)zinc [789], bis(dimethylmalonato)zinc [789]. 

The popularity of zinc acetate (68.7%) results mainly from its availability and solubility in most organic solvents. 

**Figure 37:**
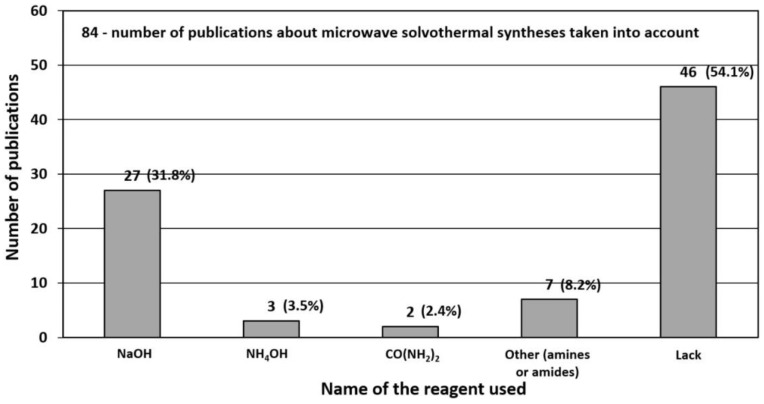
Statistics of use of reactants (OH^−^) in the microwave solvothermal synthesis. Source: [402,573,758,759,760,761,762,763,764,765,766,767,768,769,770,771,772,773,774,775,776,777,778,779,780,781,782,783,784,785,786,787,788,789,790,791,792,793,794,795,796,797,798,799,800,801,802,803,804,805,806,807,808,809,810,811,812,813,814,815,816,817,818,819,820,821,822,823,824,825,826,827,828,829,830,831,832,833,834,835,836,837,838].

The most popular reactants being hydroxide anion precursor chemicals “OH^−^” used for the ZnO synthesis according to the data collected from the literature review [758,759,760,761,762,763,764,765,766,767,768,769,770,771,772,773,774,775,776,777,778,779,780,781,782,783,784,785,786,787,788,789,790,791,792,793,794,795,796,797,798,799,800,801,802,803,804,805,806,807,808,809,810,811,812,813,814,815,816,817,818,819,820,821,822,823,824,825,826,827,828,829,830,831,832,833,834,835,836,837,838] (Table 10, Table 11 and Table 12) (Figure 37) are sodium hydroxide (NaOH), ammonia water (NH_3_·H_2_O, NH_4_(OH)), as well as amides and amines, e.g., C_2_H_8_N_2_ [775], hexamethylene tetramine (C_6_H_12_N_4_) [786], triethanolamine (TEA, C_6_H_15_NO_3_) [786], diethanolamine ((CH_2_CH_2_OH)_2_NH) [791], thioacetamide (C_2_H_5_NS) [819]. In over a half of publications, no hydroxide anion precursor chemical “OH^−^” was used, because the “OH^−^” anion was formed during the synthesis as a result of a reaction of a Zn^2+^ salt with a solvent, forming basic salts [402].

### 4.2. Surfactants

As mentioned before, surfactants are commonly used in nanotechnology for passivation of surface, control of shape, and giving of photophysical properties [740]. The following surfactants were used in microwave solvothermal syntheses of ZnO NMs for the purpose of controlling the morphology:

- Polyvinyl alcohol (PVA) for obtaining nanoflakes [779].
- Polyethylene glycol 400 (PEG-400) for obtaining nanorods [779].
- Polyvinylpyrrolidone (PVP) for obtaining flowers with rod-like petals [779] and nanorods [825,827,828,829,830].
- Cetyltrimethylammonium-bromide (CTAB) for obtaining flowers with rod-like petals [779], nanorods [827,830] and chrysanthemum-like prismatic nanorods [838].
- Polyethylene glycol for obtaining NPs [232].
- Methylimidazole for obtaining uniform particles with approximately rhombic dodecahedron facets [832].
- Triton X100 for obtaining nanorods [827,830].

### 4.3. Morphology

Morphologies of ZnO reported in the literature are presented in the description of properties in Table 10, Table 11 and Table 12 [758,759,760,761,762,763,764,765,766,767,768,769,770,771,772,773,774,775,776,777,778,779,780,781,782,783,784,785,786,787,788,789,790,791,792,793,794,795,796,797,798,799,800,801,802,803,804,805,806,807,808,809,810,811,812,813,814,815,816,817,818,819,820,821,822,823,824,825,826,827,828,829,830,831,832,833,834,835,836,837,838]. ZnO nanomaterials and micromaterials synthesised with the use of the microwave solvothermal synthesis had the following shapes: butterfly-like [765], cables [819], core-shell structures [816], hexagonal prisms [765], hierarchical architectures constructed by nanoparticles [806], mulberry-like structures [786], needle-like [785], peanut-like structures [765], pyramids [807], rice-like particles [777,787], spheroidal nanostructures [784], sword-like wires [765], tubes [770], wires [783,792], sheet-like structures [785], spheres [765,791], flower-like structures [573,775,779,785,786,787,794,838], rod-like shape structures [402,705,764,767,770,771,778,780,785,788,797,805,813,822,825,826,827,828,829,830], and spherical-like shape structures [181,402,758,759,760,761,762,763,764,766,769,772,773,774,776,778,780,789,790,793,795,796,798,799,800,801,802,803,804,808,809,810,811,812,814,818,823,824,825,826,827,828,829,830,831,833,834,835,836,837,864].

**Figure 38:**
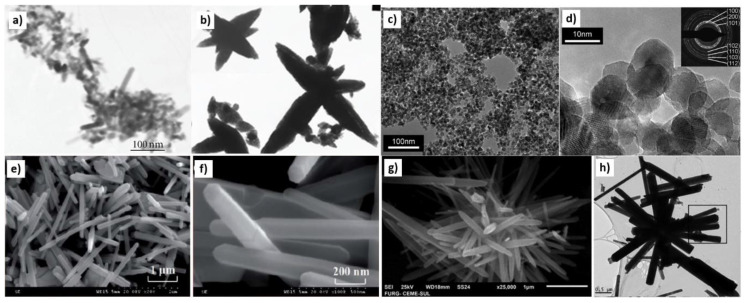
Examples of various ZnO structures obtained by the microwave solvothermal synthesis: (**a**) TEM image of ZnO nanorods (Reprinted from [778], Copyright ©2012 Khoza et al., Article licensed under a CC BY 3.0, https://creativecommons.org/licenses/by/3.0/); (**b**) TEM image of ZnO stars (Reprinted from [778], Copyright ©2012 Khoza et al., Article licensed under a CC BY 3.0, https://creativecommons.org/licenses/by/3.0/); (**c**,**d**) TEM images of ZnO nanoparticles (Reprinted with permission from [789], ©The Royal Society of Chemistry, Article licensed under a CC BY 3.0, https://creativecommons.org/licenses/by/3.0/); (**e**,**f**) SEM images of Ag/ZnO rods (Reprinted with permission from [838], Copyright ©2018 Xin et al., Article licensed under a CC BY-NC 3.0, https://creativecommons.org/licenses/by-nc/3.0/); (**g**,**h**) SEM and TEM images of nanorods agglomerated as urchin-like structures (Reprinted with permission from [797], Copyright ©2019 de Pereset et al., Article licensed under a CC BY 4.0, https://creativecommons.org/licenses/by/4.0/).

**Figure 39:**
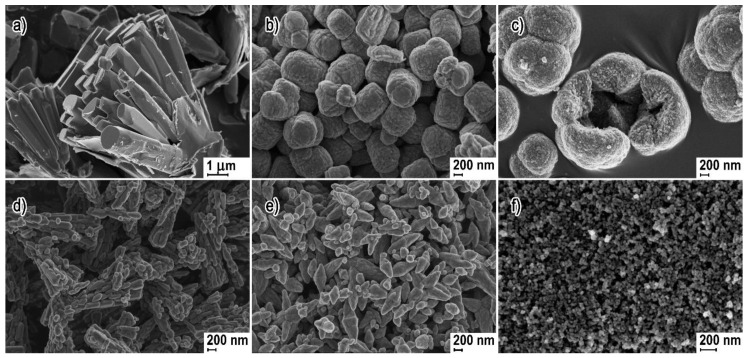
Examples of various ZnO structures obtained by the microwave solvothermal synthesis: (**a**) ZnO rods, (**b**) ZnO barrels, (**c**) ZnO flowers, (**d**), ZnO sticks, (**e**) ZnO irregular particles, (**f**) ZnO spherical NPs. Reprinted with permission from [496], Copyright ©2018 Dąbrowska et al., Article licensed under a CC BY 4.0, https://creativecommons.org/licenses/by/4.0/.

**Figure 40:**
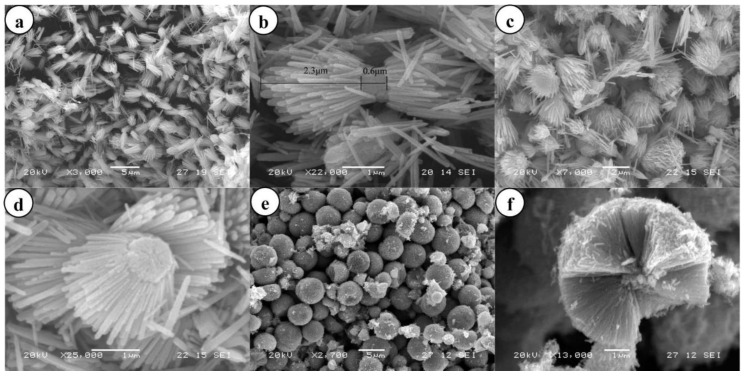
(**a**,**b**) Low- and high-magnification SEM images of the straw-bundle-like ZnO, (**c**,**d**) low-and high-magnification SEM images of the wide chrysanthemum-like ZnO, (**e**) low-magnification SEM image of the nanorod-based microspheres, (**f**) high-magnification SEM image of a typical broken microsphere. Reprinted (adapted) with permission from [761]. Copyright © 2008, American Chemical Society. All rights reserved. In order to re-use permission must be obtained from the rightsholder.

**Figure 41:**
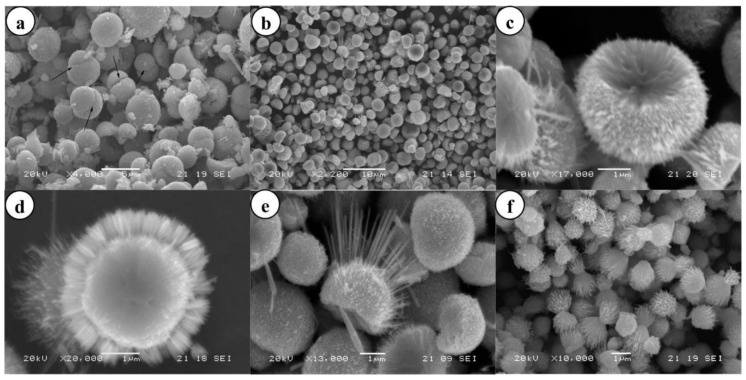
(**a**) SEM image of ZnO microspheres (irregular), (**b**) SEM image of nanorod-based microspheres, (**c**) SEM image of microspheres with a concave central part, (**d**,**e**) SEM images of microspheres with protruding nanorods, (**f**) SEM image of wide chrysanthemum-like nanostructures. Reprinted (adapted) with permission from [761]. Copyright © 2008, American Chemical Society. All rights reserved. In order to re-use permission must be obtained from the rightsholder.

**Figure 42:**
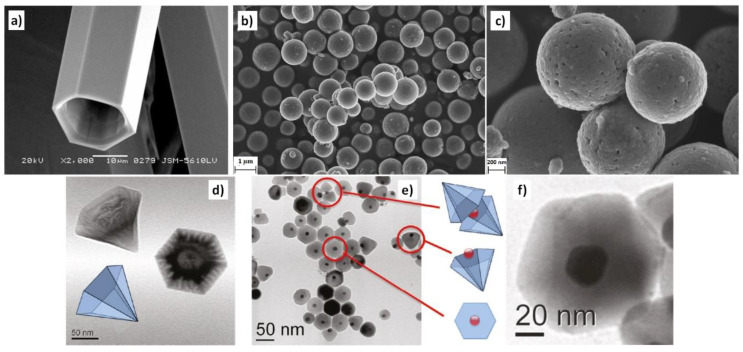
(**a**) SEM image of ZnO tubes (Reprinted from [868], Copyright (2005), with permission from Elsevier [OR APPLICABLE SOCIETY COPYRIGHT OWNER]); (**b**,**c**) SEM images of spherical Gd doped ZnO particles (own test results); (**d**–**f**) TEM images of Au-ZnO nanopyramids (Reprinted (adapted) with permission from [807]. Copyright © 2011, American Chemical Society). (**a**,**d**–**f**) All rights reserved. In order to re-use permission must be obtained from the rightsholder.

**Figure 43:**
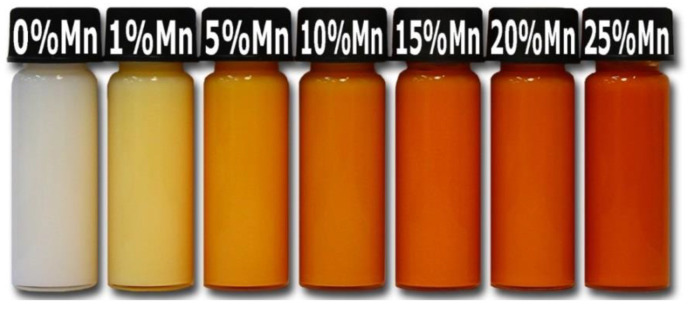
Visual comparison of changes in colours of suspensions of Zn_1−*x*_Mn*_x_*O NPs depending on the dopant content. Reprinted with permission from [802]. Copyright ©2016 Wojnarowicz et al. Article licensed under a CC BY 2.0, https://creativecommons.org/licenses/by/2.0/.

**Figure 44:**
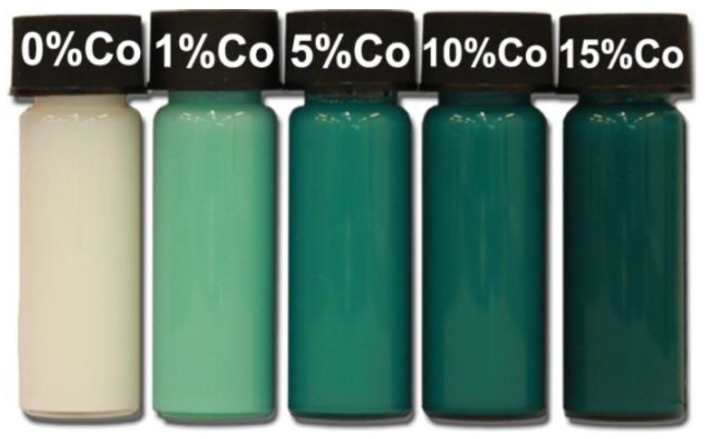
Visual comparison of changes in colours of suspensions of Zn_1−*x*_Co*_x_*O NPs depending on the dopant content. Reprinted with permission from [799], Copyright ©2015 Glass Ceram (Warsaw, Poland).

**Figure 45:**
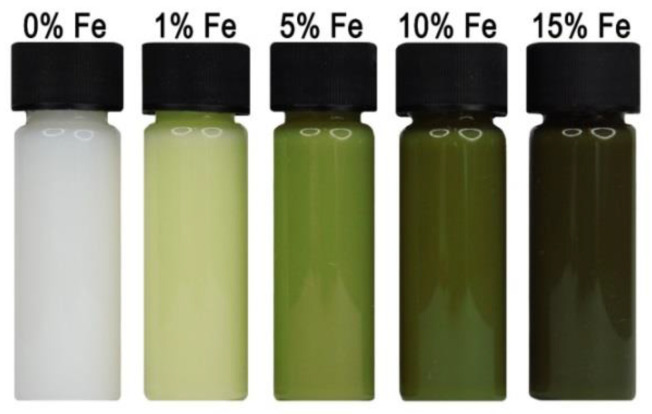
Visual comparison of changes in colours of suspensions of Zn_1−*x*_Fe*_x_*O NPs depending on the dopant content. Source: own test results.

**Figure 46:**
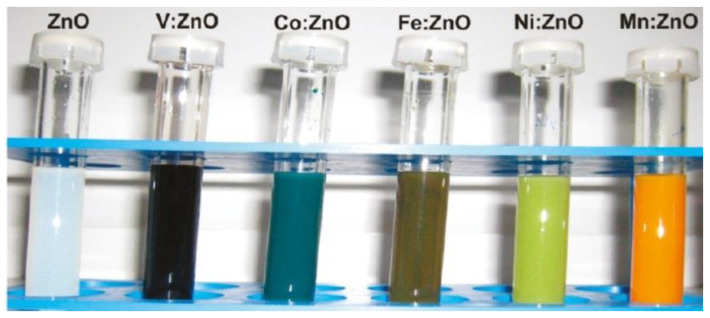
Photographs of ZnO NPs dispersions in benzyl alcohol with different colours depending on the composition. Reprinted (adapted) with permission from [810]. Copyright © 2011, American Chemical Society. All rights reserved. In order to re-use permission must be obtained from the rightsholder.

**Figure 47:**
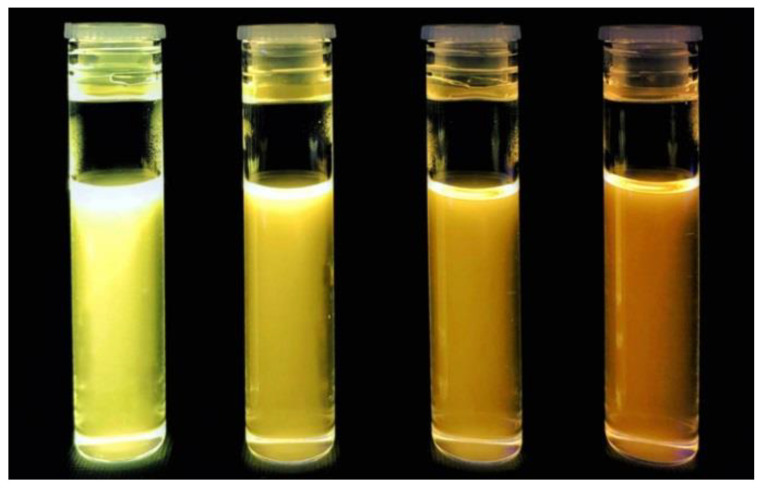
Photograph of oleate-stabilised ZnO NPs, dispersed in cyclohexane, under UV light (λ = 365 nm) in order of increasing synthesis temperature of 125 °C (2.6 nm), 150 °C (2.7 nm), 175 °C (3.1 nm), and 200 °C (3.8 nm) (left to right). Reprinted (adapted) with permission from [795]. Copyright © 2019, American Chemical Society. All rights reserved. In order to re-use permission must be obtained from the rightsholder.

Examples of various morphologies of ZnO obtained by the microwave solvothermal method are presented in Figure 38, Figure 39, Figure 40, Figure 41 and Figure 42. Changes in the colours of suspensions of doped ZnO NMs caused by the type and quantity of dopant are reflected in Figure 43, Figure 44, Figure 45 and Figure 46, while Figure 47 shows a change in the colour of ZnO NPs colloid caused by the use of various sizes of NPs.

### 4.4. Microwave Solvothermal Synthesis of ZnO without Any Additional Heat Treatment

One of the main advantages of the solvothermal technology is the possibility to use mixtures of precursors in the form of solutions rather than sedimenting suspensions of hydroxides, as is the case in the majority of hydrothermal syntheses of ZnO in water. Thanks to that, numerous problems are avoided, above all with heterogeneity and unrepeatability of the precursor (suspension sedimentation) and the lack of stirring of the precursor suspension in the reactor, which critically affects the quality and homogeneity of the obtained products. The application of non-aqueous solutions or those with a limited water content eliminates the presence of competitive mechanisms during the ZnO NPs synthesis, which lead to formation of heterogeneous products, with varying morphology and chemical composition. Among various synthesis routes, the solvothermal method is noted as attractive for its simplicity and the possibility to have a better control of the reaction mechanism and the better rate of particle growth [402,753,754,755,756,757].

The microwave solvothermal synthesis enables also a ZnO NMs synthesis from a suspension, which consists in obtaining a suspension of a Zn^2+^ salt in an organic solvent with an addition of a substance precipitating the hydroxide anion precursor chemical “OH^−^”, e.g., NaOH.

### 4.5. Microwave Solvothermal Synthesis of ZnO from a Solution

**Figure 48:**
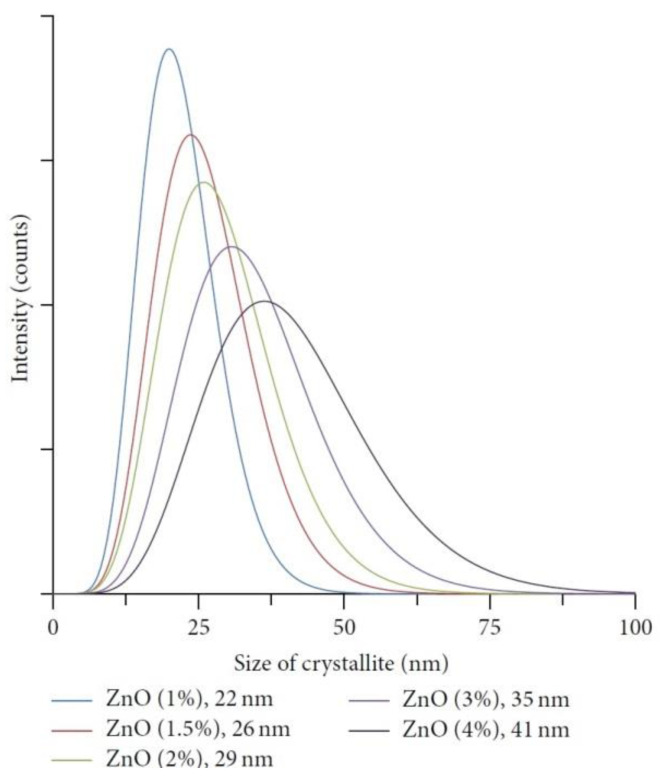
Crystallite size distribution of ZnO NPs obtained in the microwave solvothermal synthesis from precursor solutions with different H_2_O content. Reprinted with permission from [758], Copyright ©2016 Wojnarowicz et al, Article licensed under a CC BY 4.0, https://creativecommons.org/licenses/by/4.0/.

During our research on the microwave solvothermal synthesis we noticed that by changing the water content in the solution obtained by dissolving zinc acetate in ethylene glycol it is possible to control the size of ZnO NPs precisely within the range from ca. 15 nm to 120 nm [758]. Figure 48 presents an example size distribution of ZnO NPs crystallites obtained from precursor solutions with varying H_2_O contents. All microwave solvothermal syntheses were carried out with the same parameters (25 min, 230 °C). Due to the lack of access to research enabling us to analyse by-products, we proposed a general equation of the reaction (33) of ZnO NPs in ethylene glycol:

(33) $$\mathrm{Zn}{\left({\mathrm{CH}}_{3}\mathrm{COO}\right)}_{2}\cdot 2H_{2}O\overset{C_{2}H_{4}{\left(\mathrm{OH}\right)}_{2},{\;H}_{2}O,\;\mathrm{MH}\;\left(T,\;P\right)\;}{\to }\mathrm{ZnO}↓+\mathrm{other}\;\mathrm{products}\;\left(\mathrm{liquid}\;\mathrm{or}\;\mathrm{gas}\right)$$

We proved that by determining an experimental correlation between the average size of ZnO NPs and the water content in the precursor, a calibration curve of the ZnO NPs size is obtained, based on which particle size can be controlled. Of course, each calibration curve of ZnO NPs size refers to a specific lot of reactants for which it was determined. Each time, when changing the lot of reactants, the calibration curve of ZnO NPs size must be determined again to preserve the precision of particle size control.

**Figure 49:**
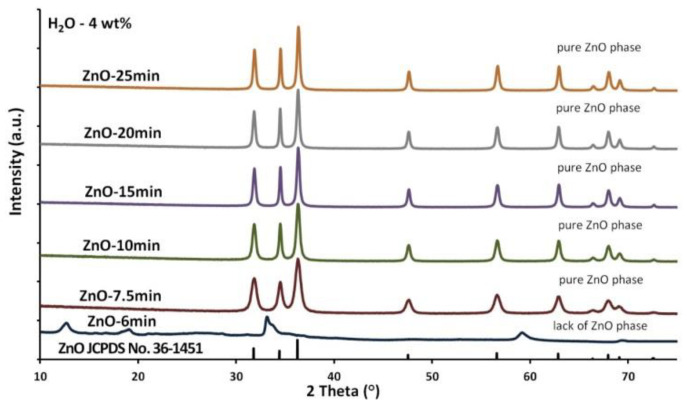
XRD diffraction patterns of ZnO NPs synthesis products obtained from the 4% H_2_O precursor, for synthesis durations of 6, 7.5, 10, 15, 20, 25 min and its comparison with the standard pattern of ZnO in wurtzite phase (JCPDS card No. 36-1451). Republished with permission of ©IOP Publishing from [402], Copyright (2018), permission conveyed through Copyright Clearance Center, INC. All rights reserved. In order to re-use permission must be obtained from the rightsholder.

**Figure 50:**
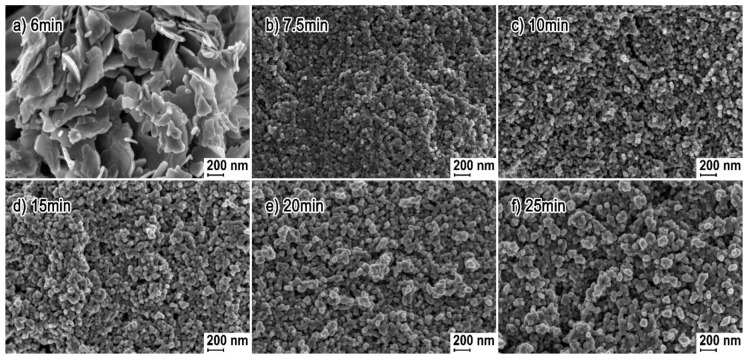
SEM images of ZnO NPs synthesis products obtained from the 4% H_2_O precursor, for synthesis durations of (**a**) 6 min; (**b**) 7.5 min; (**c**) 10 min; (**d**) 15 min; (**e**) 20 min; (**f**) 25 min, respectively. Republished with permission of ©IOP Publishing Ltd from [402], Copyright (2018), permission conveyed through Copyright Clearance Center, INC. All rights reserved. In order to re-use permission must be obtained from the rightsholder.

Thorough research on the microwave solvothermal synthesis of ZnO NPs obtained from a precursor solution, zinc acetate dissolved in ethylene glycol with an addition of heavy water, allowed us to explain and verify the synthesis mechanism [402]. It was crucial for our experiment to track and learn the fate of water at various stages of the synthesis. Owing to the results of XRD tests of ZnO NPs synthesis products for different durations (Figure 49) and the elemental analysis, we managed to determine that the solid intermediate of the solvothermal synthesis of ZnO was lamellar hydroxy-zinc acetate (LHZA, Zn_5_(OH)_8_(CH_3_COO)_2_·2H_2_O) with the lamellar structure (Figure 50a). Based on the results of the gas chromatography with mass spectrometry and Fourier transform infrared spectroscopy we proved that the liquid by-products were esters, mainly ethanediol monoacetate (HOC_2_H_4_OOCH_3_), and small quantities of ethanediol diacetate (C_2_H_4_(OOCH_3_)_2_). Thanks to the water content analysis by the Karl Fischer method, we determined that also water was one of the liquid by-products.

Learning all the primary products of the solvothermal synthesis of ZnO allowed us to write a general equation of the reaction (34):

(34) $$\mathrm{Zn}{\left({\mathrm{CH}}_{3}\mathrm{COO}\right)}_{2}+2C_{2}H_{4}{\left(\mathrm{OH}\right)}_{2}\overset{C_{2}H_{4}{\left(\mathrm{OH}\right)}_{2},{\;H}_{2}O,\;\mathrm{MH}\;\left(T,\;P\right)\;}{\to }\mathrm{ZnO}↓+H_{2}O+\left(2-m\right){\mathrm{HOC}}_{2}H_{4}{\mathrm{OOCH}}_{3}+{\mathrm{mC}}_{2}H_{4}{\left({\mathrm{OOCH}}_{3}\right)}_{2}$$

The above equation may be expressed in a more general way (35):

(35) $$\mathrm{Zn}{\left({\mathrm{CH}}_{3}\mathrm{COO}\right)}_{2}+\mathrm{alcohol}\overset{\;\left(T,\;P\right)\;}{\to }\mathrm{ZnO}↓+\mathrm{esters}+H_{2}O$$

An identical general equation of the reaction (35) was proposed by Du te al. [394], Tonto et al. [396], Šarić et al. [398], Zhao et al. [780], Yiamsawas et al. [840], and Yuan et al. [841], while the results achieved in the papers by Bilecka et al. [762], Bhatte et al. [769,776], and Demir [842] permit deriving a general equation independently (35). The results in the paper by Gotic et al. [843], which describes the solvothermal synthesis of Fe_3_O_4_ (hematite), and the results in the paper by Bilecka et al. [763], which describes the solvothermal synthesis of CoO, ZnO, Fe_3_O_4_, MnO and Mn_3_O_4_, permit deriving a general equation of the reaction (36) of synthesis of metal oxide (M_*x*_O_*z*_) NPs from a salt of a metal acetate (M(CH_3_COO)*_x_*) dissolved in an alcohol.

(36) $$M{\left({\mathrm{CH}}_{3}\mathrm{COO}\right)}_{x}+\mathrm{alcohol}\overset{\;\left(T,\;P\right)\;}{\to }M_{y}O_{z}+\mathrm{esters}+H_{2}O$$

**Figure 51:**
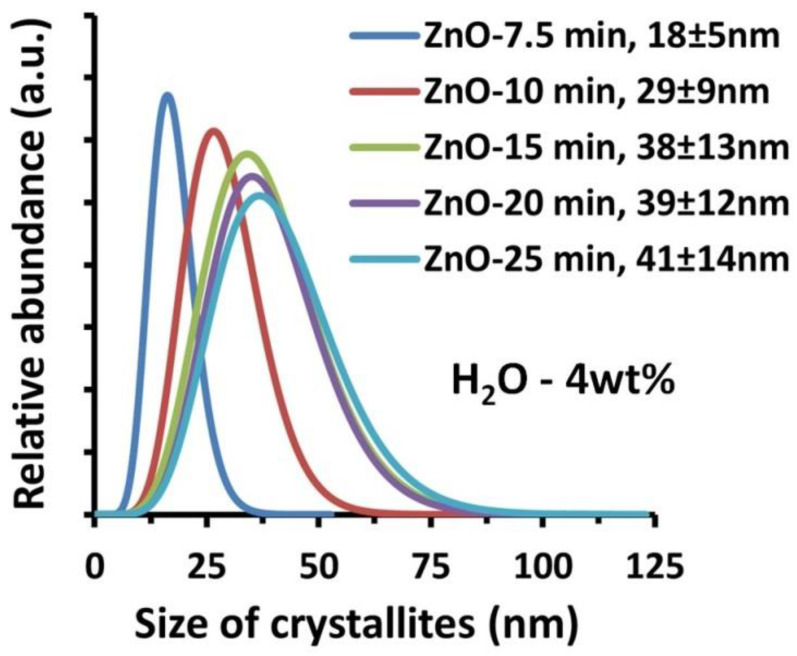
Crystallite size distribution for ZnO NPs samples obtained from the precursor with the 4% H_2_O content. Republished with permission of ©IOP Publishing from [402], Copyright (2018), permission conveyed through Copyright Clearance Center, INC. All rights reserved. In order to re-use permission must be obtained from the rightsholder.

Learning the fate of heavy water allowed us to explain the mechanism of ZnO NPs size control in the microwave solvothermal synthesis, which we described in detail in our paper [402]. The mechanism of size control of ZnO NPs obtained by the microwave solvothermal synthesis may be divided into 4 stages (Republished with permission of ©IOP Publishing from [402], Copyright (2018), permission conveyed through Copyright Clearance Center, INC. All rights reserved.):

(1) Dissolution of zinc acetate in ethylene glycol (37,38), preparation of the precursor with a specified H_2_O content (39)–(41):
  (37) $${\left({\mathrm{CH}}_{3}\mathrm{COO}\right)}_{2}\mathrm{Zn}\cdot 2H_{2}O_{\left(\mathrm{solid}\right)}\overset{70{}^{\circ}C,\;\mathrm{EG}\;}{\to }{\left({\mathrm{CH}}_{3}\mathrm{COO}\right)}_{2}\mathrm{Zn}\cdot 2H_{2}O_{\left(\mathrm{dissolved}\right)}$$
  (38) $${\left({\mathrm{CH}}_{3}\mathrm{COO}\right)}_{2}\mathrm{Zn}\cdot 2H_{2}O_{\left(\mathrm{solid}\right)}+\;2C_{2}H_{4}{\left(\mathrm{OH}\right)}_{2}\overset{70{}^{\circ}C,\;\mathrm{EG}\;}{\to }{\left({\mathrm{CH}}_{3}\mathrm{COO}\right)}_{2}\mathrm{Zn}\cdot 2C_{2}H_{4}{\left(\mathrm{OH}\right)}_{2}{}_{\left(\mathrm{dissolved}\right)}+2H_{2}O\;$$
  (39) $${\left({\mathrm{CH}}_{3}\mathrm{COO}\right)}_{2}\mathrm{Zn}\overset{{\;H}_{2}O,\;\mathrm{EG}}{↔}\left({\mathrm{CH}}_{3}\mathrm{COO}\right){\;\mathrm{Zn}}^{+}+{\;\mathrm{CH}}_{3}{\mathrm{COO}}^{-}$$
  (40) $${\mathrm{CH}}_{3}{\mathrm{COO}}^{-}+{\;H}_{2}O\overset{{\;H}_{2}O,\;\mathrm{EG}}{↔}{\mathrm{CH}}_{3}\mathrm{COOH}\;+{\;\mathrm{OH}}^{-}$$
  (41) $$\left({\mathrm{CH}}_{3}\mathrm{COO}\right){\mathrm{Zn}}^{+}+{\mathrm{OH}}^{-}\overset{{\;H}_{2}O,\;\mathrm{EG}}{↔}\left({\mathrm{CH}}_{3}\mathrm{COO}\right)\left(\mathrm{OH}\right)\mathrm{Zn}\;$$
(2) Formation (42)–(45) and growth of the intermediate (46):
  (42) $$5{\left({\mathrm{CH}}_{3}\mathrm{COO}\right)}_{2}\mathrm{Zn}+8C_{2}H_{4}{\left(\mathrm{OH}\right)}_{2}+xH_{2}O\;\overset{T,\;P\;}{\to }{\mathrm{Zn}}_{5}{\left(\mathrm{OH}\right)}_{8}{\left({\mathrm{CH}}_{3}\mathrm{COO}\right)}_{2}\cdot xH_{2}O_{\left(\mathrm{precipitation}\right)}+8{\mathrm{CH}}_{3}{\mathrm{COOC}}_{2}H_{4}\mathrm{OH}$$
  or possibly e.g.,
  (43) $$5{\left({\mathrm{CH}}_{3}\mathrm{COO}\right)}_{2}\mathrm{Zn}\cdot 2C_{2}H_{4}{\left(\mathrm{OH}\right)}_{2}+xH_{2}O\overset{T,\;P\;}{\to }{\mathrm{Zn}}_{5}{\left(\mathrm{OH}\right)}_{8}{\left({\mathrm{CH}}_{3}\mathrm{COO}\right)}_{2}\cdot xH_{2}O_{\left(\mathrm{precipitation}\right)}+8{\mathrm{CH}}_{3}{\mathrm{COOC}}_{2}H_{4}\mathrm{OH}+2C_{2}H_{4}{\left(\mathrm{OH}\right)}_{2}$$
  (44) $$5\left({\mathrm{CH}}_{3}\mathrm{COO}\right)\left(\mathrm{OH}\right)\mathrm{Zn}+3C_{2}H_{4}{\left(\mathrm{OH}\right)}_{2}+xH_{2}O\;\overset{T,\;P\;}{\to }{\mathrm{Zn}}_{5}{\left(\mathrm{OH}\right)}_{8}{\left({\mathrm{CH}}_{3}\mathrm{COO}\right)}_{2}\cdot xH_{2}O_{\left(\mathrm{precipitation}\right)}+3{\mathrm{CH}}_{3}{\mathrm{COOC}}_{2}H_{4}\mathrm{OH}\;\;\;$$
  (45) $$2\left({\mathrm{CH}}_{3}\mathrm{COO}\right)\left(\mathrm{OH}\right)\mathrm{Zn}\;+\;3{\left({\mathrm{CH}}_{3}\mathrm{COO}\right)}_{2}\mathrm{Zn}\;+\;xH_{2}O\;\overset{T,\;P\;}{\to }{\mathrm{Zn}}_{5}{\left(\mathrm{OH}\right)}_{8}{\left({\mathrm{CH}}_{3}\mathrm{COO}\right)}_{2}\cdot xH_{2}O_{\left(\mathrm{precipitation}\right)}+6{\mathrm{CH}}_{3}{\mathrm{COOC}}_{2}H_{4}\mathrm{OH}\;$$
  (46) $${\mathrm{Zn}}_{5}{\left(\mathrm{OH}\right)}_{8}{\left({\mathrm{CH}}_{3}\mathrm{COO}\right)}_{2}\cdot xH_{2}O+\;5{\left({\mathrm{CH}}_{3}\mathrm{COO}\right)}_{2}\mathrm{Zn}\;+\;8C_{2}H_{4}{\left(\mathrm{OH}\right)}_{2}+xH_{2}O\;\overset{T,\;P\;}{\to }\left(1+1\right){\mathrm{Zn}}_{5}{\left(\mathrm{OH}\right)}_{8}{\left(\mathrm{CH}3\mathrm{COO}\right)}_{2}\cdot xH_{2}O_{\left(\mathrm{growth}\right)}+8{\mathrm{CH}}_{3}{\mathrm{COOC}}_{2}H_{4}\mathrm{OH}$$
  *n*H_2_O comes from the simultaneous esterification reaction (47) or (48)
  (47) $$C_{2}H_{4}\left(\mathrm{OH}\right){\;}_{2}+{\mathrm{CH}}_{3}\mathrm{COOH}↔{\mathrm{CH}}_{3}{\mathrm{COOC}}_{2}H_{4}\mathrm{OH}+H_{2}O$$
  (48) $${\left({\mathrm{CH}}_{3}\mathrm{COO}\right)}_{2}\mathrm{Zn}\cdot 2C_{2}H_{4}{\left(\mathrm{OH}\right)}_{2}+{\mathrm{CH}}_{3}\mathrm{COOH}↔{\left({\mathrm{CH}}_{3}\mathrm{COO}\right)}_{2}\mathrm{Zn}\cdot C_{2}H_{4}{\left(\mathrm{OH}\right)}_{2}\cdot H_{2}O+{\;\mathrm{CH}}_{3}{\mathrm{COOC}}_{2}H_{4}\mathrm{OH}$$
(3) Achievement of equilibrium constant of the ester hydrolysis reaction for Equation (49) and at the same time of equilibrium constant of the esterification reaction (47) and decomposition of the intermediate caused by temperature (50):
  (49) $${\mathrm{HOC}}_{2}H_{4}{\mathrm{OOCH}}_{3}+{\;H}_{2}O↔C_{2}H_{4}{\left(\mathrm{OH}\right)}_{2}+{\mathrm{CH}}_{3}\mathrm{COOH}$$
  (50) $${\mathrm{Zn}}_{5}{\left(\mathrm{OH}\right)}_{8}{\left({\mathrm{CH}}_{3}\mathrm{COO}\right)}_{2}\cdot xH_{2}O+2C_{2}H_{4}{\left(\mathrm{OH}\right)}_{2}\;\overset{T,\;P\;}{\to }\;5\mathrm{ZnO}+2{\mathrm{CH}}_{3}{\mathrm{COOC}}_{2}H_{4}\mathrm{OH}+5H_{2}O+xH_{2}O\;$$
(4) Growth of existing ZnO NPs (51,52), which is confirmed by the results in Figure 51:
  (51) $$\mathrm{ZnO}+{\left({\mathrm{CH}}_{3}\mathrm{COO}\right)}_{2}\mathrm{Zn}+2C_{2}H_{4}{\left(\mathrm{OH}\right)}_{2}\overset{T,\;P\;}{\to }\left(1+1\right){\mathrm{ZnO}}_{\left(\mathrm{particle}\;\mathrm{growth}\right)}+H_{2}O+2{\mathrm{CH}}_{3}{\mathrm{COOC}}_{2}H_{4}\mathrm{OH}\;$$
  (52) $$\mathrm{ZnO}+\left({\mathrm{CH}}_{3}\mathrm{COO}\right)\left(\mathrm{OH}\right)\mathrm{Zn}+C_{2}H_{4}{\left(\mathrm{OH}\right)}_{2}\overset{T,\;P\;}{\to }\left(1+1\right){\mathrm{ZnO}}_{\left(\mathrm{particle}\;\mathrm{growth}\right)}+H_{2}O+{\mathrm{CH}}_{3}{\mathrm{COOC}}_{2}H_{4}\mathrm{OH}\;$$

The general equation of the microwave solvothermal synthesis reaction of ZnO NPs in ethylene glycol that takes into account obtaining only HOC_2_H_4_OOCH_3_ ester is as follows:

(53) $${\left({\mathrm{CH}}_{3}\mathrm{COO}\right)}_{2}\mathrm{Zn}+2C_{2}H_{4}{\left(\mathrm{OH}\right)}_{2}\overset{C_{2}H_{4}{\left(\mathrm{OH}\right)}_{2},{\;H}_{2}O,\;T,\;P\;}{\to }\mathrm{ZnO}+H_{2}O+2{\mathrm{CH}}_{3}{\mathrm{COOC}}_{2}H_{4}\mathrm{OH}$$

**Figure 52:**
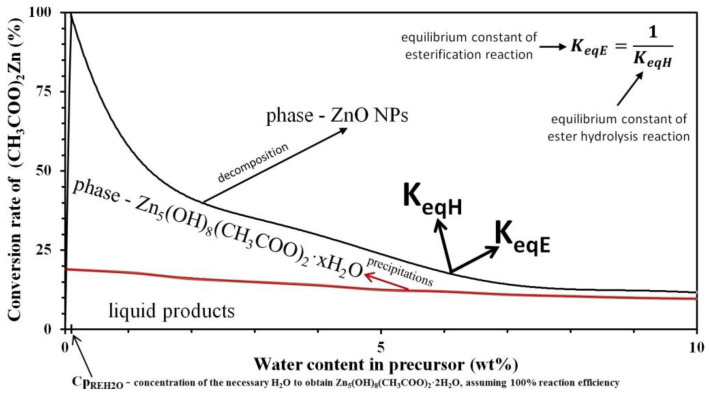
A reference phase diagram for the course of the ZnO NPs synthesis reaction, taking into account H_2_O content loss caused by zinc acetate hydrolysis. Republished with permission of ©IOP Publishing from [402], Copyright (2018), permission conveyed through Copyright Clearance Center, INC. All rights reserved. In order to re-use permission must be obtained from the rightsholder.

The detailed description of the above mechanism of the ZnO NPs synthesis, which is also presented in Figure 52, is included in the paper [402]. For the purposes of this review, we present a general description below:

1. As a result of hydrolysis of zinc acetate, water leads to the formation of acetic acid, which participates in an esterification reaction with ethylene glycol during the microwave solvothermal synthesis.
2. The products of the esterification reaction are esters and water. However, the course of the reaction of obtaining and growth of the intermediate, Zn_5_(OH)_8_(CH_3_COO)_2_·*x*H_2_O, is possible only through the co-existence of the esterification reaction. Only water forming in the esterification reaction participates in reactions of obtaining/growth of the intermediate, Zn_5_(OH)_8_(CH_3_COO)_2_·*x*H_2_O. Once the equilibrium constant of the esterification reaction is reached, the intermediate rapidly decomposes into ZnO NPs, H_2_O and esters.
3. The control of particle size arising from a change in the water content in the precursor is a consequence of the change in the quantity of formed crystalline nuclei of ZnO (NPs) relative to the remaining unconverted quantity of substrate (zinc acetate). After the decomposition of the intermediate into homogeneous nuclei of ZnO (NPs), no subsequent nuclei of ZnO (NPs) are formed as a result of further reactions. The only process that might occur is the growth of the existing nuclei of ZnO (NPs) until the still unreacted substrates are used up.
4. Water fulfils the function of a catalyst in the described ZnO NPs solvothermal synthesis reaction. Water participates in the reaction with substrates and forms an unstable intermediate, Zn_5_(OH)_8_(CH_3_COO)_2_·*x*H_2_O, which at the same time is a catalyst of the esterification reaction.

We believe that generally each ZnO solvothermal synthesis based on a precursor obtained as a result of dissolution of zinc acetate in an alcohol or a polyol will proceed according to the mechanism described by us above [402]. In this solvothermal synthesis, the alcohol/polyol fulfils a double function: the solvent of zinc acetate, and the reactant. Particular attention should be drawn to the fact that the lack of control over the H_2_O content in organic solvents will be manifested in the lack of repeatability of properties of the obtained ZnO NMs. It must be borne in mind that precursors obtained based on organic solvents must be always closed tightly because they absorb moisture from air/environment.

Other authors considered a mechanism of ZnO synthesis from zinc acetate dissolved in an alcohol but without analysing the solid intermediate, e.g., Bhatte et al. [769,776] suggest that Zn(OH)_2_ is the intermediate of the discussed solvothermal reaction of ZnO synthesis.

### 4.6. Microwave Solvothermal Synthesis of ZnO from a Suspension

The microwave solvothermal synthesis of ZnO NMs has one major advantage: once the reactants (Zn^2+^) have undergone a complete reaction, the further growth of ZnO NMs crystals through the Ostwald ripening process is considerably limited by the presence of the organic solvent. ZnO solubility in organic solvents is so low that it prevents recrystallisation. Of course, theoretically, the Ostwald ripening process may not take place where:

- content of, $m_{H_{2}O}=0\;\mathrm{wt}\%$,
- water being formed is collected physically or bound chemically,
- other substances which may digest/dissolve ZnO are not formed.

A limited Ostwald ripening process in the solvothermal synthesis enables obtaining ZnO NPs with the size below 10 nm (quantum dots), which is extremely rare in the case of hydrothermal syntheses. ZnO quantum dots are NPs with the size ranging from 2 to 10 nm.

The paper by Kuo et al. [774] describes obtaining ZnO NPs from a precursor formed by precipitation of Zn(CH_3_COO)_2_ dissolved in isopropanol, in an aqueous solution of NaOH. The authors [774] proposed the following general equation of the reaction (54):

(54) $${\left({\mathrm{CH}}_{3}\mathrm{COO}\right)}_{2}\mathrm{Zn}+\mathrm{NaOH}\overset{}{\to }\mathrm{ZnO}+H_{2}O+2\mathrm{Na}\left({\mathrm{CH}}_{3}\mathrm{COO}\right)$$

The product of their synthesis were spherical ZnO NPs with the average size of 3.5 nm.

The paper by Saloga et al. [795] describes obtaining ZnO NPs through a multi-stage synthesis. First, their prepared zinc oleate by mixing ZnCl_2_ with sodium oleate, which they rinsed with water and dried. Next, dry zinc oleate was dissolved in tetrahydrofuran and then tetrabutylammonium hydroxide was added to the solution. Thus obtained suspension was subjected to a microwave synthesis lasting 5 min at the temperatures of 125 °C, 150 °C, 175 °C and 200 °C. Saloga et al. [795] describe obtaining ZnO NPs with the average size of 2.6 nm (125 °C), 2.7 nm (150 °C), 3.1 nm (175 °C), and 3.8 nm (200 °C).

**Figure 53:**
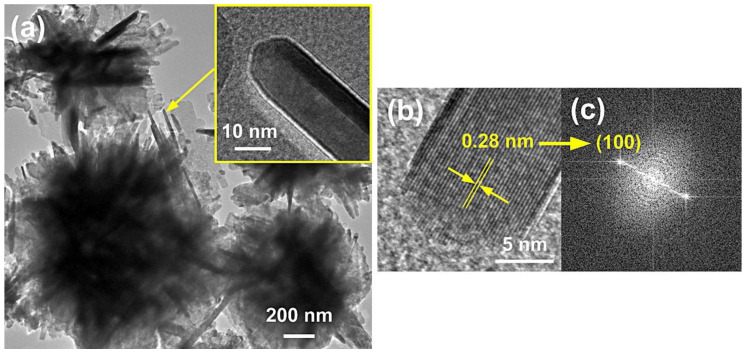
TEM micrographs of (**a**) as-prepared ZnO nanoparticles, (**b**) HRTEM micrograph showing the lattice fringes in the ZnO nano-crystal with its SAED (selected area electron diffraction) pattern as shown in (**c**). Inset in (**a**) shows that the prepared ZnO nanoparticle sample is mostly composed of small nanorods with the diameter of 10–20 nm. Reprinted from [705], Copyright (2014), with permission from Elsevier [OR APPLICABLE SOCIETY COPYRIGHT OWNER]. All rights reserved. In order to re-use permission must be obtained from the rightsholder.

Saoud et al. [705] obtained nanorods from a suspension of a precursor obtained by dissolving Zn(NO_3_)_2_ in ethanol, subsequently adding such a quantity of the NaOH solution until the pH of the obtained solution reached 10. The suspension was exposed to microwave irradiation for approximately 10–15 min and was removed upon onset of boiling. The synthesis product was nanorods with diameters ranging from 10 to 20 nm (Figure 53).

Details of other research papers concerning the microwave solvothermal synthesis of ZnO without any additional heat treatment are presented in Table 10.

### 4.7. Types of ZnO Nanocomposites or ZnO Hybrid Nanostructures Obtained by the Solvothermal Synthesis

The microwave solvothermal synthesis enables obtaining:

- ZnO doped with the following ions: Al^3+^ [811,814,815], Co^2+^ [232,798,799,800,801,808,809,810,812,833,834,835], Cr^3+^ [232,809], Fe^2+^ [232,810,836], Ga^3+^ [815], In^3+^ [811], Mg^2+^ [817], Mn^2+^ [232,802,804,809,810,812,837], Ni^2+^ [232,809,810,813] and V^5+^ [810].
- ZnO co-doped with the following ions: Co^2+^–Mn^2+^ [803] and Al^3+^–Ga^3+^ [815].
- ZnO composites or ZnO hybrid structures: Ag–ZnO [181,805,806,838], Au–ZnO [807], Fe_3_O_4_@SiO_2_/ZnO [818], coaxial ZnO/C/CdS nanocables [819], ZnO/reduced graphene oxide [819,821], Ag/ZnO/reduced graphene oxide [821], and carbon-coated ZnO nanorods [822].

### 4.8. ZnO Nanocomposites or ZnO Hybrid Nanostructures Obtained by the Microwave Solvothermal Synthesis without Any Additional Heat Treatment

One of the main advantages of the solvothermal synthesis of doped ZnO is the multifunctionality of the organic solvent, which may fulfil the function of a reactant, a dopant stabilising agent, limit the uncontrolled growth of particles, and enables a precise control of the particle size. 

Our research [798,799,801] proves that the solvothermal synthesis, through selection of appropriate reactants (Zn(CH_3_COO)_2_·2H_2_O, Co(CH_3_COO)_2_·4H_2_O, C_2_H_4_(OH)_2_), enables obtaining Co doped ZnO with the nominal content of Co^2+^ ions being at least 15 mol% in the form of a single-phase material (without foreign phase inclusions). We used ethylene glycol, which has slightly reducing properties, which enabled stabilisation of the oxidation state of the Co^2+^ dopant during the synthesis. Co doped ZnO obtained by us displayed paramagnetic properties with a contribution of antiferromagnetic coupling of Co–Co pairs [798]. We also confirmed that the method, which we discovered, of ZnO particle size control in the microwave solvothermal synthesis [402,758] permitted also control of Co doped ZnO particle size within the size range of 20–53 nm.

**Figure 54:**
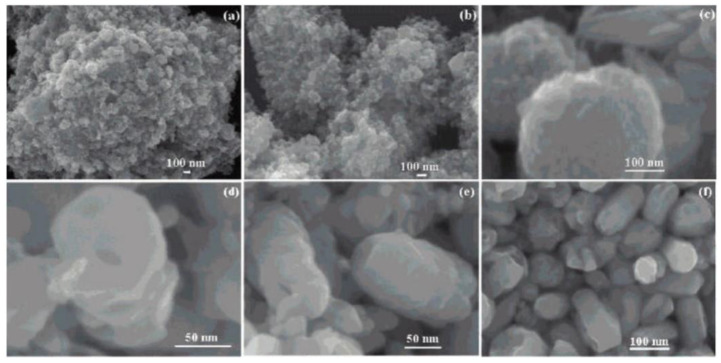
SEM images of (**a**) Ag:ZnO molar ratio of 0, (**b**) Ag:ZnO molar ratio of 0.02, (**c**) Ag:ZnO molar ratio of 0.05, (**d**,**e**) Ag:ZnO molar ratio of 0.13, (**f**) Ag:ZnO molar ratio of 0.22. Reprinted (adapted) with permission from [805]. Copyright (2008) American Chemical Society. All rights reserved. In order to re-use permission must be obtained from the rightsholder.

**Figure 55:**
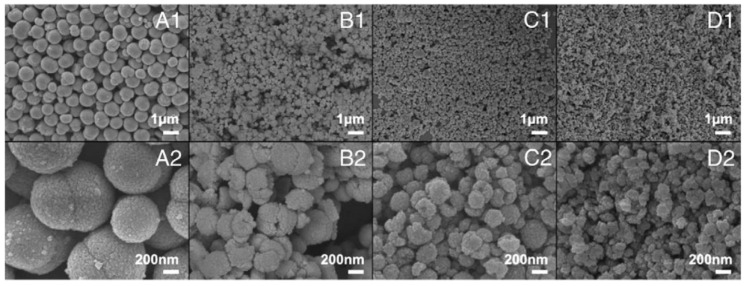
SEM images of ZnO: (**A**) pure, (**B**) 0.5% Ag, (**C**) 1.0% Ag and (**D**) 3.0% Ag. Reprinted from [806], Copyright (2012), with permission from Elsevier [OR APPLICABLE SOCIETY COPYRIGHT OWNER]. All rights reserved. In order to re-use permission must be obtained from the rightsholder.

Bhattacharyya et al. [805] and Wang et al. [806] described obtaining an Ag–ZnO composite with the use of a mixture containing identical components: (Zn(CH_3_COO)_2_·2H_2_O, AgNO_3_, C_2_H_4_(OH)_2_ (solvent), H_2_O and C_2_H_4_(OH)_2_ (solvent)). Bhattacharyya et al. [805] carried out 15-min syntheses in a domestic microwave oven modified with a refluxing system, while Wang et al. [806] carried out 30-min syntheses at the temperature of 170 °C in a microwave reactor. Bhattacharyya et al. [805] obtained an Ag–ZnO composite depending on the Ag content (Ag:ZnO molar ratio of 0.02, 0.05, 0.15, and 0.22) with the nanodisk and nanorod shape (Figure 54), where Ag NPs sized 3.5 nm were inserted into the pores of ZnO. Wang et al. [806], in turn, obtained an Ag–ZnO composite composed of microspheres (Figure 55), where the increase in the content of Ag NPs with the diameter below 5 nm from 0.5 mol% to 3 mol% resulted in a decrease in the size of irregular semi-circular microspheres. 

**Figure 56:**
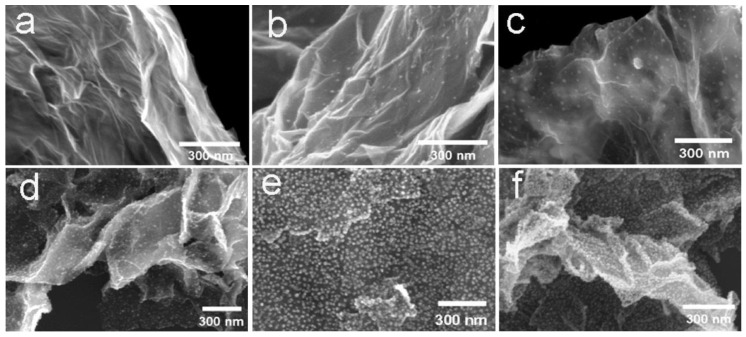
SEM images of as-prepared (**a**) reduced graphene oxide (rGO) sheets and rGO/ZnO nanohybrids obtained with different concentrations of Zn^2+^: (**b**) 0.0023 M, (**c**) 0.0046 M, (**d**) 0.0069 M, (**e**) 0.0092 M, and (**f**) 0.0115 M. Reprinted from [820], Copyright (2012), with permission from Elsevier [OR APPLICABLE SOCIETY COPYRIGHT OWNER]. All rights reserved. In order to re-use permission must be obtained from the rightsholder.

**Figure 57:**
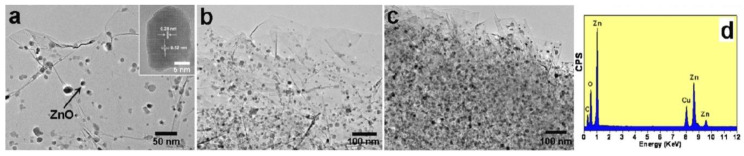
TEM images of as-prepared rGO/ZnO nanohybrids obtained with different concentrations of Zn^2+^: (**a**) 0.0023 M, (**b**) 0.0069 M and (**c**) 0.0115 M, (**d**) EDS pattern of the rGO/ZnO nanohybrids. Reprinted from [820], Copyright (2012), with permission from Elsevier [OR APPLICABLE SOCIETY COPYRIGHT OWNER]. All rights reserved. In order to re-use permission must be obtained from the rightsholder.

One of the most interesting papers is the one by Liu et al. [820], which describes obtaining reduced graphene oxide sheets covered with ZnO NPs. As the synthesis precursor, zinc acetate dissolved in diethylene glycol with the addition of reduced graphene oxide sheets was used. Synthesis reactions lasting 10 min were carried out in a microwave refluxing system. Products prepared with different amounts of zinc acetate 0.0023, 0.0046, 0.0069, 0.0092, and 0.0115 M. By changing the zinc acetate concentration, the authors controlled the degree of coverage of the reduced graphene oxide sheets surface, which is presented in Figure 56 and Figure 57. The authors [820] successfully obtained the ZnO NPs (10–14 nm) content in ZnO/reduced graphene oxide samples from 11.55 wt% to 26.89 wt%.

Details of other research papers concerning the ZnO nanocomposites or ZnO hybrid nanostructures obtained by the solvothermal synthesis without any additional heat treatment are presented in Table 11.

### 4.9. ZnO Nanocomposites or ZnO Hybrid Nanostructures Obtained by the Solvothermal Synthesis with Additional Heat Treatment

**Figure 58:**
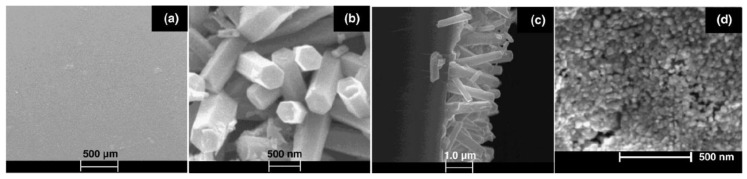
(**a**,**b**) Plan view section SEM images of a thin film of ZnO nanorods, (**c**) cross-section image of ZnO nanorods, (preparation condition—800 W power, 5 min) and (**d**) plan view section SEM image of a ZnO nanoparticle thin film coating on Si(100), (preparation condition—800 W power, 20 s). Reprinted from [825], Copyright (2010), with permission from Elsevier [OR APPLICABLE SOCIETY COPYRIGHT OWNER]. All rights reserved. In order to re-use permission must be obtained from the rightsholder.

One of the main purposes of additional heat treatment of microwave solvothermal synthesis products, among others, undoped ZnO NMs in the solvothermal synthesis according to the publication [823,824,825,826,827,828,829,830,831], was to remove unconverted reactants. A perfect example here is the cycle of papers by Brahma et al. [825,826,827,828,829,830], which describe the growth of ZnO nanorods on an amorphous or disordered surface (silica, glass and polymer substrates). Brahma et al. [825] used a mixture obtained by mixing zinc acetylacetonate dissolved in ethanol with polyvinylpyrrolidone dissolved in water for the synthesis. The obtained mixture together with the silica substrate immersed therein was introduced for a duration between 20 s and 5 min (800 W) in a domestic-type microwave oven equipped with a water-cooled condenser (reflux system) placed outside the microwave oven. After cooling the suspension, the substrates were removed, rinsed and dried. For the purposes of removing the surfactant residue, the covered substrates were soaked (2–5 min) at 500 °C in the air atmosphere. An example of the obtained morphology of the ZnO film grown on Si(100) is presented in Figure 58.

**Figure 59:**
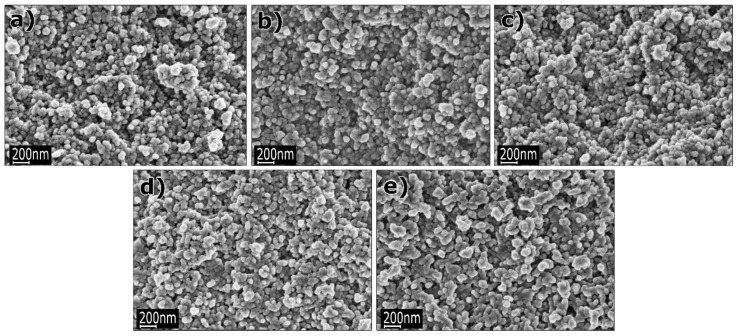
SEM images of ZnO nanopowders in their as-produced (before annealing) state: (**a**) undoped, (**b**) doped with 1 mol% of Co^2+^, (**c**) 5 mol% of Co^2+^, (**d**) 10 mol% of Co^2+^, and (**e**) 15 mol% of Co^2+^ ions. Reprinted with permission from [798], Copyright ©2015 Wojnarowicz et al., Article licensed under a CC BY 2.0.

**Figure 60:**
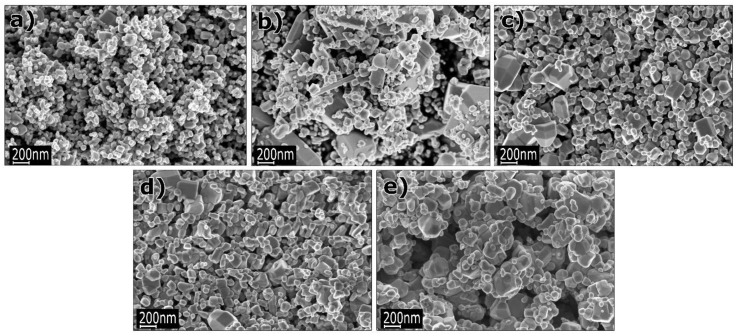
SEM images of annealed ZnO nanopowders after annealing at 800 °C in nitrogen: (**a**) undoped, (**b**) doped with 1 mol% of Co^2+^, (**c**) 5 mol% of Co^2+^, (**d**) 10 mol% of Co^2+^, and (**e**) 15 mol% of Co^2+^ ions. Reprinted with permission from [798], Copyright ©2015 Wojnarowicz et al., Article licensed under a CC BY 2.0.

**Figure 61:**
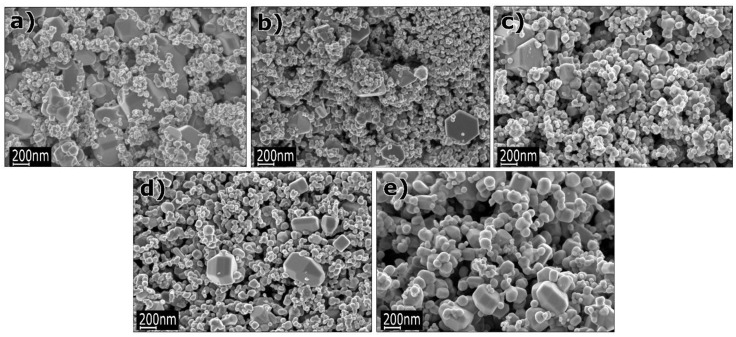
SEM images of annealed ZnO nanopowders after annealing at 800 °C in synthetic air: (**a**) undoped, (**b**) doped with 1 mol% of Co^2+^, (**c**) 5 mol% of Co^2+^, (**d**) 10 mol% of Co^2+^, and (**e**) 15 mol% of Co^2+^ ions. Reprinted with permission from [798], Copyright ©2015 Wojnarowicz et al., Article licensed under a CC BY 2.0.

In the case of a microwave solvothermal synthesis of doped ZnO, additional heat treatment of samples may lead to an increase in the ZnO particle size (recrystallisation process), oxidation or reduction of dopant ions and formation of foreign inclusions. The results of our research [798] show the effect of soaking ZnO doped with Co^2+^ ions. We obtained Co doped ZnO from a solution obtained by dissolving a mixture of zinc acetate with cobalt acetate (II) in ethylene glycol. We performed syntheses lasting 25 min at the temperature of 220 °C in a microwave reactor. Figure 59 shows a homogeneous product of Zn_1−*x*_Co*_x_*O NPs synthesis (*x* = 0, 0.01, 0.05, 0.10, 0.15) in the form of spherical particles with the size of 30–40 nm without foreign phase inclusions. The obtained samples were subjected to additional soaking for 0.5 h at 800 °C in an oxidising (synthetic air, Figure 60) and a reducing (nitrogen, Figure 61) atmosphere. XRD tests proved the presence of a Co_3_O_4_ phase in the Zn_0.85_Co_0.15_O sample soaked in air, while the presence of metallic Co in the Zn_0.95_Co_0.05_O, Zn_0.90_Co_0.10_O and Zn_0.85_Co_0.15_O samples soaked in nitrogen. Co^2+^ ions underwent oxidation to Co^3+^ ions during the soaking in synthetic air, which is presented by the equation of the reaction (55).

(55) $$4{\mathrm{Co}}^{2+}+O_{2}\to 4{\mathrm{Co}}^{3+}+2O^{2-}$$

Co^2+^ ions underwent reduction to metallic Co^0^ in nitrogen, which is presented by the equation of the reaction (56).

(56) $$2{\mathrm{Co}}^{2+}+N_{2}\to 2{\mathrm{Co}}^{0}+2N^{2+}$$

The soaking of the Zn_1−*x*_Co*_x_*O NPs samples resulted in:

- a change in their specific surface area from 37–39 m^2^/g to merely 3 m^2^/g,
- an increase in the particle size from the range of 30–40 nm to the range of 50–2000 nm depending on the amount of Co,
- a change in the morphology from homogeneous spherical NPs (Figure 59) to heterogeneous hexagonal or cubic NPs (Figure 60 and Figure 61).

Details of other research papers concerning the ZnO nanocomposites or ZnO hybrid nanostructures obtained by the solvothermal synthesis with additional heat treatment are presented in Table 12.

## 5. Microwave Hybrid Synthesis of ZnO

Microwave hybrid syntheses of ZnO NMs are divided into two following groups:

(1) Microwave hybrid synthesis method of pure ZnO nano and microstructures, where the literature review results [844,845,846,847,848,849,850,851,852,853,854,855,856,857,858,859,860,861,862,863,864,865,866,867,868,869,870,871,872,873,874,875,876,877,878,879,880,881,882,883,884,885,886] are summarised in Table 13.
(2) Microwave hybrid synthesis method of ZnO composites or ZnO hybrid structures, where the literature review results [887,888,889,890,891,892,893,894,895,896,897,898,899,900,901,902,903,904,905,906,907,908,909,910,911,912,913,914,915,916,917,918,919,920,921,922,923,924,925,926,927] are summarised in Table 14.

The following were used for obtaining ZnO nano- and microstructures in the literature:

(1) Ultrasonic microwave synthesis, which consists in the use of a new generation of microwave reactors, which permit the presence of an ultrasonic homogeniser’s sonotrode in the precursor mixture during the microwave heating. The ultrasonic homogeniser during its operation converts electrical energy into mechanical energy by moving the tip of the titanium sonotrode immersed in the fluid with a high frequency (19.5–40 kHz). Due to its inertia, the fluid no longer catches up with the rapid motion of the sonotrode, which results in cavitation, i.e., formation of gas bubbles that rapidly collapse, which is accompanied by sudden pressure changes, and as a consequence creates an impact wave.
(2) Microwave assisted combustion synthesis, which consists in an exothermic reaction of combustion of one of the reactants of the reaction mixture in an oxygen atmosphere. Generally, a mixture composed among others of a Zn^2+^ salt and an organic component (fuel) is thoroughly mixed. There are several possibilities of the final state of the reaction mixture, among others, powder, pressed pastilles, gel, emulsion. The ready reaction mixture is introduced to a microwave reactor or oven, subjected to microwave radiation, which leads to a rapid increase in the sample temperature and ignition of the fuel, resulting in the formation of a ZnO powder.
(3) Microwave assisted annealing, which consists in decomposition of the reaction mixture to ZnO only under the influence of its heating as a result of microwave radiation.
(4) Microwave assisted sintering, which consists in microwave soaking of the earlier obtained ZnO.
(5) Microwave vapour deposition, which consists in ZnO deposition from a gaseous phase, mostly at the atmospheric pressure, on the wafer (substrate) surface. For example, powdered ZnO, Zn or a Zn^2+^ salt is introduced to a ceramic crucible made of Al_2_O_3_, which is closed with a cover to which the substrate is attached on its inside part. Under the influence of microwave heating, a plasma arc appears in the crucible, enabling the evaporation of the Zn^2+^ substrate, which is deposited at the same time in the form of thin films on the whole surface of the ceramic container in the form of ZnO. Of course, there are professional microwave based plasma deposition units, which enable the application of inert carrier gases (e.g., argon, helium) or such gases (e.g., O_2_) that can participate in chemical reactions leading to the formation of ZnO layers.

### 5.1. Reactants

**Figure 62:**
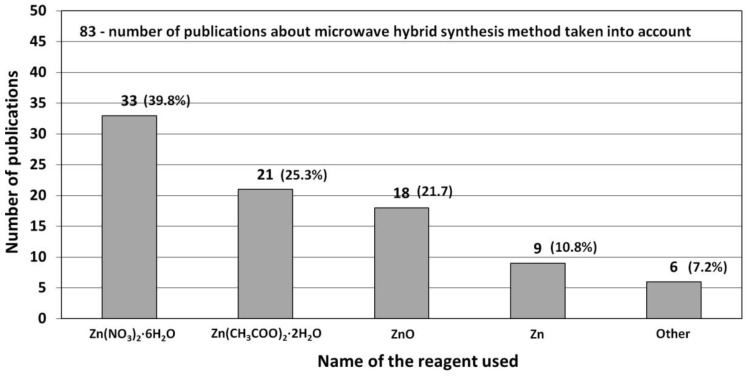
Statistics of use of reactants in the microwave hybrid synthesis method of ZnO. Source: [844,845,846,847,848,849,850,851,852,853,854,855,856,857,858,859,860,861,862,863,864,865,866,867,868,869,870,871,872,873,874,875,876,877,878,879,880,881,882,883,884,885,886,887,888,889,890,891,892,893,894,895,896,897,898,899,900,901,902,903,904,905,906,907,908,909,910,911,912,913,914,915,916,917,918,919,920,921,922,923,924,925,926,927].

The most popular reactants of the “Zn^2+^” zinc cation precursor used for the hybrid ZnO synthesis according to the data derived from the literature review [844,845,846,847,848,849,850,851,852,853,854,855,856,857,858,859,860,861,862,863,864,865,866,867,868,869,870,871,872,873,874,875,876,877,878,879,880,881,882,883,884,885,886,887,888,889,890,891,892,893,894,895,896,897,898,899,900,901,902,903,904,905,906,907,908,909,910,911,912,913,914,915,916,917,918,919,920,921,922,923,924,925,926,927] (Table 13 and Table 14) are Zn(NO_3_)_2_·6H_2_O, Zn(CH_3_COO)_2_·2H_2_O, ZnO, metallic Zn, and others (Figure 62). Other reactants are ZnCl_2_, ZnSO_4_, and Zn(OH)_2_.

### 5.2. Morphology

Morphologies of ZnO NMs reported in the literature are presented in Table 13 and Table 14 [844,845,846,847,848,849,850,851,852,853,854,855,856,857,858,859,860,861,862,863,864,865,866,867,868,869,870,871,872,873,874,875,876,877,878,879,880,881,882,883,884,885,886,887,888,889,890,891,892,893,894,895,896,897,898,899,900,901,902,903,904,905,906,907,908,909,910,911,912,913,914,915,916,917,918,919,920,921,922,923,924,925,926,927] along with a description of their properties. ZnO NMs synthesised with the use of the microwave hybrid method were characterised by the following shapes: block-shaped structures [859], cables [899], corn-like microstructures [862], cubes [925], fibres [871], fluff-like structures [923], foamy and porous structures [850], half-backed grenade-like structures [844], hourglass-like structures [845], nails [878], wrinkle structures [923], spindle-like structures [844,888], spindle-like to double-prism-like structures [844], tetrapods [883], platelets [849], needles [886,925], plates [902,903], sheet-like structures [855,877,889], flakes [853,902,903,925], flower-like structures [847,859,881,887], tubes [868,869,870,899,916], wires [867,874,880,883,899], layers (films) [856,857,858,865,873,891,900,912,913], rods [852,861,881,883,889,907,919,920], and spherical particles [846,848,851,861,863,864,884,885,889,893,905,908,910,921].

**Figure 63:**
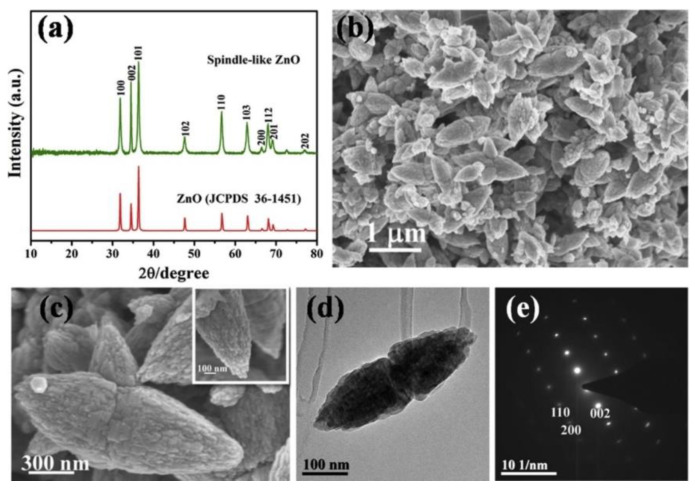
X-ray powder diffraction (XRD) pattern (**a**), SEM (**b**,**c**), TEM (**d**) images and satisfactory selected area electron diffraction (SAED) pattern (**e**) of the as-prepared spindle-like ZnO. Products synthesised by microwave and ultrasonic wave combined method. Reprinted from [888], Copyright (2019), with permission from Elsevier [OR APPLICABLE SOCIETY COPYRIGHT OWNER]. All rights reserved. In order to re-use permission must be obtained from the rightsholder.

**Figure 64:**
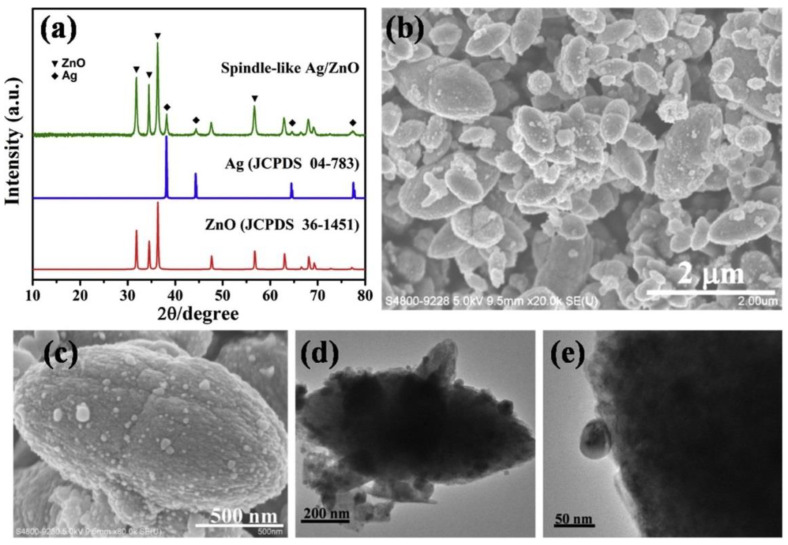
XRD pattern (**a**), SEM (**b**,**c**) and TEM (**d**,**e**) images of spindle-like Ag/ZnO nanocomposites. Products synthesised by the microwave and ultrasonic wave combined method. Reprinted from [888], Copyright (2019), with permission from Elsevier [OR APPLICABLE SOCIETY COPYRIGHT OWNER]. All rights reserved. In order to re-use permission must be obtained from the rightsholder.

**Figure 65:**
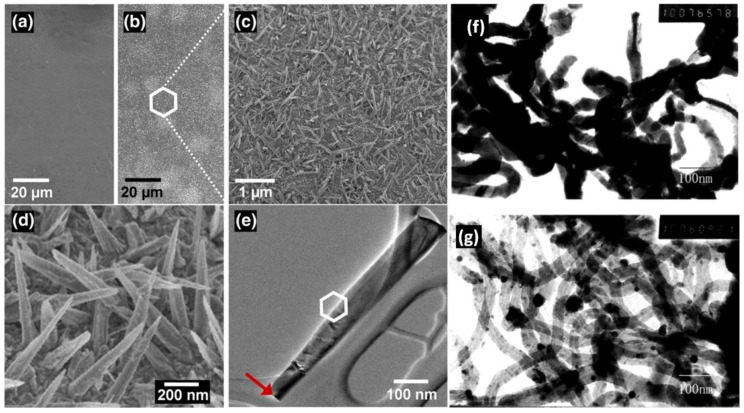
(**a**–**e**) SEM images of the surface of the Zn sheet: (**a**) Low-magnified image before the microwave irradiation. (**b**) Low-magnified image after the microwave irradiation. (**c**) Medium-magnified image after the microwave irradiation. (**d**) High-magnified image after the microwave irradiation; (**e**) TEM image of a ZnO nanoneedle. Products synthesised by direct microwave irradiation on the Zn sheet under O_2_ and Ar atmosphere (total pressure: 1 atm). Reprinted from [886], Copyright (2008), with permission from Elsevier [OR APPLICABLE SOCIETY COPYRIGHT OWNER]. All rights reserved. In order to re-use permission must be obtained from the rightsholder. (**f**–**g**) TEM images of (**f**) Zn-ZnO nanocables and (**g**) ZnO nanotubes. Reprinted (adapted) with permission from [899]. Products synthesised by microwave vapour deposition. Copyright © 2003, American Chemical Society. All rights reserved. In order to re-use permission must be obtained from the rightsholder.

**Figure 66:**
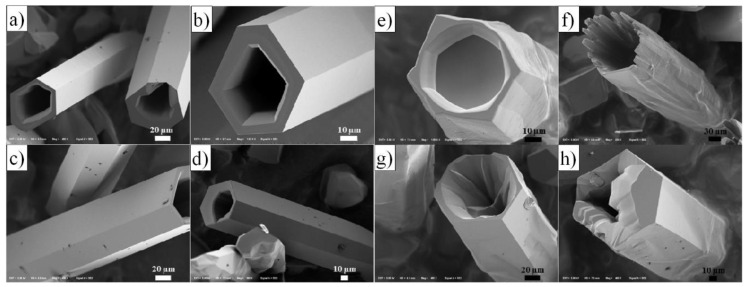
(**a**–**d**) FESEM images display the shape and morphology of the microwave grown ZnO microtubes. (**a**,**b**) FESEM images showing the outer hexagonal faceted surfaces and the inner smooth and stepped surface of the ZnO microtubes. (**c**,**d**) FESEM images showing the side facets and lengths of ZnO microtubes. (**e**–**h**) FESEM images of ZnO, (**e**,**f**) semi-microtube at higher temperature and (**g**,**h**) randomly oriented, tube-like structures of ZnO. Products synthesised by microwave assisted sintering. Reprinted from [869], Copyright (2015), with permission from Elsevier [OR APPLICABLE SOCIETY COPYRIGHT OWNER]. All rights reserved. In order to re-use permission must be obtained from the rightsholder.

**Figure 67:**
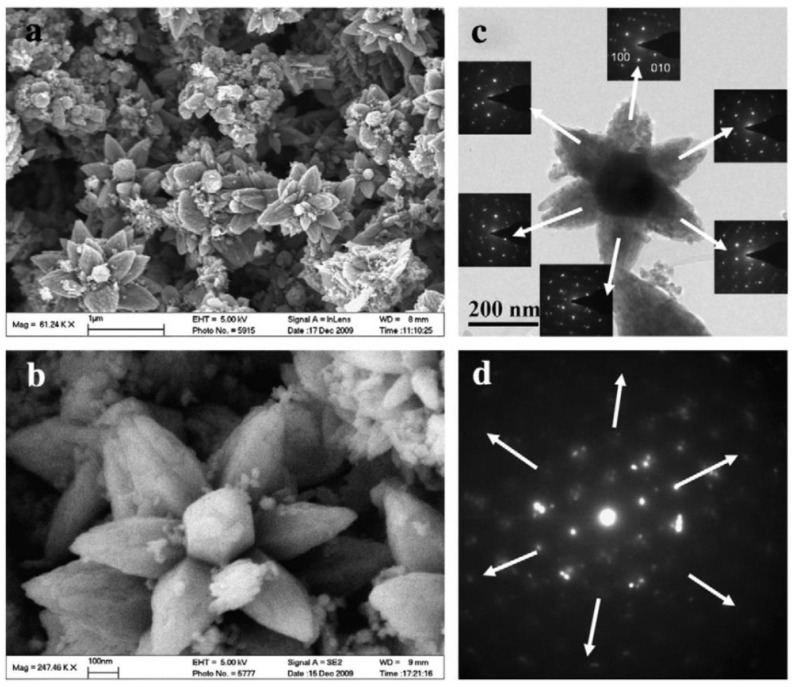
SEM images (**a**,**b**), TEM image (**c**) and SAED patterns (**d** and insets of **c**) of the flower-like ZnO nanostructures. Products synthesised by the microwave and ultrasonic wave combined method. Reprinted from [887], Copyright (2011), with permission from Elsevier [OR APPLICABLE SOCIETY COPYRIGHT OWNER]. All rights reserved. In order to re-use permission must be obtained from the rightsholder.

**Figure 68:**
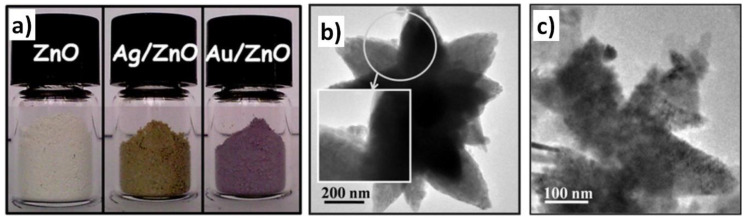
Photographs (**a**) of (Au, Ag)/ZnO nanocomposites; TEM images of Ag/ZnO nanostructures (**b**) and Au/ZnO nanostructures (**c**). Products synthesised by the microwave and ultrasonic wave combined method. Reprinted from [887], Copyright (2011), with permission from Elsevier [OR APPLICABLE SOCIETY COPYRIGHT OWNER]. All rights reserved. In order to re-use permission must be obtained from the rightsholder.

**Figure 69:**
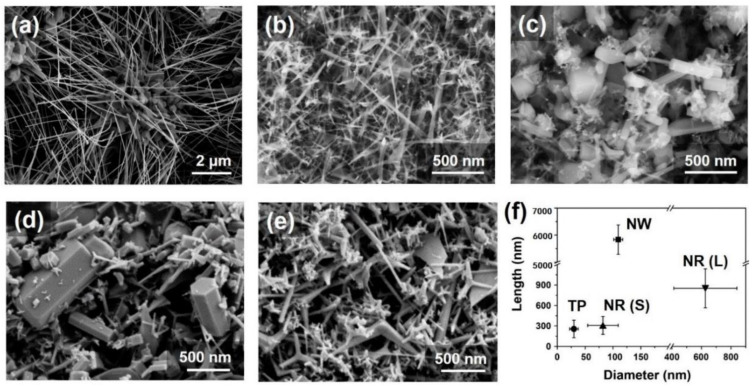
SEM images of synthesised ZnO nanomaterials using (**a**,**b**) compressed air, (**c**) high-purity air, (**d**) O_2_, and (**e**) O_2_/N_2_ mixed gas. (**f**) Diagram shows length versus diameter of synthesised ZnO nanomaterials (NW; nanowire, TP; tetrapod, and NR (S), (L); small and large nanorods). Products were synthesised using a microwave plasma torch system at the atmospheric pressure. Reprinted with permission from [883], Copyright ©2019 Lee et al., Article licensed under a CC BY 4.0, https://creativecommons.org/licenses/by/4.0/.

Examples of various morphologies of ZnO obtained by the microwave hybrid synthesis are presented in Figure 63, Figure 64, Figure 65, Figure 66, Figure 67, Figure 68 and Figure 69.

### 5.3. Synthesis of Pure ZnO by the Microwave Hybrid Method

**Figure 70:**
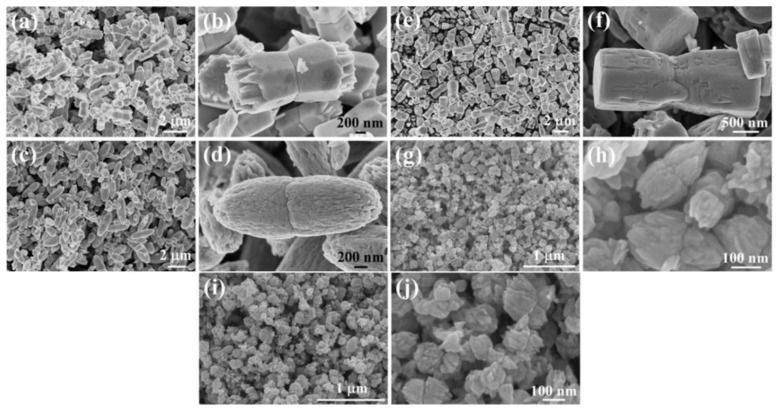
SEM images of ZnO products prepared under different Zn/2-[4-(2-hydroxyethyl)-1-piperazinyl]ethanesulfonic acid molar ratios: 3:8 (**a**,**b**); 1:4 (**c**,**d**); 1:8 (**e**,**f**); 1:20 (**g**,**h**); 1:40 (**i**,**j**). Products synthesised the by microwave and ultrasonic wave combined method. Reprinted from [844], Copyright (2013), with permission from Elsevier [OR APPLICABLE SOCIETY COPYRIGHT OWNER]. All rights reserved. In order to re-use permission must be obtained from the rightsholder.

**Figure 71:**
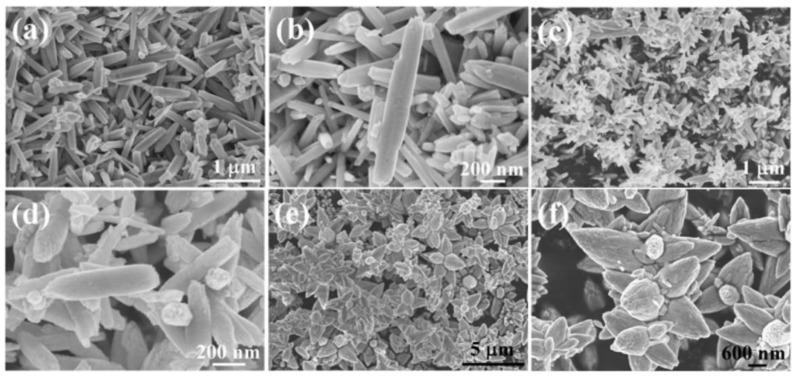
SEM images of ZnO products synthesised from zinc acetylacetonate hydrate with the Zn/HEPES molar ratio of 1:5 prepared under different pH values: 7.4 (**a**,**b**); 8.4 (**c**,**d**); 9.4 (**e**,**f**). Products synthesised by the microwave and ultrasonic wave combined method. Reprinted from [844], Copyright (2013), with permission from Elsevier [OR APPLICABLE SOCIETY COPYRIGHT OWNER]. All rights reserved. In order to re-use permission must be obtained from the rightsholder.

**Figure 72:**
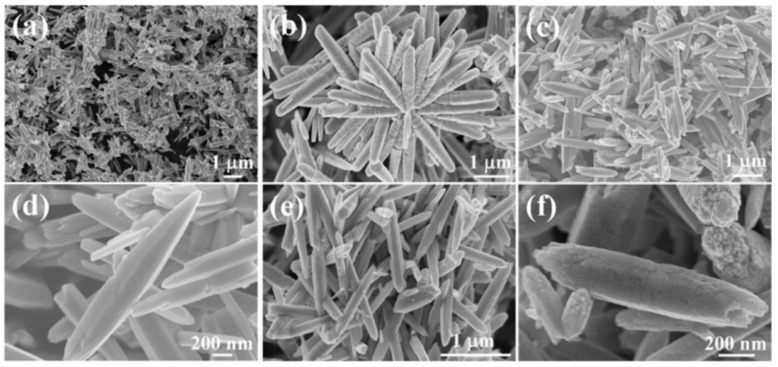
SEM images of ZnO products synthesised from zinc acetylacetonate hydrate with the Zn/HEPES molar ratio of 1:2 under different pH values: 7.4 (**a**,**b**); 8.4 (**c**,**d**); 9.4 (**e**,**f**). Products synthesised by the microwave and ultrasonic wave combined method. Reprinted from [844], Copyright (2013), with permission from Elsevier [OR APPLICABLE SOCIETY COPYRIGHT OWNER]. All rights reserved. In order to re-use permission must be obtained from the rightsholder.

An example of an interesting publication reporting results of the microwave and ultrasonic wave combined synthesis of ZnO micro-/nanostructures is the paper by Li et al. [844]. As the synthesis precursor, a solution obtained by mixing solutions of Zn^2+^ salts (zinc acetate dehydrate, zinc acetylacetonate hydrate, zinc chloride) with 2-[4-(2-hydroxyethyl)-1-piperazinyl]ethanesulfonic acid (HEPES) was used. Later, the mixture was continuously sonicated for 5 min with the power of 1000 W. Subsequently, the mixed solutions were heated to 110 °C within 3 min and kept at this temperature for 17 min under microwave heating combined with discontinuous ultrasonic irradiation (1 s of sonication and 2 s of interruption) with the power of 500 W. After finishing the synthesis the products were centrifuged, thoroughly rinsed (water, ethanol) and subsequently dried. Li et al. [844] obtained various morphologies of ZnO products, including grenade-like, column-like, spindle-like, rod-like, shuttle-like and flower-like micro-/nanostructures. The size and shape of the ZnO nanostructures was controlled by the authors by changing the Zn/HEPES molar ratio, pH value, and Zn precursor. Figure 70 demonstrates the control of the ZnO morphology by changing the Zn/HEPES molar ratio. When the Zn/HEPES molar ratio was adjusted to 3:8, half-backed grenade-like ZnO microstructures were formed (Figure 70a,b). When the Zn/HEPES molar ratio was adjusted to 1:4, spindle-like ZnO microstructures were formed (Figure 70c,d). When the Zn/HEPES molar ratio was adjusted to 1:8, spindle-like to double-prism-like ZnO structures were formed (Figure 70e,f). When the Zn/HEPES molar ratio was adjusted to 1:20, short spindle-like ZnO nanostructures were formed (Figure 70g,h). When the Zn/HEPES molar ratio was adjusted to 1:40, irregular ZnO agglomerates were formed (Figure 70g,h). Figure 71 presents a change in the ZnO morphology caused by changes in the pH (7.4, 8.4, and 9.4) in a precursor with the constant Zn/HEPES molar ratio of 1:5. Figure 72, in turn, shows a change in the ZnO morphology caused by a change in the pH (7.4, 8.4, and 9.4) in a precursor with the constant Zn/HEPES molar ratio of 1:2.

**Figure 73:**
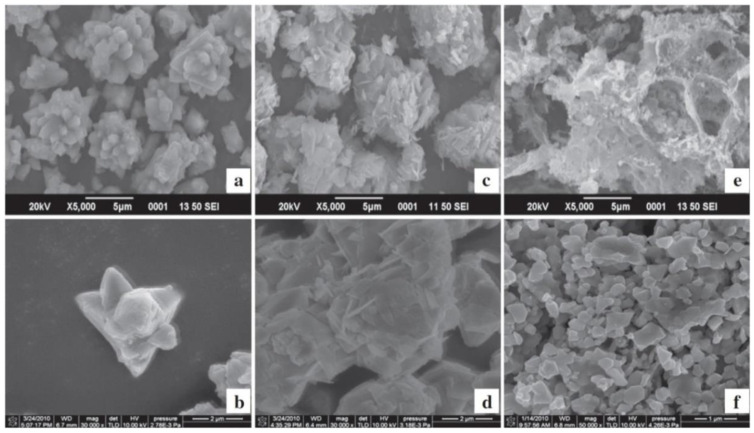
SEM images of ZnO samples with the following molar ratios of urea/Zn^2+^ (**a**,**b**) 1:1, (**c**,**d**) 5:3, and (**e**,**f**) 3:1. Products synthesised by the microwave induced combustion process. Reprinted from [847], Copyright (2010), with permission from Elsevier [OR APPLICABLE SOCIETY COPYRIGHT OWNER]. All rights reserved. In order to re-use permission must be obtained from the rightsholder.

An example of a publication showing results of obtaining ZnO with the use of the microwave induced combustion process is the paper by Cao et al. [847]. In this paper, an aqueous solution of zinc nitrate with an addition of urea was used as a reaction precursor. The samples were placed in an ordinary microwave oven and subjected to microwaves. After boiling, evaporating, and concentrating, the precursor rapidly foamed up, deflagrated, and released heat and gases. Synthesis products were heterogeneous ZnO microstructures, the morphology of which changed depending on the urea/Zn^2+^ molar ratio (1:1, 5:3, and 3:1), which is presented in Figure 73. The effect of the high temperature of the microwave induced combustion process was that the vast majority of the obtained synthesis products were ZnO microstructures.

Hu et al. [864] report obtaining ZnO NPs by sol-gel—microwave assisted annealing. As the reaction precursor, a suspension obtained by mixing a solution of zinc acetate in anhydrous ethanol with a solution of LiOH·H_2_O in anhydrous ethanol was used. Hu et al. [864] performed a range of syntheses to examine the impact of temperature and duration on a change in ZnO NPs properties, namely:

- for the same duration (20 min) at various reaction temperatures (30, 40, 50 and 60 °C),
- for the same reaction temperature (50 °C) with various durations (10, 20, 30 and 40 min).

**Figure 74:**
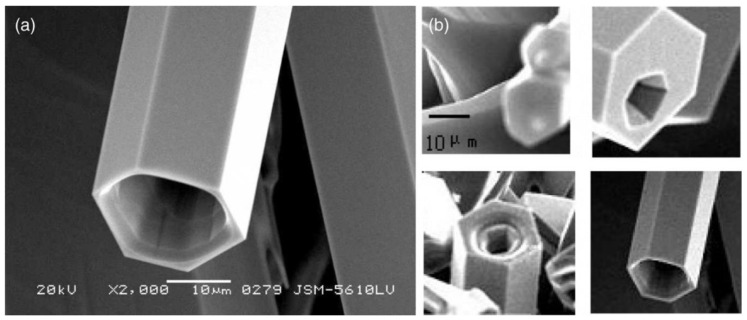
(**a**) SEM image of ZnO tubes growing by the microwave heating method showing well faceted end and side surfaces. (**b**) SEM images of different stages of ZnO tubes by the microwave heating method. Products synthesised by the microwave sintering. Reprinted from [868], Copyright (2005), with permission from Elsevier [OR APPLICABLE SOCIETY COPYRIGHT OWNER]. All rights reserved. In order to re-use permission must be obtained from the rightsholder.

The product in the form of a transparent ZnO sol was removed from the microwave reactor, and subsequently introduced to hexanol and cooled to 4 °C. Thus prepared suspensions were centrifuged and the obtained white powders were dried in the air at the temperature of 80 °C. According to the Scherrer’s formula, the particle size of ZnO prepared at 30 °C for 20 min, 40 °C for 20 min, 50 °C for 10 min, 50 °C for 20 min, 50 °C for 30 min, 60 °C for 20 min, and 50 °C for 40 min are 3.86, 4.09, 4.33, 4.78, 4.96, 5.14, and 6.68 nm, respectively [864]. Zhou et al. [868] report the results of obtaining ZnO hexagonal tubes by the microwave sintering method. For the synthesis, a mixture of nanometric and micrometric ZnO was used, which was obtained by triturating for 1 h in an agate mortar and then pressed into pills with a hollow core, which itself acted as a form of a growth chamber. Thus prepared pastilles were placed in a microwave chamber on a quartz plate surface. The pastilles were subjected to microwave heating at the temperature of 1100–1350 °C for 40 min. The product was tubes (Figure 74) with the diameter exceeding 10 μm, length exceeding 100 μm, while wall thickness ranging from 0.5 to 1 μm. The hexagonal tubes growth process was explained by the authors [868] by proposing a vapour-solid mechanism.

**Figure 75:**
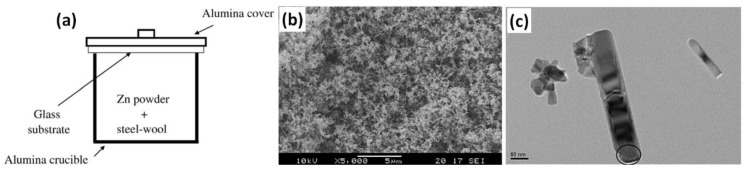
(**a**) The schematic of the sample environment. (**b**) Typical surface-section SEM micrograph of obtained ZnO powder. (**c**) TEM image of the obtained ZnO nano-fibre. Products synthesised by the microwave vapour deposition. Reprinted from [871], Copyright (2007), with permission from Elsevier [OR APPLICABLE SOCIETY COPYRIGHT OWNER]. All rights reserved. In order to re-use permission must be obtained from the rightsholder.

Takahashi [871] reports obtaining ZnO nano-fibres by means of a domestic microwave oven. As the reaction precursor, he used a mixture of Zn powder and steel-wool, which was introduced to an Al_2_O_3_ crucible. The crucible was covered by a glass substrate, on which the crucible cover was placed (Figure 75a). The sample was subjected to microwave heating with the power of 1000 W for 30 s. As a result of microwave heating, ZnO nano-fibres (Figure 75b) with the diameter of ca. 50 nm and the length of 0.5–1 μm (Figure 75c) grew on the glass substrate.

Details of other research papers concerning the pure ZnO obtained by the microwave hybrid method are presented in Table 13.

### 5.4. Types of ZnO Nanocomposites or ZnO Hybrid Nanostructures Obtained by the Microwave Hybrid Synthesis Method

The microwave hybrid synthesis enables obtaining:

- ZnO doped with the following ions: Al^3+^ [890], Ba^2+^ [893], Co^2+^ [895,896], Cu^2+^ [904,914], Cr^3+^ [897], Eu^3+^ [907,908], Ga^3+^ [910,911], Mg^2+^ [915] and Sm^3+^ [919];
- ZnO co-doped with the following ions: Ce^2+^-Cu^2+^ [894] and Mn^2+^-Co^2+^ [917], where M = Mn^2+^, Ni^2+^, Fe^3+^ and Cu^2+^ [914];
- composite and hybrid materials: Ag-ZnO [887,888,889], Au-ZnO [887], Ag–ZnO–graphene [889], Al^3+^ doped ZnO/Sn doped In_2_O_3_ [891], Au/Fe_2_O_3_–ZnO [892], ZnO/BiOBr [898], Zn–ZnO [899,900], ZnO–ZrO_2_ [901], ZnAl_2_O_4_/ZnO [902,903], Cu–ZnO–Al_2_O_3_ [905], Cu–ZnO [906], Fe_2_O_3_/ZnO [909], In_2_O_3_–Ga_2_O_3_–ZnO [912,913], MgO–ZnO [916], Sb_2_O_3_–MnO–CoO–Cr_2_O_3_–ZnO [918], Ti*_x_*O*_y_*–ZnO [920], ZnO/ZnFe_2_O_4_ [921], ZnO/multi-walled carbon nanotube [922], ZnO—exfoliated graphene [923], ZnO–expandable graphite [927], and ZnO–reduced graphene oxide [924,925].

### 5.5. Synthesis of ZnO Nanocomposites or ZnO Hybrid Nanostructures by the Microwave Hybrid Method

Dou et al. [889] report obtaining ZnO NPs, Ag/ZnO nanocomposites and Ag/ZnO/graphene nanocomposites by the microwave and ultrasonic wave combined method. In order to obtain products, the aqueous mixture of:

- zinc nitrate with hexamethylenetetramine was used to obtain ZnO NPs;
- zinc nitrate, silver nitrate with hexamethylenetetramine was used to obtain Ag/ZnO nanocomposites;
- zinc nitrate, silver nitrate with hexamethylenetetramine and an addition of graphene was used to obtain Ag/ZnO/graphene nanocomposites.

**Figure 76:**
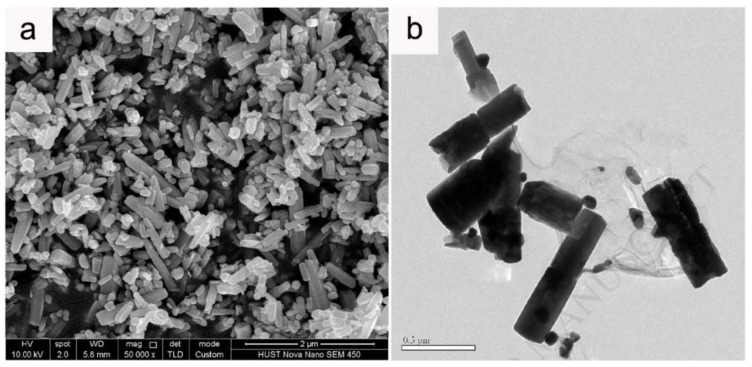
Images of the Ag/ZnO/graphene nanocomposites: (**a**) SEM; (**b**) TEM. Products synthesised by the microwave and ultrasonic wave combined method. Reprinted from [889], Copyright (2015), with permission from Elsevier [OR APPLICABLE SOCIETY COPYRIGHT OWNER]. All rights reserved. In order to re-use permission must be obtained from the rightsholder.

Identical synthesis parameters were applied for each sample, namely after stirring the suspensions for 10 min, the reaction vessel with the sample was put into an ultrasonic microwave reaction system and kept at 90 °C for 2 h and sonicated for 30 min. Subsequently, the samples were centrifuged, rinsed with water and then dried in a vacuum dryer. Dou et al. [889] provide only the results of the morphology of Ag/ZnO/graphene nanocomposites (Figure 76).

Mary et al. [894] report obtaining ZnO co-doped with Ce^2+^ and Cu^2+^ ions by the microwave induced combustion process. Reaction precursors were obtained by dissolving zinc nitrate, cerium nitrate, copper nitrate in deionised water with an addition of urea as a fuel. The crucible with a precursor was placed in a microwave oven, where the precursor solution underwent various reactions for 8 min of heating. Namely, the solution first boiled, then underwent dehydration, and subsequently decomposition with emission of large quantities of vapours in the form of smoke. When the solution reached the spontaneous ignition point, the combustion heat released and transformed the sample into a solid powder. ZnO powders were prepared with the addition of copper (Cu) and cerium (Ce) of different molar ratios (Zn_1−2*x*_Ce*_x_*Cu*_x_*)O with *x* = 0.00, 0.01, 0.02, 0.03, 0.04, and 0.05 to ZnO. The general equation of the synthesis reaction is described by Equation (57).

(57) $$\mathrm{Zn}{\left({\mathrm{NO}}_{3}\right)}_{2}+\mathrm{Cu}{\left({\mathrm{NO}}_{3}\right)}_{2}+\mathrm{Ce}{\left({\mathrm{NO}}_{3}\right)}_{2}\overset{\mathrm{urea},\;\mathrm{water}\;\mathrm{MH}\;\left(T\right)}{\to }{\mathrm{Zn}}_{1-2x}{\mathrm{Ce}}_{x}{\mathrm{Cu}}_{x}O+\mathrm{gaseous}\;\mathrm{products}$$

**Figure 77:**
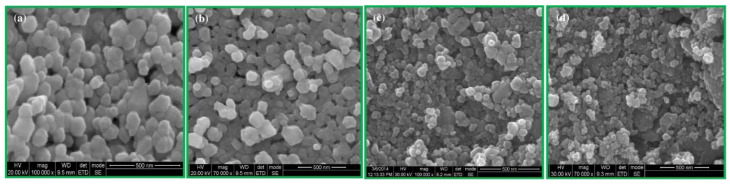
SEM images of Zn_1−2*x*_Ce*_x_*Cu*_x_*O: (**a**) Pure ZnO, (**b**) Zn_0.98_Ce_0.01_Cu_0.01_O, (**c**) Zn_0.94_Ce_0.03_Cu_0.03_O and (**d**) Zn_0.90_Ce_0.05_Cu_0.05_O. Products synthesised by the microwave induced combustion process. Reprinted from [894], Copyright (2015), with permission from Elsevier [OR APPLICABLE SOCIETY COPYRIGHT OWNER]. All rights reserved. In order to re-use permission must be obtained from the rightsholder.

The size of Zn_1−2*x*_Ce*_x_*Cu*_x_*O crystallites was 35 nm, 38 nm, 40 nm, 44 nm, 46 nm and 46 nm depending on the dopant content of 0.00, 0.01, 0.02, 0.03, 0.04 and 0.05, respectively. The SEM results (Figure 77) indicated that the size of Zn_1−2*x*_Ce*_x_*Cu*_x_*O particles decreased in line with the increase in the dopant content. The structure of the obtained pure ZnO was polycrystalline, where the ZnO particle size reached several hundred nm, while for the Zn_0.90_e_0.05_Cu_0.05_O sample the largest particle size reached up to several dozen nm.

**Figure 78:**
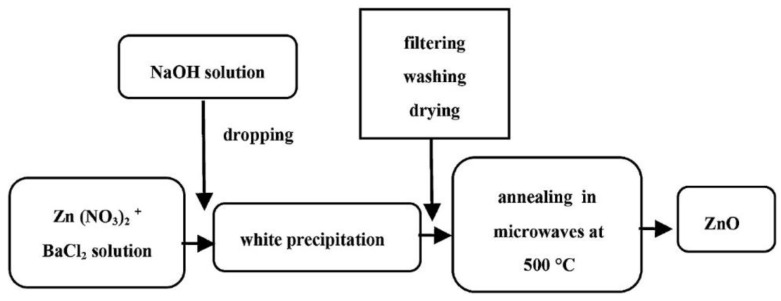
Schematic of the experimental procedure. Products synthesised by the microwave assisted annealing process. Reprinted from [893], Copyright (2015), with permission from John Wiley & Sons Inc [the Wiley Companies, or their respective licensors]. All rights reserved. In order to re-use permission must be obtained from the rightsholder.

**Figure 79:**
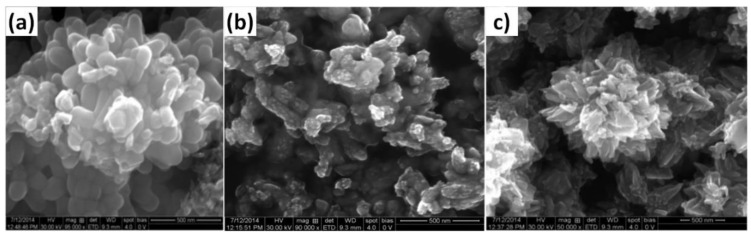
SEM images of undoped ZnO and Ba doped ZnO nanoparticles. (**a**) ZnO, (**b**) Ba (1 at%) doped ZnO and (**c**) Ba (2 at%) doped ZnO. Products synthesised by the microwave assisted annealing process. Reprinted from [893], Copyright (2015), with permission from John Wiley & Sons Inc [the Wiley Companies, or their respective licensors]. All rights reserved. In order to re-use permission must be obtained from the rightsholder.

Konou et al. [893] described Ba doped ZnO by the microwave assisted annealing process. Figure 78 shows a scheme according to which samples were obtained by Konou et al. [893]. First, NaOH (a molar ratio of OH^−^ and Zn^2+^ kept to 3) was dropped into solutions of zinc nitrate and barium chloride, and subsequently the obtained suspension was filtered, rinsed and dried. The obtained powders were soaked in a microwave furnace at the temperature of 500 °C for 30 min. The barium doping concentration of ZnO was 0, 1, and 2 at%. Figure 79 presents the morphology of ZnO, Ba (1 at%) doped ZnO and Ba (2 at%) doped ZnO. The obtained products were characterised by a compact (sintered) structure, which resulted from the application of a high soaking temperature (500 °C). All three samples were characterised by a heterogeneous shape and by a large range of particle/aggregate sizes although the crystallite size was the same in all samples (20–22 nm).

**Figure 80:**
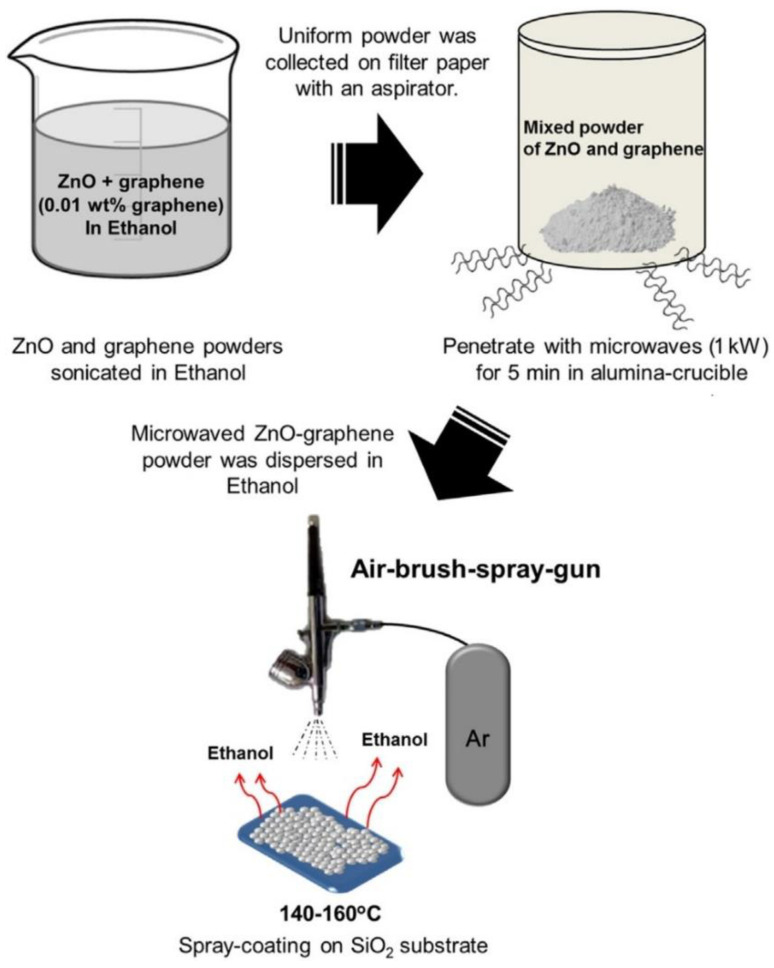
Schematic illustration of different steps of the microwave sintering synthesis of ZnO NPs/graphene nanocomposites. Products synthesised by the microwave sintering process. Reprinted from [927], Copyright (2017), with permission from Elsevier [OR APPLICABLE SOCIETY COPYRIGHT OWNER]. All rights reserved. In order to re-use permission must be obtained from the rightsholder.

**Figure 81:**
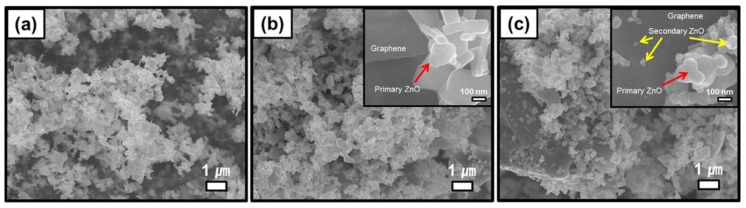
SEM images of (**a**) pristine ZnO NPs, (**b**) ZnO NPs/graphene nanocomposites without the microwave sintering process and (**c**) ZnO NPs/graphene nanocomposites after the microwave sintering process. Products synthesised by the microwave sintering process. Reprinted from [927], Copyright (2017), with permission from Elsevier [OR APPLICABLE SOCIETY COPYRIGHT OWNER]. All rights reserved. In order to re-use permission must be obtained from the rightsholder.

**Figure 82:**
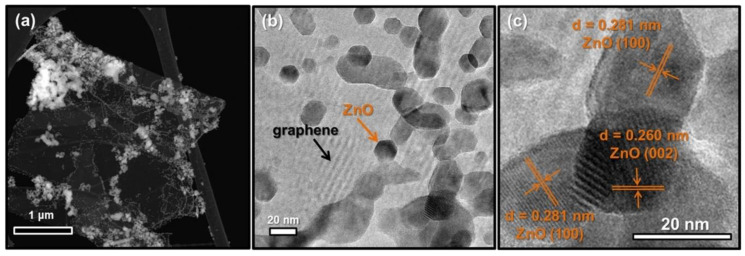
TEM images of (**a**–**c**) ZnO NPs/graphene nanocomposites. Products synthesised by the microwave sintering process. Reprinted from [927], Copyright (2017), with permission from Elsevier [OR APPLICABLE SOCIETY COPYRIGHT OWNER]. All rights reserved. In order to re-use permission must be obtained from the rightsholder.

Kim et al. [927] described a microwave sintering synthesis of ZnO NPs/graphene nanocomposites, where individual steps of obtaining ZnO NPs/graphene nanocomposites are presented in Figure 80. Commercially available ZnO NPs and expandable graphite were used for the synthesis. The expandable graphite sample was introduced to a crucible (Al_2_O_3_) and subjected to microwave heating (1 kW, 1 min), after which the product was dispersed in ethanol and sonicated for 10 min to exfoliate the graphite. Subsequently, ZnO NPs together with exfoliated graphite (0.01 wt%) were mixed in ethanol through sonication. The mixture was filtered off on a filter paper and subsequently the powders were placed in a crucible (Al_2_O_3_) and heated in a microwave oven (1 kW, 5 min). Thus obtained ZnO-graphene nanocomposite powders were dispersed in ethanol and were spray-coated on a heated SiO_2_ substrate using a spray gun (140–160 °C). Figure 81a presents the morphology of commercial ZnO NPs, while Figure 81b,c show SEM images of ZnO/graphene nanocomposites without the microwave sintering process and after the microwave sintering process, respectively. The main difference between the morphology of samples in Figure 81b,c was the emergence of secondary ZnO NPs with smaller sizes in the sample after the microwave sintering process, which is perfectly visible in TEM images (Figure 82). The results achieved by Kim et al. [927] are very interesting, because generally the sintering process of NPs leads to their size growth, but in this particular case, a secondary ZnO NPs phase with smaller sizes was obtained.

**Figure 83:**
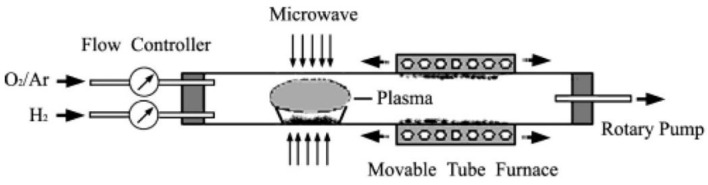
Schematic illustration of the microwave plasma system for Zn-ZnO nanocables and ZnO nanotubes growth. Reprinted (adapted) with permission from [899]. Copyright © 2003, American Chemical Society. All rights reserved. In order to re-use permission must be obtained from the rightsholder.

Zhang et al. report [899] obtaining Zn-ZnO nanocables and ZnO nanotubes by microwave vapour deposition with the use of a microwave plasma system. The scheme of their experiment is shown in Figure 83. Zinc powder present in the quartz crucible was placed inside a horizontal quartz tube. Pure hydrogen (H_2_) was used as the protective gas and the carrier gas, which was introduced to the reaction chamber with the flow rate of 50–90 sccm. The pressure in the reaction chamber was maintained at ca. 4 Torr. Microwaves (400 W) were introduced with the use of a wave guide in the central part of the quartz tube for generating stable plasma. The temperature of the microwave plasma was estimated at about 1000 °C. As an additional heat source, a movable tubular furnace was used, which generated the temperature of 700 °C at a stretch of 20 cm, while the estimated temperature in the quartz tube along the same stretch was ca. 500 °C. After switching the heating off for 30 min, the H_2_ flow was stopped and a black product covering the internal surface of the quartz tube was observed. For the purpose of oxidising the formed product, O_2_/Ar gas (volume ratio 1:50) was introduced to the microwave plasma system for 15–30 min. Then, the tubular furnace was moved back-and-forth along the quartz tube until only white powder was left. The morphology of the obtained products could be controlled through the oxidation duration (15–30 min) and the H_2_ flow rate (50–90 sccm). Figure 65f,g shows the morphology of the final products. The nanotubes had a uniform inner and outer diameter of about 10 and 40 nm, respectively, while the average length of the nanotubes was ca. 1 µm. Figure 65f,g reveal that the outer diameter of ZnO nanotubes is virtually identical to the outer diameter of Zn–ZnO nanocables.

Details of other research papers concerning the of ZnO nanocomposites or ZnO hybrid nanostructures obtained by the microwave hybrid method are presented in Table 14.

## 6. Conclusions

ZnO is a multifunctional material, among others thanks to its semi-conductor, optical, biological, antibacterial, piezoelectric properties. Probably, to the surprise of many, the popularity of ZnO contyinues to grow, which is mainly caused by the development of new methods of obtaining ZnO NMs. These methods permit a synthesis of various ZnO NMs, e.g., owing to the possibility to control the shape and size, and to modify the chemical composition (dopants, composites etc.), which defines their properties and enables their use in new applications. ZnO NMs are also promising unique components for producing various innovative devices. However, for the potential of all ZnO NMs to be fully tapped in consumer products, numerous comprehensive tests are still needed. Nowadays, the microwave heating technology is already a classic method for fabrication of distinct chemical compounds and materials, including ZnO NMs. This has become possible thanks to the availability of new professional microwave apparatus on the market, which is continually improved by the manufacturers to meet the users’ requirements. A microwave apparatus can be encountered increasingly often in laboratories, the industry, and virtually all households. Microwave heating is environment-friendly and has been classified as a green chemistry approach. The main advantages of microwave heating include speed, homogeneity, and purity (contactless method).

This comprehensive review describes the microwave synthesis of ZnO NMs (pure, doped, composites). We concentrated in particular on reactants, process parameters and morphologies of the obtained products. The microwave synthesis of ZnO NMs was divided into three primary groups: hydrothermal, solvothermal and hybrid methods. Hybrid methods include such methods as: ultrasonic microwave synthesis, microwave assisted combustion synthesis, microwave assisted annealing, microwave assisted sintering, and microwave vapour deposition. Statistics of the use of reactants were presented. The overall results point that the microwave synthesis of ZnO NMs has an enormous potential that enables obtaining a wide range of product morphologies, beginning with quantum dots, to core-shell structures and hierarchical structures, and ending with films on substrates (surface modification). The discovery and explanation of some of the mechanisms of microwave syntheses have made it possible to obtain ZnO NMs with controlled properties (among others size, shape) and has eliminated the unrepeatability of syntheses. The present review indicates that the microwave synthesis of ZnO NMs is an extremely vast research topic, with many phenomena yet to be explained. Moreover, new issues (effects, mechanisms) related to the microwave synthesis of ZnO NMs certainly remain to be discovered.

Unfortunately, the cited literature does not provide examples of the achieved daily capacity of the microwave synthesis of ZnO NMs. Nevertheless, based on our many years of experience with the microwave synthesis of nanomaterials, we have become convinced in practice that, thanks to microwave solvothermal synthesis, ca. 100 g of ZnO NPs can be achieved daily.

## Figures and Tables

**Table 1:** Physical properties of ZnO with the wurtzite structure. Data were derived from literature [98,99,100,101,102,103,104,105,106,107].

Properties	Value
Molecular formula	ZnO
State (colour, form)	white powder
CAS Reg No.	314-13-2
Molar mass	81.39 g/mol
Density at room temperature	5.606 g/cm^3^ (crystal theoretical density 5.61 g/cm^3^)
Solubility in water (25 °C)	1.6 mg/L
Melting point	1975 °C
Boiling point	2360 °C
Stable phase at room temperature	wurtzite
Structure	Hexagonal, where *a*_0_ = *b*_0_ ≠ *c*_0_
Space group symmetries	C4 6v (P63mc)
Bulk effective piezoelectric constant	9.9 pm/V
Hardness	5.0 ± 0.1 GPa
Lattice parameters at 300 K	
*a*_0_	3.2495 Å
*c*_0_	5.2069 Å
*c*_0_/*a*_0_	1.602 (ideal hexagonal structure shows 1.633)
U	0.345
Thermal conductivity	0.6, 1–1.2 W·cm^−1^·K^−1^
Specific heat	0.125 cal/gm·°C
Linear expansion coefficient	*a*_0_: 6.5 cm 3 × 10^−6^ K
	*c*_0_: 3.0 cm 3 × 10^−6^ K
Static dielectric constant	8.656 ε(0), ε(∞)
Thermoelectric constant at 573 K	1200 mV/K
Refractive index	2.008–2.029
Band gap	at RT: 3.370 eV
	at 4 K: 3.437 eV
Exciton binding energy	60 meV
Intrinsic carrier concentration	<10^6^ cm^3^
Electron effective mass	0.24 m_0_
Hole effective mass	0.59 m_0_
Electron Hall mobility at 300 K	200 cm^2^/V·s
Hole Hall mobility at 300 K	5–50 cm^2^/V·s
Ionicity	62%

**Table 2:** tanδ values of different solvents at 2.45 GHz and 20 °C [428,458,459].

High (>0.5)		Medium (0.1–0.5)		Low (<1)	
Solvent	tanδ	Solvent	tanδ	Solvent	tanδ
Ethylene glycol	1.350	2-Butanol	0.447	Chloroform	0.091
Ethanol	0.941	Dichlorobenzene	0.280	Acetonitrile	0.062
DMSO	0.825	NMP	0.275	Ethyl acetate	0.059
2-Propanol	0.799	Acetic acid	0.174	Acetone	0.054
Formic acid	0.722	DMF	0.161	THF	0.047
Methanol	0.659	Dichloroethane	0.127	Dichloromethane	0.042
Nitrobenzene	0.589	Water	0.123	Toluene	0.040
1-Butanol	0.571	Chlorobenzene	0.101	Hexane	0.020

**Table 3:** Penetration depths of the 2.45 GHz microwaves for common solvents and materials.

Material	Temperature (°C)	Penetration Depth (cm)	Ref.
Water (distilled)	20	1.6	[463]
Water (distilled)	25	2.88	[456]
Water (distilled)	100	80	[463]
0.125 M NaCl solution of salt water	25	0.88	[464]
0.5 M NaCl solution of salt water	25	0.45	[456]
2 M NaCl solution of salt water	25	0.14	[464]
Water (ice)	−12	1100	[465]
Ethylene glycol	25	0.46	[464]
Methanol	25	0.68	[464]
Ethanol	25	0.93	[464]
1-propanol	25	1.39	[464]
Acetone	25	7.07	[464]
Ethyl acetate	25	11.05	[464]
Xylene	25	28.32	[464]
Rubber, styrene-butadiene (SBR), vulc.	-	19	[463]
Nitrile rubber, natural	-	65	[463]
Aluminium oxide (Al_2_O_3_) ceram, for MW use	-	3000	[463]
Polyethylene	25	4000	[463]
Polyethylene	-	5907.1	[456]
Polystyrene	-	7619.3	[456]
PTFE (Teflon®)	-	9000	[463]
Quartz, pure	-	20,000	[463]
Silver	-	0.33 × 10^−4^	[463]
Zinc, pure (Zn)	-	1.24·× 10^−4^	[463]
Copper (Cu)	-	1.3 × 10^−4^	[456]
Aluminium 100% (Al)	-	0.86 × 10^−4^	[463]
Aluminium (Al)	-	1.7 × 10^−4^	[456]
Nickel (Ni)	-	2.7 × 10^−4^	[456]
Iron (Fe)	-	3.2 × 10^−4^	[456]
Titanium, pure (Ti)	-	3.3 × 10^-4^	[463]
Stainless steel (304)	-	4.3 × 10^−4^	[463]

**Table 4:** Summary of durations of microwave heating of a 70 mL distilled water sample. Source: Experimental data achieved in the MSS3 reactor (IHPP PAS).

Microwave Power	Heating Time after Which the Temperature Was Reached (s)	
	100 °C	140 °C
100 W	443	-
200 W	139	353
300 W	106	171
400 W	61	112
500 W	43	141
600 W	37	61
700 W	31	52
800 W	27	45
900 W	22	41
1000 W	22	39

**Table 5:** Summary of microwave hydrothermal synthesis of ZnO without any additional heat treatment. SSA: specific surface area.

Substrates	Conditions during Preparation	Properties	Ref.
Zn(NO_3_)_2_·6H_2_O (0.1, 0.5 and 2 M), NaOH, H_2_O	pH: 8–12; *T*: 100–190 °C; *P*: 1–13 bar, duration: 2 min–2 h; microwave reactor	SSA: 4.7–18.1 m^2^/g; particles, submicrometre grains and star-like morphology	[506]
Zn(NO_3_)_2_·6H_2_O (0.1 M), NaOH (2 M), H_2_O	*P*: 9–39 bar, duration: 3–7 min; power: 70–100%; microwave reactor (750 W)	heterogeneous nano- and microstructures; particles size: 10–300 nm	[507]
ZnCl_2_·2H_2_O (0.1 M), KOH or urea, H_2_O	pH: 12; *P*: 10–40 bar; duration: 3–15 min; microwave reactor	SSA: 8.6–102.2 m^2^/g; particles size: 37–114 nm, flower-like morphology	[508]
Zn(NO_3_)_2_·6H_2_O (0.005 M), hexamethylenetetramine (C_6_H_12_N_4_) (0.005 M), H_2_O	*T*: 90 °C; duration: 2 min; microwave reactor	ZnO rods (e.g., bipods, tripods, tetrapods and multipods); diameter: 160–220 nm; length: 1.25–1.3 µm	[509]
Zn(NO_3_)_2_·6H_2_O (0.13 M), NaOH (1.3 M), 1-n-butyl-3-methyl imidazolium tetrafluoroborate, H_2_O	*T*: 90–125 °C; duration: 2–10 min; microwave reactor	morphology: flower-like + needle-like, from 60 to 450 nm and lengths up to several micrometres	[510]
Zn(CH_3_COO)_2_·2H_2_O, N,N dimethylformamide, H_2_O	duration: 23 min; power: 50%; microwave oven	spherical particles ~160 nm and nanoplatelets and nanorods ~2 nm in diameter and ~80 nm in length	[511]
ZnO was dissolved in NH_4_(OH) and Zn^2+^ 0.08 M, H_2_O, NH_4_(OH) (0.5, 1, 5, 10 and 14.8 M) was obtained	*T*: 90–150 °C; *P*: 0.7–4.8 bar; power: 1000 W; microwave reactor	flower-like agglomeration	[512]
Zn(NO_3_)_2_·6H_2_O (0.005 M), hexamethylenetetramine (C_6_H_12_N_4_) (0.010 M), NaOH (3 M)	pH: 9 and 13; *T*: 96 °C; duration: 60 min;	flower-like ZnO microstructures (2–3 µm) of hexagonal prisms (length: 1–2 μm, diameter: 50–130 nm) with planar and hexagonal pyramid tips (length: 1.5 μm, diameter: 300 nm)	[513]
Zn(NO_3_)_2_·6H_2_O (0.43 M), NaOH (0.43 M), H_2_O, NaCl, wet mechanical mixtures obtained	*T*: 75–135 °C; power: 650 W; microwave oven	SSA: 9–13 m^2^/g; microtubes	[514]
Zn(NO_3_)_2_·6H_2_O (0.05 M), urea (0.05 M), H_2_O	duration: 40 min; power: 180 W; microwave oven	nanotubes have regular polyhedral shapes, hollow cores with diameters of 100–200 nm, lengths of 1–3 mm and wall thicknesses of 10–40 nm.	[515]
Zn(NO_3_)_2_·6H_2_O (0.43 M), NaOH (0.43 M), H_2_O	duration: 15 min; *T*: 75–170 °C; power: 40–450 W; Teflon cell in microwave oven; pulsed mode	SSA: 9–19 m^2^/g, crystallite size: 30–45 nm	[516]
Zn(CH_3_COO)_2_·2H_2_O and hydrazine (N_2_H_4_·H_2_O) in a molar ratio of 1:4 in H_2_O	duration: 10 min; power: 150 W; microwave oven	nanorods; diameter about 25–75 nm and length in the range of 500–1500 nm	[517]
Zn(NO_3_)_2_·6H_2_O, NaOH, H_2_O	duration: 20 min; *T*: 100–180 °C; power: 0–1000 W; microwave reactor	nanorods; nanowires; nanothruster vanes; nanodandelions; nanospindles	[518]
Zn(NO_3_)_2_·6H_2_O (1.6 M), NaOH (3.2 M), H_2_O	pH: 8.3; duration: 1–5 min; microwave oven	nanorods (diameter: 100–200 nm) and flower structures	[519]
Zn(NO_3_)_2_·6H_2_O, NaOH (different concentrations), H_2_O	duration: 1 h; *T*: 110 °C; microwave oven	submicron starshaped structures, chrysanthemum flower structures, nanoflakes	[520]
Zn(NO_3_)_2_·6H_2_O, pyridine (C_5_H_5_N), aniline (C_6_H_5_NH_2_) and triethanolamine (TEA, C_6_H_15_NO_3_) (different concentrations), H_2_O	duration: 10 min; *T*: 90 °C; microwave reactor	various morphologies: linear linked needles, regularly hexagonal cross section of a needle, hollow structures, hexagonal nanorings, hexagonal columns, nanosheets	[521]
Zn(CH_3_COO)_2_·2H_2_O, NaOH, 1-ethyl-3- methylimidazolium bis(trifluoromethylsulfonyl)imid, H_2_O	duration: 2–7 min; *T*: power: 255 W; microwave oven (850 W)	nanoparticles, length less than 100 nm	[522]
Zn(NO_3_)_2_·6H_2_O (0.025 M); hexamethylenetetramine (C_6_H_12_N_4_) (0.025 M), H_2_O	duration: 1–30 min; *T*: 100–180 °C; power: 120–700 W; microwave oven	nanowires	[523]
Zn(NO_3_)_2_·6H_2_O, NaOH (5 M) nasturtium officinale leaf extract, H_2_O	pH: 10; duration: 10 min; microwave oven (1000 W)	heterogeneous aggregates of NPs	[524]
Zn(NO_3_)_2_·6H_2_O (0.025 M), Zn(CH_3_COO)_2_·2H_2_O, NH_4_(OH) (0.16 M), H_2_O, ethylenediamine (EDA, C_2_H_8_N_2_), hexamethylenetetramine (C_6_H_12_N_4_), triethyl citrate (C_12_H_20_O_7_), tripotassium citrate monohydrate (C₆H₅K₃O₇·H₂O)	duration: 15 min; *T*: 90 °C; microwave reactor	nanorods, nanoneedles, nanocandles, nanodisks and nanonuts	[525]
Zn(NO_3_)_2_·6H_2_O, hexamethylenetetramine (HMTA), polyvinylpyrrolidone (PVP), H_2_O	duration: 60 min; *T*: 100 °C; power: 300 W; microwave reactor	nanostars, average size: ≈625 nm, crystallite size ≈550 nm, SSA = 20.6 m^2^/g	[526]
Zn(CH_3_COO)_2_·2H_2_O (different concentrations), NH_4_(OH), H_2_O	duration: 85 s; *T*: 90 °C; power: 800 W; microwave oven	flower-like shapes with diameter of 3 to 5 µm, flowers with rod-like nanostructures, spherical particles in 2–4 µm diameter; 2–3 µm structured balls with occasional large 10 µm lumps	[527]
Zn(CH_3_COO)_2_·2H_2_O, NH_4_(OH) (different concentrations), H_2_O	pH: 7.0–11.1; duration: 2 h; *T*: 150 °C; microwave reactor	hexagonally shaped prismatic (width ≈ 1 μm, length ≈ 5 μm); flower-like structures formed by a micron sized crystals; heterogeneous particles (size from ~50 nm to 300 nm)	[528]
Zn(CH_3_COO)_2_·2H_2_O, NaOH, (o- and m- and p)-nitrobenzoic acid, H_2_O	duration: 10 min; microwave reactor	flower-like products consist of sword-like ZnO nanorods, which were 60–100 nm in width and several micrometres in length.	[529]
ZnCl_2_·2H_2_O, NaOH, H_2_O, bis(dodecyldimethyl ammonium bromide) (C_26_H_56_BrN)	duration: 5–10 h; *T*: 100–140 °C; power: 300–400 W; microwave reactor	flower-like with mean r 0.5–1.5 µm, sphere-like with mean diameter of 0.5 µm	[530]
ZnCl_2_·2H_2_O, NaOH, H_2_O, cetyltrimethylammonium bromide (CTAB, C_19_H_42_BrN), Pluronic F127	duration: 5 min; power: 130 W; microwave oven	SSA: 15.5–24.8 m^2^/g, diameter: 58–93 nm, heterogeneous shape	[531]
Zn(CH_3_COO)_2_·2H_2_O, Na(OH) (different concentrations), H_2_O	duration: 5 min; power: 450 W; microwave oven	nanoplates flowers	[532]
Zn(CH_3_COO)_2_·2H_2_O, NaOH, H_2_O, polyethylene glycol, ethanol	duration: 30 min; *T*: 140 °C; power: 700 W; microwave reactor (multimode)	nanorods, flowers	[533]
Zn(CH_3_COO)_2_·2H_2_O (0.5 M), KOH (2 M), H_2_O	pH: 8; *T*: 120 and 140 °C; duration: 8 min; microwave oven (800 W)	multiwires with a flower-like shape of 50–400 nm in width and length	[534]
Zn(CH_3_COO)_2_·2H_2_O (0.1 M), Zn(NO_3_)_2_·6H_2_O (0.1 M), NH_4_OH, polyvinilpirrolidone, hydrazine hydrate solution (N_2_H_4_ in H_2_O), H_2_O	pH: 7.5–8; duration: 5–10 min; microwave oven (1000 W)	spherical nanoparticles, stars, flowers	[535]
Zn(NO_3_)_2_·6H_2_O, NaOH, gum arabic (stabilising agent), NaOH, H_2_O	pH: 10; duration: 5 min; power: 450 W	stars (diameter: 1020 nm), spherical particles (diameter: 240 nm)	[536]
Zn(NO_3_)_2_·6H_2_O, NaOH, gum arabic (stabilising agent), NaOH, H_2_O	pH: 10; duration: 2–10 min; power: 350 W	aggregates of NPs (20–40 nm), size of aggregates: 150–200 nm	[537]
Zn(NO_3_)_2_·6H_2_O (0.1 M), NH_4_(OH), hydrazine hydrate (N_2_H_4_·H_2_O), H_2_O	pH: 8; duration: 10 min; microwave oven	flower morphology consists of sharp nanorods which look like petals	[538]
Zn(NO_3_)_2_·6H_2_O (0.005 M), KOH (4 M), H_2_O	pH: 12; *T*: 120 °C; duration: 4 h; microwave oven	nanowires with diameter of 80 nm and lengths of up to 10 µm.	[539]
Zn(NO_3_)_2_·6H_2_O (0.01 M), urea (0.1 M), H_2_O	*T*: 120 °C; duration: 10–24 min; power: 150 W; microwave oven	javelins, length: 14–17 µm, width: 0.9–1.4 µm	[540]
Zn(CH_3_COO)_2_·2H_2_O, KOH	duration: 20 min; power: 180 W; microwave oven	flowerlike structures composed of hexagonal ZnO spear-shaped nanorods with diameters and lengths of 50 nm and 2–4 μm,	[541]
ZnCl_2_·2H_2_O (different concentrations), NH_4_(OH), H_2_O	duration: 10–40 min; power: 10–50%; microwave oven (800 W)	nanorods	[542]
ZnCl_2_·2H_2_O, arginine (C_6_H_14_N_4_O_2_), H_2_O	*T*: 120–180 °C; duration: 3–10 min; microwave reactor	rods and flowers	[543]
Zn(CH_3_COO)_2_·2H_2_O (different concentrations), NaOH, H_2_O	pH: 12; *T*: 120–140 °C; duration: 15–60 min; power: 0–100%; microwave oven (900 W)	nanosheets	[544]
Zn(CH_3_COO)_2_·2H_2_O, NH_4_(OH), H_2_O	pH: 9; duration: 90 sec; microwave oven (900 W)	narcissus-like nanostructures with crystallite sizes of 10–15 nm and average diameter of 1–2.5 µm	[545]
Zn(CH_3_COO)_2_·2H_2_O, NaHCO_3_, H_2_O	duration: 15 min; power: 200 W; microwave oven	amorphous material	[546]
Zn(CH_3_COO)_2_·2H_2_O (0.0026 M), NaOH (1 M), H_2_O, cetyltrimethylammonium bromide (CTAB, C_19_H_42_BrN)	*T*: 130 °C; duration: 30–180 min; Teflon autoclave in microwave oven (800 W)	nanowires and microwires of about 50–400 nm in width and several micrometres in length	[547]
Zn(NO_3_)_2_·6H_2_O (different concentrations), hexamethylenetetramine (C_6_H_12_N_4_), H_2_O	duration: 2–3 min; power: 600–900 W; Teflon bottle in microwave oven (1000 W)	nanorods diameters from 117 to 156 nm	[548,549]
Zn(NO_3_)_2_·6H_2_O (different concentrations), NaOH, H_2_O	pH: 7–13.1; duration: 20; power: 180 W; microwave oven	nanoparticles in clusters, nanoplates in flower-like clusters, and spear-shaped particles in flower-like clusters	[550]
ZnCl_2_·2H_2_O (0.066 M), NaOH (1.75 M), H_2_O	pH: 13.75; duration: 5 min; power: 150–1000 W; microwave oven (1000 W)	nanoparticles, nanoneedles, nanosheets (leaf-like)	[551]
Zn(CH_3_COO)_2_·2H_2_O (0.1 M), NaOH (4 M), CH_3_(CH_2_)_11_OSO_3_Na (0.1 M), C_12_H_25_C_6_H_4_SO_3_Na (0.025 M), H_2_O	*T*: 75–130 °C; duration: 1–5 h; power: 400 and 700 W; microwave reactor	SSA: 33.1–419.7 m^2^/g; square shaped sheets	[552]
Zn(NO_3_)_2_·6H_2_O, hexamethylenetetramine (C_6_H_12_N_4_)	duration: 30–45 sec; power: 700 W, microwave oven	multiple linked rods such as bipods, tripods (T- shaped), tetrapods (+ and X-shaped), tassel brush and flower shaped and individual rods	[553]
Zn(CH_3_COO)_2_·2H_2_O (different concentrations), triethanolamine (TEA, C_6_H_15_NO_3_), H_2_O	*T*: 80–100 °C; duration: 10–30 min; microwave reactor (1000 W)	pompon-like spheres, peach nut-like spheres, misshapen spheres	[554]
Zn(CH_3_COO)_2_·2H_2_O, NaOH, bis(triaminomethyl) carbonate (C_3_H_12_N_6_O_3_), H_2_O	pH: 12; duration: 2 min; power: 600 W; microwave oven	flower-like structure (2 µm) composed of petals with average size of about 600–700 nm in length, 300–400 nm in width, and 50–70 nm in tip	[555]
Zn(NO_3_)_2_·6H_2_O, NaOH, H_2_O	duration: 15–50 min; power: 120–420 W; microwave reactor (700 W)	nanostructures consisted of flower-like, sword-like, needle-like and rods-like structures	[556]
Zn(CH_3_COO)_2_·2H_2_O (different concentrations), NH_4_(OH), H_2_O	pH: 10.2; duration: 50–70 s; microwave oven (800 W)	rod-arrays film on glass	[557]
Zn(NO_3_)_2_·6H_2_O, polyvinyl pyrrolidone, NH_4_(OH), H_2_O	pH: 10.2; duration: 10 min; microwave oven (1000 W)	star-shaped nanostructures	[558]
Zn(NO_3_)_2_·6H_2_O (0.06 M), NaOH (0.06 M), polyethylene glycols (PEG)-2000, H_2_O	*T*: 180 °C; duration: 10, 20, 30, 60 min; microwave reactor	hierarchical structured nanorods	[559]
ZnSO_4_·7H_2_O (0.1 M), NaOH (0.4 M)	duration: 2 min; microwave oven	nanoparticles (10–15 nm)	[560]
Zn(CH_3_COO)_2_·2H_2_O, NaOH, 1-butyl-3-ethyl imidazolium tetrafluoroborate (C_8_H_15_BF_4_N_2_), H_2_O	*T*: 120–140 °C; duration: 5 min; microwave reactor (800 W)	calthrop-like framework	[561]
Zn(NO_3_)_2_·6H_2_O (0.03 M), NaOH (0.06 M), H_2_O, polyethylene glycol (PEG) 2000, H_2_O	*T*: 180 °C; duration: 30 min; microwave reactor	rods with the diameter of 300 nm and length of 1 µm	[562]
Zn(NO_3_)_2_·6H_2_O, Zn(C_5_H_7_O_2_)_2_·*x*H_2_O, urea, C_2_H_4_(OH)_2_ (different concentrations), H_2_O	*T*: 150 °C; duration: 1–30 min; microwave reactor	microrods with width of 200–300 nm and length of up to 4 µm	[563]
Zn(NO_3_)_2_·6H_2_O, dodecylamine (C_12_H_27_N), H_2_O	*T*: 80–130 °C; duration: 1–50 min; power: 150 W; microwave oven	hexagonal quasi-hourglasses (tripods, tetrapods, pentapods, multipods)	[564]
Zn(CH_3_COO)_2_·2H_2_O, triethanolamine (TEA, C_6_H_15_NO_3_), NH_4_(OH), H_2_O	pH: 6-12; *T*: 80–160 °C; duration: 10–60 min; power: 150 W; microwave reactor	spherical nanoparticles 60–90 nm, rugby-like nanostructures with diameter of 450 nm and length of about 700 nm	[565]
Zn(CH_3_COO)_2_·2H_2_O, triethanolamine (TEA, C_6_H_15_NO_3_, (different concentrations), NaOH (different concentrations), H_2_O	pH: 9.0–12; duration: 90 s; power: 900 W; microwave oven	nanospheres with the crystallite size of 57 nm; raspberry-like nanostructures with the crystallite size of 62 nm; hollow nanospheres with the crystallite size of 78 nm; nanoparticles with the crystallite size of 24 nm	[566]
Zn(NO_3_)_2_·6H_2_O (0.1 M), C_6_H_12_N_4_ (0.1 M)	*T*: 90 °C; duration: 2 h; microwave oven	nanorods on the surface of GaN 80–170 nm, nanorods on the surface of glass 40–100 nm	[567,568,569]
Zn(NO_3_)_2_·6H_2_O, C_6_H_12_N_4_	duration: 5 h; microwave oven	nanorods on the surface of glass, average width of nanorod: 20–1000 nm, average length of nanorod: 150–5000 nm	[570]
Zn(CH_3_COO)_2_·2H_2_O, KOH,	*T*: 90 °C; duration: 10–30 min; microwave oven	micro-tube structure 200–400 nm in diameter, flower-like structure composed of spear-shaped nanorods with diameters and lengths of 70 nm and 1–5 μm,	[571]
Zn(CH_3_COO)_2_·2H_2_O (different concentrations), NaOH, C_2_H_5_OH	pH: 10; *T*: 100 °C duration: 45–60 min; power: 800 W; microwave reactor	plates (SSA: 10.7 m^2^/g), rounded plates (SSA: 9.18 m^2^/g), brush-like (SSA: 9.5 m^2^/g), flower-like (SSA: 8.5 m^2^/g)	[572]
Zn(CH_3_COO)_2_·2H_2_O, NH_4_(OH), H_2_O	pH: 8; duration: 180 s; microwave oven (900 W)	SSA: 22.9 m^2^/g; uniform flower-like nanostructures composed of petals attached in the centre with lengths in the range of 700–950 nm and a width in the range of 130–230 nm; each single petal is composed of nanoparticles with lengths of 45–95 nm	[573]
Zn(CH_3_COO)_2_·2H_2_O, NH_4_(OH), C_25_N_3_H_30_Cl, Polyethylene glycol (PEG) 400, C_19_H_42_BrN, H_2_O	duration: 10 min; microwave reactor	rod-like nanostructures, star-like nanostructures	[574]
ZnSO_4_·7H_2_O, NaOH, H_2_O	pH: 9; duration: 5–25 min; microwave reactor	sheet nanostructures	[575]
Zn(NO_3_)_2_·6H_2_O (0.1 M), NH_4_(OH), H_2_O, cetyltrimethylammonium bromide (CTAB, C19H42BrN)	pH: 7; *T*: 150 °C; duration: 1 h; microwave reactor	nanorods, length: 1–2 μm and width: 100–150 nm	[576]
Zn(CH_3_COO)_2_·2H_2_O, NH_4_(OH), H_2_O	duration: 8 min; power: 900 W; microwave oven	nanoparticles (15 nm) which were self-assembled to form a sheet-like structure	[577]
Zn(NO_3_)_2_·6H_2_O, NaOH, polyvinyl alcohol, H_2_O	duration: 10 min; power: 700 W; microwave oven	nanoparticles (40 nm)	[578]
Zn(NO_3_)_2_·6H_2_O (0.005 M), Zn(CH_3_COO)_2_·2H_2_O (0.005 M), ZnSO_4_·7H_2_O (0.005 M), KOH (2 M), H_2_O	pH: 12; *T*: 130 °C; duration: 1 h; microwave reactor (800 W)	flower-like structures, plates	[579]
ZnCl_2_·2H_2_O (0.5 M), urea, H_2_O	duration: 5 min; power: 800 W microwave reactor	sponge-like nanostructure	[580]
Zn(CH_3_COO)_2_·2H_2_O, Zn(NO_3_)_2_·6H_2_O, ZnCl_2_·2H_2_O, NaOH, KOH, NH_4_(OH), sodium di-2-ethylhexyl-sulfosuccinate (C_20_H_36_Na_2_O_7_S), H_2_O	*T*: 80–140 °C; duration: 5–20 min; power: 300–1200 W; microwave reactor	hexagonal rods (3–4 μm long and 1 μm wide), hexagonal prismatic, bihexagonal rod-like structure (6 μm long and 2 μm wide), hexagonal prismatic particles (60–80 nm in diameter and length between 90 and 110 nm)	[581]
ZnCl_2_·2H_2_O, NH_4_(OH), H_2_O	*T*: 80–140 °C; duration: 20 min; power: 240 W; microwave oven	flower-shaped ZnO microcrystals (about 5 μm)	[582]
Zn(NO_3_)_2_·6H_2_O, hexamethylenetetramine (C_6_H_12_N_4_), H_2_O	*T*: 170 °C; duration: 2–20 min; microwave reactor	irregular sheet-like structures and rods, tripods	[583]
Zn(NO_3_)_2_·6H_2_O, hexamethylenetetramine (C_6_H_12_N_4_)_,_ H_2_O	*T*: 90 °C; duration: 3 h; microwave reactor	nanorods on paper	[584]
Zn(NO_3_)_2_·6H_2_O (0.1 M), NH_4_(OH), albumen, H_2_O	pH: 8; duration: 5 min; microwave oven	sheet-like and spherical-like nanostructures (13–50 nm), nanowhiskers and nanorods (10–57 nm)	[585]
Zn(NO_3_)_2_·6H_2_O (0.03 M), hexamethylenetetramine (C_6_H_12_N_4_), H_2_O	duration: 2–30 min; power: 10–100%; microwave oven (1100 W)	nanostructured-films (networked-nanoflakes morphology)	[586,587]
Zn(CH_3_COO)_2_·2H_2_O, NaOH, glutamic tetrofluoroborate (different concentrations), H_2_O	*T*: 80 °C; duration: 10 min; power: 1000 W; microwave reactor	clew-like hierarchical nanosheet spheres, nanoneedle-like structures	[588]
Zn(NO_3_)_2_·6H_2_O (different concentrations), hexamethylenetetramine (C_6_H_12_N_4_), H_2_O	duration: 5–20 s; power: 180-1100 W; microwave oven	growth of nanorods, diameters: 50–80 nm	[589,590,591]
Zn(NO_3_)_2_·6H_2_O (0.03 M), hexamethylenetetramine (C_6_H_12_N_4_), H_2_O	duration: 30 min; power: 110 W; modified microwave oven (1100 W)	porous nanostructures grown on Al-Si substrate (Al layer thickness form 0 to 150 nm); Al-free Si substrate: nanorods were formed (length: 350 nm, diameter: 50 nm); Al on Si substrate: nanoflake (height ~380 nm ) with pores sizes ranging from 50 nm to several hundreds of nanometres.	[592]
Zn(NO_3_)_2_·6H_2_O (different concentrations), hexamethylenetetramine (C_6_H_12_N_4_, different concentrations), H_2_O	*T*: 80 °C; duration: 10 min; power: 30–50%; microwave oven	ZnO nanoarray (rod-like structures) on glass, size control achieved by regulating the parameters	[473]
Zn(NO_3_)_2_·6H_2_O (different concentrations), NH_4_(OH), H_2_O	duration: 8 min; power: 800 W; microwave oven	flower-like and rod-like structures	[593]
Zn(CH_3_COO)_2_·2H_2_O (0.45 M), NaOH (8 M), Triton X-100	duration: 1–6 min; power: 100–600 W; microwave oven	rods (400–800 nm), flower structures	[594]
Zn(CH_3_COO)_2_·2H_2_O, KOH, H_2_O	duration: 3 min; power: 800 W; microwave oven	hexagonal nanorods (length from ~1.5 μm to 3 μm and in diameter from ~30 nm to 80 nm)	[595]
Zn-dust, HNO_3_, NaOH, polyethylene glycol (PEG, MW 2000), H_2_O	duration: 10–20 min; microwave oven	SSA: 14.4-21.8 m^2^/g; particles with irregular shape (plate and rod-like particles), crystallite size: 34–42 nm	[596]
Zn(CH_3_COO)_2_·2H_2_O, NaOH, cetyltrimethylammonium bromide (CTAB, C_19_H_42_BrN, different concentrations), H_2_O	*T*: 130°; duration: 15–60 min; microwave oven	wire-like architecture with a width in the range of 60–80 nm, flower-like microstructures composed of nanorods, rod has a width of 300–400 nm and a length of 3–4 µm	[597]
Zn(CH_3_COO)_2_·2H_2_O, Zn(NO_3_)_2_·6H_2_O, NaOH, NH_4_(OH), di-2-ethylhexyl sodium sulfosuccinate (C_20_H_36_Na_2_O_7_S), H_2_O	*T*: 80–140 °C; duration: 5–20 min; power: 300–1200 W; microwave reactor	cauliflower-like structures, hexagonal prismatic type particles (200–300 nm)	[598]
Zn(CH_3_COO)_2_·2H_2_O, Zn(NO_3_)_2_·6H_2_O, NH_4_(OH), hydrazine hydrate (N_2_H_4_·H_2_O), H_2_O	pH: 11.5; duration: 10–25 min; power: 510–680 W; microwave oven	nanoparticles, nanorods, flowers	[599]
Zn(CH_3_COO)_2_·2H_2_O, NaOH, H_2_O	*T*: 140 °C; duration: 45 min; power: 400 W; microwave reactor (1600 W)	nanorods, diameter ranging from 60 to 80 nm with average length of about 250 nm	[600]
Zn(CH_3_COO)_2_·2H_2_O (0.005 M), NaOH (0.025 M), H_2_O	duration: 6 min; power: 400–600 W; microwave oven	mixture of nanorods and nanoplates	[601]
Zn(CH_3_COO)_2_·2H_2_O (0.18 M), NaOH (different concentrations), H_2_O	pH: 7–9.5; *T*: 50 °C; duration: 3 min; microwave reactor (600 W)	nanorods (width: 80–300 nm, height: 150–1000 nm) grown on Si, GaAs and GaN substrate	[602]
Zn(CH_3_COO)_2_·2H_2_O (0.18 M), NaOH (different concentrations), H_2_O	pH: 6.75–7.75; *T*: 50 °C; duration: 2 min; microwave reactor (600 W)	rods (width: 0.5–2.5, height: 1.5–2.2 µm) grown on GaN substrate	[603]
Zn(CH_3_COO)_2_·2H_2_O, NH_4_(OH), H_2_O	pH: 10.1–10.9; *T*: 90 °C; duration: 20 min; power: 100 W; microwave oven	nanorods grown on Si substrate	[604]
Zn(NO_3_)_2_·6H_2_O, hexamethylenetetramine (C_6_H_12_N_4_ ), H_2_O	*T*: 90 °C; duration: 2 h (switched on and off automatically); microwave oven	nanorods grown on Si substrate (thickness: ~1 µm)	[605]
Zn(NO_3_)_2_·6H_2_O, hexamethylenetetramine (C_6_H_12_N_4_), H_2_O	duration: 10–30 min; power: 120 W; microwave oven	rods, bipods (length: 0.46–1 µm, width: 0.1–0.13 µm)	[606]
Zn(NO_3_)_2_·6H_2_O, hexamethylenetetramine (C_6_H_12_N_4_), H_2_O, oxalic acid dihydrate (C_2_H_2_O_4_·2H_2_O)	*P*: 20.68 bar; duration: 15 min; microwave reactor (300 W)	nanorods or flower grown on paper	[607]
Zn(NO_3_)_2_·6H_2_O, hexamethylene tetramine (C_6_H_12_N_4_), H_2_O	*T*: 80 °C; duration: 5–20 min; power: 100–1600 W; microwave reactor (1600 W)	nanorods grown on glass substrate	[608]
Zn(NO_3_)_2_·6H_2_O, hexamethylene tetramine (C_6_H_12_N_4_, different concentrations), H_2_O	duration: 10 min; power: 240 W; microwave oven	nanorods (diameter: 89–216 nm) grown on glass substrate	[609]
Zn(NO_3_)_2_·6H_2_O, NH_4_(OH), H_2_O	pH: 10.0–12.0; *T*: 90–120 °C; duration: 1 h min; microwave reactor	nanorods grown on glass substrate, control of size of diameter of rods within the size range between circa 125 and 770 nm	[610]
Zn(CH_3_COO)_2_·2H_2_O (different concentrations), hexamethylenetetramine (C_6_H_12_N_4_), H_2_O	*T*: 60–110 °C; duration: 5–40 min; microwave reactor	nanorods (diameter from ~30 to ~300 nm, length from ~60 nm to ~520 nm) grown on Si substrate	[611]
Zn(CH_3_COO)_2_·2H_2_O, NH_4_(OH), NaOH, CH_3_COOH, H_2_O	pH: 9.8 or 10.8; duration: five steps (each step included 30 s of irradiation and 10 s off); microwave oven	dandelion-like nanostructures (needles: 50–200 nm, height ~2 µm) or a flower-like microstructures grown on activated carbon cloth	[612]
Zn(CH_3_COO)_2_·2H_2_O, NH_4_(OH), palmitic acid (CH_3_(CH_2_)_14_COOH), H_2_O	pH: 4–5; duration: 10–30 min; microwave oven	rod shaped structures	[613]
ZnSO_4_·7H_2_O, NH_4_(OH), H_2_O	pH: 10; duration: 2 min; power: 600 W; microwave oven	nanoparticles (~50 nm)	[614]
Zn(NO_3_)_2_·6H_2_O, potassium sodium citrate, NaOH, H_2_O	*T*: 90 °C; duration: 2 min; power: 600 W; microwave oven (650 W) with a refluxing apparatus	sphere-like particles (~2.32 μm)	[615]
tris(ethylenediamine)zinc nitrate ([Zn(en)_3_](NO_3_)_2_), NaOH, H_2_O	pH: 7–12; *T*: 180 °C; duration: 20 min; power: 400 W; microwave reactor (1600 W)	nanorods, diameter: from 40 nm (pH 12) to 600 nm (pH 7)	[616]
Zn(NO_3_)_2_·6H_2_O, hexamethylenetetramine (C_6_H_12_N_4_)_,_ H_2_O	*T*: 100 °C; duration: 60 min; power: 100 W; microwave reactor	nanorods (180–350 nm) grown on glass	[617]
Zn(NO_3_)_2_·6H_2_O, hexamethylenetetramine (C_6_H_12_N_4_)_,_ polyethylenimine, NH_4_OH, H_2_O	*T*: power on and off in order to control the solution temperature; duration: 6–10 × (30–60 s “on” and 5 min “off”); microwave oven (350 W)	nanorods (180–350 nm) grown on glass	[618]
Zn(CH_3_COO)_2_·2H_2_O, NH_4_(OH), carbon fibre, H_2_O	duration: 3 × (30 s irradiation and 30 s stop); microwave oven (1120 W)	rods (diameter: 0.3–0.5 µm, length: 1.0–1.5 µm) grown on carbon fibre	[619]
Zn(NO_3_)_2_·6H_2_O, NaOH, NH_4_(OH), polyethylene glycol (PEG, MW 400), H_2_O	*T*: 100 °C, duration: 5 min; microwave oven (800 W)	quasi-spherical shape and dimensions of less than 5 μm, flower-like structures (>5 μm),	[620]
Zn(NO_3_)_2_·6H_2_O, C_6_H_12_N_4_, polyethylenimine, NH_4_(OH), H_2_O	duration: 5–15 min; microwave oven (800 W)	nanoflowers and nanowalls grown on P–Si	[621]
Zn(CH_3_COO)_2_·2H_2_O, NaOH, 1-hexyl-2-ethyl-3-methylimidazoliumtetrafluoroborate (C_6_H_11_BF_4_N_2_), H_2_O	duration: 2–9 min; power: 30%; microwave oven	flakes-shaped particles, flower-like shaped particles	[622]
Zn(NO_3_)_2_·6H_2_O, NaOH, plant extract, H_2_O	pH: 10; duration: 15 min; microwave oven	nanoparticles	[623]
Zn(NO_3_)_2_·6H_2_O, (C_6_H_12_N_4_)_2_, H_2_O	*T*: 70–130 °C; duration: 10 min; microwave reactor	nanorods on paper, length 120–480 nm, thickness 55–75 nm	[624]
Zn(NO_3_)_2_·6H_2_O, triethanolamine (TEA, C_6_H_15_NO_3_), H_2_O	duration: 10 min; power: 640 W; microwave oven	nanoparticles	[625]
Zn(CH_3_COO)_2_·2H_2_O, KOH, triethanolamine (TEA, C_6_H_15_NO_3_), 1,2,4,5-benzenetetracarboxylic acid, H_2_O	pH: 8–12; *T*: 150 °C; duration: 30 min; power: 800 W; microwave reactor	dumbbell-like structures, football-like structures, hexagonal bi-pyramidal structures, SSA: 7–24 m^2^/g; size 50 nm–10 μm	[626]
Zn(CH_3_COO)_2_·2H_2_O, NaOH, H_2_O	pH: 8–10; duration: 6 min; microwave oven (700 W)	sheet-like structures and uniform microstructures	[627]
Zn(CH_3_COO)_2_·2H_2_O, Tris (hydroxymethyl) aminomethane (C_4_H_11_NO_3_), H_2_O	duration: 3 min; microwave oven (300 W)	spherical nanoparticles	[628]
Zn(CH_3_COO)_2_·2H_2_O, KOH, benzene-1,2-dicarboxylic acid, benzene-1,3-dicarboxylic acid, benzene-1,4-dicarboxylic acid, H_2_O	pH: 7–12; *T*: 150 °C; duration: 30 min; power: 800 W; microwave reactor	rod-like structures, needle-like structures, platelet-like structures, hexagonal columnar shape of the particles, rice-grain shape structures	[629]
Zn(CH_3_COO)_2_·2H_2_O (0.1 M), NaOH (0.1 M), {4-[(E)-2-(furan-2-yl)ethenyl]pyridin-1-ium-1-yl} acetate (1 wt% and 3 wt%), CH_3_OH, H_2_O	duration: 20 min; microwave oven	heterogeneous shape of nanoparticles, size: 200–800 nm, average crystallite size: 21–23 nm	[630]
Zn(NO_3_)_2_·6H_2_O, hexamethylenetetramine (C_6_H_12_N_4_), NH_4_(OH), H_2_O	pH, 6.8–13, duration: 10–15 min; pulsed microwave heating in microwave oven (850 W)	rod, flower, star, tetrapod	[631]
Zn(NO_3_)_2_·6H_2_O (different concentrations), hexamethylenetetramine (C_6_H_12_N_4_), H_2_O	*T*: 105 °C; duration: 10–30 min; microwave oven (850 W)	growth of nanorods on P-type silicon wafer, diameters: from 26–32 nm to 35–40 nm, lengths: from 330 nm–607 nm	[632]
Zn(NO_3_)_2_·6H_2_O, hexamethylenetetramine (C_6_H_12_N_4_), H_2_O	duration: 20 min; power: 750 W; microwave oven	growth of nanorods on silicon substrate, diameters ~80 nm, lengths ~500 nm	[633]
Zn(NO_3_)_2_·6H_2_O, coffee powder extract, H_2_O	duration: 5 min; power: 540 W; microwave oven	spherical nanoparticles (80–120 nm)	[634]
Zn(NO_3_)_2_·6H_2_O, tomato extract, H_2_O	duration: 5 min; power: 180–540 W; microwave oven	spherical nanoparticles (40–100 nm)	[635]
Zn(NO_3_)_2_·6H_2_O, tea leaf extract, H_2_O	duration: 7 min; power: 540 W; microwave oven	spherical nanoparticles (26 nm)	[636]
Zn(CH_3_COO)_2_·2H_2_O, Longan fruits extract, H_2_O	duration: 1 min on & 1 min off irradiation cycle (1–30 cycles); power: 450–800 W; microwave oven	SSA: 35 m^2^/g, diameter: 10–100 nm, heterogeneous shape	[637]
Zn(CH_3_COO)_2_·2H_2_O (0.22 M), carbinol, H_2_O	duration: 5–15 min; power: 900 W; microwave oven	spherical nanoparticles, diameters: 30 nm–50 nm; hexagonal facetted nanostructures, average size: 400–450 nm	[638]
Zn(NO_3_)_2_·6H_2_O, NH_4_(OH) (28%), H_2_O	pH: 12; duration: 5–25 min; power: 180–540 W; microwave oven (1200 W)	spherical and flower-like particles on paper; non-uniform size	[639]
ZnO powder, hydrogen peroxide (H_2_O_2_, 30%)	*P*: 30 bar; duration: 15 min; power: 1200 W; microwave oven (1200 W)	rod-like nanostructures, average size: 36 nm	[640]

**Table 6:** Summary of microwave hydrothermal synthesis of ZnO nanostructures with additional heat treatment.

Substrates	Conditions during Preparation	Parameters of Additional Heat Treatment	Properties	Ref.
Zn(CH_3_COO)_2_·2H_2_O, NH_4_OH, H_2_O	duration: 5–10 min; power: 240–400 W; microwave oven	500 °C in air for 1 h	dumbbell-shaped structures built of particles sized ~100 nm	[641]
Zn(CH_3_COO)_2_·2H_2_O, KOH, H_2_O	duration: 15 min (irradiation 12 s, stop 10 s); power: 180 W; microwave oven	400 °C in air for 1 h	nanorods assembled in flower shaped, rods: diameter 150–190 nm (tip diameter ~15 nm), length 2 μm, with an aspect ratio of 20–22	[642,643]
Zn(CH_3_COO)_2_·2H_2_O, NaOH, guanidinium carbonate, acetyl acetone (ACAC), H_2_O	pH: 8–12; duration: 2 min; power level: 75%; microwave oven	without and 600 °C in air for 2 h	petals: length 600–700 nm, width 300–400 nm, tip 50–70 nm; rod-like nanostructures diameters 60–90 nm maximal length 1.5 µm; spherical-like nanostructures: diameter 50 nm	[644]
Zn(CH_3_COO)_2_·2H_2_O (0.2 M), NaOH (0.4 M), H_2_O, triethanolamine (TEA, C_6_H_15_NO_3_)	duration: 20 min; power: 20%; microwave oven	900 °C in air for 1 h	spherical particles ~50 nm	[645]
Zn(NO_3_)_2_·6H_2_O, hexamethylenetetramine (C_6_H_12_N_4_), H_2_O	duration: 2 h; microwave oven	250–550 °C for 1 h in oxygen flow (5 cm^3^/min)	nanorods (diameter from 50–300 nm) grown on surface of silicon substrates	[568]
Zn(NO_3_)_2_·6H_2_O, hexamethylenetetramine (C_6_H_12_N_4_), polyethylenimine, NH_4_OH, H_2_O	duration: 4–80 min; power: 180–850 W; microwave oven	350 °C for 20 min in air	nanowire grown on an ITO-coated glass substrate	[646]
Zn(CH_3_COO)_2_·2H_2_O, NaOH, H_2_O	duration: 10 min; power: 200 W; microwave oven	400–800 °C for 1 h in air	circular- and hexagonal-shaped particles	[647]
Zn(CH_3_COO)_2_·2H_2_O, sodium dodecyl, NH4OH, (CH_3_)_2_CHOH (2-propanol) H_2_O	duration: 2 h; power: 100–800 W; microwave oven	500 °C for 3 h in air	flakes-like structures, spherical-like, crystallite size: 31–39 nm (morphology dependent on the microwave power)	[648]
Zn(CH_3_COO)_2_·2H_2_O, ZnCl_2_·2H_2_O, Zn(NO_3_)_2_·6H_2_O, pyridine (C_5_H_5_N), H_2_O	pH: 13.75; duration: 2–5 min; power: 1000 W; microwave oven	300–500 °C for 2 h in air	nanoparticles, nanoflowers, nanorods	[649]
Zn(CH_3_COO)_2_·2H_2_O, urea, H_2_O	*T*: 220 °C; duration: 15 min; microwave oven	400 °C for 90 min in air	nanosheets	[650]
Zn(NO_3_)_2_·6H_2_O, hexamethylenetetramine (C_6_H_12_N_4_), NaOH, H_2_O	pH: 13; *T*: 90–220 °C; duration: 15 min; power: 110–710 W; microwave reactor (1400 W)	200 °C for 2 h in air	nano-platelets	[651]
Zn(CH_3_COO)_2_·2H_2_O, trisodium citrate dihydrate (Na_3_C_6_H_5_O_7_·2H_2_O) (different amounts), NH_4_OH, H_2_O	*T*: 90 °C; duration: 10–45 min; power: 300 W; microwave reactor	400 °C for 4 h in air	hollow microspheres average dimensions ~4 μm; thickness 400–600 nm.	[652]
Zn(NO_3_)_2_·6H_2_O, NH_4_OH, albumen, H_2_O	pH: 8; duration: 5 min; microwave oven (1000 W)	130 °C for 5 h in air	whisker-like and rod-like nanostructures: thickness 10–57 nm	[585]
Zn(NO_3_)_2_·6H_2_O, KOH, H_2_O	pH: 9–13; duration: 30 min; power: 180 W microwave oven	200 °C for 2 h in vacuum	nanorods	[653]
Zn(NO_3_)_2_·6H_2_O, urea (different concentrations), H_2_O	*T*: 90 °C; duration: 40 min; power: 400 W microwave oven	400–600 °C for 3 h in different atmospheres (O_2_, N_2_, H_2_ and air),	various flower-like nano and microstructures, urchin-like structures	[654]
Zn(CH_3_COO)_2_·2H_2_O, NH_4_OH, hexamethylenetetramine ((CH_2_)_6_N_4_), hexadecyl trimethyl ammonium bromide, polyethylene glycol (PEG400), H_2_O	duration: 10 min; power: 300 W; microwave reactor	500 °C for 2 h in air	needle-assembled structures with flower-like morphology; bundle-like microstructures assembled by nanorods; flower-like hierarchical structures composed of some tight aggregations	[655]
Zn(CH_3_COO)_2_·2H_2_O, trisodium citrate dihydrate (Na_3_C_6_H_5_O_7·_2H_2_O), urea, H_2_O	*T*: 140 °C; duration: 20 min; microwave reactor	500 °C for 2 h in air	porous core–shell microstructures	[656]
Zn(NO_3_)_2_·6H_2_O, NaOH, H_2_O,	pH: 13; duration: 1–5 min with 30 s on–off cycling mode; power: 900 W; microwave oven	400 °C for 1 h in air	microspheres	[657]
Zn(CH_3_COO)_2_·2H_2_O, NaOH, H_2_O,	pH: 12.66–13; duration: 10–30 min; power: 300 W; microwave reactor	500 °C for 2 h in air	nanorods, nanoplates	[658]
Zn(NO_3_)_2_·6H_2_O, hexamethylenetetramine (C_6_H_12_N_4_), H_2_O	duration: 45 min; power: 1800 W; microwave oven	350 °C for 1 h in air	nanorods, length: ~4.3 ± 0.2 µm, diameter: 100 ± 10 nm	[659]
Zn(NO_3_)_2_·6H_2_O, hexamethylenetetramine (C_6_H_12_N_4_) (different concentrations), H_2_O	duration: 10 min; power: 750 W; microwave reactor	400 °C for 1 h in air	shape from random spherical to highly conserved hexagonal shaped rods, size: from ~25 nm to µm/sub µm	[660]
Zn(NO_3_)_2_·6H_2_O, hexamethylenetetramine (C_6_H_12_N_4_), hydrazine hydrate (N_2_H_4_), H_2_O	pH: 10; duration: 10–30 min; microwave reactor (1000 W)	500 °C for 2 h in air	nanorods, length: 579–909 nm, diameter: 116–240 nm	[661]
Zn(CH_3_COO)_2_·2H_2_O, Tuber (Amorphophallus konjac) extract, H_2_O	duration: 5 min; microwave oven	400 °C for 1 h in air	rice shaped nanoparticles, length: 237 nm, diameter: 76 nm	[662]
ZnCl_2_, NaOH, H_2_O	pH: 6.1–13.7; *T*: 80 °C; duration: 5–20 min; microwave reactor (900 W)	150 °C for 3 h in air	hexagonal flake, velvet flower-like, rough globular, needle bunch-like, cauliflower-like, clew-like, nanorods, and rhombic microstructures	[663]
Zn(CH_3_COO)_2_·2H_2_O, ZnCl_2_·3H_2_O, Zn(NO_3_)_2_·6H_2_O, ZnSO_4_·7H_2_O, NH_4_OH, H_2_O	duration: 10 min; microwave reactor	600 °C for 2 h in air	nanoflakes, nanorods, hexagonal tubular, pseudo-spherical	[664]
Zn(NO_3_)_2_·6H_2_O, NaOH, H_2_O (solvent) and C_2_H_5_OH (solvent)	P: 20–40 bars; duration: 15 min; microwave reactor	750 °C for 1 h in air	particles, irregular shape	[665]
ZnCl_2_·3H_2_O, H_2_O, microcrystalline cellulose	*T*: 100 °C; duration: 10–60 min; microwave oven	600 °C for 3 h in air	heterogeneous nanostructures and microstructures	[666]
Zn(CH_3_COO)_2_·2H_2_O, ZnCl_2_·3H_2_O, ZnSO_4_·7H_2_O, ZnCO_3_, Psidium guajava Linn. Extract, H_2_O	*T*: 100 °C; duration: varying cycles of 3 min-on and 1 min-off min; power: 720 W; microwave oven	900 °C for 1.5 h in air	diameter: 60–180 nm	[667]
Zn(NO_3_)_2_·6H_2_O, pelargonium leaf extract, H_2_O	duration: 3 min; power: 800 W; microwave oven	400 °C for 2 h in air	heterogeneous particles	[668]
ZnSO_4_·7H_2_O (different concentrations), banana corm extract, H_2_O	duration: 15 min; power: 540 W; microwave oven	400 °C for 3 h in air	microparticles	[669]
Zn(CH_3_COO)_2_·2H_2_O, KOH, H_2_O	duration: 0.5–2 min; microwave oven (1000 W)	400 °C for 1 h in air	nanorods, tetrapods (length: 255 nm), flowers (petal length: 387 nm)	[670]
Zn_5_(OH)_8_(NO3)_2_·2H_2_O, 1,2,3- trimethyl-imidazole tetrafluoroborate, H_2_O	duration: 2 h; power: 180 W; microwave oven (900 W)	400 °C for 2 h in air	nanobelts, width: 500–800 nm; length: several micrometres, thickness: 100 nm	[671]
Zn(CH_3_COO)_2_·2H_2_O, NH_4_OH, C_2_H_5_OH, H_2_O	*T*: 120 °C; duration: 10 min; power: 180 W; microwave reactor	527 °C in air	heterogeneous hollow NPs, SSA: 17.1 m^2^/g	[672]
Zn(NO_3_)_2_·6H_2_O, NaOH, polyethylene glycols (MW= 1500 and MW= 4000), sugar, cassava starch, H_2_O	duration: 10 min (with 5 s/15 s on/off step); power: 320 and 480 W; microwave oven	450 °C for 1 h in air	rod-like structures and needle-like structures, length: 300–3000 nm	[673]

**Table 7:** Summary of the microwave hydrothermal synthesis of ZnO nanocomposites or ZnO hybrid nanostructures without any additional heat treatment.

Type of Composite	Substrates	Conditions during Preparation	Properties	Ref.
Mn (5%) doped ZnO	Zn(CH_3_COO)_2_·2H_2_O, Mn(CH_3_COO)_2_·4H_2_O, NaOH, polyvinylpyrrolidone	*T*: 60 °C, power: 700 W; microwave oven	nanoparticles sized 10–59 nm	[674]
Mn (5–70% wt%) doped ZnO	Zn(NO_3_)_2_·6H_2_O, Mn(NO_3_)_2_·4H_2_O, KOH, H_2_O	*P*: 38 bars; duration: 15 min; microwave reactor	nanoparticles, irregular spherical shape, ZnO and ZnMn_2_O_4_ phases, size: 33–99 nm	[675]
ZnO/ZnMn_2_O	Zn(NO_3_)_2_·6H_2_O, Mn(NO_3_)_2_·4H_2_O, KOH, H_2_O	*P*: 38 bars; duration: 15 min; microwave reactor	ZnO (30 wt%)-MnO (70 wt%); nearly spherical in shape and agglomerated to the form of irregular clusters; crystallite size of ZnO and ZnMn_2_O_4_ was 99 and 27 nm, respectively; SSA = 25 m^2^/g	[676,677]
Fe (0.18, 1.70 and 3.05 at%) doped ZnO	ZnSO_4_·7H_2_O, FeSO_4_·7H_2_O, NaOH, H_2_O	power: 140 W; microwave oven	nanorods length ~1 µm and diameter in the range of ~50 nm	[678]
Fe doped ZnO	Zn(CH_3_COO)_2_·2H_2_O, Fe(NO_3_)_3_⋅9H_2_O, NH_4_(OH), H_2_O	*T*: 80 °C; power: 400 W; duration: 40 min; microwave oven	star-like structure (~433 nm)	[679]
ZnO/ZnFe_2_O_4_, Fe (5–95%) doped ZnO	Zn(NO_3_)_2_·6H_2_O, Fe(NO_3_)_2_·4H_2_O, KOH, H_2_O	*P*: 39 bars; duration: 15 min; microwave reactor	particles, irregular spherical shape, diameter 4–13 nm	[680,681,682,683,684,685]
Ce (0–0.15 mol) doped ZnO	Zn(CH_3_COO)_2_·2H_2_O, Ce(SO_4_)_2_, NaOH, H_2_O	pH: 13; duration: 10 min; power: 55%; microwave oven (1000 W)	nanosheets	[686]
Ce doped ZnO	Zn(CH_3_COO)_2_·2H_2_O, Ce(CH_3_COO)_3_·*x*H_2_O, NH_3_·H_2_O, H_2_O	pH: 9.5–11; duration: 5–15 min (10/20 s power on/off); power: 200 W; microwave oven	heterogeneous particles (2% of dopant)	[687]
CoO doped ZnO	Zn(NO_3_)_2_·6H_2_O, Co(NO_3_)_2_·4H_2_O, KOH, H_2_O	duration: 15 min; pressure: 38 bar; microwave reactor	CoO content from 5 to 50%, particles >100 nm	[688,689,690,691]
K (0–5 mol%) doped ZnO	Zn(NO_3_)_2_·6H_2_O, KNO_3_, H_2_O	*T*: 160 °C; duration: 30 min; microwave reactor	lamellar-like and granule-like structures (100–300 nm)	[692]
Ce (5 wt%) doped ZnO, Ce (5 wt%) doped carbon nanotube/ZnO	Zn(CH_3_COO)_2_·2H_2_O, Ce(SO_4_)_2_, NaOH, H_2_O, commercial multi-walled carbon nanotubes (length 5–9 μm, diameter 110–170 nm)	pH: 11; duration: 5 min; power: 450 W; microwave oven	nanorods on carbon nanotubes	[693]
Eu doped ZnO	Zn(NO_3_)_2_·5H_2_O, Eu(NO_3_)_3_·5H_2_O, NH_4_(OH), H_2_O	pH: 10; pressure: 1–100 bar; duration: 20 min; microwave reactor	nanoplate-like structures, elongated hexagonal prisms	[694]
Cu doped ZnO	Zn(CH_3_COO)_2_·2H_2_O, Cu(CH_3_COO)_2_·2H_2_O, NaOH, H_2_O	*T*: 100 °C; duration: 20 min; microwave reactor	Zn_1−*x*_Cu*_x_*O (*x* = 0.00, 0.01, 0.02, 0.03, and 0.04), particles	[695]
Ga doped ZnO	Zn(CH_3_COO)_2_·2H_2_O, Ga(NO_3_)_3_·*x*H_2_O, NH_4_(OH), H_2_O	pH: 10; duration: 15–20 min; microwave oven (850 W)	Zn_(1−*x*)_Ga*_x_*O (*x* = 1, 2, and 5 mol%), nanorods are grown on p-Si substrates	[696]
Ga doped ZnO	Zn(CH_3_COO)_2_·2H_2_O, Ga(NO_3_)_3_·xH_2_O, KOH, H_2_O	*T*: 180 °C; duration: 15 min; power 250 W; microwave reactor (1900 W)	Zn_(1-x)_Ga_x_O (x=0, 0.05, 1.00, 3.00 mol%), micron-sized rods (slightly above 1 mm in length)	[697]
Cd doped ZnO/ carbon nanotube	commercial carbon nanotube diameter 10–20 nm and length 30 μm (modified HNO_3_), Zn(CH_3_COO)_2_·2H_2_O, Cd(NO_3_)_2_·5H_2_O, NH_4_(OH), H_2_O	pH: 10; duration: 6 min; power: 490 W; microwave reactor (1000 W)	growing Cd doped ZnO nanoparticles over the surface of carbon nanotube	[698]
Sr doped ZnO	Zn(NO_3_)_2_·6H_2_O, Sr(NO_3_)_2_·6H_2_O (different concentrations), NaOH, H_2_O	*T*: 160 °C, duration: 30 min; microwave reactor	Zn_1−*x*_Sr*_x_*O (*x* = 0.00, 0.001, 0.002, and 0.003), ZnO - lamellar structures, size: 200–300 nm; Zn_1−*x*_Sr*_x_*O - heterogeneous granule nano and microstructures	[699]
ZnO/ZnS	ZnCl_2_·2H_2_O, NH_4_(OH), thioacetamide, H_2_O	duration: 30 min; power: 400 W; microwave refluxing system	core–shell nanorods	[700]
Au-decorated ZnO	Zn(NO_3_)_2_·6H_2_O, hexamethylenetetramine, NaOH, HAuCl_4_·3H_2_O, sodium citrate dihydrate, H_2_O	duration: 10 min; power: 475 W; microwave oven	growing Au NPs (~20 nm) over the surface of ZnO nanorods (diameter of 162 nm and an average length of 1.27 μm)	[701]
Au-decorated ZnO	Zn(CH_3_COO)_2_·2H_2_O, Ag(NO_3_)·6H_2_O, C_6_H_12_N_4_, H_2_O	duration: 5 min; microwave oven	ZnO particles sized up to 2 μm and spherical particles are sized up to 200 nm	[702]
Ag-decorated ZnO	ZnO, Ag(NO_3_), cetyltrimethylammonium bromide (CTAB, C_19_H_42_BrN), H_2_O	duration: 20 min; power: 900 W; microwave oven	spike-like nanostructures, length: few microns, diameter: 50–100 nm	[638]
Ag-ZnO	Zn(NO_3_)_2_·6H_2_O, Ag(NO_3_), C_6_H_12_N_4_, H_2_O	*T*: 120 °C, duration: 30 min; power: 400 W; microwave reactor	star-like structures (up to 2 μm), rod-like structures (~0.5 μm)	[703]
Ag-ZnO	Zn(CH_3_COO)_2_·2H_2_O, AgNO_3_, C_2_H_4_(OH)_2_ (ethylene glycol, EG), Na_2_O_2,_ H_2_O	duration: 5 min; power: 400 W, microwave oven	flower-like structures Ag-ZnO with a molar ratio of 0:100; 2:98; 4:96; 6:94; 8:92; 10:90	[704]
Ag-ZnO	ZnO (nanorods 10–20 nm), AgNO_3_, glucose, H_2_O	pH: 7; duration: 10 min; microwave oven	Ag/ZnO nanoparticles (ZnO nanorods 10–20 nm; Ag nanoparticles ~60 nm)	[705]
Ag-ZnO-clay composite, ZnO-clay composite	chemically activated bentonite clay, AgNO_3_, ZnO NPs, H_2_O	*T*: 80 °C, duration: 20 min; power: 500 W; microwave reactor	nanoparticles in clay, ZnO NPs size 15–70 nm, Ag NPs size 9–30 nm, SSA(ZnO-clay) = 33 m^2^/g, SSA(Ag-ZnO-clay) = 25 m^2^/g	[706]
ZnO-reduced graphene oxide	graphite (modified Hummers method), ZnSO_4_·7H_2_O, NaOH, H_2_O	pH: 8; *T*: 150 °C, duration: 10 min; microwave reactor	graphene nanosheets are decorated densely by ZnO nanosheets (20–30 nm),	[707]
ZnO-reduced graphene oxide	graphite (modified Hummers method), ZnSO_4_·7H_2_O, NaOH, H_2_O	pH: 9; *T*: 150 °C, duration: 30 min; microwave reactor	graphene sheets packed by nanosized and irregularly shaped ZnO nanoparticles	[708]
ZnO-reduced graphene oxide	graphite (modified Hummers method), Zn(NO_3_)_2_·6H_2_O, NaOH, H_2_O	pH: 9–11; *T*: 100 °C, duration: 30 min; microwave reactor	reduced graphene oxide sheets with wrinkles and folds are decorated densely by the ZnO nanorods	[709]
ZnO-reduced graphene oxide	graphite (modified Hummers method), Zn(CH_3_COO)_2_·2H_2_O, HN(CH_2_CH_2_NH_2_)_2_, NaOH, H_2_O	pH: 13; duration: 30 min; microwave oven	reduced graphene oxide sheets with ZnO rod-like (diameter ~100 nm and length ~1 μm)	[710]
ZnO-reduced graphene oxide	graphite (modified Hummers method), ZnCl_2_·2H_2_O, NaOH, H_2_O	duration: 5 min; power: 450 W; microwave oven	nanowires of ZnO were decorated/anchored on the surface of graphene oxide	[711]
ZnO-reduced graphene oxide	graphite (modified Hummers method), Zn(CH_3_COO)_2_·2H_2_O, NaOH, H_2_O	duration: 20 min; power: 450 W; microwave oven	reduced graphene oxide sheets with ZnO nanoparticles (10–20 nm)	[712]
ZnO-reduced graphene oxide	graphite (modified Hummers method), Zn(CH_3_COO)_2_·2H_2_O, KOH, H_2_O	*T*: 100 °C; duration: 8 min; power: 450 W; microwave reactor	reduced graphene oxide sheets with ZnO nanoparticles (irregular elongated shapes and agglomerates with particle size of 122 nm)	[713]
ZnO, ZnO-reduced graphene oxide	graphite (modified Hummers method), Zn(NO_3_)_2_·6H_2_O, hexamethylenetetramine (HMTA), polyvinylpyrrolidone (PVP), H_2_O	*T*: 100 °C; duration: 8 min; power: 450 W; microwave reactor	reduced graphene oxide nanosheets with ZnO nanostars, SSA = 34.3 m^2^/g, size of ZnO nanostars: ~625 nm	[526]
ZnO-TiO_2_-reduced graphene oxide	graphite (modified Hummers method), commercial TiO_2_, ZnSO_4_·7H_2_O, NaOH, H_2_O	pH: 9; *T*: 150 °C, duration: 10 min; power: 150 W; microwave reactor	reduced graphene oxide nanosheets are decorated by ZnO nanosheets and TiO_2_ nanoparticles	[714]
ZnO-MOF-reduced graphene oxide	zeolitic imidazolate framework-8, graphite (modified Hummers method), ZnO, H_2_O_2_, H_2_O	*T*: 150 °C, duration: 10 min; power: 150 W; microwave reactor	ZnO-MOF-reduced graphene oxide with 0, 0.5, 1.0, 1.5 and 2 wt% reduced graphene oxide	[715]
ZrO_2_-ZnO	ZnSO_4_·5H_2_O (1 M), Na_2_CO_3_ (2 M), ZrOCl_2_·8H_2_O (1 M), H_2_O	*T*: 180 °C, duration: 10 min; microwave reactor	needle-shaped micro- and nanoparticles, mass concentrations of ZrO_2_: 1%, 5%, 10%, 20%	[716]
ZrO_2_ coated ZnO	ZnO NPs (diameter: 12–25 nm, 38.4 m^2^/g), (Zr(SO_4_)_2_·4H_2_O), NH_4_OH	*T*: 70 °C, duration: 5 min; microwave reactor (500 W)	core-shell nanocomposites	[717]
ZnO chitosan/ZnO	Zn(NO_3_)_2_·2H_2_O, NaOH, chitosan, H_2_O	duration: 4–8 min; power: 400–800 W; microwave oven	nanoparticles, diameter: 32–82 nm	[718]
Fe_3_O_4_/ZnO/AgBr	Zn(NO_3_)_2_·2H_2_O, FeCl_3_, AgNO_3_, NaBr, NH_4_OH, NaOH, C_2_H_5_OH, malt extract agar (MEA), H_2_O	duration: 10 min; power: 550 W (55%); microwave oven (1000 W)	composition (weight ratio): Fe_3_O_4_/ZnO (1:8), Fe_3_O_4_/ZnO/AgBr (1:2; 1:4; 1:6; 1:8; 1:10); in homogeneous oval particles	[719]
MoS_2_/ZnO, ZnO	ZnCl_2_·2H_2_O, Na_2_MoO_4_·2H_2_O, C_2_H_5_NS, H_2_O	*T*: 60 °C; duration: 20 min; power: 140 W	heterogeneous nano- and microstructures	[720]
ZnO doped CeO_2_	ZnCl_2_·2H_2_O, Ce(NO_3_)_3_·6H_2_O; NH_4_OH, H_2_O	pH: 8; *T*: 200 °C, duration: 30 min; microwave reactor (1500 W)	homogeneous spherical particles (diameter ≈ 5–40 nm)	[721]
mesoporous Si@ZnO	Zn(NO_3_)_2_·6H_2_O, 3-aminopropyl trimethoxy silane (APTMS, (CH_3_O)_3_-Si-(CH_2_)_3_NH_2_), LiOH, H_2_O	*T*: 80 °C; duration: 30 min; power: 300 W; microwave reactor (1000 W)	APTMS/ZnO molar ratios: 0.15, 0.2, 0.3 and 0.4.; agglomerate size ≈ 200 nm	[722]
ZnO/zinc aluminium hydroxide	Zn(CH_3_COO)_2_·2H_2_O, AlCl_3_, NH_4_OH, tripotassium citrate monohydrate (HOC(COOK)(CH_2_COOK)_2_·H2O, H_2_O	*T*: 95 °C; duration: 20 min; microwave reactor	ZnO nanorod on zinc aluminium hydroxide heterostructures; sunflower-like ZnO nanorods on zinc aluminium hydroxide heterostructures; ZnO nanotubes on zinc aluminium hydroxide heterostructures; ZnO film on zinc aluminium hydroxide heterostructures	[723]

**Table 8:** Summary of the microwave hydrothermal synthesis of ZnO nanocomposites or ZnO hybrid nanostructures with additional heat treatment.

Type of Composite	Substrates	Conditions during Preparation	Parameters of Additional Heat Treatment	Properties	Ref.
ZnO/TiO_2_	Zn(NO_3_)_2_·6H_2_O, C_4_K_2_O_9_Ti·2H_2_O, NH_4_(OH), H_2_O	pH: 10; duration: 40 min; power: 180 W; microwave oven	without and *T*: 500–700 °C; duration: 3 h	without calcination: nanoparticles <10 nm; after calcination: nanoparticles 25 nm and 80–100 nm	[724]
ZnO/TiO_2_	Zn(CH_3_COO)_2_·2H_2_O, titanium isopropoxide, NaOH, H_2_O	*T*: 180 °C; duration: 5 min; microwave reactor	*T*: 500–800 °C	rods: diameters: 150–250 nm, length: 1000–2000 nm	[725]
ZnO/CuO	Zn(CH_3_COO)_2_·2H_2_O, Cu(CH_3_COO)_2_·2H_2_O NaOH, H_2_O	duration: 15 min; power: 450 W; microwave oven	*T*: 800 °C; duration: 7 h	nanorod like structures	[726]
Cr doped ZnO	Zn(CH_3_COO)_2_·2H_2_O, (Cr(CH_3_COO)_2_)_2_·2H_2_O, NaOH, H_2_O	duration: 5 min; power: 400 W; microwave oven (1350 W)	*T*: 700 °C; duration: 2 h	particles (~100 nm)	[727]
Cr doped ZnO	Zn(NO_3_)_2_·6H_2_O, Cr(NO_3_)_3_·9H_2_O, citric acid (C_6_H_8_O_7_), H_2_O	microwave oven (650 W)	250 °C for 1 h in air	doped concentration (5%, 10%, and 15%), porous structures	[728]
Co doped ZnO	Zn(NO_3_)_2_·6H_2_O, Co(NO_3_)_2_·6H_2_O, citric acid (C_6_H_8_O_7_), H_2_O	duration: until reaction was completed; microwave oven (650 W)	250 °C for 1 h in air	doped concentration (5%, 10%, and 15%), porous structures	[728]
Co doped ZnO	Zn(CH_3_COO)_2_·2H_2_O, Co(CH_3_COO)_2_·4H_2_O, polyethylene glycol, NaOH, H_2_O	pH: 9; duration: 30 min; power: 300 W; microwave oven	*T*: 400 °C; duration: 1 h	Zn_1−*x*_Co*_x_*O (*x* = 0.00, 0.01, 0.03 and 0.05), needle shaped microstructures, nanospheres	[729]
CdO-ZnO	ZnCl_2_·2H_2_O, CdCl_2_·2H_2_O, NH_4_OH	pH: 8; duration: 10–20 min; microwave oven (1000 W)	500 °C for 4 h in air	heterogeneous nanostructures and microstructures	[730]
Eu doped ZnO	Zn(NO_3_)_2_·5H_2_O, Eu(NO_3_)_3_·5H_2_O, NaOH, H_2_O	pressure: 60 bar; duration: 15 min; microwave reactor (600 W)	750 °C for 1 h in air	10 mol% of Eu, heterogeneous size and shape of NPs	[731]
In_2_O_3_-ZnO	Zn(CH_3_COO)_2_·2H_2_O, In(NO_3_)_3_·4.5H_2_O, CO(NH_2_)_2_, H_2_O	*T*: 120 °C; duration: 30 min; power: 800 W; microwave reactor	550 °C for 2 h in air	n(In):n(Zn) (0.03:1, 0.05:1, 0.07:1), rods (long: 200–300 nm; wide 75 nm) and flowers	[732]
Sn doped ZnO	Zn(NO_3_)_2_·6H_2_O, SnCl_4_, H_2_O	duration: 5 min; microwave oven	400 °C for 3 h in air	0, 5, 10 and 15 wt% Sn doped ZnO; needle-like structures for the pure ZnO (30–70 nm); agglomerated spherical crystallites in all Sn doped ZnO samples	[733]
ZnO-reduced graphene oxide	graphite (modified Hummers method), ZnCl_2_·2H_2_O, NaOH, H_2_O	duration: 2 min; power: 450 W; microwave oven	stainless steel (SS-316) Teflon lined autoclaves were kept at 150 °C in hot air oven for 24 h	SSA: 140–182 m^2^/g; flower-like ZnO nanoparticles well decked on graphene/graphene oxide sheet	[734]
Ag-ZnO	Zn(NO_3_)_2_·6H_2_O, AgNO_3_, citric acid (C_6_H_8_O_7_)	duration: 15–20 cycles (cycling mode: on for 30 s and off for 30 s); power: 800 W; microwave oven	500 °C for 2 h in air	nanoparticles; SSA: 61 m^2^/g; size: ~17 nm	[735]
Ag/Ag_2_SO_4_/ZnO	Zn(CH_3_COO)_2_·2H_2_O, AgNO_3_ (different concentrations), urea, thiourea (different concentrations),	duration: 30 min; power: 400 W; microwave reactor	500 °C for 4 h in air	plate-like aggregates, diameter: 1.5–2 µm, thickness: 100–200 nm	[736]
Ag-N co-doped ZnO	Zn(NO_3_)_2_·6H_2_O, hexamethylenetetramine (HMT; C_6_H_12_N_4_), CH_3_COONH_4_, AgNO_3_	duration: 40 min; power: 500 W; microwave oven	800 °C for 1 h in oxygen	Ag-N co-doped ZnO nanorods (diameter: 50–200 nm) were vertically grown on n-type Si substrate	[737]
Au-ZnO	Zn(NO_3_)_2_·6H_2_O, hexamethylenetetramine (HMT; C_6_H_12_N_4_), hydrazine hydrate (N_2_H_4_), HAuCl_4_, H_2_O	pH: 10; microwave oven (1000 W)	500 °C in oxygen	Au NPs on the surface of ZnO nanorods (width: 140 nm, length: 626 nm) Au size of NPs: 11–36 nm	[738]

**Table 9:** Summary of boiling points of several selected organic solvents for the pressure of 1013.25 hPa.

Solvent	Boiling Point (°C)
Acetone	56.2
Methanol	64.6
Tetrahydrofuran	65–66
Ethanol	78.5
2-Propanol	82.4
1-Propanol	97.0
Water	100.0
1-Butanol	117.6
1-Hexanol	156.5
Ethylene glycol	197.6
1,3-Butanediol	207.0
Benzyl alcohol	203–205
1,4-Butanediol	228.0
Diethylene glycol	244.9
Triethylene glycol	288.0
Glycerine	290.0
Tetraethylene glycol	~327

**Table 10:** Summary of the microwave solvothermal synthesis of ZnO without any additional heat treatment.

Substrates	Conditions during Preparation	Properties	Ref.
Zn(CH_3_COO)_2_·2H_2_O, C_2_H_4_(OH)_2_ (solvent), H_2_O (different concentrations)	*T*: 190–220 °C, duration: 25 min; power: 100%; microwave reactor (600 W)	control of size of particles within the size range between circa 15 and 120 nm	[402,758]
Zn(CH_3_COO)_2_·2H_2_O, diethylene glycol (solvent), triethylene glycol (solvent), tetraethylene glycol (solvent), H_2_O (constant concentration)	*T*: 190 °C, duration: 25 min; power: 100%; microwave reactor (600 W)	spherical nanoparticles and rod-like shape nanoparticles; diameters: 32–47 nm	[402]
Zn(CH_3_COO)_2_·2H_2_O, C_2_H_4_(OH)_2_ (solvent), H_2_O (constant concentration)	pressure: 4 bar; duration: 12 min; power: 1000–3000 W; microwave reactor (3000 W)	nanoparticles (27 nm) aggregates precise size-control ranging from about 60 to 120 nm	[502]
Zn(CH_3_COO)_2_·2H_2_O (different concentrations), diethylene glycol (solvent)	*T*: 180 °C, duration: 5 min; microwave reactor (300 W)	nanoparticles (6–10 nm) clusters precise size-control ranging from about 57 to 274 nm	[759]
Zn(CH_3_COO)_2_·2H_2_O (different concentrations), oleic acid, diethylene glycol (solvent)	*T*: 250 °C, duration: 15 min; power: 100%; microwave reactor	nanoparticles (4–14 nm)	[760]
Zn(CH_3_COO)_2_·2H_2_O, C_2_H_4_(OH)_2_ (ethylene glycol - solvent), H_2_O	different irradiation cycling modes, duration: 3–60 min; power: 200–600 W; microwave oven (750 W)	straw-bundle-like, wide chrysanthemum-like, nanorod-based microspheres, microspheres (irregular), nanorod-based microspheres, mixture of straw-bundle-like, wide chrysanthemum-like oat-arista-like	[761]
Zn(CH_3_COO)_2_·2H_2_O, C_6_H_5_CH_2_OH (anhydrous benzyl alcohol, solvent)	*T*: 120–180 °C duration: 30 s–35 min; power: 300 W; microwave reactor	nanoparticles (4–8 nm)	[762]
Zn(CH_3_COO)_2_, Zn(C_5_H_7_O_2_)_2_, C_6_H_5_CH_2_OH (benzyl alcohol, solvent)	*T*: 120–180 °C duration: 30 s–35 min; power: 300 W; microwave reactor	nanoparticles (20 nm for Zn(C_5_H_7_O_2_)_2_; 25–30 nm for Zn(CH_3_COO)_2_)	[763]
Zn(CH_3_COO)_2_·2H_2_O, C_2_H_5_OH (solvent); C_3_H_7_OH (solvent), H_2_O (solvent), NaOH	duration: 5 min; power: 150 W; microwave oven	water (solvent): ZnO nanoparticles had elliptical shape and large size with size of larger axis of about 100 nm and size of the other axis of about 40 nm; ethanol (solvent): rod nanostructures form with length of ~45 nm and radius of ~20 nm; isopropanol (solvent): spherical particles with radius of 10–12 nm.	[764]
Zn(CH_3_COO)_2_·2H_2_O, CH_3_OH (solvent), N(CH_2_CH_2_OH)_3_, NaOH, H_2_O	pH: 9.5; duration: 150 s; microwave oven (900 W)	sword-like wires with diameters of about 80–250 nm and the length of ∼1–4 μm	[765]
Zn(C_5_H_7_O_2_)_2_·*x*H2O, CH_3_OH+C_6_H_13_OH (1-hexanol) (solvent)	*T*: 160 °C; duration: 1 h; microwave reactor	spherical aggregates of NPs, SSA: 52 m^2^/g	[766]
Zn(CH_3_COO)_2_·2H_2_O, C_2_H_4_(OH)_2_ (solvent), H_2_O (solvent), various volume ratio H_2_O:C_2_H_4_(OH)_2_	*T*: 200 °C; duration; 30 min; power: 1000 W; microwave reactor	rods, hexagonal prisms, peanut-like, butterfly-like, spheres	[767]
Zn(CH_3_COO)_2_·2H_2_O, Na_2_CO_3_, polyethylene glycol (PEG400) (solvent)	duration: 10 min; microwave oven (700 W)	nanorods, diameters: 10–25 nm, length: 60–200 nm	[768]
Zn(CH_3_COO)_2_·2H_2_O, C_4_H_8_(OH)_2_ (1,4- butanediol, solvent)	duration: 2 min with on–off mode with a duration interval of 30 sec; power: 200 W; microwave oven (800 W)	nanoparticles 59 ± 16 nm	[769]
Zn(C_5_H_7_O_2_)_2_ (zinc acetylacetonate), C_2_H_5_OH (solvent), polyvinylpyrrolidone (various concentrations)	duration: 15 min; power: 800 W; microwave oven	irregularly shaped nanoparticles, nanorods and nanotubes	[770]
Zn(C_5_H_7_O_2_)_2_ (zinc acetylacetonate), C_2_H_5_OH + H_2_O (solvent), cetyltrimethylammonium bromide (CTAB)	duration: 15 min; power: 100 W; microwave reactor	nanorods coatings deposited on Cr/Si, Al/Si, Au/Si, ITO/glass; nanorod (length > 1 μm, width ~140–180 nm)	[771]
Zn(C_5_H_7_O_2_)_2_·H_2_O (zinc acetylacetonate monohydrate), alkoxyethanol (solvent), methoxyethanol (solvent), ethoxyethanol (solvent), n-butoxyethanol (solvent),	duration: 4 min; power: 800 W; microwave oven	SSA: 10–70 m^2^/g, nanoparticles 30–200 nm	[772]
Zn(C_5_H_7_O_2_)_2_·*x*H2O (zinc acetylacetonate hydrate, various concentrations), C_4_H_9_OH (1-butanol, solvent)	*T*: 120 °C; duration: 30 min; power: 250 W; pulsed microwave irradiation in microwave reactor	spherical-shaped (diameter 10–60 nm) and rod-shaped structures (length 100 nm)	[773]
Zn(CH_3_COO)_2_·2H_2_O, NaOH, (CH_3_)_2_CHOH (isopropanol, solvent)	duration: 5 min; power: 150 W; microwave reactor	spherical nanoparticles (3–5 nm)	[774]
commercial ZnO was dissolved in 5 M NaOH, C_2_H_8_N_2,_ C_2_H_5_OH (solvent)	*T*: 150 °C; duration: 30 min; microwave reactor	flower-like microstructures, rod-like microstructures (length: 5 μm, diameter: 1 μm)	[775]
Zn(CH_3_COO)_2_·2H_2_O, C_3_H_6_(OH)_2_ (1,3-propanediol, solvent)	duration: 3 min with on–off mode having duration interval of 30 s; power: 360 W; microwave oven (800 W)	nanoparticles, diameter: 10–50 nm	[776]
Zn(CH_3_COO)_2_·2H_2_O, N(CH_2_CH_2_OH)_3_), CH_3_OH (solvent), H_2_O (solvent), NaOH	pH: 10.6–11.0; duration: 120–180 s; microwave oven (900 W)	uniform flower-like ZnO nanostructures (water), lengths: 700–950 nm, width: 130–230 nm; spheres (methanol): 250–400 nm	[573]
Zn(CH_3_COO)_2_·2H_2_O, N(CH_2_CH_2_OH)_3_, NaOH, C_4_H_9_OH (butanol, solvent)	pH: 8–10; *T*: 110 °C; duration: 40–60 s; microwave oven (900 W)	aggregated particles, average diameters: 550 nm; semi-spherical particles along with rice-like particles, average diameters ~600 nm; nanospheres average diameter 250 nm	[777]
Zn(NO_3_)_2_·6H_2_O, CH_3_(CH_2_)_15_NH_2_ (hexadecylamine), NaOH, C_2_H_5_OH (solvent), C_3_H_6_O (acetone, solvent), H_2_O (solvent)	*T*: 120 °C; duration: 15 min; microwave reactor	ethanol: spherical particles (20–60 nm) and rods with aspect ratio of 8–60; acetone: spherical particles (45–100 nm) and rods with aspect ratio which ranges from 37 to 94. water: star-shaped nanoparticles	[778]
Zn(C_5_H_7_O_2_)_2_·*x*H_2_O (zinc acetylacetonate hydrate), ethylenediamine (solvent), water (solvent), polyvinyl alcohol (PVA), polyethylene glycol 400 (PEG-400), polyvinylpyrrolidone (PVP), cetyltrimethylammonium bromide (CTAB)	*T*: 100–110 °C; duration: 10 min, microwave oven	ethylenediamine: flower with rod-like petals, nanorod, nanoflake, flower with rod-like petals, flower with rod-like petals; water: flower cluster with spindle-like petals	[779]
Zn(CH_3_COO)_2_·2H_2_O, C_2_H_5_OH (anhydrous solvent), C_6_H_5_CH_2_OH (benzyl alcohol, solvent), H_2_O (solvent)	*T*: 200 °C; duration: 5–30 min; microwave reactor	SSA: 32–34 m^2^/g; ethanol: nanoparticles (~20 nm), hollow spheres consisting of nanoparticles (20–30 nm) ranging from 200 to 700 nm in diameter; benzyl alcohol: nanoparticles (10–80 nm) water: nanorods, length: 1–2 µm	[780]
Zn(CH_3_COO)_2_·2H_2_O, NaOH, C_2_H_5_OH (solvent)	*T*: 60 °C; duration: 5–6 min; microwave oven	spherical nanoparticles (4–8 nm)	[781,782]
Zn(CH_3_COO)_2_·2H_2_O, (CH_3_)_2_CHOH (isopropanol, solvent)	duration: 180 s; power: 140 W; microwave oven	nanowires	[783]
Zn(CH_3_COO)_2_·2H_2_O, (CH_3_)_2_CHOH (isopropanol) + H_2_O (solvent, various concentrations)	duration: 1–3 min; microwave oven (1450 W)	spheroidal nanostructures	[784]
Zn(NO_3_)_2_·6H_2_O, NaOH (different concentrations), sodium dodecyl sulfate (SDS), C_2_H_5_OH + H_2_O (solvent)	*T*: 80–180 °C duration: 5–60 min; microwave reactor	rod-like, sheet-like, needle-like and flower-like nanostructures	[785]
Zn(NO_3_)_2_·6H_2_O, NaOH, C_2_H_5_OH + H_2_O (solvent), hexamethylene tetramine (C_6_H_12_N_4_), triethanolamine ((TEA, C_6_H_15_NO_3_, various concentrations)	*T*: 180 °C duration: 15 min; microwave reactor (600 W)	mulberry-like structures (~150 nm) was constructed by many nanoparticles (~5 nm); flower-like structures; hexagonal structure	[786]
Zn(CH_3_COO)_2_·2H_2_O, Zn(NO_3_)_2_·6H_2_O, zinc metal powder, NaOH, NH_4_(OH), C_2_H_5_OH (solvent), C_2_H_4_(OH)_2_ (solvent)	pH: 10–14; duration: 5–10 min: power: 300–900 W: reactor microwave	marigold-flower like, multipod jasmine- flower like, urchin-rod-flower like, calendula-flower like and rice-grain-shape like	[787]
Zn(CH_3_COO)_2_·2H_2_O, NaOH, H_2_O (solvent), C_2_H_4_(OH)_2_ (solvent), 2-ethoxyethanol (solvent), Triton X-100	duration: 3 min; power: 300 W; reactor microwave	nanorods with a pencil-like tip, nanorods with hexagonal flat tops, flower-like nanostructures	[788]
bis(acetylacetonato)zinc monohydrate, bis(methylacetato)zinc, bis(dimethylmalonato)zinc, C_2_H_3_N (acetonitrile, solvent)	*T*: 160–220 °C; duration: 30 min; power: 300 W; microwave reactor	spherical nanoparticles (3–16 nm)	[789]
Zn(CH_3_COO)_2_·2H_2_O, NaOH, C_2_H_5_OH (solvent), H_2_O	*T*: 90 °C; duration: 20 min; microwave reactor	spherical nanoparticles (20–25 nm)	[790]
Zn(CH_3_COO)_2_·2H_2_O, (CH_3_)_2_CHOH (isopropanol, solvent), (CH_2_CH_2_OH)_2_NH (diethanolamine)	*T*: 150–200 °C; microwave reactor	nanospheres	[791]
Zn(CH_3_COO)_2_·2H_2_O, C_4_H_9_NO (N,N-dimethylacetamide, DMAc, solvent), H_2_O	duration: 1.5–6 min; microwave oven (800 W)	particles (300–510 nm) and nanowires	[792]
Zn(CH_3_COO)_2_·2H_2_O, oleic acid, diethylene glycol (solvent)	*T*: 220–230 °C; duration: 10–15 min; microwave reactor	nanoparticles; diameters: 5–9 nm	[793]
Zn(NO_3_)_2_·6H_2_O, CO(NH_2_)_2_, H_2_O, C_2_H_4_(OH)_2_ (ethylene glycol, solvent)	*T*: 150 °C; duration: 15 min; microwave reactor (850 W)	flowers	[794]
ZnCl_2_·2H_2_O, sodium oleate, tetrabutylammonium hydroxide, tetrahydrofuran (C_4_H_8_O, THF, solvent)	*T*: 125–200 °C; duration: 5 min; microwave reactor	spherical nanoparticles, size: 2.6–3.8 nm	[795]
Zn(CH_3_COO)_2_·2H_2_O, Na_2_CO_3_, polyvinyl alcohol (PVA), polyethylene glycol (PEG), diethylene glycol (DEG, solvent),	duration: 1.5–5 min; microwave oven (1150 W)	nanoparticles, SSA: 35–86 m^2^/g	[796]
Zn(NO_3_)_2_·6H_2_O, NaOH, polyethylene glycol, C_2_H_5_OH (solvent)	*T*: 140 °C; duration: 10 min; microwave oven	rods, diameter: 0.167 ± 0.05 µm, length: 1.63 ± 0.33 µm	[797]
Zn(NO_3_)_2_·6H_2_O, NaOH, C_2_H_5_OH (solvent)	duration: 10–15 min; power 33%; microwave reactor (800 W)	nanorods, diameters: 10–20 nm	[705]

**Table 11:** Summary of the microwave solvothermal synthesis of ZnO nanocomposites or ZnO hybrid nanostructures without any additional heat treatment.

Type of Composite	Substrates	Conditions during Preparation	Properties	Ref.
Co doped ZnO	Zn(CH_3_COO)_2_·2H_2_O, Co(CH_3_COO)_2_·4H_2_O, C_2_H_4_(OH)_2_ (solvent)	*T*: 220 °C, duration: 25 min; power: 100%; microwave reactor (600 W)	Zn_1−*x*_Co*_x_*O (*x* = 0, 0.01, 0.05, 0.10 and 0.15); spherical nanoparticles: SSA: 37–39 m^2^/g, diameter: 30–32 nm, paramagnetic behaviour	[798,799]
Co doped ZnO	Zn(CH_3_COO)_2_·2H_2_O, Co(CH_3_COO)_2_·4H_2_O, C_2_H_4_(OH)_2_ (solvent), H_2_O (different concentrations)	*T*: 190 °C, duration: 25 min; power: 100%; microwave reactor (600 W)	Zn_0.90_Co_0.10_O, control of size of particles within the size range between circa 20 and 53 nm, SSA: 43–21 m^2^/g	[800]
Co doped ZnO	Zn(CH_3_COO)_2_·2H_2_O, Co(CH_3_COO)_2_·4H_2_O, oleic acid, (HOC_2_H_4_)_2_O (diethylene glycol, DEG, solvent)	*T*: 250 °C, duration: 15 min; power: 100%; microwave reactor	Zn_1−*x*_Co*_x_*O (*x* = 0, 0.01, 0.05, 0.10), spherical nanoparticles: diameter: 5–40 nm	[801]
Mn doped ZnO	Zn(CH_3_COO)_2_·2H_2_O, Mn(CH_3_COO)_2_·4H_2_O, C_2_H_4_(OH)_2_ (solvent)	*T*: 200 °C, duration: 25 min; power: 100%; microwave reactor (600 W)	Zn_1−*x*_Mn*_x_*O (*x* = 0, 0.01, 0.05, 0.10 0.15, 0.20, 0.25); spherical nanoparticles, diameter: 19–30 nm, SSA: 40–63 m^2^/g	[802]
Co-Mn co-doped ZnO	Zn(CH_3_COO)_2_·2H_2_O, Co(CH_3_COO)_2_·4H_2_O Mn(CH_3_COO)_2_·4H_2_O, C_2_H_4_(OH)_2_ (solvent)	*T*: 190 °C, duration: 25 min; power: 100%; microwave reactor (600 W)	Zn_(1−*x*−*y*)_Mn_x_Co_y_O NPs was *x* = *y* = 0.00, 0.01, 0.05, 0.10, 0.15 (the amount of both ions was equal), spherical nanoparticles, diameter: 19–30 nm, SSA: 40–56 m^2^/g, paramagnetic and ferromagnetic behaviour	[803]
M doped ZnO (M = Co, Cr, Fe, Mn, Ni)	Zn(NO_3_)_2_·6H_2_O, Co(NO_3_)_2_·6H_2_O, Cr(NO_3_)_3_·9H_2_O, Cr(NO_3_)_3_·9H_2_O, Mn(NO_3_)_2_·4H_2_O, Ni(NO_3_)_2_·6H_2_O, NaOH, C_2_H_5_(OH) (solvent), polyethylene glycol MW ≈ 2000	*T*: 280 °C; pressure: 20 bar; microwave reactor (300 W)	nanoparticles, paramagnetic behaviour	[232]
Mn doped ZnO	Zn(CH_3_COO)_2_·2H_2_O, Mn(CH_3_COO)_2_·4H_2_O, C_2_H_4_(OH)_2_ (solvent)	duration: 30 s cycles (on for 10 s, off for 20 s) for 10 min; power: 33%; microwave reactor (650 W)	Zn_1−*x*_Mn*_x_*O (*x* = 0, 0.05, 0.10, 0.20), nanoparticles	[804]
Ag-ZnO	Zn(CH_3_COO)_2_·2H_2_O, Ag(CH_3_COO), C_2_H_4_(OH)_2_ (solvent)	duration: 12 min; power: 1 kW (33%); microwave reactor (3 kW)	ZnO spherical nanoparticles (30–35 nm); Ag spherical nanoparticles (35–39 nm)	[181]
Ag-ZnO	Zn(CH_3_COO)_2_·2H_2_O, AgNO_3,_ C_2_H_4_(OH)_2_ (solvent), H_2_O	duration: 30 s cycles (on for 21 s, off for 9 s) for 10 min; power: 900 W; microwave oven	spherical nanoparticles (13–30 nm); hexagonal disks (14–165 nm); nanorods diameter: 104 nm, aspect ratio of 2.8; 3.5 nm Ag particles were inserted into the pores of ZnO; SSA: 25–51 m^2^/g	[805]
Ag-ZnO	Zn(CH_3_COO)_2_·2H_2_O, AgNO_3,_ C_2_H_4_(OH)_2_ (solvent), H_2_O	*T*: 170 °C; duration: 30 min; microwave oven	hierarchical architectures constructed by nanoparticles (50 nm)	[806]
Au-ZnO	Zn(CH_3_COO)_2,_ HAuCl_4_, oleic acid + oleylamine (solvent)	duration: 10 s–15 min; power: 100–1000 W; microwave oven	nanopyramids, height: 100–130 nm, diameter of the hexagonal basal plane of the final Au-ZnO nanopyramid is about 50–60 nm	[807]
Co doped ZnO	Zn(CH_3_COO)_2_·2H_2_O, Co(CH_3_COO)_2_·2H_2_O, C_2_H_4_(OH)_2_ (solvent)	*T*: 280 °C; pressure: 20 bar; duration 20 min; microwave reactor (300 W)	Zn_1−*x*_Co*_x_*O (*x* = 0, 0.001, 0.01; 0.05, 0.10, 0.15), nanoparticles	[808]
M doped ZnO (M = Mn, Ni, Co, Cr)	Zn(CH_3_COO)_2_·2H_2_O, Mn(CH_3_COO)_2_·4H_2_O, Ni(CH_3_COO)_2_·4H_2_O, Co(CH_3_COO)_2_·2H_2_O, Cr(CH_3_COO)_3_, C_2_H_4_(OH)_2_ (solvent)	*T*: 280 °C; pressure: 20 bar; duration 40 min; microwave reactor (300 W)	Zn_1−*x*_M*_x_*O (*x* = 0, 0.05, 0.10, 0.15), nanoparticles (20–30 nm)	[809]
M doped ZnO (M = V, Co, Fe, Ni, Mn)	Zn(CH_3_COO)_2_, Co(CH_3_COO)_2_, Fe(CH_3_COO)_2_, Ni(CH_3_COO)_2_·4H_2_O, Mn(CH_3_COO)_2_, C_6_H_5_CH_2_OH (benzyl alcohol, solvent)	*T*: 160 °C; duration: 3 min; microwave reactor	Zn_1−*x*_M*_x_*O (*x* = 0–0.3), nanoparticles (10–20 nm), Fe doped samples showed room temperature ferromagnetism	[810]
In doped ZnO, Al doped ZnO	Zn(CH_3_COO)_2_·2H_2_O, InCl_3_·4H_2_O, AlCl_3_·6H_2_O, diethylene glycol (solvent), H_2_O	*T*: 200 °C; duration 30 min; laboratory microwave oven (1200 W), 1 h at 400 °C in H_2_/N_2_ = 10/90%	nanoparticles (10–15 nm)	[811]
Co doped ZnO, Mn doped ZnO	Zn(NO_3_)_2_, Co(NO_3_)_2_, Mn(NO_3_)_2_, NaOH, C_2_H_5_(OH) (solvent), H_2_O	pH: 12; duration 5 min; power: 150 W; microwave oven	concentration of the dopant was 5%; spherical nanoparticles (10–15 nm), paramagnetic	[812]
Ni doped ZnO	Zn(CH_3_COO)_2_·2H_2_O, Ni(CH_3_COO)_2_·2H_2_O, NaOH, polyvinylpyrrolidone, (CH_3_)_2_CHOH (isopropanol, solvent)	duration 5 min; power: 150 W; microwave oven	ZnO:Ni nanorods with diameter: 8–10 nm and length: 35-45 nm	[813]
Al doped ZnO	Zn(CH_3_COO)_2_·2H_2_O, Al(NO_3_)_3_, NaOH, C_2_H_5_(OH) (solvent)	*T*: 80 °C; duration 60 min; power: 400 W; microwave oven	Al doping levels: 0, 1.0, 2.0, 3.0, 4.0 at%; spherical-like structures, crystallite size: 11–15 nm	[814]
Al. doped ZnO, Ga doped ZnO, Al, Ga co-doped ZnO	Zn(CH_3_COO)_2_·2H_2_O, Al(NO_3_)_3_·9H_2_O, Ga(NO_3_)_3_·*x*H_2_O, diethylene glycol (solvent), H_2_O	*T*: 200 °C; duration 30 min; microwave reactor (1500 W)	Al and Ga dopant levels were from 0.5 to 2.5 at%; doped and co-doped powders exhibited a broad size distribution with particles around 100–200 nm	[815]
Al_2_O_3_ coated ZnO	ZnO NPs (diameter: 12–25 nm, 38.4 m^2^/g), aluminium triisopropoxide (Al(O-i-Pr)3), NH_4_OH, C_2_H_5_(OH) (solvent)	pH: 12; *T*: 70 °C; duration 5 min; microwave reactor (500 W)	core-shell structures	[816]
Mg doped ZnO	Zn(NO_3_)_2_·6H_2_O, Mg(NO_3_)_2_·6H_2_O, CO(NH_2_)_2_, urea, C_2_H_4_(OH)_2_ (solvent), H_2_O	*T*: 130 °C; duration 4 h; power: 150–200 W; microwave reactor	Zn_1−*x*_Mg*_x_*O (*x* = 0, 0.2, 0.4, 0.6, 0.8), nano- and sub-micron particle size	[817]
Fe_3_O_4_@SiO_2_/ZnO	Fe_3_O_4_@SiO_2_ (different quantities), Zn(CH_3_COO)_2_·2H_2_O, diethylene glycol (DEG, solvent)	*T*: 160 °C; duration 15–60 h; mechanical stirring	Fe_3_O_4_ content: 10–30 wt%; spherical shape, size 250–850 nm	[818]
coaxial ZnO/C/CdS nanocables	ZnO/C core-shell nanocables (80 nm in diameter and a range of 0.5–2 mm in length), CdCl_2_·2H_2_O, C_2_H_5_(OH) (solvent), thioacetamide	duration 10 min; power: 280 W; microwave refluxing system	CdS nanoparticles (5.5 nm) uniformly deposited on the surface of nanocables	[819]
ZnO/reduced graphene oxide	graphite (modified Hummers method and Fan’s method), Zn(CH_3_COO)_2_·2H_2_O (various concentrations), diethylene glycol (solvent)	duration 10 min; power: 300 W; microwave refluxing system	ZnO nanocrystals (10–14 nm) anchored onto reduced graphene oxide sheets	[820]
ZnO/reduced graphene oxide, Ag/ZnO/reduced graphene oxide	graphite, Zn(CH_3_COO)_2_·2H_2_O (various concentrations), AgNO_3_, NaOH, C_2_H_4_(OH)_2_	duration: 4 cycles (heated 1 min, stirred for 3 min); microwave oven	ZnO NPs and Ag NPs anchored onto reduced graphene oxide sheets	[821]
C/ZnO	ZnO nanorods grafted by glucose, glycerol	*T*: 100 °C; duration 30 min; microwave reactor	carbon-coated ZnO nanorods	[822]

**Table 12:** Summary of the microwave solvothermal synthesis of ZnO nanocomposites or ZnO hybrid nanostructures with additional heat treatment.

Type of Composite	Substrates	Conditions during Preparation	Calcination Parameters	Properties	Ref.
undoped ZnO	Zn(CH_3_COO)_2_·2H_2_O, NaOH, isopropanol (solvent)	duration 5 min; microwave oven	600 °C in air for 1 h	spherical nanoparticles (30 nm)	[823]
undoped ZnO	Zn(CH_3_COO)_2_·2H_2_O, NaOH, 1-butyl-3-methylimidazolium chloride (solvent)	duration 1 min; power: 400–1000 W; microwave oven	500 °C in air for 3 h	nanoparticles; size from 15–25 to 50–70 nm	[824]
undoped ZnO	Zn(C_5_H_7_O_2_)_2_·*x*H_2_O, C_2_H_5_(OH) (solvent), CH_3_OH (solvent), polyvinylpyrrolidone (PVP), cetyltrimethylammonium bromide (CTAB), and Triton X100, H_2_O	duration: 20 s–5 min; power: 160–800 W; microwave oven (800 W) with a water-cooled condenser (reflux system)	500 °C in air for 2–5 min	films on Si, Ge, Cr/Si, glass, indium tin oxide coated glass and polymer substrates, (spherical nanoparticles (diameter: ∼15) nm or nanorods (length: 1–3 μm))	[825,826,827,828,829,830]
undoped ZnO	Zn(CH_3_COO)_2_·2H_2_O, NaOH, dimethylformamide (DMF)	duration 60 min; power: 300 W; microwave oven	500 °C in air for 3 h	spherical nanoparticles (as-synthesised 24–26 nm, annealed 33–34 nm)	[831]
undoped ZnO	Zn(NO_3_)_2_·6H_2_O, 2-methylimidazole, HCOONa, CH_3_OH (solvent)	*T*: 120 °C; duration: 2 h; microwave reactor	550 °C in air for 2 h	uniform particle approximately rhombic dodecahedron facets, size: ~97 nm	[832]
Co doped ZnO	Zn(CH_3_COO)_2_·2H_2_O, Co(CH_3_COO)_2_·2H_2_O, urea, C_2_H_4_(OH)_2_ (solvent)	duration 30 min; power: 500 W; microwave oven	400 °C in air	Zn_1−*x*_Co*_x_*O (*x* = 0.1, 0.2, 0.3, 0.4), nanoparticles (~24 nm), paramagnetic behaviour	[833]
Co doped ZnO	Zn(CH_3_COO)_2_·2H_2_O, Co(CH_3_COO)_2_·4H_2_O, C_2_H_4_(OH)_2_ (solvent)	*T*: 220 °C, duration: 25 min; power: 100%; microwave reactor (600 W)	800 °C in nitrogen for 0.5 h, 800 °C in synthetic air for 0.5 h	Zn_1−*x*_Co*_x_*O (*x* = 0, 0.01, 0.05, 0.10, 0.15); spherical nanoparticles: SSA: 3 m^2^/g, diameter: 300–400 nm, paramagnetic behaviour	[798]
Co doped ZnO	Zn(CH_3_COO)_2_·2H_2_O, Co(CH_3_COO)_2_·2H_2_O, NaOH, HCl, C_2_H_4_(OH)_2_ (solvent)	pH: 6–12; duration: until microwave heating solvents were evaporated; power: 500 W; microwave oven	400 °C in air	Zn_0.94_Co_0.06_O; nanoparticles (7–23 nm), paramagnetic behaviour	[834]
Co doped ZnO	Zn(CH_3_COO)_2_·2H_2_O, Co(CH_3_COO)_2_·2H_2_O, urea, C_2_H_4_(OH)_2_ (solvent)	until microwave heating solvents were evaporated; microwave oven	300 °C in air for 1 h	Zn_1−*x*_Co*_x_*O (*x* = 0.001-0.004), average crystallite size 18–28 nm; super paramagnetic nature and ferromagnetic behaviour	[835]
Fe doped ZnO	Zn(CH_3_COO)_2_·2H_2_O, Fe(NO_3_)_2_·9H_2_O, NaOH, polyvinylpyrrolidone (PVP), C_2_H_5_OH (solvent)	pH: 11; duration 2 min; power: 140 W; microwave oven	200 °C in air for 1 h	nanoparticles (11–17 nm), Fe content: 0–20%	[836]
Mn doped ZnO	Zn(CH_3_COO)_2_·2H_2_O, Mn(CH_3_COO)_2_·4H_2_O, C_2_H_4_(OH)_2_ (solvent)	duration: 30 s cycles for 20 min in total; power: 650 W; microwave oven	400 °C in air	Zn_1−*x*_Mn*_x_*O (*x* = 0.1–0.4), spherical nanoparticles	[837]
Ag-ZnO	Zn(CH_3_COO)_2_·2H_2_O, AgNO_3_, Na_2_O_2_, isopropanol (solvent), cetyltrimethylammonium bromide (CTAB)	*T*: 200 °C; duration 2–6 h; microwave reactor	300 °C in air for 2 h	chrysanthemum-like prismatic nanorods	[838]

**Table 13:** Summary of synthesis of pure ZnO by the microwave hybrid method.

Type of Method	Substrates	Conditions during Preparation	Calcination Parameters	Properties	Ref.
Ultrasonic microwave synthesis	Zn(CH_3_COO)_2_·2H_2_O, ZnCl_2_·2H_2_O, 2-[4-(2-hydroxyethyl)-1-piperazinyl]ethanesulfonic acid (HEPES) (different concentrations), H_2_O	pH: 5.4–9.4; T: 110 °C; duration: 17 min (discontinuous ultrasonic irradiation (1 s sonication and 2 s interruption); power: 500 W; microwave and ultrasonic wave combined reactor	-	half-backed grenade-like ZnO microstructures, uniform spindle-like ZnO microstructures; spindle-like to double-prism-like structures	[844]
Ultraviolet assisted - ultrasonic microwave synthesis	Zn(CH_3_COO)_2_·2H_2_O, C_16_H_33_(CH_3_)_3_NBr (cetyltrimethylammonium bromide, CTAB), (CH_2_)_6_N_4_ (hexamethylenetetramine, HMT), H_2_O	*T*: 98 °C; duration: 10–25 min; power: 150 W; UV- microwave and ultrasonic wave combined reactor	-	hourglass-like ZnO microstructures (diameter: 1 µm, length: 2 µm)	[845]
Microwave induced combustion synthesis	ZnSO_4_, oxalic acid, polyvinyl alcohol (fuel)	duration: 0–30 min; power level: 0–90%; microwave oven	-	nanoparticles: crystallite size: ~33 nm	[846]
Microwave induced combustion process	Zn(NO_3_)_2_·6H_2_O, urea (fuel), H_2_O	power: 170–680 W; microwave oven	-	flower-like microstructures (2–5 μm) and irregular block-shaped particles (100–300 nm)	[847]
Microwave induced combustion process	Zn(NO_3_)_2_·6H_2_O, urea (fuel), H_2_O	duration: 10 min; microwave oven (700 W)		spherical nanoparticles (10–100 nm)	[848]
Microwave induced combustion process	Zn(NO_3_)_2_·6H_2_O, urea (fuel), H_2_O	duration: 20 min; microwave oven (800 W)		nanoplatelets	[849]
Microwave induced combustion process	Zn(NO_3_)_2_·6H_2_O, C_2_H_4_(OH)_2_ (fuel)	microwave oven (800 W)	-	foamy and porous structures	[850]
Microwave induced combustion process	Zn(NO_3_)_2_·6H_2_O, glycine (fuel)	duration: 2 min; power: 80%; microwave oven	-	nanoparticles (20–25 nm)	[851]
Microwave induced combustion process	Zn(NO_3_)_2_·6H_2_O, using citric acid (C_6_H_8_O_7_) as a fuel, NH_4_OH, H_2_O	pH: 4; duration: 2 min; power: 80%; microwave oven	800 °C for 2 h in air	rods	[852]
Microwave induced combustion process	Zn(NO_3_)_2_·6H_2_O, glycine (fuel), H_2_O	duration: 10 min; microwave oven (750 W)	-	nanoflakes	[853]
Microwave induced combustion process	Zn(CH_3_COO)_2_·2H_2_O, H_2_O	duration: 6 min; power: 800 W; microwave oven	-	powder	[854]
Microwave induced combustion process	Zn(NO_3_)_2_·6H_2_O, Indian bael (Aegle marmelos) juice - different volumes (fuel)	duration: 10 min; microwave oven	500 °C for 1 h in air	heterogeneous particles, sheet like structures,	[855]
Microwave assisted annealing	Zn(OH)_2_, NH_4_(OH)	*T*: 140–320 °C, duration: 30 min; microwave reactor	-	layers on SiO_2_	[856]
Sol-gel -microwave assisted annealing	Zn(CH_3_COO)_2_·2H_2_, 2-methoxyethanol (ME), monoethanolamine (MEA),	*T*: 140–320 °C, duration: 3–12 min; power: 65 W; microwave oven (750 W)	-	layers (40 nm) on indium-tin oxide cathodes	[857]
Microwave assisted annealing	Zn(NO_3_)_2_·6H_2_O, H_2_O	*T*: 250 °C, duration: 3 min; microwave oven (3 kW)	-	layers on SnO_2_	[858]
Microwave assisted annealing	Zn(NO_3_)_2_·6H_2_O, extract (fruit, seed or pulp), H_2_O	duration: 8 min; power: 340 W; microwave oven	-	flower-like, hexagonal, and block-shaped nanostructures, size: 27–85	[859]
Microwave assisted annealing	Zn(NO_3_)_2_·6H_2_O, cetyltrimethylammonium bromide (CTAB), hexamethylenetetramine – (HMTA) (different concentrations), H_2_O	duration: 30, 60 min; microwave oven	-	nanopowders	[860]
Microwave assisted annealing	Zn(NO_3_)_2_·6H_2_O, PEG400 (polyethylene glycol, M = 400), NaOH, H_2_O, C_2_H_5_(OH)	duration: 10 × 10 min and 4 × 10 min with on–off mode with a duration interval of 1 min; power: 136–800 W; microwave oven (800 W)	500 °C in air for 0.5 h	sphere particles (10–50 nm) and rods (lengths: 50–200 nm, diameters: 15–50 nm)	[861]
Sol-gel – microwave assisted annealing	Zn(NO_3_)_2_·6H_2_O, HNO_3_ (65%), H_2_O	duration: 30–40 min; microwave oven	-	SSA: 0.12–0.2 m^2^/g, corn-like microstructures	[862]
Sol-gel - microwave assisted annealing	Zn(CH_3_COO)_2_·2H_2_O, C_2_H_4_(OH)_2_ (solvent)	pH: 6; duration: 10 min; power: 600 W; microwave oven	450 °C in air for 2 h	nanoparticles (average crystallite size 24 nm)	[863]
Sol-gel - microwave assisted annealing	Zn(CH_3_COO)_2_·2H_2_O, LiOH·H_2_O, C_2_H_5_OH (solvent)	*T*: 30–60 °C; duration: 10–40 min; microwave reactor (600 W)	80 °C in air for 2 h	quantum-sized particles 4–7 nm	[864]
Microwave assisted sintering	ZnO, organic compounds	duration: 10–30 min; microwave oven (900 W)	-	thick films on alumina substrate, heterogeneous microstructures	[865]
Microwave assisted sintering	ZnO powder, carbon charcoal powder	duration: 20–60 min; microwave oven (1000 W)	-	ZnO nanoclusters, size from 8 μm to 10 μm	[866]
Microwave sintering	ZnO powder, graphite, O_2_	duration: 3 min; power: 100–1200 W; microwave oven; oxygen flow	-	nanowires, diameters: 2–70 nm, length: 5–15 µm	[867]
Microwave sintering	ZnO nano and micropowders, acetone	*T*: 1100–1350 °C; duration: 40 min; power 5 kW;	-	hexagonal tubes, diameter: 10 µm, length: 100 µm, wall thickness: 0.5–1 µm	[868]
Microwave vapour deposition	ZnO nanoparticles (20–30 nm; 48.9 m^2^/g) )	*T*: 1350–1400 °C; duration: 15 min; power: 3850 W; traveling-wave mode microwave system (5000 W)	-	microtubes (hexagonal hollow), average diameter: 60 µm, length: 250 µm; wall thickness: 3–5 µm	[869,870]
Microwave vapour deposition	Zn powder, steel-wool	duration: 30 s; microwave oven (1000 W)	-	nanofiber: diameters 50 nm, lengths 0.5–1 μm	[871]
Microwave vapour deposition	Zn powder, steel-wool, O_2_/N_2_ = 20/80, 40/60, 60/40, 80/20	microwave oven (1000 W)	-	nanoparticles with controlled morphologies	[872]
Microwave vapour deposition	Zn(CH_3_COO)_2_·2H_2_O, C_2_H_5_OH, vacuum of 5 kPa, O_2_	power: 400–1200 W; microwave based plasma deposition unit (2000 W)	-	films (NPs diameter: 10–18 nm) deposited on glass substrates	[873]
Microwave vapour deposition	metallic Zn flakes (2–3 mm)	duration: <5 min; microwave oven (800 W)	-	nanowires: diameter 70–80 nm	[874]
Microwave vapour deposition	ZnO microtubes (wall thickness less than 0.5–1)	*T*: 800–1450 °C; microwave system (3000 W)	-	single-crystal microtubes rods (above 1300 °C); crystal rods (below 1150 °C, diameters from 50 nm to a few μm)	[875]
Microwave vapour deposition	ZnO, Zn, graphite, N_2_ (carrier gas, 25 cm^3^)	*T*: 1100 °C; duration: 3–5 min microwave system (3000 W)	-	nanowires, nanobelts and microrods on different substrates materials (sapphire, silicon carbide, polycrystalline alumina); micro/nanotubes up 150 mm, wall thickness 0.3–1 mm, length up to 2–4 mm	[876]
Microwave vapour deposition	ZnO, graphite	duration: 2 min; microwave oven (800 W)	-	nanosheets: widths several to tens of µm thickness 20–80 nm	[877]
Microwave plasma assisted chemical vapour deposition	ZnO powder, graphite powder, He (carrier gas)	duration: 5 min.; microwave oven (1000 W)	-	nanonails: size of caps: 25–150 nm, necks: 30–200 nm, shank: 250–450 nm	[878]
Microwave vapour deposition	Zn(CH_3_COO)_2_·2H_2_O, C_2_H_5_(OH)	duration: 70 s; carrier gas: air and O_2_; microwave plasma system (700 W)	-	film like, worm-like, flower like and dot-like structures on glass, silicon wafer and Al_2_O_3_/Si wafer substrates	[879]
Microwave vapour deposition	Zn, air	duration: 4 s; carrier gas: air; microwave plasma system	-	nanowires deposited on aluminium foil substrate, glass, paper, microfibre, polycarbonate film, paraffin wax	[880]
Microwave vapour deposition	Zn, H_2_O	*P*: 20 kPa; power: 135 W; microwave plasma system	-	flower-like structures composed of nanorods (diameter 50 nm, lengths 150–200 nm)	[881]
Microwave vapour deposition	Zn(CH_3_COO)_2_·2H_2_O, Zn(NO_3_)_2_·6H_2_O, ZnCl_2_·2H_2_O, ethanol	duration: 10–120 s; protective gas and carrier gas: pure Ar (300 cm^3^/g); microwave plasma system	-	glass quadratic slides were coated by ZnO and/or Zn particles, whose sizes ranged from a few micrometres to ∼20 nm	[882]
Microwave plasma	Zn powder (10 µm)	protective gas and carrier gas: air, O_2_, O_2_/N_2_ (20/80 vol%); flow rate 10 l/m; microwave plasma system	-	wires (diameter: 111 nm, length: 5835 nm), tetrapods (diameter: 30 nm, length: 257 nm), rods (diameter: 82–627 nm, length: 309–853 nm), tetrapods (diameter: 30 nm, length: 257 nm)	[883]
Microwave vapour deposition	Zn(CH_3_COO)_2_·2H_2_O, NaOH, H_2_O, atmospheric pressure	pH: 11; power: 800 W; home-made microwave induced plasma in liquid system (1.5 kW)	-	NPs; diameter: 23 nm	[884]
Microwave assisted ball milling	Zn(CH_3_COO)_2_·2H_2_O	duration: 4–20 h; power: 800 W; microwave reactor	-	nanoparticles, diameters: 15 nm	[885]
-	1 cm × 1 cm × 125 μm Zn sheet, gas mixture of O_2_ (20 sccm) and Ar (80 sccm)	duration: 3 s; microwave oven (1000 W)	-	growth of nanoneedles on the Zn sheet; length ~500 nm, tip ~40, pillar: ~100	[886]

**Table 14:** Summary of the microwave hybrid synthesis method of ZnO nanocomposites or ZnO hybrid nanostructures.

Type of Product	Type of Method	Substrates	Conditions during Preparation	Calcination Parameters	Properties	Ref.
Ag/ZnO, Au/ZnO, ZnO	ultrasonic microwave synthesis - UV irradiation	Zn(CH_3_COO)_2_·2H_2_O, NaOH, H_2_O, HAuCl_4_ - C_2_H_5_(OH) (solvent) AgNO_3_ - C_2_H_5_(OH) (solvent)	duration: 30 min (combined discontinuous ultrasonic irradiation (1 s sonication and 2 s interruption); power: 500 W; microwave and ultrasonic wave combined reactor	-	flower-like ZnO nanostructures (~800 nm)	[887]
Ag/ZnO, ZnO	ultrasonic microwave synthesis and deposition-precipitation	Zn(CH_3_COO)_2_·2H_2_O (0.5 M), urea (0.001 M), cetyltrimethylammonium bromide (CTAB_ (0.00035 M), C_2_H_4_(OH)_2_ (solvent) AgNO_3_ (10 M), NaOH (1 M)	duration: 20 min (combined discontinuous ultrasonic irradiation (1 s sonication and 2 s interruption); power: 500 W; microwave and ultrasonic wave combined reactor	-	spindle-like micro-and nanostructures	[888]
Ag/ZnO, Ag/ZnO/graphene, ZnO	ultrasonic microwave synthesis	graphite oxide (modified Hummers method), Zn(NO_3_)_2_·6H_2_O, AgNO_3_, hexamethylenetetramine [HMT, (CH_2_)_6_N_4_], H_2_O	*T*: 90 °C, duration: 2 h, ultrasonic microwave reaction system (700 W)	-	ZnO: rods, Ag/ZnO: rods with nanoparticles Ag/ZnO/graphene: rods with nanoparticles and sheets	[889]
Al doped ZnO	microwave induced combustion process	Zn(NO_3_)_2_·6H_2_O, Al(NO_3_)_3_·9H_2_O, C_2_H_4_(OH)_2_ (fuel)	duration: 10 min with on/off cycles of 30/10 s; power: 300 W; microwave oven	-	0 to 7% atomic weight percentage Al doped ZnO, hexagonal shaped, diameter: 20–53 nm	[890]
Al doped ZnO/Sn doped In_2_O_3_	microwave plasma assisted chemical vapour deposition	In_2_O_3_:SnO_2_ = 90:10 wt%, ZnO:Al_2_O_3_ = 98:2 wt%, gas pressure: 25 torr, hydrogen flow rate: 100 sccm	duration: 5 min; power: 400–800 W; microwave plasma system	-	films on glass substrates	[891]
Au/Fe_2_O_3_-ZnO	microwave assisted annealing	ZnCl_2_·5H_2_O, HAuCl_4_, FeSO_3_∙7H_2_O, NaOH, H_2_O	power: 700 W	400 °C for 4 h in air	nanostructures	[892]
Ba doped ZnO	microwave assisted annealing	Zn(NO_3_)_2_·6H_2_O, BaCl_2_, NaOH, H_2_O	*T*: 500 °C, duration: 30 min; microwave oven	-	doping range of 0 to 2 at%; nanoparticles (crystallite sizes: 20–22 nm)	[893]
Ce–Cu co-doped ZnO	microwave induced combustion process	Zn(NO_3_)_2_·6H_2_O, Ce(NO_3_)_2_·6H_2_O, Cu(NO_3_)_2_·6H_2_O, H_2_O, using urea as a fuel	duration: 20 min; power: 800 W; microwave oven	600 °C in air for 2 h	Zn_1−2*x*_Ce*_x_*Cu*_x_*O (*x* = 0.00, 0.01, 0.02, 0.03, 0.04 and 0.05), crystallite size: 35–46 nm, heterogeneous shape	[894]
Co doped ZnO	microwave induced combustion process	Zn(NO_3_)_2_·6H_2_O, Co(NO_3_)_2_·6H_2_O, using citric acid (C_6_H_8_O_7_) as a fuel (solution obtained was heated up to 80-90 °C until the excess water was removed and a highly viscous precursor gel was gained)	*T*: 900 and 1000 °C; duration: 50 s: power level: 100%; microwave oven (900 W)	-	Zn_0.90_Co_0.10_O, micron particle size	[895]
Co doped ZnO	microwave sintered	Co doped ZnO from (Zn(CH_3_COO)_2_·2H_2_O, Co(CH_3_COO)_2_·4H_2_O, C_2_H_5_OH)	*T*: 900–1075 °C duration: 15 min microwave furnace (850 W)	-	doping range of 0 to 7 mol%, powders, crystallite size: 36–210 nm	[896]
Cr doped ZnO	microwave induced combustion process	Zn(NO_3_)_2_·6H_2_O, Cr_2_(SO_4_)_3_·6H_2_O (various concentrations), C_2_H_4_(OH)_2_ (fuel)	duration: 8 min; power 210 W; microwave oven (420 W)	500 °C in air	Cr doped ZnO (0.00≤ *x* ≤0.15), heterogeneous nano/microstructures	[897]
ZnO/BiOBr ZnO	ultrasonic microwave synthesis	Bi(NO_3_)_3_·5H_2_O, KBr, Zn(CH_3_COO)_2_·2H_2_O, NH_4_OH, C_2_H_5_OH	pH: 8–9; T: 85 °C; duration: 35 min (discontinuous ultrasonic irradiation (2 s sonication and 1 s interruption); power: 500 W; microwave and ultrasonic wave combined reactor	-	ZnO/BiOBr grown on cotton fabric	[898]
Zn–ZnO, ZnO	microwave plasma	Zn powder and bulk ZnO, H_2_, Ar, O_2_	duration: 15–30 min; protective gas and carrier gas: pure H_2_; microwave plasma system (400 W)	-	nanowire-nanocable-nanotube route has been designed to fabricate ZnO nanotubes with desired dimensions	[899]
Zn–ZnO ZnO	microwave plasma	Zn(CH_3_COO)_2_·2H_2_O, H_2_O, air (0.3 L/min)	gases: Ar (flow rate 10 L/m) N_2_ (flow rate 5 L/m); power: 650 W; microwave plasma system	400 °C for 1 h in air	ZnO/Zn films on glass substrate	[900]
ZnO–ZrO_2_, ZnO	microwave induced combustion process	Zn(NO_3_)_2_·6H_2_O, ZrO(NO_3_)_2_·*x*H_2_O, urea (fuel), H_2_O	duration: 7 min; power: 850 W; microwave oven	-	heterogeneous particles, ZnO-ZrO_2_ M ratio: 1-1, 2-1, 1-2	[901]
ZnAl_2_O_4_/ZnO, ZnO	microwave induced combustion process	Zn(NO_3_)_2_·6H_2_O, Al(NO3)3·9H2O, urea (fuel), H_2_O	duration: 1–2 min; power: 900 W; microwave oven	1000 °C for 1 h in air	ZnAl_2_O_4_/ZnO nanocomposites with different ZnO (20, 30, and 40 mol%); flakes and plates structures of fine particles (ZnAl_2_O_4_ diameter 55 nm; ZnO diameter 38 nm)	[902,903]
Cu doped ZnO	microwave induced combustion process	Zn(NO_3_)_2_·6H_2_O, Cu(CH_3_COO)_2_·2H_2_O, C_2_H_4_(OH)_2_	duration: 10 min; power: 320 W; microwave oven (800 W)	450–750 °C	ZnO micro/nanostructures, wt% of Cu: 0, 0.8, 1.6, 2.5 and 5%	[904]
Cu–ZnO–Al_2_O_3_	microwave induced combustion process	Zn(NO_3_)_2_·6H_2_O, Cu(NO_3_)_2_·3H_2_O, Al(NO_3_)_3_·9H_2_O, H_2_O, using C_2_H_4_(OH)_2_ as a fuel(Cu, Zn and Al nitrates with 3/6/1 molar ratio)	microwave oven	-	nanoparticles	[905]
Cu–ZnO, ZnO	microwave assisted annealing	ZnSO_4_·7H_2_O (0.1 M), CuSO_4_·5H_2_O (0.1 M) NaOH (0.2 M), ascorbic acid, H_2_O	microwave oven	-	heterogeneous structures	[906]
Eu doped ZnO	microwave assisted annealing	Zn(CH_3_COO)_2_·2H_2_O, Eu(NO_3_)_3_·5H_2_O, NaOH, polyvinyl alcohol, H_2_O	pH: 10; duration: 5 min with on-off cycle (20 s on - 40 s off); microwave oven (600 W)	200 °C for 3 h in air	doping range of 0 to 0.5 mol%, nanorods	[907]
Eu doped ZnO	microwave induced gel combustion process	Zn(NO_3_)_2_·6H_2_O, Eu_2_O_3_, HNO_3_, using citric acid (C_6_H_8_O_7_) and glycine (NH_2_CH_2_COOH) as a fuel (different fuel mixtures); (solution obtained was heated up to 80 °C for 1 h until the excess water was removed and a highly viscous precursor gel was gained)	duration: 50 s, microwave oven	900 °C for 1 h in air	Zn_0.99_Eu_0.01_O nanoparticles; diameter: 30 nm	[908]
Fe_2_O_3_/ZnO	microwave sintering (solid state reaction)	ZnO powders (1 µm), γ-Fe_2_O_3_ (20–40 nm)	*T*: 1000–1400 °C; duration: different; power: 3 kW and 4 kW; microwave system (2.45 GHz and 28 GHz)	-	Fe_2_O_3_(ZnO)*_x_* (*x* = 6, 8, 34); homogeneous micro- and nanostructures	[909]
Ga doped ZnO	ultrasonic microwave synthesis	Zn(NO_3_)_2_·6H_2_O, Ga(NO_3_)_3_·*x*H_2_O, NaOH	*T*: 140 °C, duration: 30 min, ultrasonic microwave reaction system (150 W)	-	nanoparticles, Ga mol% content: 1, 2, 3, 5 and 10%)	[910]
Ga doped ZnO	microwave assisted annealing	Zn(CH_3_COO)_2_·2H_2_O, Ga(NO_3_)_3_·*x*H_2_O, NaOH, H_2_O	*T*: 150 °C, duration: 5 min, microwave reactor (4 × 800 W)	-	Zn_1−*x*_Ga*_x_*O (*x* = 0, 0.01, 0.02, 0.03), heterogeneous nanoparticles	[911]
In_2_O_3_-Ga_2_O_3_-ZnO	microwave assisted annealing	Zn(CH_3_COO)_2_·2H_2_O, Ga(NO3)3·*x*H2O, In(NO_3_)_3_·*x*H2O, mono-ethanolamine (C_2_H_7_NO), methoxyethanol (solvent) (various reactant concentrations)	duration: 2 min; power: 150–1400 W; microwave annealing system	600 °C for 0.5 h in air	thin films	[912]
In_2_O_3_-Ga_2_O_3_-ZnO	post treatment process microwave assisted annealing	In_2_O_3_-Ga_2_O_3_-ZnO (layer is deposited by atmospheric pressure plasma enhanced chemical vapour deposition (AP-PECVD))	duration: 50/100 s; power: 150/300 W; microwave oven	-	thin films on silicon wafer	[913]
M and Co co-doped ZnO	microwave induced combustion process	Mn(NO_3_)_2_·4H_2_O, Co(NO_3_)_2_·6H_2_O, Zn(NO_3_)_2_·6H_2_O, Fe(NO_3_)_3_·9H_2_O, Ni(NO_3_)_2_·6H_2_O, water, using urea as a fuel	duration: 20 min microwave oven (1000 W)		M_0.1_Co_0.1_Zn_0.8_O (M = Cu, Fe, Mn, Ni); nanoparticles (25–34 nm)	[914]
Mg doped ZnO	microwave induced combustion process	Zn(NO_3_)_2_·6H_2_O, Mg(NO_3_)_2_·6H_2_O (various concentrations), vera plant extract (fuel), H_2_O	duration: 10 min; microwave oven (800 W)	-	0 and 1.5 wt% Mg doped ZnO	[915]
MgO doped ZnO	microwave sintered	ZnO, MgO powders used with the same purity and particle size of (99.9%, 20–30 nm)	*T*: 1400–1470 °C; duration: 20 min; power: 1300–1500 W; microwave reactor (5000 W)	-	microtubes, lengths of up to several micrometres in the ranges between 150 and 200 µm and an average diameter of 70 µm	[916]
Mn and Co co-doped ZnO	microwave induced combustion process	Mn(NO_3_)_2_·4H_2_O, Co(NO_3_)_2_·6H_2_O, Zn(NO_3_)_2_·6H_2_O, water, using urea as a fuel	duration: 15 min microwave oven (800 W)	-	Mn*_x_*Co_0.1_Zn_0.9−*x*_O (*x* = 0.0, 0.05, 0.1, 0.15, and 0.2); nanoparticles (24–33 nm)	[917]
Sb_2_O_3_-MnO-CoO-Cr_2_O_3_-ZnO	microwave sintering	ZnO nanopowder (35 nm), Sb_2_O_3_, MnO, CoO, Cr_2_O_3_	*T*: 1000–1150 °C; duration: 1–60 min; microwave oven (2000 W)	-	average particle size: 2.1 µm	[918]
Sm doped ZnO	sol-gel-ultrasonic microwave sintering	Zn(NO_3_)_2_·6H_2_O, Sm(NO_3_)_3_·6H_2_O, polyvinylpyrrolidone (PVP, MW ≈ 40,000), diethylene glycol (DEG), triethylenetetramine (TETA), NaOH, H_2_O	pH: 9; duration: 7 min; power: 700 W; microwave reactor	400 °C for 2 h in air	nanorods, lengths: 200–400 nm, widths: 50–90 nm	[919]
Ti*_x_*O*_y_*–ZnO	microwave sintering(solid state reaction)	ZnO powder (1 µm), Ti powder (53 µm)	*T*: 550–1100 °C; duration: 1 min; microwave sintering furnace	-	rods (lengths: 1.5–3 µm, diameters: 0.2–0.5 µm) and coarser and irregularly shaped ZnO whiskers	[920]
ZnO/ZnFe_2_O_4_	microwave induced combustion process	Zn(NO_3_)_2_·6H_2_O, Fe(NO_3_)_2_·9H_2_O, NaCH_3_COO (different concentrations) using polyethylene glycol as a fuel	duration: 5 and 10 min; power: 120 and 700 W; microwave oven	-	nanoparticles, size in range from 23–54 nm (SSA = 94.5 m^2^/g) to 102–209 nm (SSA = 59.5 m^2^/g)	[921]
ZnO/multi-walled carbon nanotube	microwave assisted annealing	Zn(CH_3_COO)_2_·2H_2_O, multi-walled carbon nanotubes, (diameter about 20–50 nm), H_2_O	power: 300–700 W; microwave oven	-	nanocomposites	[922]
ZnO–exfoliated graphene	microwave assisted annealing	graphite (modified Staudenmaiers method), Zn(NO_3_)_2_·6H_2_O, NH_4_OH, H_2_O	duration: 5 min; power: 700 W; microwave oven	-	wrinkles and a fluff-like structure	[923]
ZnO–reduced graphene oxide	microwave assisted annealing	graphite (modified Hummers method), Zn(CH_3_COO)_2_·2H_2_O, H_2_O	duration: 5 min; power: 1000 W; microwave reactor	-	reduced graphene oxide sheets with ZnO nanoparticles (10–20 nm), SSA = 109.5 m^2^/g; ZnO NPs diameter range of 50 to 100 nm	[924]
ZnO–graphene oxide	microwave assisted annealing	graphite (modified Staudenmaiers method), Zn(NO_3_)_2_·6H_2_O, NaOH, C_2_H_5_OH, H_2_O	duration: 3–7.5 min; power: 800 W; microwave oven	-	ZnO microcubes-graphene oxide; ZnO nanoflakes-graphene oxide; ZnO nanoneedles-graphene oxide	[925]
ZnO–Pr_2_O_3_–CoO–Cr_2_O_3_–K_2_O	microwave induced combustion process	ZnO (96.7 mol%), Pr_2_O_3_ (2 mol%), CoO (0.5 mol%), Cr_2_O_3_ (0.5 mol%), K_2_O (0.3 mol%); agate mortar in presence of n-hexane as a fuel	duration: 8 min; power: 600 W; microwave oven	1200, 1250 and 1350 °C for 2 h in air	Agglomerated particles, size 4–6 µm	[926]
expandable graphite–ZnO	microwave sintered	exfoliated graphite, ZnO nanopowder	duration: 5 min; power: 1000 W; microwave oven	-	graphite-ZnO nanocomposite powders	[927]

## References

[1] Drexler E., Peterson C., Pergamit G. (1991). Unbounding the Future: The Nanotechnology Revolution.

[2] Lloyd’s Register Foundation (2014). Foresight Review of Nanotechnology, the Next Industrial Revolution.

[3] Shrivastava S., Dash D. (2009). Applying nanotechnology to human health: Revolution in biomedical sciences. J. Nanotechnol..

[4] Stirling D.A. (2018). The Nanotechnology Revolution: A Global Bibliographic Perspective.

[5] Lowry G.V., Avellan A., Gilbertson L.M. (2019). Opportunities and challenges for nanotechnology in the agri-tech revolution. Nat. Nanotechnol..

[6] Durkan C. (2019). Size Really Does Matter: The Nanotechnology Revolution.

[7] McClintock P.V.E. (2019). Size really does matter: The nanotechnology revolution. Contemp. Phys..

[8] Wolf E.L., Medikonda M. (2012). Understanding the Nanotechnology Revolution.

[9] Nasrollahzadeh M., Sajadi S.M., Sajjadi M., Issaabadi Z. (2019). An introduction to nanotechnology. Interface Sci. Technol..

[10] Wennersten R., Fidler J., Spitsyna A., Misra K.B. (2008). Nanotechnology: A New Technological Revolution in the 21st Century. Handbook of Performability Engineering.

[11] Vance M.E., Kuiken T., Vejerano E.P., McGinnis S.P., Hochella M.F., Rejeski D., Hull M.S. (2015). Nanotechnology in the real world: Redeveloping the nanomaterial consumer products inventory. Beilstein J. Nanotechnol..

[12] Sanders W.C. (2018). Basic Principles of Nanotechnology.

[13] García-Martínez J., Wang Z.L. (2013). Nanotechnology for the Energy Challenge.

[14] Raj B., Van de Voorde M., Mahajan Y. (2017). Nanotechnology for Energy Sustainability.

[15] Fakruddin M., Hossain Z., Afroz H. (2012). Prospects and applications of nanobiotechnology: A medical perspective. J. Nanobiotechnol..

[16] Ramos A.P., Cruz M.A.E., Tovani C.B., Ciancaglini P. (2017). Biomedical applications of nanotechnology. Biophys. Rev..

[17] Kumar A., Gupta K., Dixit S., Mishra K., Srivastavaet S. (2019). A review on positive and negative impacts of nanotechnology in agriculture. Int. J. Environ. Sci. Technol..

[18] Prasad R., Bhattacharyya A., Nguyen Q.D. (2017). Nanotechnology in sustainable agriculture: Recent developments, challenges, and perspectives. Front. Microbiol..

[19] Cheraghian G., Hendraningrat L. (2016). A review on applications of nanotechnology in the enhanced oil recovery part A: Effects of nanoparticles on interfacial tension. Int. Nano Lett..

[20] Hannah W., Thompson P.B. (2008). Nanotechnology, risk and the environment: A review. J. Environ. Monit..

[21] Kumar S., Nehra M., Kedia D., Dilbaghi N., Tankeshwar K., Kim K.-H. (2020). Nanotechnology-based biomaterials for orthopaedic applications: Recent advances and future prospects. Mater. Sci. Eng. C.

[22] King T., Osmond-McLeod M.J., Duffy L.L. (2018). Nanotechnology in the food sector and potential applications for the poultry industry. Trends Food Sci. Technol..

[23] He X., Deng H., Hwang H.M. (2019). The current application of nanotechnology in food and agriculture. J. Food Drug Anal..

[24] Gothandam K., Ranjan S., Dasgupta N., Ramalingam C., Lichtfouse E. (2018). Nanotechnology, Food Security and Water Treatment.

[25] AlKahtani R.N. (2018). The implications and applications of nanotechnology in dentistry: A review. Saudi Dent. J..

[26] Saeedi M., Eslamifar M., Khezri K., Dizaj S.M. (2019). Applications of nanotechnology in drug delivery to the central nervous system. Biomed. Pharmacother..

[27] Sanchez F., Sobolev K. (2010). Nanotechnology in concrete—A review. Constr. Build. Mater..

[28] Keskinbora K.H., Jameel M.A. (2018). Nanotechnology applications and approaches in medicine: A review. J. Nanosci. Nanotechnol. Res..

[29] Krishna V.D., Wu K., Su D., Cheeran M.C.J., Wang J.P., Perez A. (2018). Nanotechnology: Review of concepts and potential application of sensing platforms in food safety. Food Microbiol..

[30] Bayford R., Rademacher T., Roitt I., Wang S.X. (2017). Emerging applications of nanotechnology for diagnosis and therapy of disease: A review. Physiol. Meas..

[31] Kaul S., Gulati N., Verma D., Mukherjee S., Nagaich U. (2018). Role of nanotechnology in cosmeceuticals: A review of recent advances. J. Pharm..

[32] Schulte J., Dutta J. (2005). Nanotechnology in environmental protection and pollution. Sci. Technol. Adv. Mater..

[33] Matteucci F., Giannantonio R., Calabi F., Agostiano A., Gigli G., Rossi M. (2018). Deployment and exploitation of nanotechnology nanomaterials and nanomedicine. AIP Conf. Proc..

[34] Jaishree V., Gupta P.D. (2012). Nanotechnology: A revolution in cancer diagnosis. Ind. J. Clin. Biochem..

[35] Contera S. (2019). Nano Comes to Life: How Nanotechnology is Transforming Medicine and the Future of Biology.

[36] Kumar A., Pandey A.N., Jain S.K. (2016). Nasal-nanotechnology: Revolution for efficient therapeutics delivery. Drug Deliv..

[37] Silva G.A. (2010). Nanotechnology applications and approaches for neuroregeneration and drug delivery to the central nervous system. Ann. N. Y. Acad. Sci..

[38] Agrawal S., Rathore P. (2014). Nanotechnology pros and cons to agriculture: A review. Int. J. Curr. Microbiol. Appl. Sci..

[39] (2015). International Organisation for Standarization ISO/TS 80004-2:2015.

[40] Burda C., Chen X., Narayanan R., El-Sayed M.A. (2005). Chemistry and properties of nanocrystals of different shapes. Chem. Ver..

[41] Rao C.N.R., Kulkarni G.U., Thomas P.J., Edwards P.P. (2002). Size-dependent chemistry: Properties of nanocrystals. Chemistry.

[42] Uglov V.V., Doroshevich I.L., Kvasov N.T., Remnev G.E., Shymanski V.I. (2016). On physical properties of nanoparticles: Size effect and scale of nanoobjects. Phys. Status Solidi C.

[43] Guisbiers G., Mejía-Rosales S., Deepak F.L. (2012). Nanomaterial properties: Size and shape dependencies. J. Nanomater..

[44] Prabha S., Arya G., Chandra R., Ahmed B., Nimesh S. (2016). Effect of size on biological properties of nanoparticles employed in gene delivery. Artif. Cells Nanomed. Biotechnol..

[45] Mohanraj V.J., Chen Y. (2006). Nanoparticles—A review. Trop. J. Pharm. Res..

[46] Roduner E. (2006). Size matters: Why nanomaterials are different. Chem. Soc. Rev..

[47] Lundqvist M., Stigler J., Elia G., Lynch I., Cedervall T., Dawson K.A. (2008). Nanoparticle size and surface properties determine the protein corona with possible implications for biological impacts. Proc. Natl. Acad. Sci. USA.

[48] Rodríguez-López J.L., Montejano-Carrizales J.M., Palomares-Báez J.P., Barrón-Escobar H., Velázquez-Salazar J.J., Cabrera-Trujillo J.M., José-Yacamán M. (2010). Size effect and shape stability of nanoparticles. Key Eng. Mater..

[49] Heera P., Shanmugam S. (2015). Nanoparticle characterization and application: An overview. Int. J. Curr. Microbiol. Appl. Sci..

[50] Albanese A., Tang P.S., Chan W.C.W. (2012). The effect of nanoparticle size, shape, and surface chemistry on biological systems. Annu. Rev. Biomed. Eng..

[51] Klabunde K.J. (2001). Nanoscale Materials in Chemistry.

[52] Yaghmaee M.S., Shokri B., Rahimipour M.R. (2009). Size dependence surface activity of metallic nanoparticles. Plasma Process. Polym..

[53] Ramsden J. (2012). Nanotechnology for military applications. Nanotechnol. Percept..

[54] Shafique M., Luo X. (2019). Nanotechnology in transportation vehicles: An overview of its applications, environmental, health and safety concerns. Materials.

[55] Foltynowicz Z., Czajka B., Maranda A., Wachowski L. (2017). Aspects of nanomaterials for civil and military applications Part 1. The origin, characterization and methods of obtaining. Mater. Wysokoenergetyczne/High. Energy Mater..

[56] Foltynowicz Z., Czajka B., Maranda A., Wachowski L. (2017). Aspects of nanomaterials for civil and military applications. Part 2. The use of and concerns arising from infiltration of the natural environment. Mater. Wysokoenergetyczne/High. Energy Mater..

[57] Abd Elkodous M., El-Sayyad G.S., Abdelrahman I.Y., El-Bastawisy H.S., Mohamed A.E., Mosallam F.M., Nassere H.A., Gobaraf M., Barakaf A., Elsayedf M.A. (2019). Therapeutic and diagnostic potential of nanomaterials for enhanced biomedical applications. Colloids Surf. B.

[58] Nasrollahzadeh M., Sajadi S.M., Mohaddeseh S., Issaabadi Z. (2019). Applications of nanotechnology in daily life. Interface Sci. Technol..

[59] Zare-Zardini H., Ferdowsian F., Soltaninejad H., Ghorani Azam A., Soleymani S., Zare-Shehneh M., Mofidi M., Rafati R., Ebrahimi L. (2016). Application of nanotechnology in biomedicine: A major focus on cancer therapy. J. Nano Res..

[60] Jędrzak A., Grześkowiak B.F., Coy E., Wojnarowicz J., Szutkowski K., Jurg S., Jesionowski T., Mrówczyński R. (2019). Dendrimer based theranostic nanostructures for combined chemo- and photothermal therapy of liver cancer cells in vitro. Colloids Surf. B.

[61] Mrówczyński R., Jędrzak A., Szutkowski K., Grześkowiak B.F., Coy E., Markiewicz R., Jesionowski T., Jurga S. (2018). Cyclodextrin-based magnetic nanoparticles for cancer therapy. Nanomaterials.

[62] AbouAitah K., Hassan H.A., Swiderska-Sroda A., Gohar L., Shaker O.G., Wojnarowicz J., Opalinska A., Smalc-Koziorowska J., Gierlotka S., Lojkowski W. (2020). Targeted nano-drug delivery of colchicine against colon cancer cells by means of mesoporous silica nanoparticles. Cancers.

[63] Pietrzykowska E., Mukhovskyi R., Chodara A.A., Wojnarowicz J., Koltsov I., Chudoba T., Łojkowski W. (2019). Composites of polylactide and nano-hydroxyapatite created by cryomilling and warm isostatic pressing for bone implants applications. Mater. Lett..

[64] Rogowska-Tylman J., Locs J., Salma I., Woźniak B., Pilmane M., Zalite V., Wojnarowicz J., Kędzierska-Sar A., Chudoba T., Szlązak K. (2019). In vivo and in vitro study of a novel nanohydroxyapatite sonocoated scaffolds for enhanced bone regeneration. Mater. Sci. Eng. C.

[65] Pokrowiecki R., Zareba T., Mielczarek A., Opalińska A., Wojnarowicz J., Majkowski M., Lojkowski W., Tyski S. (2013). Evaluation of biocidal properties of silver nanoparticles against cariogenic bacteria. Med. Dosw. Mikrobiol..

[66] Werengowska-Ciećwierz K., Wiśniewski M., Terzyk A.P., Furmaniak S. (2015). The chemistry of bioconjugation in nanoparticles-based drug delivery system. Adv. Cond. Matter Phys..

[67] Heath J.R. (2015). Nanotechnologies for biomedical science and translational medicine. Proc. Natl. Acad. Sci. USA.

[68] Jeevanandam J., Barhoum A., Chan Y.S., Dufresne A., Danquah M.K. (2018). Review on nanoparticles and nanostructured materials: History, sources, toxicity and regulations. Beilstein J. Nanotechnol..

[69] Khan I., Saeed K., Khan I. (2019). Nanoparticles: Properties, applications and toxicities. Arab. J. Chem..

[70] Ali A., Phull A.R., Zia M. (2018). Elemental zinc to zinc nanoparticles: Is ZnO NPs crucial for life? Synthesis, toxicological and environmental concerns. Nanotechnol. Rev..

[71] Willander M. (2014). Zinc Oxide Nanostructures: Advances and Applications.

[72] Fortunato E., Gonçalves A., Pimentel A., Barquinha P., Gonçalves G., Pereira L., Ferreira I., Martins R. (2009). Zinc oxide, a multifunctional material: From material to device applications. Appl. Phys. A.

[73] Xu S., Wang Z.L. (2011). One-dimensional ZnO nanostructures: Solution growth and functional properties. Nano Res..

[74] Morkoç H., Özgür Ü. (2009). Zinc Oxide: Fundamentals, Materials and Device Technology.

[75] Borysiewicz M.A. (2019). ZnO as a functional material, a review. Crystals.

[76] Kołodziejczak-Radzimska A., Jesionowski T. (2014). Zinc oxide—From synthesis to application: A review. Materials.

[77] Roberts W.L., Campbell T.J., Rapp G.R. (1990). Encyclopedia of Minerals.

[78] Ashrafi A., Jagadish C. (2007). Review of zincblende ZnO: Stability of metastable ZnO phases. J. Appl. Phys..

[79] Janotti A., Van de Walle C.G. (2009). Fundamentals of zinc oxide as a semiconductor. Rep. Prog. Phys..

[80] Litton C.W., Reynolds D.C., Collins T.C. (2011). Zinc Oxide Materials for Electronic and Optoelectronic Device Applications.

[81] Liu H., Yang D., Yang H., Zhang H., Zhang W., Fang Y., Liu Z., Tian L., Lin B., Yan J. (2013). Comparative study of respiratory tract immune toxicity induced by three sterilization nanoparticles: Silver, zinc oxide and titanium oxide. J. Hazard. Mater..

[82] Mirhosseini M., Firouzabadi F. (2012). Antibacterial activity of zinc oxide nanoparticle suspensions on food-borne pathogens. Int. J. Dairy Technol..

[83] Frederickson C.J., Koh J.Y., Bush A.I. (2005). The neurobiology of zinc in health and disease. Nat. Rev. Neurosci..

[84] Craddock P.T., Gurjar L.K., Hegde K.T.M. (1983). Zinc production in medieval india. World Archaeol..

[85] Halioua B., Ziskind B. (2005). Medicine in the Days of the Pharaohs.

[86] Moezzi A., McDonagh A.M., Cortie M.B. (2012). Zinc oxide particles: Synthesis, properties and applications. Chem. Eng. J..

[87] Schmalz G., Arenholt-Bindslev D. (2009). Biocompatibility of Dental Materials.

[88] Barja-Fidalgo F., Moutinho-Ribeiro M., Oliveira M.A.A., Heloísa de Oliveira B.H. (2011). A systematic review of root canal filling materials for deciduous teeth: Is there an alternative for zinc oxide-eugenol?. Int. Sch. Res. Not..

[89] Trckova M., Lorencova A., Hazova K., Sramkova Zajacova Z. (2015). Prophylaxis of post-weaning diarrhoea in piglets by zinc oxide and sodium humate. Vet. Med. (Praha.).

[90] Long L., Chen J., Zhang Y., Liang X., Ni H., Zhang B., Yin Y. (2017). Comparison of porous and nano zinc oxide for replacing high-dose dietary regular zinc oxide in weaning piglets. PLoS ONE.

[91] Fraga C.G. (2005). Relevance, essentiality and toxicity of trace elements in human health. Mol. Asp. Med..

[92] Mońka I., Wiechuła D. (2017). Importance of zinc for the human body in the aspect of zinc supplementation. Ann. Acad. Med. Siles..

[93] Hernandezbattez A., Gonzalez R., Viesca J., Fernandez J., Diazfernandez J., MacHado A., Chou R., Riba J. (2008). CuO, ZrO_2_ and ZnO nanoparticles as antiwear additive in oillubricants. Wear.

[94] Sturdy L.F., Wright M.S., Yee A., Casadio F., Faber K.T., Kenneth R., Shull K.R. (2020). Effects of zinc oxide filler on the curing and mechanical response of alkyd coatings. Polymer.

[95] Heideman G., Datta R.N., Noordermeer J.W.M., van Baarle B. (2004). Activators in accelerated sulfur vulcanization. Rubber Chem. Technol..

[96] Know the True Facts and Benefits of Zinc Oxide. www.uizincoxide.com/know-true-facts-benefits-zinc-oxide.

[97] Morkoç H., Özgür Ü., Morkoç H., Özgür U. (2009). General properties of ZnO. Zinc Oxide: Fundamentals, Materials and Device Technology.

[98] Umar A., Hahn Y.-B. (2010). Metal Oxide Nanostructures and Their Applications.

[99] Pearton S.J., Norton D.P., Ip K., Heo Y.W., Steiner T. (2003). Recent progress in processing and properties of ZnO. Superlattice Microst..

[100] Pearton S.J., Norton D.P., Ip K., Heo Y.W., Steiner T. (2005). Recent progress in processing and properties of ZnO. Prog. Mater. Sci..

[101] Heo Y.W., Pearton S.J., Norton D.P., Ren F., Ren F., Pearton S.J. (2017). ZnO thin-film and nanowire based sensor apllications. Semiconductor Device-Based Sensors for Gas, Chemical, and Biomedical Applications.

[102] Birnboim A., Gershon D., Calame J., Birman A., Carmel Y., Rodgers J., Levush B., Bykov Y.V., Eremeev A.G., Holoptsev V.V. (1998). Comparative study of microwave sintering of zinc oxide at 2.45, 30, and 83 GHz. J. Am. Ceram. Soc..

[103] Singh S., Thiyagarajan P., Mohan Kant K., Anita D., Thirupathiah S., Rama N., Tiwari B., Kottaisamy M., Ramachandra Rao M.S. (2007). Structure, microstructure and physical properties of ZnO based materials in various forms: Bulk, thin film and nano. J. Phys. D Appl. Phys..

[104] David R.L. (2005). CRC Handbook of Chemistry and Physics.

[105] Khan A. Synthesis, Characterization and Luminescence Properties of Zinc Oxide Nanostructures. https://search.proquest.com/docview/305294746.

[106] Jagadish C., Pearton S. (2006). Zinc Oxide Bulk, Thin Films and Nanostructures, Processing, Properties and Applications.

[107] Ashcroft N.W., Mermin N.D. (1976). Solid State Physics.

[108] Klingshirn C.F., Waag A., Hoffmann A., Geurts J. (2010). Zinc Oxide.

[109] Theerthagiri J., Salla S., Senthil R.A., Nithyadharseni P., Madankumar A., Arunachalam P., Maiyalagan T., Kim H.-S. (2019). A review on ZnO nanostructured materials: Energy, environmental and biological applications. Nanotechnology.

[110] Ealias A.E., Saravanakumar M.P. (2017). A review on the classification, characterisation, synthesis of nanoparticles and their application. IOP Conf. Ser. Mater. Sci. Eng..

[111] Nikam A.V., Prasad B.L.V., Kulkarni A.A. (2018). Wet chemical synthesis of metal oxide nanoparticles: A review. CrystEngComm.

[112] Mourdikoudis S., Pallares R.M., Thanh N.T.K. (2018). Characterization techniques for nanoparticles: Comparison and complementarity upon studying nanoparticle properties. Nanoscale.

[113] Mansfield E., Kaiser D.L., Fujita D., Van de Voorde M. (2017). Metrology and Standardization of Nanotechnology: Protocols and Industrial Innovations.

[114] Schilling K., Bradford B., Castelli D., Dufour E., Nash J.F., Wolfgang Pape W., Schulte S., Tooley I., van den Bosch J., Schellauf F. (2010). Human safety review of “nano” titanium dioxide and zinc oxide. Photochem. Photobiol. Sci..

[115] Future Markets, Inc. (2015). The Global Market for Zinc Oxide Nanoparticles.

[116] Newman M.D., Stotland M., Ellis J.I. (2009). The safety of nanosized particles in titanium dioxide- and zinc oxide-based sunscreens. J. Am. Acad. Dermatol..

[117] Schneider S.L., Lim H.W. (2019). A review of inorganic UV filters zinc oxide and titanium dioxide. Photodermatol. Photoimmunol. Photomed..

[118] Ajdary M., Moosavi M.A., Rahmati M., Falahati M., Mahboubi M., Mandegary A., Jangjoo S., Mohammadinejad R., Varma R.S. (2018). Health concerns of various nanoparticles: A review of their in vitro and in vivo toxicity. Nanomaterials.

[119] Smijs T.G., Pavel S. (2011). Titanium dioxide and zinc oxide nanoparticles in sunscreens: Focus on their safety and effectiveness. Nanotechnol. Sci. Appl..

[120] Lewicka Z.A., Benedetto A.F., Benoit D.N., Yu W.W., Fortner J.D., Colvin V.L. (2011). The structure, composition, and dimensions of TiO_2_ and ZnO nanomaterials in commercial sunscreens. Nanopart. Res..

[121] Mohammed Y.H., Holmes A., Haridass I.N., Sanchez W.Y., Studier H., Grice J.E., Benson H.A.E., Roberts M.S. (2019). Support for the safe use of zinc oxide nanoparticle sunscreens: Lack of skin penetration or cellular toxicity after repeated application in volunteers. J. Investig. Dermatol..

[122] (2013). The Global Market for Metal. Oxide Nanoparticles to 2020.

[123] Listewnik P., Hirsch M., Struk P., Weber M., Bechelany M., Jędrzejewska-Szczerska M. (2019). Preparation and characterization of microsphere ZnO ALD coating dedicated for the fiber-optic refractive index sensor. Nanomaterials.

[124] Barbillon G. (2019). Fabrication and SERS performances of metal/Si and metal/ZnO nanosensors: A review. Coatings.

[125] Djurišić A.B., Ng A.M.C., Chen X.Y. (2010). ZnO nanostructures for optoelectronics: Material properties and device applications. Prog. Quantum Electron..

[126] Boscarino S., Filice S., Sciuto A., Libertino S., Scuderi M., Galati C., Scalese S. (2019). Investigation of ZnO-decorated CNTs for UV Light Detection Applications. Nanomaterials.

[127] Chen C., Zhou P., Wang N., Ma Y., San H. (2018). UV-assisted photochemical synthesis of reduced graphene oxide/ZnO nanowires composite for photoresponse enhancement in UV photodetectors. Nanomaterials.

[128] Look D.C. (2001). Recent advances in ZnO materials and devices. Mater. Sci. Eng. B-Adv..

[129] Bhati V.S., Hojamberdiev M., Kumar M. (2019). Enhanced sensing performance of ZnO nanostructures-based gas sensors: A review. Energy Rep..

[130] Praveenkumar S., Manikandan S., Lingaraja D., Sugapriya T. (2018). A review of doped and undoped ZnO nanoparticles for fabrication of gas sensor. Sens. Lett..

[131] Zhu L., Zeng W. (2017). Room-temperature gas sensing of ZnO-based gas sensor: A review. Sens. Actuators A-Phys..

[132] Weber M., Kim J.-Y., Lee J.-H., Kim J.-H., Iatsunskyi I., Coy E., Miele P., Bechelany M., Sub Kim S. (2019). Highly efficient hydrogen sensors based on Pd nanoparticles supported on boron nitride coated ZnO nanowires. J. Mater. Chem. A.

[133] Park S., Lee D., Kwak B., Lee H.-S., Lee S., Yoo B. (2018). Synthesis of self-bridged ZnO nanowires and their humidity sensing properties. Sens. Actuators B-Chem..

[134] Kumar R., Al-Dossary O., Kumar G., Umar A. (2015). Zinc oxide nanostructures for NO_2_ gas–sensor applications: A review. Nano-Micro Lett..

[135] Hjiri M., El Mir L., Leonardi S.G., Donato N., Neri G. (2013). CO and NO_2_ selective monitoring by ZnO-based sensors. Nanomaterials.

[136] Procek M., Stolarczyk A., Pustelny T. (2017). Impact of temperature and UV irradiation on dynamics of NO_2_ sensors based on ZnO nanostructures. Nanomaterials.

[137] Wu C.-H., Jiang G.-J., Chang K.-W., Deng Z.-Y., Li Y.-N., Chen K.-L., Jeng C.-C. (2018). Analysis of the sensing properties of a highly stable and reproducible ozone gas sensor based on amorphous In-Ga-Zn-O thin film. Sensors.

[138] Shewale P.S., Yu Y.S., Kim J.H., Bobade C.R., Uplane M.D. (2015). H_2_S gas sensitive Sn-doped ZnO thin films: Synthesis and characterization. J. Anal. Appl. Pyrol..

[139] Gupta S.K., Joshi A., Kaur M. (2010). Development of gas sensors using ZnO nanostructures. J. Chem. Sci..

[140] Kanaparthi S., Singh S.G. (2019). Chemiresistive sensor based on zinc oxide nanoflakes for CO_2_ detection. ACS Appl. Nano Mater..

[141] Wanga Y., Meng X., Yao M., Sun G., Zhan Z. (2019). Enhanced CH_4_ sensing properties of Pd modified ZnO nanosheets. Ceram. Int..

[142] Shinde V.R., Gujar T.P., Lokhande C.D., Mane R.S., Han S.-H. (2007). Development of morphological dependent chemically deposited nanocrystalline ZnO films for liquefied petroleum gas (LPG) sensor. Sens. Actuators B-Chem..

[143] Dighavkar C. (2013). Characterization of nanosized zinc oxide based ammonia gas sensor. Arch. Appl. Sci. Res..

[144] Du H., Li X., Yao P., Wang J., Sun Y., Dong L. (2018). Zinc oxide coated tin oxide nanofibers for improved selective acetone sensing. Nanomaterials.

[145] Choi H., Kwon S.H., Kang H., Kim J.H., Choi W. (2020). Zinc-oxide-deposited carbon nanowalls for acetone sensing. Thin Solid Films.

[146] Zhou Q., Hong C., Yao Y., Ibrahim A.M., Xu L., Kumar R., Talballa S.M., Kim S.H., Umar A. (2017). Fabrication and characterization of highly sensitive acetone chemical sensor based on ZnO nanoballs. Materials.

[147] Sholehah A., Faroz D.F., Huda N., Utari L., Septiani N.L.W., Yuliarto B. (2020). Synthesis of ZnO flakes on flexible substrate and its application on ethylene sensing at room temperature. Chemosensors.

[148] Pronin I., Yakushova N., Averin I., Karmanov A., Moshnikov V., Dimitrov D. (2019). Investigation of gas-sensitive properties of thin-film thermovoltaic sensor elements based on zinc oxide. Coatings.

[149] Lu Y., Hsieh C., Su G. (2019). The Role of ALD-ZnO seed layers in the growth of ZnO nanorods for hydrogen sensing. Micromachines.

[150] Calestani D. (2019). ZnO nanostructures for gas sensing applications: From tetrapods-based chemoresistive devices to carbon fiber integration. Proceedings.

[151] Ocola L.E., Wang Y., Divan R., Chen J. (2019). Multifunctional UV and gas sensors based on vertically nanostructured zinc oxide: Volume versus surface effect. Sensors.

[152] Rai P., Kwak W.-K., Yu Y.-T. (2013). Solvothermal synthesis of ZnO nanostructures and their morphology-dependent gas-sensing properties. ACS Appl. Mater. Interfaces.

[153] Kwoka M., Lyson-Sypien B., Kulis A., Maslyk M., Borysiewicz M.A., Kaminska E., Szuber J. (2018). Surface properties of nanostructured, porous ZnO thin films prepared by direct current reactive magnetron sputtering. Materials.

[154] Zhang Y.H., Mei Z.X., Liang H.L., Du X.L. (2017). Review of flexible and transparent thin-film transistors based on zinc oxide and related materials. Chin. Phys. B.

[155] Rong P., Ren S., Yu Q. (2019). Fabrications and applications of ZnO nanomaterials in flexible functional devices—A review. Crit. Rev. Anal. Chem..

[156] Yoshida T., Komatsu D., Shimokawa N., Minoura H. (2004). Mechanism of cathodic electrodeposition of zinc oxide thin films from aqueous zinc nitrate baths. Thin Solid Films.

[157] Wang F., Liu R., Pan A., Cao l., Cheng K., Xue B., Wang G., Meng Q., Li J., Li Q. (2007). The optical properties of ZnO sheets electrodeposited on ITO glass. Mater. Lett..

[158] Gao X.D., Li X.M., Yu W.D. (2005). Rapid preparation, characterization, and photoluminescence of ZnO films by a novel chemical method. Mater. Res. Bull..

[159] Du X.Y., Fu Y.Q., Tan S.C., Luo J.K., Flewitt A.J., Milne W.I., Lee D.S., Park N.M., Park J., Choi Y.J. (2008). ZnO film thickness effect on surface acoustic wave modes and acoustic streaming. Appl. Phys. Lett..

[160] Shinde V.R., Gujar T.P., Lokhande C.D. (2007). Studies on growth of ZnO thin films by a novel chemical method. Sol. Energy Mater. Sol. Cells.

[161] Daksh D., Agrawal Y.K. (2016). Rare earth-doped zinc oxide nanostructures: A review. Rev. Nanosci. Nanotechnol..

[162] Sirelkhatim A., Mahmud S., Seeni S., Kaus A.S.N., Ann L.C., Bakhori S.K.M., Hasan H., Mohamad D. (2015). Review on zinc oxide nanoparticles: Antibacterial activity and toxicity mechanism. Nano-Micro Lett..

[163] Sun Q., Li J., Le T. (2018). Zinc oxide nanoparticle as a novel class of antifungal agents: Current advances and future perspectives. J. Agric. Food Chem..

[164] Siddiqi K.S., ur Rahman A., Tajuddin, Husen A. (2018). Properties of zinc oxide nanoparticles and their activity against microbes. Nanoscale Res. Lett..

[165] Oprea O., Andronescu E., Ficai D., Ficai A., Oktar F.N., Yetmez M. (2014). ZnO applications and challenges. Curr. Org. Chem..

[166] Martínez-Carmona M., Gun’ko Y., Vallet-Regí M. (2018). ZnO nanostructures for drug delivery and theranostic applications. Nanomaterials.

[167] Jiang J., Pi J., Cai J. (2018). The advancing of zinc oxide nanoparticles for biomedical applications. Bioinorg. Chem. Appl..

[168] Zhang Y., Nayak T.R., Hong H., Cai W. (2013). Biomedical applications of zinc oxide nanomaterials. Curr. Mol. Med..

[169] Jin S.-E., Jin H.-E. (2019). Synthesis, characterization, and three-dimensional structure generation of zinc oxide-based nanomedicine for biomedical applications. Pharmaceutics.

[170] Mohd Yusof H., Mohamad R., Zaidan U.H., Abdul Rahman N.A. (2019). Microbial synthesis of zinc oxide nanoparticles and their potential application as an antimicrobial agent and a feed supplement in animal industry: A review. J. Anim. Sci. Biotechnol..

[171] Król A., Pomastowski P., Rafińska K., Railean-Plugaru V., Buszewski B. (2017). Zinc oxide nanoparticles: Synthesis, antiseptic activity and toxicity mechanism. Adv. Colloid Interface Sci..

[172] Król A., Railean-Plugaru V., Pomastowski P., Buszewski B. (2019). Phytochemical investigation of Medicago sativa L. extract and its potential as a safe source for the synthesis of ZnO nanoparticles: The proposed mechanism of formation and antimicrobial activity. Phytochem. Lett..

[173] Das S., Chakraborty T. (2018). A review on green synthesis of silver nanoparticle and zinc oxide nanoparticle from different plants extract and their antibacterial activity against multi-drug resistant bacteria. J. Innov. Pharm. Biol. Sci..

[174] Kalpana V.N., Rajeswari V.D. (2018). A review on green synthesis, biomedical applications, and toxicity studies of ZnO NPs. Bioinorg Chem. Appl..

[175] Cierech M., Kolenda A., Grudniak A.M., Wojnarowicz J., Woźniak B., Gołaś M., Swoboda-Kopeć E., Łojkowski W., Mierzwińska-Nastalska E. (2016). Significance of polymethylmethacrylate (PMMA) modification by zinc oxide nanoparticles for fungal biofilm formation. Int. J. Pharm..

[176] Cierech M., Wojnarowicz J., Szmigiel D., Bączkowski B., Grudniak A., Wolska K., Łojkowski W., Mierzwińska-Nastalska E. (2016). Preparation and characterization of ZnO-PMMA resin nanocomposites for denture bases. Acta Bioeng. Biomech..

[177] Cierech M., Osica I., Kolenda A., Wojnarowicz J., Szmigiel D., Łojkowski W., Kurzydłowski K., Ariga K., Mierzwińska-Nastalska E. (2018). Mechanical and physicochemical properties of newly formed ZnO-PMMA nanocomposites for denture bases. Nanomaterials.

[178] Cierech M., Wojnarowicz J., Kolenda A., Krawczyk-Balska A., Prochwicz E., Woźniak B., Łojkowski W., Mierzwińska-Nastalska E. (2019). Zinc oxide nanoparticles cytotoxicity and release from newly formed PMMA–ZnO nanocomposites designed for denture bases. Nanomaterials.

[179] Sruthi S., Ashtami J., Mohanan P.V. (2018). Biomedical application and hidden toxicity of zinc oxide nanoparticles. Mater. Today Chem..

[180] Carrouel F., Viennot S., Ottolenghi L., Gaillard C., Bourgeois D. (2020). Nanoparticles as anti-microbial, anti-inflammatory, and remineralizing agents in oral care cosmetics: A review of the current situation. Nanomaterials.

[181] Pokrowiecki R., Wojnarowicz J., Zareba T., Koltsov I., Lojkowski W., Tyski S., Mielczatek A., Zawadzki P. (2019). Nanoparticles and human saliva: A step towards drug delivery systems for dental and craniofacial biomaterials. Int. J. Nanomed..

[182] Vigneshwaran N., Kumar S., Kathe A.A., Varadarajan P.V., Prasad V. (2006). Function finishing of cotton fabrics using zinc oxide-soluble starch nanocomposites. Nanotechnology.

[183] Bisht G., Rayamajhi S. (2016). ZnO nanoparticles: A promising anticancer agent. Nanobiomedicine.

[184] Ancona A., Dumontel B., Garino N., Demarco B., Chatzitheodoridou D., Fazzini W., Engelke H., Cauda V. (2018). Lipid-coated zinc oxide nanoparticles as innovative ROS-generators for photodynamic therapy in cancer cells. Nanomaterials.

[185] Elsayed E.A., Moussa S.A., El-Enshasy H.A., Wadaan M.A. (2020). Anticancer potentials of zinc oxide nanoparticlesagainst liver and breast cancer cell lines. J. Sci. Ind. Res..

[186] Kielbik P., Kaszewski J., Dominiak B., Damentko M., Serafińska I., Rosowska J., Gralak M.A., Krajewski M., Witkowski B.S., Gajewski Z. (2019). Preliminary studies on biodegradable zinc oxide nanoparticles doped with fe as a potential form of iron delivery to the living organism. Nanoscale Res. Lett..

[187] Garino N., Limongi T., Dumontel B., Canta M., Racca L., Laurenti M., Castellino M., Casu A., Falqui A., Cauda V. (2019). A microwave-assisted synthesis of zinc oxide nanocrystals finely tuned for biological applications. Nanomaterials.

[188] Napi M.L.M., Sultan S.M., Ismail R., How K.W., Ahmad M.K. (2019). Electrochemical-based biosensors on different zinc oxide nanostructures: A review. Materials.

[189] Cao L., Kiely J., Piano M., Luxton R. (2019). A copper oxide/zinc oxide composite nano-surface for use in a biosensor. Materials.

[190] Hahm J. (2016). Fundamental properties of one-dimensional zinc oxide nanomaterials and implementations in various detection modes of enhanced biosensing. Annu. Rev. Phys. Chem..

[191] Banchero M., Mohamed S.S.Y., Leone F., Lopez F., Ronchetti S., Manna L., Onida B. (2019). Supercritical solvent impregnation of different drugs in mesoporous nanostructured ZnO. Pharmaceutics.

[192] Pokrowiecki R., Pałka K., Mielczarek A. (2018). Nanomaterials in dentistry: A cornerstone or a black box?. Nanomedicine.

[193] Leone F., Cataldo R., Mohamed S.S.Y., Manna L., Banchero M., Ronchetti S., Mandras N., Tullio V., Cavalli R., Onida B. (2019). Nanostructured ZnO as multifunctional carrier for a green antibacterial drug delivery system—A feasibility study. Nanomaterials.

[194] Viter R., Savchuk M., Riekstina U., Poletaev N., Pleiko K., Ramanavicius A. Photoluminescence ZnO nanorod biosensors for medical and food safety application. Proceedings of the 2017 IEEE 7th International Conference Nanomaterials: Application & Properties (NAP).

[195] Viter R., Khranovskyy V., Starodub N., Ogorodniichuk Y., Gevelyuk G., Gertnere Z., Poletaev N., Yakimova R., Erts D., Smyntyna V. (2014). Application of room temperature photoluminescence from ZnO nanorods for salmonella detection. IEEE Sens. J..

[196] Sodzel D., Khranovskyy V., Beni V., Turner A.P.F., Viter R., Eriksson M.O., Holtz P.O., Janot J.M., Bechelany M., Balme S. (2015). Continuous sensing of hydrogen peroxide and glucose viaquenching of the UV and visible luminescenceof ZnO nanoparticles. Microchim. Acta.

[197] Tereshchenkoa A., Bechelany M., Viter R., Khranovskyy V., Smyntyna V., Starodub N., Yakimovada R. (2016). Optical biosensors based on ZnO nanostructures: Advantages and perspectives. A review. Sens. Actuators B-Chem..

[198] Tripathy N., Kim D. (2018). Metal oxide modified ZnO nanomaterials for biosensor applications. Nano Converg..

[199] Nagi J.S., Skorenko K., Bernier W., Jones W.E., Doiron A.L. (2020). Near infrared-activated dye-linked ZnO nanoparticles release reactive oxygen species for potential use in photodynamic therapy. Materials.

[200] Sharma P., Jang N.-Y., Lee J.-W., Park B.C., Kim Y.K., Cho N.-H. (2019). Application of ZnO-based nanocomposites for vaccines and cancer immunotherapy. Pharmaceutics.

[201] Bogdan J., Pławińska-Czarnak J., Zarzyńska J. (2017). Nanoparticles of titanium and zinc oxides as novel agents in tumor treatment: A review. Nanoscale Res. Lett..

[202] Nguyen H., Tinet E., Chauveau T., Geinguenaud F., Lalatonne Y., Michel A., Aid-Launais R., Journé C., Lefèbvre C., Simon-Yarza T. (2019). Bimodal fucoidan-coated zinc oxide/iron oxide-based nanoparticles for the imaging of atherothrombosis. Molecules.

[203] Malaikozhundan B. (2018). Pharmaceutical applications of zinc oxide nanoparticles—A review. Acta Sci. Pharm. Sci..

[204] TelesSouza J.M., de Araújo A.R., de Carvalho A.M.A., das Graças Nascimento Amorim A., Daboit T.C., de Souza de Almeida Leite J.R., da Silva D.A., Eaton P. (2020). Sustainably produced cashew gum-capped zinc oxide nanoparticles show antifungal activity against Candida parapsilosis. J. Clean. Prod..

[205] Lee K.S., Song Y., Kim C.H., Kim Y.T., Kang T., Lee S.J., Choi B.G., Lee K.G. (2020). Development of zinc oxide-based sub-micro pillar arrays for on-site capture and DNA detection of foodborne pathogen. J. Colloid Interface Sci..

[206] Păunica-Panea G., Ficai A., Marin M.M., Marin S., Albu M.G., Constantin V.D., Dinu-Pîrvu C., Vuluga Z., Corobea M.C., Ghica M.V. (2016). New Collagen-dextran-zinc oxide composites for wound dressing. J. Nanomater..

[207] Rasmussen J.W., Martinez E., Louka P., Wingett D.G. (2010). Zinc oxide nanoparticles for selective destruction of tumor cells and potential for drug delivery applications. Expert. Opin. Drug. Deliv..

[208] Jin S.-E., Jin J.E., Hwang W., Hong S.W. (2019). Photocatalytic antibacterial application of zinc oxide nanoparticles and self-assembled networks under dual UV irradiation for enhanced disinfection. Int. J. Nanomed..

[209] Ghaffari H., Tavakoli A., Moradi A., Tabarraei A., Bokharaei-Salim F., Zahmatkeshan M., Farahmand M., Javanmard D., Kiani S.J., Esghaei M. (2019). Inhibition of H1N1 influenza virus infection by zinc oxide nanoparticles: Another emerging application of nanomedicine. J. Biomed. Sci..

[210] Pérez R., Sanchez-Salcedo S., Lozano D., Heras C., Esbrit P., Vallet-Regí M., Salinas A.J. (2018). Osteogenic effect of ZnO-mesoporous glasses loaded with osteostatin. Nanomaterials.

[211] Beyene Z., Ghosha R. (2019). Effect of zinc oxide addition on antimicrobial and antibiofilm activity of hydroxyapatite: A potential nanocomposite for biomedical applications. Mater. Today Commun..

[212] Grenho L., Salgado C.L., Fernandes M.H., Monteiro F.J., Ferraz F.J. (2015). Antibacterial activity and biocompatibility of three-dimensional nanostructured porous granules of hydroxyapatite and zinc oxide nanoparticles—An in vitro and in vivo study. Nanotechnology.

[213] Laurenti M., Cauda V. (2017). ZnO nanostructures for tissue engineering applications. Nanomaterials.

[214] Subramaniam V.D., Prasad S.V., Banerjee A., Gopinath M., Murugesan R., Marotta F., Sun X.-F., Pathak S. (2019). Health hazards of nanoparticles: Understanding the toxicity mechanism of nanosized ZnO in cosmetic products. Drug Chem. Toxicol..

[215] Choi S.J., Choy J.H. (2014). Biokinetics of zinc oxide nanoparticles: Toxicokinetics, biological fates, and protein interaction. Int. J. Nanomed..

[216] Hossain F., Perales-Perez O.J., Hwang S., Román F. (2014). Antimicrobial nanomaterials as water disinfectant: Applications, limitations and future perspectives. Sci. Total Environ..

[217] Lu-E Shi L.E., Li Z.H., Zheng W., Zhao Y.F., Jin Y.F., Tang Z.X. (2014). Synthesis, antibacterial activity, antibacterial mechanism and food applications of ZnO nanoparticles: A review. Food Addit. Contam. Part. A.

[218] Długosz O., Szostak K., Staroń A., Pulit-Prociak J., Banach M. (2020). Methods for reducing the toxicity of metal and metal oxide NPs as biomedicine. Materials.

[219] Singh S. (2019). Zinc oxide nanoparticles impacts: Cytotoxicity, genotoxicity, developmental toxicity, and neurotoxicity. Toxicol. Mech. Method..

[220] Schmidt-Mende L., MacManus-Driscoll J.L. (2007). ZnO—Nanostructures, defects, and devices. Mater. Today.

[221] Willander M., Alnoor H., Savoyant A., Adam R.E., Nur O. (2018). Optical and magneto-optical properties of zinc-oxide nanostructures grown by the low-temperature chemical route. Oxide-Based Materials and Devices IX.

[222] Kayani Z.N., Abbas E., Saddiqe Z., Riaz S., Naseem S. (2018). Photocatalytic, antibacterial, optical and magnetic properties of Fe-doped ZnO nano-particles prepared by sol-gel. Mater. Sci. Semicond. Process..

[223] Ohno H. (1998). Making nonmagnetic semiconductors ferromagnetic. Science.

[224] Norris D.J., Efros A.L., Erwin S.C. (2008). Doped nanocrystals. Science.

[225] Dietl T., Ohno H., Matsukura M., Cibert J., Ferrand D. (2000). Zener model description of ferromagnetism in zinc-blende magnetic semiconductors. Science.

[226] Sato K., Katayama-Yosida H. (2002). First principles materials design for semiconductor spintronics. Semicond. Sci. Technol..

[227] Geetha N., Sivaranjani S., Ayeshamariam A., Suthan Kissinger J., Valan Arasu M., Jayachandran M. (2016). ZnO doped oxide materials: Mini review. Fluid. Mech. Open Acc..

[228] Pearton S.J., Norton D.P., Ivill M.P., Hebard A.F., Chen W.M., Buyanova I.A., Zavada J.M. (2007). Transition metal doped ZnO for spintronics. J. Electron. Mater..

[229] Zhang Y., Apostoluk A., Theron C., Cornier T., Canut B., Daniele S., Masenelli B. (2019). Doping of ZnO inorganic-organic nanohybrids with metal elements. Sci. Rep..

[230] Bharat T.C., Shubham, Mondal S., Gupta H.S., Singh P.K., Das A.K. (2019). Synthesis of doped zinc oxide nanoparticles: A review. Mater. Today Proc..

[231] Shohany B.G., Zak A.K. (2020). Doped ZnO nanostructures with selected elements - Structural, morphology and optical properties: A review. Ceram. Int..

[232] Glaspell G., Dutta P., Manivannan A. (2005). A room-temperature and microwave synthesis of M-doped ZnO (M = Co, Cr, Fe, Mn & Ni). J. Clust. Sci..

[233] Rahman F. (2019). Zinc oxide light-emitting diodes: A review. Opt. Eng..

[234] Ding M., Guo Z., Zhou L., Fang X., Zhang L., Zeng L., Xie L., Zhao H. (2018). One-dimensional zinc oxide nanomaterials for application in high-performance advanced optoelectronic devices. Crystals.

[235] Jamadi O., Reveret F., Disseix P., Medard F., Leymarie J., Moreau A., Solnyshkov D., Deparis C., Leroux M., Cambril E. (2018). Edge-emitting polariton laser and amplifier based on a ZnO waveguide. Light Sci. Appl..

[236] Kennedy J., Fang F., Futter J., Leveneur J., Murmu P.P., Panin G.N., Kang T.W., Manikandan E. (2017). Synthesis and enhanced field emission of zinc oxide incorporated carbon nanotubes. Diam. Relat. Mater..

[237] Mao D.S., Wang X., Li W., Liu X.H., Li Q., Xu J.F. (2002). Electron field emission from hydrogen-free amorphous carbon-coated ZnO tip array. J. Vac. Sci. Technol. B.

[238] Nunes Melo A.H., Andrade Macêdo M. (2016). Permanent data storage in ZnO thin films by filamentary resistive switching. PLoS ONE.

[239] Laurenti M., Porro S., Pirri C.F., Ricciardi C., Chiolerio A. (2017). Zinc oxide thin films for memristive devices: A review. Crit. Rev. Solid State Mater. Sci..

[240] Jamalullail N., Salwani Mohamad I., Norizan M.N., Mahmed N., Taib B.N. (2017). Recent improvements on TiO_2_ and ZnO nanostructure photoanode for dye sensitized solar cells: A brief review. Web Conf..

[241] Yu L., Li Z. (2019). Synthesis of Zn_x_Cd_1-x_Se@ZnO hollow spheres in different sizes for quantum dots sensitized solar cells application. Nanomaterials.

[242] Alhammadi S., Park H., Kim W.K. (2019). Optimization of intrinsic ZnO thickness in Cu(In,Ga)Se_2_-based thin film solar cells. Materials.

[243] Vittal R., Ho K.-C. (2017). Zinc oxide based dye-sensitized solar cells: A review. Rene. Sust. Energ. Rev..

[244] Anta J.A., Guillén E., Tena-Zaera R. (2012). ZnO-based dye-sensitized solar cells. J. Phys. Chem. C.

[245] Giannouli M., Govatsi Κ., Syrrokostas G., Yannopoulos S.N., Leftheriotis G. (2018). Factors affecting the power conversion efficiency in ZnO DSSCs: Nanowire vs. nanoparticles. Materials.

[246] Zhang Q., Li C. (2019). TiO_2_ coated ZnO nanorods by mist chemical vapor deposition for application as photoanodes for dye-sensitized solar cells. Nanomaterials.

[247] Omelchenko M.M., Wojnarowicz J., Salamonczyk M., Lojkowski W. (2017). Lyotropic liquid crystal based on zinc oxide nanoparticles obtained by microwave solvothermal synthesis. Mater. Chem. Phys..

[248] Salzano de Luna M., Galizia M., Wojnarowicz J., Rosa R., Lojkowski W., Acierno D., Filippone G., Leonelli C. (2014). Dispersing hydrophilic nanoparticles in hydrophobic polymers: HDPE/ZnO nanocomposites by a novel template-based approach. Express Polym. Lett..

[249] Dong Y., Argaiz M., He B., Tomovska R., Sun T., Martín-Fabiani I. (2020). Zinc oxide superstructures in colloidal polymer nanocomposite films: Enhanced antibacterial activity through slow drying. ACS Appl. Polym. Mater..

[250] Prasert A., Sontikaew S., Sriprapai D., Chuangchote S. (2020). Polypropylene/ZnO nanocomposites: Mechanical properties, photocatalytic dye degradation, and antibacterial property. Materials.

[251] Zhu C., Wang H., Mahmood Z., Wang Q., Ma H. (2018). Biocompatibility and biodegradability of polyacrylate/ZnO nanocomposite during the activated sludge treatment process. PLoS ONE.

[252] Abbas M., Buntinx M., Deferme W., Peeters R. (2019). (Bio)polymer/ZnO nanocomposites for packaging applications: A review of gas barrier and mechanical properties. Nanomaterials.

[253] Luzi F., Di Michele A., Torre L., Puglia D. (2019). Active role of ZnO nanorods in thermomechanical and barrier performance of poly(vinyl alcohol-co-ethylene) formulations for flexible packaging. Polymers.

[254] Mizielińska M., Kowalska U., Jarosz M., Sumińska P., Landercy N., Duquesne E. (2018). The effect of UV aging on antimicrobial and mechanical properties of PLA films with incorporated zinc oxide nanoparticles. Int. J. Environ. Res. Public Health.

[255] Roy S., Rhim J.-W. (2020). Carboxymethyl cellulose-based antioxidant and antimicrobial active packaging film incorporated with curcumin and zinc oxide. Int. J. Biol. Macromol..

[256] Kim I., Viswanathan K., Kasi G., Thanakkasaranee S., Sadeghi K., Seo J. (2020). ZnO nanostructures in active antibacterial food packaging: Preparation methods, antimicrobial mechanisms, safety issues, future prospects, and challenges. Food Rev. Int..

[257] Mania S., Cieślik M., Konzorski M., Święcikowski P., Nelson A., Banach A., Tylingo R. (2020). The Synergistic Microbiological Effects of Industrial Produced Packaging Polyethylene Films Incorporated with Zinc Nanoparticles. Polymers.

[258] Kim I., Viswanathan K., Kasi G., Sadeghi K., Thanakkasaranee S., Seo J. (2019). Poly(Lactic Acid)/ZnO bionanocomposite films with positively charged ZnO as potential antimicrobial food packaging materials. Polymers.

[259] Bolognesi C., Castle L., Cravedi J., Engel K., Franz R., Fowler P., Grob K., Gürtler R., Husøy T., Kärenlampi S. (2016). Safety assessment of the substance zinc oxide, nanoparticles, for use in food contact materials. EFSA J..

[260] Sun Y., Chen L., Bao Y., Zhang Y., Wang J., Fu M., Wu J., Ye D. (2016). The applications of morphology controlled ZnO in catalysis. Catalysts.

[261] Zhang Z.-Y., Xiong H.-M. (2015). Photoluminescent ZnO nanoparticles and their biological applications. Materials.

[262] Lamba N., Gupta R., Modak J.M., Madras G. (2019). ZnO catalyzed transesterification of Madhuca indica oil in supercritical methanol. Fuel.

[263] Ong C.B., Ng L.Y., Mohammad A.W. (2018). A review of ZnO nanoparticles as solar photocatalysts: Synthesis, mechanisms and applications. Renew. Sustain. Energy Rev..

[264] Chang J.S., Strunk J., Chong M.N., Poh P.E., Ocon J.D. (2020). Multi-dimensional zinc oxide (ZnO) nanoarchitectures as efficient photocatalysts: What is the fundamental factor that determines photoactivity in ZnO?. J. Hazard. Mater..

[265] Shen Z., Zhou H., Chen H., Xu H., Feng C., Zhou X. (2018). Synthesis of nano-zinc oxide loaded on mesoporous silica by coordination effect and its photocatalytic degradation property of methyl orange. Nanomaterials.

[266] Hamid S.B.A., Teh S.J., Lai C.W. (2017). Photocatalytic water oxidation on ZnO: A review. Catalysts.

[267] Tudose I.V., Suchea M. (2016). ZnO for photocatalytic air purification applications. IOP Conf. Ser. Mater. Sci. Eng..

[268] Horikoshi S., Matsubara A., Takayama S., Sato M., Sakai F., Kajitani M., Abe M., Serpone N. (2010). Characterization of microwave effects on metal-oxide materials: Zinc oxide and titanium dioxide. App. Catal. B.

[269] Kumari V., Mittal A., Jindal J., Yadav S., Kumar N. (2019). S-, N- and C-doped ZnO as semiconductor photocatalysts: A review. Front. Mater. Sci..

[270] Zhang Y., Ram M.K., Stefanakos E.K., Goswami D.Y. (2012). Synthesis, characterization, and applications of ZnO nanowires. J. Nanomater..

[271] Mirzaei A., Chen Z., Haghighat F., Yerushalmi L. (2016). Removal of pharmaceuticals and endocrine disrupting compounds from water by zinc oxide-based photocatalytic degradation: A review. Sustain. Cities Soc..

[272] Siwińska-Stefańska K., Kubiak A., Piasecki A., Goscianska J., Nowaczyk G., Jurga S., Jesionowski T. (2018). TiO_2_-ZnO binary oxide systems: Comprehensive characterization and tests of photocatalytic activity. Materials.

[273] Kubiak A., Siwińska-Ciesielczyk K., Jesionowski T. (2018). Titania-based hybrid materials with ZnO, ZrO_2_ and MoS_2_: A review. Materials.

[274] Ko H.-U., Mun S., Min S.-K., Kim G.-W., Kim J. (2014). Fabrication of cellulose ZnO hybrid nanocomposite and its strain sensing behavior. Materials.

[275] Ibrahim A.A., Tiwari P., Al-Assiri M.S., Al-Salami A.E., Umar A., Kumar R., Kim S.H., Ansari Z.A., Baskoutas S. (2017). A highly-sensitive picric acid chemical sensor based on ZnO nanopeanuts. Materials.

[276] Chung R.-J., Wang A.-N., Liao Q.-L., Chuang K.-Y. (2017). Non-enzymatic glucose sensor composed of carbon-coated nano-zinc oxide. Nanomaterials.

[277] Pavlenko M., Myndrul V., Gottardi G., Coy E., Jancelewicz M., Iatsunskyi I. (2020). Porous silicon-zinc oxide nanocomposites prepared by atomic layer deposition for biophotonic applications. Materials.

[278] Blachowicz T., Ehrmann A. (2020). Recent developments in electrospun ZnO nanofibers: A short review. J. Eng. Fiber. Fabr..

[279] Ferrone E., Araneo R., Notargiacomo A., Pea M., Rinaldi A. (2019). ZnO nanostructures and electrospun ZnO–Polymeric hybrid nanomaterials in biomedical, health, and sustainability applications. Nanomaterials.

[280] Justeau C., Slimani Tlemcani T., Poulin-Vittrant G., Nadaud K., Alquier D. (2019). A comparative study on the effects of Au, ZnO and AZO seed layers on the performance of ZnO nanowire-based piezoelectric nanogenerators. Materials.

[281] Rafique S., Kasi A.K., Kasi J.K., Aminullah, Bokhari M., Shakoor Z. (2020). Fabrication of silver-doped zinc oxidenanorods piezoelectric nanogeneratoron cotton fabric to utilize and optimizethe charging system. Nanomater. Nanotechnol..

[282] Wang X., Zhou J., Wang Z.L., Zhou Z., Wang Z., Lin L. (2012). Nanopiezotronics and nanogenerators. Microsystems and Nanotechnology.

[283] Wang S., Gao F., Ma R., Du A., Tan T., Du M., Zhao X., Fan Y., Wen M. (2018). ZnO nanoparticles anchored on a N-doped graphene-coated separator for high performance lithium/sulfur batteries. Metals.

[284] Fernando J.F.S., Zhang C., Firestein K.L., Nerkarb J.Y., Golberg D.V. (2019). ZnO quantum dots anchored in multilayered and flexible amorphous carbon sheets for high performance and stable lithium ion batteries. J. Mater. Chem. A.

[285] Movsesyan L., Maijenburg A.W., Goethals N., Sigle W., Spende A., Yang F., Kaiser B., Jaegermann W., Park S.-Y., Mul G. (2018). ZnO nanowire networks as photoanode model systems for photoelectrochemical applications. Nanomaterials.

[286] Cardoza-Contreras M.N., Vásquez-Gallegos A., Vidal-Limon A., Romo-Herrera J.M., Águila S., Contreras O.E. (2019). Photocatalytic and antimicrobial properties of Ga doped and Ag doped ZnO nanorods for water treatment. Catalysts.

[287] Dimapilis E.A.S., Hsu C.S., Mendoza R.M.O., Lu M.-C. (2018). Zinc oxide nanoparticles for water disinfection. Sustain. Environ. Res..

[288] Butt N.M., Chaudhary M.F., Rubab F. (2015). Antibacterial effect of zinc oxide nanoparticles against water borne bacteria. TechConnect Briefs.

[289] Azzouz I., Habba Y., Capochichi-Gnambodoe M., Marty F., Vial J., Leprince-Wang Y., Bourouina T. (2018). Zinc oxide nano-enabled microfluidic reactor for water purification and its applicability to volatile organic compounds. Microsyst. Nanoeng..

[290] Al-Issai L., Elshorbagy W., Maraqa M.A., Hamouda M., Soliman A.M. (2019). Use of nanoparticles for the disinfection of desalinated water. Water.

[291] Lee K.M., Lai C.W., Ngai K.S., Juan J.C. (2016). Recent developments of zinc oxide based photocatalyst in water treatment technology: A review. Water Res..

[292] Huang J., Huang G., An C., He Y., Yao Y., Zhang P., Shen J. (2018). Performance of ceramic disk filter coated with nano ZnO for removing Escherichia coli from water in small rural and remote communities of developing regions. Environ. Pollut..

[293] Kegel J., Povey I.M., Pemble M.E. (2018). Zinc oxide for solar water splitting: A brief review of the material’s challenges and associated opportunities. Nano Energy.

[294] Lede J., Elorza-Ricart E., Ferrer M. (2001). Solar thermal splitting of zinc oxide: A review of some of the rate controlling factors. J. Sol. Energy Eng..

[295] Liu M., Nam C.-T., Black C.T., Kamcev J., Zhang L. (2013). Enhancing water splitting activity and chemical stability of zinc oxide nanowire photoanodes with ultrathin titania shells. J. Phys. Chem. C.

[296] Al-Fori M., Dobretsov S., Myint M.T., Dutta J. (2014). Antifouling properties of zinc oxide nanorod coatings. Biofouling.

[297] Pulit-Prociak J., Pszczółka K., Chwastowski J., Staroń P., Staroń A., Sikora E., Michałowski S., Banach M. (2019). Preparation of PVA-based composites with the addition of zinc oxide nanoparticles. J. Inorg. Organomet. Polym..

[298] Verbič A., Gorjanc M., Simončič B. (2019). Zinc oxide for functional textile coatings: Recent advances. Coatings.

[299] Woźniak B., Dąbrowska S., Wojnarowicz J., Chudoba T., Łojkowski W. (2017). Content available remote Coating synthetic materials with zinc oxide nanoparticles acting as a UV filter. Glass Ceram..

[300] Pulit-Prociak J., Chwastowski J., Kucharski A., Banach M. (2016). Functionalization of textiles with silver and zinc oxide nanoparticles. App. Surf. Sci..

[301] Fu Y., Fu W., Liu Y., Zhang G., Liu Y., Yu H. (2015). Comparison of ZnO nanorod array coatings on wood and their UV prevention effects obtained by microwave-assisted hydrothermal and conventional hydrothermal synthesis. Holzforschung.

[302] Tański T., Matysiak W. (2017). Optical properties of PVP/ZnO composite thin films. J. Achiev. Mater. Manuf. Eng..

[303] Fiedot-Toboła M., Ciesielska M., Maliszewska I., Rac-Rumijowska O., Suchorska-Woźniak P., Teterycz H., Bryjak M. (2018). Deposition of zinc oxide on different polymer textiles and their antibacterial properties. Materials.

[304] Mahamuni-Badiger P.P., Patil P.M., Badiger M.V., Patel P.R., Thorat-Gadgil B.S., Pandit A., Bohara R.A. (2020). Biofilm formation to inhibition: Role of zinc oxide-based nanoparticles. Mater. Sci. Eng. C.

[305] Wallenhorst L., Gurau L., Gellerich A., Militz H., Ohms G., Viöl W. (2018). UV-blocking properties of Zn/ZnO coatings on wood deposited by cold plasma spraying at atmospheric pressure. Appl. Surf. Sci..

[306] García-López J.I., Niño-Medina G., Olivares-Sáenz E., Lira-Saldivar R.H., Barriga-Castro E.D., Vázquez-Alvarado R., Rodríguez-Salinas P.A., Zavala-García F. (2019). Foliar application of zinc oxide nanoparticles and zinc sulfate boosts the content of bioactive compounds in habanero peppers. Plants.

[307] Singh A., Singh N.B., Afzal S., Hussain I. (2018). Zinc oxide nanoparticles: A review of their biological synthesis, antimicrobial activity, uptake, translocation and biotransformation in plants. J. Mater. Sci..

[308] Chaudhary S., Umar A. (2017). ZnO nanostructures and their sensing applications: A review. Nanosci. Nanotechnol. Lett..

[309] Chaudhary S., Umar A., Bhasin K.K., Baskoutas S. (2018). Chemical sensing applications of ZnO nanomaterials. Materials.

[310] Sabir S., Arshad M., Chaudhari K.C. (2014). Zinc oxide nanoparticles for revolutionizing agriculture: Synthesis and applications. Sci. World J..

[311] Rajput V.D., Minkina T.M., Behal A., Sushkova S.N., Mandzhieva S., Singh R., Gorovtsov A., Tsitsuashvili V.S., Purvis W.O., Karen A. (2018). Effects of zinc-oxide nanoparticles on soil, plants, animals and soil organisms: A review. Environ. Nanotechnol. Monit. Manag..

[312] Heggelund L.H., Diez-Ortiz M., Lofts M.S., Lahive E., Jurkschat K., Wojnarowicz J., Cedergreen N., Spurgeon D., Svendsen C. (2014). Soil pH effects on the comparative toxicity of dissolved zinc, non-nano and nano ZnO to the earthworm Eisenia fetida. Nanotoxicology.

[313] Lončarić Z., Hackenberger D.K., Jug I., Hackenbergera B.K. (2020). Is nano ZnO/chlorpyrifos mixture more harmful to earthworms than bulk ZnO? A multigeneration approach. Chemosphere.

[314] Hooper H.L., Jurkschat K., Morgan A.J., Bailey J., Lawlor A.J., Spurgeon D.J., Svendsen C. (2011). Comparative chronic toxicity of nanoparticulate and ionic zinc to the earthworm Eisenia veneta in a soil matrix. Environ. Int..

[315] Laycock A., Diez-Ortiz M., Larnerm F., Dybowska A., Spurgeon D., Valsami-Jones E., Rehkämper M., Svendsen C. (2016). Earthworm uptake routes and rates of ionic Zn and ZnO nanoparticles at realistic concentrations, traced using stable isotope labeling. Environ. Sci. Technol..

[316] Polak N., Read D.S., Jurkschat K., Matzke M., Kelly F.J., Spurgeon D.J., Stürzenbaum S.R. (2014). Metalloproteins and phytochelatin synthase may confer protectionagainst zinc oxide nanoparticle induced toxicity in Caenorhabditis elegans. Comp. Biochem. Phys. C.

[317] Read D.S., Matzke M., Gweon H.S., Newbold L.K., Heggelund L., Diez Ortiz M., Lahive E., Spurgeon D., Svendsen S. (2016). Soil pH effects on the interactions between dissolved zinc, non-nano- and nano-ZnO with soil bacterial communities. Environ. Sci. Pollut. Res..

[318] Waalewijn-Kool P.L., Diez Ortiz M., Cornelis S.L., van Gestel C.A.M. (2013). The effect of pH on the toxicity of zinc oxide nanoparticles to Folsomia candida in amended field soil. Environ. Toxicol. Chem..

[319] Waalewijn-Kool P.L., Diez Ortiz M., van Straalen N.M., van Gestel C.A.M. (2013). Sorption, dissolution and pH determine the long-term equilibration and toxicity of coated and uncoated ZnO nanoparticles in soil. Environ. Pollut..

[320] Waalewijn-Kool P.L., Diez Ortiz M., van Gestel C.A.M. (2012). Effect of different spiking procedures on the distribution and toxicity of ZnO nanoparticles in soil. Ecotoxicology.

[321] Romero-Freire A., Lofts S., Martin Peinado F.J., van Gestel C.A.M. (2017). Effects of aging and soil properties on zinc oxide nanoparticle availability and its ecotoxicological effects to the earthworm Eisenia Andrei. Environ. Toxicol. Chem..

[322] Hrdá K., Opršal J., Knotek P., Pouzar M., Vlček M. (2016). Toxicity of zinc oxide nanoparticles to the annelid Enchytraeus crypticus in agar-based exposure media. Chem. Pap..

[323] Hu C.W., Lia M., Cui Y.B., Li D.S., Chen J., Yang L.Y. (2010). Toxicological effects of TiO_2_ and ZnO nanoparticles in soilon earthworm Eisenia fetida. Soil Biol. Biochem..

[324] van Gestel C.A.M., Kool P.L., Diez Ortiz M. (2010). Metal-based nanoparticles in soil: New research themes should not ignore old rules and theories. Comments on the paper by Hu et al., 2010 “Toxicological effects of TiO_2_ and ZnO nanoparticles in soil on earthworm Eisenia fetida”. Soil Biol. Biochem..

[325] Świątek Z.M., van Gestel C.A.M., Bednarska A.J. (2017). Toxicokinetics of zinc-oxide nanoparticles and zinc ions in the earthworm Eisenia andrei. Ecotox. Environ. Saf..

[326] Tourinho P.S., van Geste C.A.M., Lofts S., Svendsen C., Soares A.M.V.M., Loureiro S. (2012). Metal-based nanoparticles in soil: Fate, behavior, and effects on soil invertebrates. Environ. Toxicol. Chem..

[327] Tourinho P.S., van Gestel C.A.M., Lofts S., Soares A.M.V.M., Loureiro S. (2013). Influence of soil pH on the toxicity of zinc oxide nanoparticles to the terrestrial isopod Porcellionides pruinosus. Environ. Toxicol. Chem..

[328] Lopes S., Ribeiro F., Wojnarowicz J., Łojkowski W., Jurkschat K., Crossley A., Soares A.M., Loureiro S. (2014). Zinc oxide nanoparticles toxicity to Daphnia magna: Size-dependent effects and dissolution. Environ. Toxicol. Chem..

[329] Tomczyk-Wydrych I., Rabajczyk A. (2019). Transformations of metal nanoparticles in the aquatic environment and threat to environmental safety. SFT.

[330] Maiga D.T., Nyoni H., Nkambule T.T., Mamba B.B., Makudali Msagati T.A. (2020). Impact of zinc oxide nanoparticles in aqueous environments: Influence of concentrations, natural organic matter and ionic strength. Inorg. Nano-Met. Chem..

[331] Garner K.L., Keller A.A. (2014). Emerging patterns for engineered nanomaterials in the environment: A review of fate and toxicity studies. J. Nanopart. Res..

[332] Leareng S.K., Ubomba-Jaswa E., Musee N. (2020). Oxicity of zinc oxide and iron oxide engineered nanoparticles to Bacillus subtilis in river water systems. Environ. Sci. Nano.

[333] Spurgeon D.J., Lahive E., Schultz C.L. (2020). Nanomaterial Transformations in the Environment: Effects of Changing Exposure Forms on Bioaccumulation and Toxicity. Small.

[334] Tomczyk-Wydrych I., Rabajczyk A. (2019). Metal nanoparticles in surface waters—A risk to aquatic organisms. SFT.

[335] Hou J., Wu Y., Li X., Wei B., Li S., Wang X. (2018). Toxic effects of different types of zinc oxide nanoparticles on algae, plants, invertebrates, vertebrates and microorganisms. Chemosphere.

[336] Rastogi A., Zivcak M., Sytar O., Kalaji H.M., He X., Mbarki S., Brestic M. (2017). Impact of metal and metal oxide nanoparticles on plant: A critical review. Front. Chem..

[337] Xu X., Yi Z., Chen D., Duan X., Zhou Z., Fan X., Jiang M. (2011). Evaluation of photocatalytic production of active oxygen and decomposition of phenol in ZnO suspensions. Rare Metals.

[338] Vasile O.R., Andronescu E., Ghiţulică C., Vasile B.S., Vasile E. (2014). Grain size effect on photocatalytic properties of nanocrystalline ZnO. UPB Sci. Bull. Ser. B.

[339] Ruiz-Hitzky E., Aranda P., Akkari M., Khaorapapong N., Ogawa M. (2019). Photoactive nanoarchitectures based on clays incorporating TiO_2_ and ZnO nanoparticles. Beilstein J. Nanotechnol..

[340] Mclaren A., Valdes-Solis T., Li G., Tsang S.C. (2009). Shape and size effects of ZnO nanocrystals on photocatalytic activity. J. Am. Chem. Soc..

[341] Han N.S., Kim D., Lee J.W., Kim J., Shim H.S., Lee Y., Lee D., Song J.K. (2016). Unexpected size effect observed in ZnO-Au composite photocatalysts. ACS Appl. Mater. Interfaces.

[342] Shahine I., Beydoun N., Gaumet J.J., Bendeif E.-E., Rinnert H., Magri P., En Naciri A., Miska P., Jradi S., Akil S. (2019). Pure, size tunable ZnO nanocrystals assembled into large area PMMA layer as efficient catalyst. Catalysts.

[343] Li N., Jiao F., Pan X., Ding Y., Feng J., Bao X. (2019). Size effects of ZnO nanoparticles in bifunctional catalysts for selective syngas conversion. ACS Catal..

[344] Parvez Ahmada M.D., Venkateswara Ra A., Suresh Babu K., Narsinga Rao G. (2019). Particle size effect on the dielectric properties of ZnO nanoparticles. Mater. Chem. Phys..

[345] Sun X., Shen Y., Lin P., Kang Z., Zhang Y., Zhang Y. (2017). Property characterisation and optimisation. ZnO Nanostructures: Fabrication and Applications.

[346] Li T., Li Y.T., Qin W.W., Zhang P.P., Chen X.Q., Hu X.F., Zhang W. (2015). Piezoelectric size effects in a zinc oxide micropillar. Nanoscale Res. Lett..

[347] Macary L.S., Kahn M.L., Estournès C., Fau P., Trémouilles D., Bafleur M., Renaud P., Chaudret B. (2009). Size effect on properties of varistors made from zinc oxide nanoparticles through low temperature spark plasma sintering. Adv. Func. Mater..

[348] Zhang L., Yin L., Wang C., Lun N., Qi Y., Xiang D. (2010). Origin of visible photoluminescence of ZnO quantum dots: Defect-dependent and size-dependent. J. Phys. Chem. C.

[349] Wang S., Cui Z., Xue Y. (2015). Size-dependent thermodynamic properties of the reaction of nano-ZnO with benzoic acid. NANO.

[350] Herrera-Rivera R., Maldonado A., Olvera M.L. Particle size effect on gas sensing properties of ZnO pellets. Proceedings of the 13th International Conference on Electrical Engineering, Computing Science and Automatic Control (CCE).

[351] Xu J., Pan Q., Shun Y., Tiana Z. (2000). Grain size control and gas sensing properties of ZnO gas sensor. Sens. Actuators B-Chem..

[352] Park Y., Yoo R., Park S., Lee J., Jung H., Lee W. Size effects on sensing properties of ZnO nanoparticles for detection of isoprene. Proceedings of the 17th International Meeting on Chemical Sensors—IMCS.

[353] Ramya M., Nideep T.K., Nampoori V.P.N., Kailasnath M. (2019). Particle size and concentration effect on thermal diffusivity of water-based ZnO nanofluid using the dual-beam thermal lens technique. Appl. Phys. B.

[354] Wang X.F., Fang Y.L., Li L.L., Wang F.J. (2014). Size-dependence of photoluminescence property of ZnO nanoparticles. Adv. Mater. Res..

[355] Irimpan L., Nampoori V.P.N., Radhakrishnan P. (2008). Size-dependent enhancement of nonlinear optical properties in nanocolloids of ZnO. J. Appl. Phys..

[356] Goh E.G., Xu X., McCormick P.G. (2014). Effect of particle size on the UV absorbance of zinc oxide nanoparticles. Scr. Mater..

[357] Babu P., Subastri E., Suyavaran A., Premkumar K., Sujatha V., Aristatile B., Alshammari G.M., Dharuman V., Thirunavukkarasu C. (2017). Size dependent uptake and hemolytic effect of zinc oxide nanoparticles on erythrocytes and biomedical potential of ZnO-ferulic acid conjugates. Sci. Rep..

[358] Pan C., Liu W., Bien M., Lin I., Hsiao T., Ma C., Lai C., Chen M., Chuang K., Chuang H. (2014). Effects of size and surface of zinc oxide and aluminum-doped zinc oxide nanoparticles on cell viability inferred by proteomic analyses. Int. J. Nanomed..

[359] Kong I.C., Raliya R., Ko K.-S., Biswas P. (2018). ZnO nanoparticles: Effect of size on bacterial bioluminescence, seed germination, algal growth, and gene mutation. Environ. Eng. Sci..

[360] Sahu D., Kannan G.M., Vijayaraghavan R. (2014). Size-dependent effect of zinc oxide on toxicity and inflammatory potential of human monocytes. J. Toxicol. Environ. Health Part A.

[361] Baek M., Kim M.K., Cho H.J., Lee J.A., Yu J., Chung H.E., Choi S.J. (2011). Factors influencing the cytotoxicity of zinc oxide nanoparticles: Particle size and surface charge. J. Phys. Conf. Ser..

[362] Shalini D., Senthilkumar S., Rajaguru P. (2018). Effect of size and shape on toxicity of zinc oxide (ZnO) nanomaterials in human peripheral blood lymphocytes. Toxicol. Mech. Methods.

[363] Kim M.-K., Lee J.-A., Jo M.-R., Choi S.-J. (2016). Bioavailability of silica, titanium dioxide, and zinc oxide nanoparticles in rats. J. Nanosci. Nanotechnol..

[364] Yu J., Kim H.-J., Go M.-R., Bae S.-H., Choi S.-J. (2017). ZnO interactions with biomatrices: Effect of particle size on ZnO-protein corona. Nanomaterials.

[365] Zhang J., Wang C., Chowdhuryb R., Adhikaria S. (2013). Size- and temperature-dependent piezoelectric properties of gallium nitride nanowires. Scr. Mater..

[366] Umbaidilah S.Z., Asib N.A.M., Afaah A.N., Rusop M., Khusaimi Z. (2019). A review on the effect of metal doped ZnO nanostructures on ultraviolet photoconductive sensor performance. AIP Conf. Proc..

[367] Hirohata A., Takanashi K. (2014). Applied physics topical review future perspectives for spintronic devices. J. Phys. D Appl. Phys..

[368] Joshi V.K. (2016). Spintronics: A contemporary review of emerging electronics devices. Eng. Sci. Technol. Int. J..

[369] Teulon J.-M., Godon C., Chantalat L., Moriscot C., Cambedouzou J., Odorico M., Ravaux J., Podor R., Gerdil A., Habert A. (2019). On the operational aspects of measuring nanoparticle sizes. Nanomaterials.

[370] Żelechowska K. (2014). Methods of ZnO nanoparticles synthesis. BioTechnologia.

[371] Mirzaei H., Darroudi M. (2017). Zinc oxide nanoparticles: Biological synthesis and biomedical applications. Cer. Int..

[372] Agarwal H., Venkat Kumar S., Rajeshkumar S. (2017). A review on green synthesis of zinc oxide nanoparticles—An eco-friendly approach. Resour.-Effic. Technol..

[373] Manjunatha R.L., Usharani K.V., Naik D. (2019). Synthesis and characterization of ZnO nanoparticles: A review. J. Pharmacogn. Phytochem..

[374] Umamaheswari A., Lakshmana Prabu S., Puratchikody A. (2018). Biosynthesis of zinc oxide nanoparticle: A review on greener approach. MOJ Bioequiv. Availab..

[375] Parihar V., Raja M., Paulose R. (2018). A brief review of structural, electrical and electrochemical properties of zinc oxide nanoparticles. Rev. Adv. Mater. Sci..

[376] Raut S.B., Thorat P.V. (2015). A review on preparation, characterization and application of zinc oxide (ZnO) nanoparticles by green synthesis method. Int. J. Emerg. Technol. Adv. Eng..

[377] Kumar P., Kumar Walia Y. (2014). Synthesis and structural properties of zinc oxide nano particles (ZnO NPs): A review. Asian J. Adv. Basic Sci..

[378] Ul Haq A.N., Nadhman A., Ullah I., Mustafa G., Yasinzai M., Khan I. (2017). Synthesis approaches of zinc oxide nanoparticles: The dilemma of ecotoxicity. J. Nanomater..

[379] Wang Z.L. (2004). Zinc oxide nanostructures: Growth, properties and applications. J. Phys. Condens. Matter.

[380] Lakshmi Ranganatha V., Nithin K.S., Khanum S.A., Nagaraju G., Mallikarjunaswamy C. (2019). Zinc oxide nanoparticles: A significant review on synthetic strategies, characterization and applications. AIP Conf. Proc..

[381] Bandeira M., Giovanela M., Roesch-Ely M., Devine D.M., da Silva Crespo J. (2020). Green synthesis of zinc oxide nanoparticles: A review of the synthesis methodology and mechanism of formation. Sustain. Chem. Pharm..

[382] Güllüce M., Karaday M., Demir A.Y., Işık C., Alaylar B., İspirli N.H. (2020). Genotoxic potentials of biosynthesized zinc oxide nanoparticles. Pol. J. Environ. Stud..

[383] Basnet P., Chanu T.I., Samanta D., Chatterjee S. (2018). A review on bio-synthesized zinc oxide nanoparticles using plant extracts as reductants and stabilizing agents. J. Photoch. Photobiol. B.

[384] Lakshmi S.J., Bai R.S., Sharanagouda H., Ramachandra C., Nidoni U. (2017). A review study of zinc oxide nanoparticles synthesis from plant extracts. Green Chem. Technol. Lett..

[385] Tynell T., Karppinen M. (2014). Atomic layer deposition of ZnO: A review. Semicond. Sci. Technol..

[386] Rodrigues J., Fernandes A.J.S., Monteiro T., Costa F.M. (2019). A review on the laser-assisted flow deposition method: Growth of ZnO micro and nanostructures. CrystEngComm.

[387] Znaidi L. (2010). Sol–gel-deposited ZnO thin films: A review. Mater. Sci. Eng. B.

[388] Taghizadeh S.-M., Lal N., Ebrahiminezhad A., Moeini F., Seifan M., Ghasemi Y., Berenjian A. (2020). Green and economic fabrication of zinc oxide (ZnO) nanorods as a broadband UV blocker and antimicrobial agent. Nanomaterials.

[389] Biron D.D.S., Santos V.D., dos Bergmann C.P. (2020). Synthesis and Characterization of Zinc Oxide Obtained by Combining Zinc Nitrate with Sodium Hydroxide in Polyol Medium. Mater. Res..

[390] Salah N., Habib S.S., Khan Z.H., Memic A., Azam A., Alarfaj E., Zahed N., Alhamedi S. (2011). High-energy ball milling technique for ZnO nanoparticles as antibacterial material. Int. J. Nanomed..

[391] Thanh N.T.K., Maclean N., Mahiddine S. (2014). Mechanisms of nucleation and growth of nanoparticles in solution. Chem. Rev..

[392] Niederbergera M., Cölfena H. (2006). Oriented attachment and mesocrystals: Non-classical crystallization mechanisms based on nanoparticleassembly. Phys. Chem. Chem. Phys..

[393] Inubushi Y., Takami R., Iwasaki M., Tada H., Ito S. (1998). Mechanism of formation of nanocrystalline ZnO particles through the reaction of [Zn(Acac)_2_] with NaOH in EtOH. J. Colloid Interface Sci..

[394] Du H., Yuan F., Huang S., Li J., Zhu Y. (2004). A new reaction to ZnO nanoparticles. Chem. Lett..

[395] Rodríguez-Paéz J.E., Caballero A.C., Villegas M., Moure C., Durán P., Fernández J.F. (2001). Controlled precipitation methods: Formation mechanism of ZnO nanoparticles. J. Eur. Ceram. Soc..

[396] Tonto P., Mekasuwandumrong O., Phatanasri S., Pavarajarn V., Praserthdam P. (2008). Preparation of ZnO nanorod by solvothermal reaction of zinc acetate in various alcohols. Ceram. Int..

[397] Šarića A., Despotović I., Štefanić G. (2019). Alcoholic solvent influence on ZnO synthesis: A joint experimental and theoretical study. J. Phys. Chem. C.

[398] Šarić A., Gotić M., Štefanić G., Dražić G. (2017). Synthesis of ZnO particles using water molecules generated in esterification reaction. J. Mol. Struct..

[399] Lee W., Leem J.Y. (2017). Size control of ZnO nanorods using the hydrothermal method in conjunction with substrate rotation. J. Nanosci. Nanotechnol..

[400] Niederberger M., Pinna N. (2009). Metal Oxide Nanoparticles in Organic Solvents.

[401] Duraimurugan J., Kumar G.S., Venkatesh M., Maadeswaran P., Girija E.K. (2018). Morphology and size controlled synthesis of zinc oxide nanostructures and their optical properties. J. Mater. Sci. Mater. Electron..

[402] Wojnarowicz J., Chudoba T., Koltsov I., Gierlotka S., Dworakowska S., Lojkowski W. (2018). Size control mechanism of ZnO nanoparticles obtained in microwave solvothermal synthesis. Nanotechnology.

[403] Konduracka E. (2019). A link between environmental pollution and civilization disorders: A mini review. Rev. Environ. Health.

[404] Ali S.H., Puppim de Oliveira J.A. (2018). Pollution and economic development: An empirical research review. Environ. Res. Lett..

[405] Weiand L., Schmitz S., Becker S., Niehoff N., Schwartzbach F., von Schneidemesser E. (2019). Climate change and air pollution: The connection between traffic intervention policies and public acceptance in a local context. Environ. Res. Lett..

[406] Tsuji M. (2017). Microwave-assisted synthesis of metallic nanomaterials in liquid phase. ChemistrySelect.

[407] Zhu Y.J., Chen F. (2014). Microwave-assisted preparation of inorganic nanostructures in liquid phase. Chem. Rev..

[408] Saloga P.E.J., Kästner C., Thünemann A.F. (2018). High-speed but not magic: Microwave-assisted synthesis of ultra-small silver nanoparticles. Langmuir.

[409] Kusnieruk S., Wojnarowicz S., Chodara A., Chudoba T., Gierlotka S., Lojkowski W. (2016). Influence of hydrothermal synthesis parameters on the properties of hydroxyapatite nanoparticles. Beilstein J. Nanotechnol..

[410] Szałaj U., Świderska-Środa A., Chodara A., Gierlotka S., Łojkowski W. (2019). Nanoparticle size effect on water vapour adsorption by hydroxyapatite. Nanomaterials.

[411] Opalinska A., Malka I., Dzwolak W., Chudoba T., Presz A., Lojkowski W. (2015). Size-dependent density of zirconia nanoparticles. Beilstein J. Nanotechnol..

[412] Opalinska A., Leonelli C., Lojkowski W., Pielaszek R., Grzanka E., Chudoba T., Matysiak H., Wejrzanowski T., Kurzydlowski T. (2006). Effect of pressure on synthesis of Pr-doped zirconia powders produced by microwave-driven hydrothermal reaction. J. Nanomater..

[413] Falk G.S., Borlaf M., López-Muñoz M.J., Fariñas J.C., Rodrigues Neto J.B., Moreno R. (2018). Microwave-assisted synthesis of TiO_2_ nanoparticles: Photocatalytic activity of powders and thin films. J. Nanopart. Res..

[414] Bonamartini Corradia A., Bondiolia F., Ferrarib A.M., Fochera B., Leonelli C. (2006). Synthesis of silica nanoparticles in a continuous-flow microwave reactor. Powder Technol..

[415] He D., Wan G., Hao H., Chen D., Lu J., Zhang L., Liu F., Zhong L., He S., Luo Y. (2016). Microwave-assisted rapid synthesis of CeO_2_ nanoparticles and its desulfurization processes for CH_3_SH catalytic decomposition. Chem. Eng. J..

[416] Krishnakumar T., Pinna N., Prasanna Kumari K., Perumal K., Jayaprakash R. (2008). Microwave-assisted synthesis and characterization of tin oxide nanoparticles. Mater. Lett..

[417] Koltsov I., Przesniak-Welenc M., Wojnarowicz J., Rogowska A., Mizeracki J., Malysa M., Kimmel G. (2017). Thermal and physical properties of ZrO_2_-AlO(OH) nanopowders synthesised by microwave hydrothermal method. J. Therm. Anal. Calorim..

[418] Koltsov I., Wojnarowicz J., Nyga P., Smalc-Koziorowska J., Stelmakh S., Babyszko A., Morawski A.W., Lojkowski W. (2019). Novel photocatalytic nanocomposite made of polymeric carbon nitride and metal oxide nanoparticles. Molecules.

[419] Zeng G., Huang L., Huang Q., Liu M., Xu D., Huang H., Yang Z., Deng F., Zhang X., Wei Y. (2018). Rapid synthesis of MoS_2_-PDA-Ag nanocomposites as heterogeneous catalysts and antimicrobial agents via microwave irradiation. Appl. Surf. Sci..

[420] Hitchcock R.T. (2004). Radio-Frequency and Microwave Radiation.

[421] Vollmer M. (2004). Physics of the microwave oven. Phys. Educ..

[422] International Telecommunication Union www.itu.int/en/Pages/default.aspx.

[423] ITU-R V.li-8 (08/2015) Nomenclature of the Frequency and Wavelength Bands Used in Telecommunications. www.itu.int/rec/R-REC-V.431-8-201508-I/en.

[424] IEC (1988). Part 713: Radiocommunications: Transmitters, receivers, networks and operation. International Electrotechnical Vocabulary (IEV).

[425] Official Conference Website “World Radiocommunication Conferences (WRC)”. www.itu.int/en/ITU-R/conferences/wrc/Pages/default.aspx.

[426] Official Website of the Ministry of Infrastructure (Poland) www.gov.pl/web/infrastruktura.

[427] Torgovnikov G.I. (1993). Dielectric Properties of Wood and Wood-Based Materials.

[428] Rana K., Rana S. (2014). Microwave reactors: A brief review on its fundamental aspects and applications. Open Access Libr. J..

[429] Mohammadi E., Aliofkhazraei M., Hasanpoor M., Chipara M. (2018). Hierarchical and complex ZnO nanostructures by microwave-assisted synthesis: Morphologies, growth mechanism and classification. Crit. Rev. Solid State Mater. Sci..

[430] Bilecka I., Niederberger M. (2010). Microwave chemistry for inorganic nanomaterials synthesis. Nanoscale.

[431] Falciglia P.P., Roccaro P., Bonanno L., De Guidi G., Vagliasi F.G.A., Romano S. (2018). A review on the microwave heating as a sustainable technique for environmental remediation/detoxification applications. Renew. Sustain. Energy Rev..

[432] Gaba M., Dhingra N. (2011). Microwave chemistry: General features and applications. Indian J. Pharm. Educ. Res..

[433] Leadbeater N.E. (2011). Microwave Heating as a Tool for Sustainable Chemistry.

[434] Leadbeater N.E., McGowan C.B. (2013). Laboratory Experiments Using Microwave Heating.

[435] Kappe C.O. (2004). Controlled microwave heating in modern organic synthesis. Angew. Chem..

[436] Kappe C.O., Stadler A., Dallinger D. (2012). Microwaves in Organic and Medicinal Chemistry.

[437] Cravotto G., Carnaroglio D. (2017). Microwave Chemistry.

[438] Horikoshi S., Serpone N. (2013). Microwaves in Nanoparticle Synthesis: Fundamentals and Applications.

[439] Yang G., Park S.-J. (2019). Conventional and microwave hydrothermal synthesis and application of functional materials: A review. Materials.

[440] Yang G., Park S.-J. (2019). Author response to comment on: Conventional and microwave hydrothermal synthesis and application of functional materials: A review. Materials.

[441] Jalouli B., Abbasi A., Musavi Khoei S.M. (2019). A comment on: “Conventional and microwave hydrothermal synthesis and application of functional materials: A review”. Materials.

[442] Horikoshi S., Schiffmann R.F., Fukushima J., Serpone N. (2018). Microwave Chemical and Materials Processing.

[443] Lidstrom P., Tierney J., Wathey B., Westman J. (2001). Microwave assisted organic synthesis—A review. Tetrahedron.

[444] Feliczak-Guzik A., Górzyńska M., Zielińska A., Wawrzyńczak A. (2014). Promieniowanie mikrofalowe w laboratorium. Laborant.

[445] Chandrasekaran S., Ramanathan S., Basak T. (2013). Microwave food processing—A review. Food Res. Int..

[446] Feng H., Yin Y., Tang J. (2012). Microwave drying of food and agricultural materials: Basics and heat and mass transfer modeling. Food Eng. Rev..

[447] Vasudev H., Singh G., Bansal A., Vardhan S., Thakur L. (2019). Microwave heating and its applications in surface engineering: A review. Mater. Res. Express.

[448] Agrawal D. (2006). Microwave sintering of ceramics, composites and metallic materials, and melting of glasses. Trans. Indian Ceram. Soc..

[449] Oghbaei M., Mirzaee O. (2010). Microwave versus conventional sintering: A review of fundamentals, advantages and applications. J. Alloys Compd..

[450] Mishra R.R., Sharma A.K. (2016). A review of research trends in microwave processing of metal-based materials and opportunities in microwave metal casting. Crit. Rev. Solid State Mater. Sci..

[451] Singh S., Gupta D., Jain V., Sharma A.K. (2015). Microwave processing of materials and applications in manufacturing industries: A review. Mater. Manuf. Process..

[452] Leonelli C., Mason T.J. (2010). Microwave and ultrasonic processing: Now a realistic option for industry. Chem. Eng. Process..

[453] Sun J., Wang W., Yue Q. (2016). Review on microwave-matter interaction fundamentals and efficient microwave-associated heating strategies. Materials.

[454] Chavan R.S., Chavan S.R. (2010). Microwave baking in food industry: A review. Int. J. Dairy Sci..

[455] Bogdal D. (2005). Microwave-Assisted Organic Synthesis. One Hundred Reaction Procedures.

[456] Kim T., Lee J., Lee K.-H. (2014). Microwave heating of carbon-based solid materials. Carbon Lett..

[457] Jones D.A., Lelyveld T.P., Mavrofidis S.D., Kingman S.W., Mile N.J. (2002). Microwave heating applications in environmental engineering—A review. Resour Conserv. Recycl..

[458] Hayes B.L. (2002). Microwave Synthesis: Chemistry at the Speed of Light.

[459] Microwave-Assisted Synthesis. wiki.anton-paar.com/en/microwave-assisted-synthesis/.

[460] Sweygers N., Alewaters N., Dewil R., Appels L. (2018). Microwave effects in the dilute acid hydrolysis of cellulose to 5-hydroxymethylfurfural. Sci. Rep..

[461] Majcher A., Wiejak J., Przybylski J., Chudoba T., Wojnarowicz J. (2013). A novel reactor for microwave hydrothermal scale-up nanopowder synthesis. Int. J. Chem. React. Eng..

[462] Metaxas A.C. (1996). Foundations of Electroheat: A Unified Approach.

[463] General on Microwave Absorption—Examples of Penetration Depth. www.por.se/references/dieldata.html.

[464] Horikoshi S., Serpone N., de la Hoz A., Loupy A. (2013). Microwave frequency effects in organic synthesis. Microwaves in Organic Synthesis.

[465] Afolabi O.O.D., Sohail M. (2017). Microwaving human faecal sludge as a viable sanitation technology option for treatment and value recovery—A critical review. J. Environ. Manag..

[466] Fang Z., Smith R.L., Qi X. (2015). Production of Biofuels and Chemicals with Microwave, Biofuels and Biorefineries.

[467] Lojkowski W., Leonelli C., Chudoba T., Wojnarowicz J., Majcher A., Mazurkiewicz A. (2014). High-energy-low-temperature technologies for the synthesis of nanoparticles: Microwaves and high pressure. Inorganics.

[468] Schanche J.S. (2003). Microwave synthesis solutions from personal chemistry. Mol. Divers..

[469] Leonelli C., Lojkowski W. (2007). Main development directions in the application of microwave irradiation to the synthesis of nanopowders. Chem. Today.

[470] Wojnarowicz J., Chudoba T., Smoleń D., Łojkowski W., Majcher A. (2014). Mazurkiewicz, examples of the nanoparticles produced by microwave solvothermal synthesis (MSS) route. A. Glass Ceram..

[471] Shah J.J., Mohanraj K. (2014). Comparison of conventional and microwave-assisted synthesis of benzotriazole derivatives. Indian J. Pharm. Sci..

[472] Zhu Y.J., Wang W.W., Qi R.J., Hu X.L. (2004). Microwave- assisted synthesis of single-crystalline tellurium nanorods and nanowires in ionic liquids. Angew. Chem. Int. Ed..

[473] Li Q., Cao W., Lei J., Zhao X., Hou T., Fan B., Chen D., Zhang L., Wang H., Xu H. (2014). Synthesis and growth mechanism of ZnO rod-like nanostructures by a microwave-assisted low-temperature aqueous solution route. Cryst. Res. Technol..

[474] Rizzuti A., Leonelli C. (2008). Crystallization of aragonite particles from solution under microwave irradiation. Powder Technol..

[475] Stefanidis G., Stankiewicz A. (2016). Alternative Energy Sources for Green Chemistry.

[476] Ameta S.C., Punjabi P.B., Ameta R., Ameta C. (2015). Microwave-Assisted Organic Synthesis A Green Chemical Approach.

[477] Bhusnure O.G., Gholve S.B., Giram P.S., Warad T.A., Pangave V.S., Sangshetti J.N. (2015). Green approaches for the industrial production of active pharmaceutical ingredients. World J. Pharm. Res..

[478] Bose A.K., Manhas M.S., Ganguly S.N., Sharma A.H., Banik B.K. (2002). More chemistry for less pollution: Applications for process development. Synthesis.

[479] Kumar A., Kuang Y., Liang Z., Sun X. (2020). Microwave chemistry, recent advancements and eco-friendly microwave-assisted synthesis of nanoarchitectures and their applications: A review. Mater. Today Nano.

[480] Pal A. (2018). Microwave assisted synthesis: A green chemistry approach. Int. J. Eng. Sci. Invent..

[481] Huang J., Xu G., Liang Y., Hu G., Changa P. (2020). Improving coal permeability using microwave heating technology—A review. Fuel.

[482] Leonelli C., Veronesi P., Denti L., Gatto A., Iuliano L. (2008). Microwave assisted sintering of green metal parts. J. Mater. Process. Technol..

[483] Das S., Mukhopadhyay A.K., Datta S., Basu D. (2009). Prospects of microwave processing: An overview. Bull. Mater. Sci..

[484] Irving J., Cepeda M.F.J., Valdés F., Guerrero G. (2015). Microwave ablation: State-of-the-art review. OncoTargets Ther..

[485] Giberson R.T., Demaree R.S. (2001). Microwave Techniques and Protocols.

[486] Dworakowska S., Bogdal D., Prociak A. (2012). Microwave-assisted synthesis of polyols from rapeseed oil and properties of flexible polyurethane foams. Polymers.

[487] Dworakowska S., Bogdał D., Zaccheria F., Ravasio N. (2014). The role of catalysis in the synthesis of polyurethane foams based on renewable raw materials. Catal. Today.

[488] Bogdał D., Prociak A. (2007). Microwave-Enhanced Polymer Chemistry and Technology.

[489] Kempe K., Becer C.R., Schubert U.S. (2011). Microwave-assisted polymerizations: Recent status and future perspectives. Macromolecules.

[490] Adam D. (2003). Microwave chemistry: Out of the kitchen. Nature.

[491] Kappe C.O., Dallinger D. (2006). The impact of microwave synthesis on drug discovery. Nat. Rev. Drug Discov..

[492] Nagariya A.K., Meena A.K., Kiran K., Yadav A.K., Niranjan U.S., Pathak A.K., Singh B., Rao M.M. (2010). Microwave assisted organic reaction as new tool in organic synthesis. J. Pharm. Res..

[493] Jignasa K.S., Ketan T.S., Bhumika S.P., Anuradha K.G. (2010). Microwave assisted organic synthesis: An alternative synthetic strategy. Der Pharma Chem..

[494] Kłosowski G., Mikulski D., Menka A. (2019). Microwave-Assisted One-Step Conversion of Wood Wastes into Levulinic Acid. Catalysts.

[495] Díaz-Ortiz A., Prieto P., de la Hoz A. (2019). A critical overview on the effect of microwave irradiation in organic synthesis. Chem. Rec..

[496] Dąbrowska S., Chudoba T., Wojnarowicz J., Łojkowski W. (2018). Current trends in the development of microwave reactors for the synthesis of nanomaterials in laboratories and industries: A review. Crystals.

[497] Buttress A.J., Hargreaves G., Ilchev A., Monti T., Sklavounou A., Katrib J., Martin-Tanchereau P., Unthank M.G., Irvine D.J., Dodds C.D. (2019). Design and optimisation of a microwave reactor for kilo-scale polymer synthesis. Chem. Eng. Sci. X.

[498] Priecel P., Lopez-Sanchez J.A. (2019). Advantages and limitations of microwave reactors: From chemical synthesis to the catalytic valorization of biobased chemicals. ACS Sustain. Chem. Eng..

[499] Mitani T., Hasegawa N., Nakajima R., Shinohara N., Nozaki Y., Chikata T., Watanabe T. (2016). Development of a wideband microwave reactor with a coaxial cable structure. Chem. Eng. J..

[500] Leonelli C., Veronesi P., Fang Z., Smith R.L., Qi X. (2015). Microwave reactors for chemical synthesis and biofuels preparation. Production of Biofuels and Chemicals with Microwave.

[501] Kappe C.O. (2019). My twenty years in microwave chemistry: From kitchen ovens to microwaves that aren’t microwaves. Chem. Rec..

[502] Wojnarowicz J., Chudoba T., Gierlotka S., Lojkowski W. (2018). Effect of microwave radiation power on the size of aggregates of ZnO NPs prepared using microwave solvothermal synthesis. Nanomaterials.

[503] Dąbrowska S., Chudoba T., Wojnarowicz J., Łojkowski W. (2018). Problems of exploitations of microwave reactors for nanoparticles synthesis. J. Mach. Constr. Maint..

[504] Byrappaa K., Adschirib T. (2017). Hydrothermal technology for nanotechnology. Prog. Cryst. Growth Ch. Mater..

[505] Byrappa K., Yoshimura M. (2001). Handbook of Hydrothermal Technology.

[506] Komarneni S., Bruno M., Mariani E. (2000). Synthesis of ZnO with and without microwaves. Mater. Res. Bull..

[507] Pulit-Prociak J., Banach M. (2016). Effect of process parameters on the size and shape of nano- and micrometric zinc oxide. Acta Chim. Slov..

[508] Strachowski T., Grzanka E., Palosz B.F., Presz A., Ślusarski L., Łojkowski Ł. (2003). Microwave driven hydrothermal synthesis of zinc oxide nanopowders. Solid State Phenom..

[509] Hu Z.-L., Zhu Y.-J., Wang S.-W. (2004). Sonochemical and microwave-assisted synthesis of linked single-crystalline ZnO rods. Mater. Chem. Phys..

[510] Wang W.-W., Zhu Y.-J. (2004). Shape-controlled synthesis of zinc oxide by microwave heating using an imidazolium salt. Inorg. Chem. Commun..

[511] Abhulimen I.U., Das K., Rangari V.K., Bergman L., Chen X.-B., Morrison J.L. (2005). Sonochemical and microwave synthesis and characterization of ZnO nanoparticles. Proceedings of the Thirty-Seventh Southeastern Symposium on System Theory.

[512] Lu C.-H., Hwang W.-J., Godbole S.V. (2005). Microwave-hydrothermal synthesis and photoluminescence characteristics of zinc oxide powders. J. Mater. Res..

[513] Phuruangrat A., Thongtem T., Thongtem S. (2014). Controlling morphologies and growth mechanism of hexagonal prisms with planar and pyramid tips of ZnO microflowers by microwave radiation. Ceram. Int..

[514] Ivanov V.K., Shaporev A.S., Sharikov F.Y., Baranchikov A.Y. (2007). Hydrothermal and microwave-assisted synthesis of nanocrystalline ZnO photocatalysts. Superlattices Microst..

[515] Kong X., Duan Y., Peng P., Qiu C., Wu L., Liu L., Zheng W. (2007). A novel route to prepare ZnO nanotubes by using microwave irradiation method. Chem. Lett..

[516] Shaporev S., Ivanov V.K., Baranchikov A.E., Tret’yakov Y.D. (2007). Microwave-assisted hydrothermal synthesis and photocatalytic activity of ZnO. Inorg. Mater..

[517] Bhat D. (2008). Facile synthesis of ZnO nanorods by microwave irradiation of zinc–hydrazine hydrate complex. Nanoscale Res. Lett..

[518] Huang J., Xia C., Cao L., Zeng X. (2008). Facile microwave hydrothermal synthesis of zinc oxide one-dimensional nanostructure with three-dimensional morphology. Mater. Sci. Eng. B.

[519] Sadhukhan P., Kundu M., Rana S., Kumar R., Das J., Sil P.C. (2019). Microwave induced synthesis of ZnO nanorods and their efficacy as a drug carrier with profound anticancer and antibacterial properties. Toxicol. Rep..

[520] Marzouqi F., Al Adawi H., Qi K., Liu S.Y., Kim Y., Selvaraj R. (2019). A green approach to the microwave-assisted synthesis of flower-like ZnO nanostructures for reduction of Cr(VI). Toxicol. Environ. Chem..

[521] Ma M.G., Zhu Y.J., Cheng G.F., Huang Y.H. (2008). Microwave synthesis and characterization of ZnO with various morphologies. Mater. Lett..

[522] Goharshadi E.K., Ding Y., Nancarrow P. (2008). Green synthesis of ZnO nanoparticles in a room-temperature ionic liquid 1-ethyl-3-methylimidazolium bis(trifluoromethylsulfonyl)imide. J. Phys. Chem. Solids.

[523] Unalan H.E., Hiralal P., Rupesinghe N., Dalal S., Milne W.I., Amaratunga G.A.J. (2008). Rapid synthesis of aligned zinc oxide nanowires. Nanotechnology.

[524] Bayrami A., Ghorbani E., Pouran S.R., Habibi-Yangjeh A., Khataee A., Bayramia M. (2019). Enriched zinc oxide nanoparticles by Nasturtium officinale leaf extract: Joint ultrasound-microwave-facilitated synthesis, characterization, and implementation for diabetes control and bacterial inhibition. Ultrason. Sonochem..

[525] Cho S., Jung S.H., Lee K.H. (2008). Morphology-controlled growth of ZnO nanostructures using microwave irradiation: From basic to complex structures. J. Phys. Chem. C.

[526] Manavalan S., Veerakumar P., Chen S.-M., Lin K.-C. (2020). Three-dimensional zinc oxide nanostars anchored on graphene oxide for voltammetric determination of methyl parathion. Microchim. Acta.

[527] Chaudhuri T.K., Kothari A. (2009). Microwave-assisted chemical bath deposition of nanostructured ZnO particles. J. Nanosci. Nanotechnol..

[528] Sakata K., Macounová K.M., Nebel R., Krtil P. (2020). pH dependent ZnO nanostructures synthesized by hydrothermal approach and surface sensitivity of their photoelectrochemical behavior. SN Appl. Sci..

[529] Su X., Zhao H., Xiao F., Jian J., Wang J. (2012). Synthesis of flower-like 3D ZnO microstructures and their size-dependent ethanol sensing properties. Ceram. Int..

[530] Chen L., Xie H. (2009). Synthesis of ZnO microcrystals with controllable morphology by microwave reaction. 2009 Symposium on Photonics and Optoelectronics.

[531] Marković S., Stojković Simatović I., Sanita Ahmetović S., Veselinović L., Stojadinović S., Rac V., Škapin S.D., Bogdanović D.B., Častvang I.J., Uskoković D. (2019). Surfactant-assisted microwave processing of ZnO particles: A simple way for designing the surface-to-bulk defect ratio and improving photo(electro)catalytic properties. RSC Adv..

[532] Wannapop S., Somdee A., Thongtem T., Thongtem S. (2019). Synthesis of ZnO nanostructures by microwave irradiation for energy conversion material in for dye sensitized solar cells and materials for photocatalytic dye degradation applications. J. Ceram. Soc. Jap..

[533] Erten-Ela S., Cogal S., Icli S. (2009). Conventional and microwave-assisted synthesis of ZnO nanorods and effects of PEG400 as a surfactant on the morphology. Inorg. Chim. Acta.

[534] Rocha L.S.R., Foschini C.R., Silva C.C., Longo E., Simões A.Z. (2016). Novel ozone gas sensor based on ZnO nanostructures grown by the microwave-assisted hydrothermal route. Ceram. Int..

[535] Krishnakumar T., Jayaprakash R., Pinna N., Donato N., Bonavita A., Micali G., Neri G. (2009). CO gas sensing of ZnO nanostructures synthesized by an assisted microwave wet chemical route. Sens. Actuators B-Chem..

[536] Pauzi N., Zain N.M., Amira N., Yusof A. (2019). Gum arabic as natural stabilizing agent in green synthesis of ZnO nanofluids for antibacterial application. J. Environ. Chem. Eng..

[537] Pauzi N., Zain N.M., Amira N., Yusof A. (2019). Microwave-assisted synthesis of ZnO nanoparticles stabilized with gum arabic: Effect of microwave irradiation time on ZnO nanoparticles size and morphology. Bull. Chem. React. Eng. Catal..

[538] Krishnakumar T., Jayaprakash R., Pinna N., Singh V.N., Mehta B.R., Phani A.R. (2009). Microwave-assisted synthesis and characterization of flower shaped zinc oxide nanostructures. Mater. Lett..

[539] Min C., Shen X., Sheng W. (2009). Microwave-assisted aqueous synthesis of ultralong ZnO nanowires: Photoluminescence and photovoltaic performance for dye-sensitized solar cell. Appl. Phys. A.

[540] Padmanabhan S.C., Ledwith D., Pillai S.C., McCormack D.E., Kelly J.M. (2009). Microwave-assisted synthesis of ZnO micro-javelins. J. Mater. Chem..

[541] Phuruangrat A., Thongtem T., Thongtem S. (2009). Microwave-assisted synthesis of ZnO nanostructure flowers. Mater. Lett..

[542] Zhu J.Y., Zhang J.X., Zhou H.F., Qin W.Q., Chai L.Y., Hu Y.H. (2009). Microwave-assisted synthesis and characterization of ZnO-nanorod arrays. Trans. Nonferrous Met. Soc. China.

[543] Wu S.S., Jia Q.M., Sun Y.L., Shan S.Y., Jiang L.H., Wang Y.M. (2012). Microwave-hydrothermal preparation of flower-like ZnO microstructure and its photocatalytic activity. Trans. Nonferrous Met. Soc. China.

[544] Boudjadar S., Achour S., Boukhenoufa N., Guerbous L. (2010). Microwave hydrothermal synthesis and characterization of ZnO nanosheets. Int. J. Nanosci..

[545] Kajbafvala A., Zanganeh S., Kajbafvalac K., Zargarc H.R., Bayatic M.R., Sadrnezhaad S.K. (2010). Microwave-assisted synthesis of narcis-like zinc oxide nanostructures. J. Alloys Compd..

[546] Lee Y.C., Yang C.S., Huang H.J., Hu S.Y., Lee J.W., Cheng C.F., Huang C.C., Tsai M.K., Kuang H.C. (2010). Structural and optical properties of ZnO nanopowder prepared by microwave-assisted synthesis. J. Lumin..

[547] De Moura A.P., Lima R.C., Moreira M.L., Volanti D.P., Espinosa J.W.M., Orlandi M.O., Pizani P.S., Varela J.A., Longo E. (2010). ZnO architectures synthesized by a microwave-assisted hydrothermal method and their photoluminescence properties. Solid State Ion..

[548] Shojaee N., Ebadzadeh T., Aghaei A. (2010). Effect of concentration and heating conditions on microwave-assisted hydrothermal synthesis of ZnO nanorods. Mater. Charact..

[549] Shojaee N., Ebadzadeh T., Aghaei A. (2012). Microwave assisted hydrothermal synthesize of ZnO nanorods and their characterization. Int. J. Mod. Phys. Conf. Ser..

[550] Thongtem T., Phuruangrat A., Thongtem S. (2010). Characterization of nanostructured ZnO produced by microwave irradiation. Ceram. Int..

[551] Al-Gaashani R., Radiman S., Tabet N., Razak Daud A. (2011). Effect of microwave power on the morphology and optical property of zinc oxide nano-structures prepared via a microwave-assisted aqueous solution method. Mater. Chem. Phys..

[552] Chen Y.C., Lo S.L. (2011). Effects of operational conditions of microwave-assisted synthesis on morphology and photocatalytic capability of zinc oxide. Chem. Eng. J..

[553] Kathalingam A., Chae Y.S., Rhee J.K. (2011). Synthesis of multi-linked ZnO rods by microwave heating. Cryst. Res. Technol..

[554] Deng C.H., Hu H.M., Han C.L., Yang B.H., Shao G.Q. (2011). Shape-controlled synthesis of zinc oxide spherical structures by microwave-assisted chemical aqueous refluxing process. Asian J. Chem..

[555] Hamedani N.F., Mahjoub A.R., Khodadadi A.A., Mortazavi Y., Farzaneh F. (2011). Microwave assisted fast synthesis of flower-like ZnO based guanidinium template for photodegradation of azo dye congo red. Int. J. Chem. Mol. Nucl. Mater. Met. Eng..

[556] Kondawar S.B., Acharya S.A., Dhakate S.R. (2011). Microwave assisted hydrothermally synthesized nanostructure zinc oxide reinforced polyaniline nanocomposites. Adv. Mater. Lett..

[557] Kothari A., Chaudhuri T.K. (2011). One-minute microwave-assisted chemical bath deposition of nanostructured ZnO rod-arrays. Mater. Lett..

[558] Krishnakumar T., Jayaprakash R., Sathya Raj D., Pinna N.P., Singh V.N., Phani A.R., Neri G. (2011). Microwave-assisted synthesis, characterization and ammonia sensing properties of polymer-capped star-shaped zinc oxide nanostructures. J. Nanopart. Res..

[559] Liu H., Zhu F., Yang D., Sun H.J. (2011). Microwave assisted hydrothermal synthesis of hierarchical structured ZnO nanorods. Mater. Technol..

[560] Sharma D., Sharma S., Kaith B.S., Rajputa J., Kaur M. (2011). Synthesis of ZnO nanoparticles using surfactant free in-air and microwave method. Appl. Surf. Sci..

[561] Jugong Z., Ting Y. (2011). Microwave assisted synthesis of ZnO Calthrop-like Nanostructures in Ionic-Liquid. Adv. Mater. Res..

[562] Zhu Z., Yang D., Liu H. (2011). Microwave-assisted hydrothermal synthesis of ZnO rod-assembled microspheres and their photocatalytic performances. Adv. Powder Technol..

[563] Baghbanzadeh M., Skapin S.D., Orel Z.C., Kappe C.O. (2012). A critical assessment of the specific role of microwave irradiation in the synthesis of ZnO micro- and nanostructured materials. Chem. Eur. J..

[564] Chen W., Yu D., Ruan H., Li D., Hu Y., Chen Y., He Y., Fu X., Shao Y. (2012). Microwave-assisted rapid synthesis of zno hexagonal quasi-hourglasses. J. Am. Ceram. Soc..

[565] Guo L.T., Wu J., Guo L.Z., Zhu Y.B., Xu C., Qiang Y.H. (2012). Microwave hydrothermal synthesis and characterization of ZnO nanostructures in aqueous solution. J. Shanghai Jiaotong Univ. (Sci.).

[566] Mohajerani M.S., Mazloumi M., Lak A., Kajbafvala A., Zanganeh S., Sadrnezhaad S.K. (2008). Self-assembled zinc oxide nanostructures via a rapid microwave-assisted route. J. Cryst. Growth.

[567] Hassana J.J., Mahdi M.A., Chin C.W., Hassan Z., Abu-Hassan H. (2012). Microwave assisted chemical bath deposition of vertically aligned ZnO nanorods on a variety of substrates seeded by PVA–Zn(OH)_2_ nanocomposites. Appl. Surf. Sci..

[568] Hassan J.J., Hassan Z., Abu-Hassan H. (2011). High-quality vertically aligned ZnO nanorods synthesized by microwave-assisted CBD with ZnO–PVA complex seed layer on Si substrates. J. Alloys Compd..

[569] Hassan J.J., Mahdi M.A., Chin C.W., Abu-Hassan H., Hassan Z. (2013). Room temperature hydrogen gas sensor based on ZnO nanorod arrays grown on a SiO_2_/Si substrate via a microwave-assisted chemical solution method. J. Alloys Compd..

[570] Baruah S., Mahmood M.A., Myint M.T.Z., Bora T., Dutta J. (2010). Enhanced visible light photocatalysis through fast crystallization of zinc oxide nanorods. Beilstein J. Nanotechnol..

[571] Jiang Q., Liu Y., Kan H., Yuan B., Zhao H. (2012). Microwave-assisted synthesis of hexagonal structure ZnO micro-tubes. Mater. Lett..

[572] Byzynski G., Pereira A.P., Volanti D.P., Ribeiro C., Longo E. (2018). High-performance ultraviolet-visible driven ZnO morphologies photocatalyst obtained by microwave-assisted hydrothermal method. J. Photoch. Photobiol. A-Chem..

[573] Kajbafvala A., Ghorbani H., Paravar A., Joshua P., Samberg J.P., Kajbafvala E., Sadrnezhaad S.K. (2012). Effects of morphology on photocatalytic performance of Zinc oxide nanostructures synthesized by rapid microwave irradiation methods. Superlattice. Microst..

[574] Klofac J., Munster L., Bazant P., Sedlak J., Kuritka I. (2012). Preparation of Flower-Like ZnO Microparticles by Microwave Assisted Synthesis.

[575] Liu X., Lv T., Pan L., Sun Z., Sun C.Q. (2012). Microwave-assisted synthesis of ZnO for photocatalytic reduction of Cr(VI) in aqueous solution. Desalin. Water Treat..

[576] Rai P., Song H.M., Kim Y.S., Song M.K., Oh P.R., Yoon J.M., Yu Y.T. (2012). Microwave assisted hydrothermal synthesis of single crystalline ZnO nanorods for gas sensor application. Mater. Lett..

[577] Sahoo S., Barik S.K., Gaur A.P.S., Correa M., Singh G., Katiyar R.K., Puli V.S., Liriano J., Katiyar R.S. (2012). Microwave assisted synthesis of ZnO nano-sheets and their application in UV-detector. ECS J. Solid State Sci. Technol..

[578] Sapnar K.B., Ghule L.A., Bankar A., Zinjarde S., Bhoraskar V.N., Garadkar K.M., Dhole S.D. (2012). Antimicrobial activity of 6.5 MeV electron-irradiated ZnO nanoparticles synthesized by microwave-assisted method. Int. J. Green Nanotechnol..

[579] Xavier C.S., de Moura A.P., Li M.S., Varela J.A., Longo E., Cava S. (2012). Microwave-assisted hydrothermal synthesis of ZnO powders with different reagents. TechConnect Briefs.

[580] Tseng C.C., Chou Y.H., Liu C.M., Liu Y.M., Ger M.D., Shu Y.Y. (2012). Microwave-assisted hydrothermal synthesis of zinc oxide particles starting from chloride precursor. Mater. Res. Bull..

[581] Barreto G.P., Morales G., Quintanilla Ma L.L. (2013). Microwave assisted synthesis of ZnO nanoparticles: Effect of precursor reagents, temperature, irradiation time, and additives on nano-ZnO morphology development. J. Mater..

[582] Liu D. (2013). Technical study on microwave method for the preparation of flower-like ZnO. Adv. Mater. Res..

[583] Majithia R., Speich J., Meissner K.E. (2013). Mechanism of generation of ZnO microstructures by microwave-assisted hydrothermal approach. Materials.

[584] Ogata K., Koike K., Sasa S., Inoue M., Yano M. (2013). ZnO nanorod growth from aqueous solution via microwave heating on paper substrates. Phys. Status Solidi C.

[585] Prakash T., Jayaprakash R., Neri G., Kumar S. (2013). Synthesis of ZnO nanostructures by microwave irradiation using albumen as a template. J. Nanopart. Res..

[586] Ridha N.J., Umar A.A., Alosfur F., Jumali M.H., Salleh M.M. (2013). Microwave assisted hydrothermal method for porous zinc oxide nanostructured-films. J. Manosci. Nanotechnol..

[587] Ridha N.J., Jumali M.H.H., Umar A.A., Mohama F.K. Ethanol sensor based on ZnO nanostructures prepared via microwave oven. Proceedings of the 2013 Seventh International Conference on Sensing Technology (ICST).

[588] Tong L., Liu Y., Rong H., Gong L. (2013). Microwave-assisted synthesis of hierarchical ZnO nanostructures. Mater. Lett..

[589] Tan S.T., Umar A.A., Yahaya M., Yap C.C., Salleh M.M. (2013). Ultrafast formation of ZnO nanorods via seed-mediated microwave assisted hydrolysis process. J. Phys. Conf. Ser..

[590] Tan S.T., Tan C.H., Chong Y., Yap C.C., Umar A.A., Ginting R.T., Lee H.B., Lim K.S., Yahayaa M., Salleh M.M. (2016). Microwave-assisted hydrolysis preparation of highly crystalline ZnO nanorod array for room temperature photoluminescence-based CO gas sensor. Sens. Actuators B-Chem..

[591] Yahaya M., Tan S.T., Umar A.A., Yap C.C., Salleh M.M. (2014). Synthesis of ZnO nanorod arrays by chemical solution and microwave method for sensor application. Key Eng. Mater..

[592] Ridha N.J., Alosfur F.K.M., Hafizuddin Haji Jumali M., Radiman S. (2020). Effect of Al thickness on the structural and ethanol vapor sensing performance of ZnO porous nanostructures prepared by microwave-assisted hydrothermal method. Nanotechnology.

[593] Liang S., Zhu L., Gai G., Yao Y., Huang J., Ji X., Zhou X., Zhang D., Zhang P. (2014). Synthesis of morphology-controlled ZnO microstructures via a microwave-assisted hydrothermal method and their gas-sensing property. Ultrason. Sonochem..

[594] Pimentel A., Nunes D., Duarte P., Rodrigues J., Costa F.M., Monteiro T., Martins R., Fortunato E. (2014). Synthesis of long ZnO nanorods under microwave irradiation or conventional heating. J. Phys. Chem. C.

[595] Singh N.P., Shivashankar S.A., Pratap R. (2014). In Defect driven emission from ZnO nano rods synthesized by fast microwave irradiation method for optoelectronic applications. Mater. Res. Soc. Symp. Proc..

[596] Sujaridworakun P., Natrchalayuth K. (2014). Photocatalytic performance of ZnO nanoparticles synthesized by microwave-assisted process using zinc-dust waste as a starting material. Key Eng. Mater..

[597] Yu H., Fan H., Wang X., Wang J. (2014). Synthesis and characterization of ZnO microstructures via microwaveassisted hydrothermal synthesis process. Optik.

[598] Barreto G., Morales G., Cañizo A., Eyler N. (2015). Microwave assisted synthesis of ZnO tridimensional nanostructures. Procedia Mater. Sci..

[599] Hasanpoora M., Aliofkhazraeia M., Delavaria H. (2015). Microwave-assisted synthesis of zinc oxide nanoparticles. Procedia Mater. Sci..

[600] Ocakoglu K., Mansour S.A., Yildirimcan S., Al-Ghamdi A.A., El-Tantawy F., Yakuphanoglu F. (2015). Microwave- assisted hydrothermal synthesis and characterization of ZnO nanorods. Spectrochim. Acta-Part A Mol. Biomol. Spectrosc..

[601] Promnopas W., Thongtem T., Thongtem S. (2015). Effect of microwave power on energy gap of ZnO nanoparticles synthesized by microwaving through aqueous solutions. Superlattices Microstruct..

[602] Witkowski S., Wachnicki L., Gierałtowska S., Dluzewski P., Szczepanska A., Kaszewski J., Godlewski M. (2014). Ultra-fast growth of the monocrystalline zinc oxide nanorods from the aqueous solution. Int. J. Nanotechnol..

[603] Witkowski S.B., Dluzewski P., Kaszewski J., Wachnicki L., Gieraltowska S., Kurowska B., Godlewski M. (2018). Ultra-fast epitaxial growth of ZnO nano/microrods on a GaN substrate, using the microwave-assisted hydrothermal method. Mater. Chem. Phys..

[604] Tang J., Chai J., Huang J., Deng L., Nguyen X.S., Sun L., Venkatesan T., Shen Z., Tay C.B., Chua S.J. (2015). ZnO nanorods with low intrinsic defects and high optical performance grown by facile microwave-assisted solution method. ACS Appl. Mater. Interfaces.

[605] Husham M., Hamidon M.N., Paiman S., Abuelsamen A.A., Farhat O.F., Al-Dulaimi A.A. (2017). Synthesis of ZnO nanorods by microwave-assisted chemical-bath deposition for highly sensitive self-powered UV detection application. Sens. Actuators A-Phys..

[606] Sun F., Zhao Z., Qiao X., Tan F., Wang W. (2016). Microwave synthesis and photocatalytic activities of ZnO bipods with different aspect ratios. Mater. Res. Bull..

[607] Matias M.L., Nunes D., Pimentel A., Ferreira S.H., d’Agua R.B., Duarte M.P., Fortunato E., Martins R. (2019). Paper-based nanoplatforms for multifunctional applications. J. Nanomater..

[608] Gray R.J., Jaafar A.H., Verrelli E., Kemp N.T. (2018). Method to reduce the formation of crystallites in ZnO nanorod thin-films grown via ultra-fast microwave heating. Thin Solid Films.

[609] Ridwan M., Fauzia V., Roza L. (2019). Synthesis and characterization of ZnO nanorods prepared using microwave-assisted hydrothermal method. IOP Conf. Ser. Mater. Sci. Eng..

[610] Shinde V.R., Gujar T.P., Noda T., Fujita D., Vinu A., Grandcolas M., Ye J. (2010). Growth of shape- and size-selective zinc oxide nanorods by a microwave- assisted chemical bath deposition method: Effect on photocatalysis properties. Chem. Eur. J..

[611] Fang M., Liu Z.W. (2017). Controllable size and photoluminescence of ZnO nanorod arrays on Si substrate prepared by microwave-assisted hydrothermal method. Ceram. Int..

[612] Mosayebi E., Azizian S., Hajian A. (2015). Synthesis of nanostructured and microstructured ZnO and Zn(OH)2 on activated carbon cloth by hydrothermal and microwave-assisted chemical bath deposition methods. Superlattice Microst..

[613] Thamima T., Karuppuchamy S. (2015). Microwave assisted synthesis of zinc oxide nanoparticles. Int. J. Chem. Tech. Res..

[614] Khaghani S., Ghanbari B. (2016). Microwave synthesis of Fe_2_O_3_ and ZnO nanoparticles and evaluation its application on grain iron and zinc concentrations of wheat (triticum aestivum L.) and their relationships to grain yield. J. Nanostruct..

[615] Ou M., Ma L., Xu L., Li X., Yang Z., Lan Z. (2016). Microwave-assisted synthesis of hierarchical ZnO nanostructures and their photocatalytic properties. MATEC Web Conf..

[616] Mostafa N.Y., Heiba Z.K., Ibrahim M.M. (2015). Structure and optical properties of ZnO produced from microwave hydrothermal hydrolysis of tris(ethylenediamine)zinc nitrate complex. J. Mol. Struct..

[617] Pimentel A., Ferreira S.H., Nunes D., Calmeiro T., Martins R., Fortunato E. (2016). Microwave synthesized ZnO nanorod arrays for UV sensors: A seed layer annealing temperature study. Materials.

[618] Soleimanzadeh R., Mousavi M.S.S., Mehrfar A., Esfahanic Z.K., Kolahdouz M., Zhang K. (2015). Sequential microwave-assisted ultra-fast ZnO nanorod growth on optimized sol–gel seedlayers. J. Cryst. Growth.

[619] Thi V.H.T., Lee B.K. (2017). Great improvement on tetracycline removal using ZnO rod-activated carbon fiber composite prepared with a facile microwave method. J. Hazard. Mater..

[620] Quirino M.R., Oliveira M.J.L., Keyson D., Lucena G.L., Oliveira J.B.L., Gama L. (2017). Synthesis of zinc oxide by microwave hydrothermal method for application to transesterification of soybean oil (biodiesel). Mater. Chem. Phys..

[621] Rana A.U., Kang M., Kim H.S. (2016). Microwave-assisted facile and ultrafast growth of ZnO nanostructures and proposition of alternative microwave-assisted methods to address growth stoppage. Sci. Rep..

[622] Saravanan K., Rajathi K. (2016). Microwave-assisted synthesis of ZnO nanoparticles from 1, 2, 3 tri substituted ionic liquid and their antimicrobial and biofilm activities. Int. J. Chem. Sci..

[623] Bayrami A., Parvinroo S., Habibi-Yangjeh A., Rahim Pouran S. (2018). Bio-extract-mediated ZnO nanoparticles: Microwave-assisted synthesis, characterization and antidiabetic activity evaluation. Artif. Cells Nanomed. Biotechnol..

[624] Pimentel A., Samouco A., Nunes D., Araújo A., Martins R., Fortunato E. (2017). Ultra-fast microwave synthesis of ZnO nanorods on cellulose substrates for UV sensor applications. Materials.

[625] Song H., Zhu K., Liu Y., Zhai X. (2017). Microwave-assisted synthesis of ZnO and its photocatalytic activity in degradation of CTAB. Russ. J. Phys. Chem. A.

[626] Sun H., Sun L., Sugiura T., White M.S., Stadler P., Sariciftci N.S., Masuhara A., Yoshida T. (2017). Microwave-assisted hydrothermal synthesis of struture-controlled ZnO nanocrystals and their properties in dye-sensitized solar cells. Electrochemistry.

[627] Chauhan D.S., Gopal C.S.A., Kumar D., Mahato N., Quraishi M.A., Chod M.H. (2018). Microwave induced facile synthesis and characterization of ZnO nanoparticles as efficient antibacterial agents. Mater. Discov..

[628] Goswami S.R., Singh M. (2018). Microwave-mediated synthesis of zinc oxide nanoparticles: A therapeutic approach against Malassezia species. IET Nanobiotechnol..

[629] Hirai Y., Furukawa K., Sun H., Matsushima Y., Shito K., Masuhara A., Ono R., Shimbori Y., Shiroishi H., Schuette White M. (2018). Microwave-assisted hydrothermal synthesis of ZnO and Zn-terephthalate hybrid nanoparticles employing benzene dicarboxylic acids. Microsyst. Technol..

[630] Giridhar M., Naik H.S.B., Sudhamani C.N., Prabakara M.C., Kenchappa R., Venugopal N., Patil S. (2019). Microwave-assisted synthesis of water-soluble styrylpyridine dye-capped zinc oxide nanoparticles for antibacterial applications. J. Chin. Chem. Soc..

[631] Rana A.U.H.S., Chang S.B., Kim H.S. (2018). NH₄OH-Oriented and pH-Dependent growth of ZnO nanostructures via microwave-assisted growth method. J. Nanosci. Nanotechnol..

[632] Chae H.U., Rana A.U.H.S., Park Y.J., Kim H.S. (2018). High-speed growth of ZnO nanorods in preheating condition using microwave-assisted growth method. J. Nanosci. Nanotechnol..

[633] Ahmed F., Arshi N., Anwar M.S., Danish R., Koo B.H. (2013). Facile growth of ZnO nanorod arrays by a microwave-assisted solution method for oxygen gas sensing. Thin Solid Films.

[634] Sutradhar P., Debbarma M., Saha M. (2016). Microwave synthesis of zinc oxide nanoparticles using coffee powder extract and its application for solar cell. Syn. React. Inorg. Metal.-Org. Nano-Met. Chem..

[635] Sutradhar P., Saha M. (2016). Green synthesis of zinc oxide nanoparticles using tomato (Lycopersicon esculentum) extract and its photovoltaic application. J. Exp. Nanosci..

[636] Sutradhar P., Saha M. (2015). Synthesis of zinc oxide nanoparticles using tea leaf extract and its application for solar cell. Bull. Mater. Sci..

[637] Chankaew C., Tapala W., Grudpan K., Rujiwatra A. (2019). Microwave synthesis of ZnO nanoparticles using longan seeds biowaste and their efficiencies in photocatalytic decolorization of organic dyes. Environ. Sci. Poll. Res..

[638] Rakkesh R.A., Durgalakshmi D., Karthe P., Balakumar S. (2020). Anisotropic growth and strain-induced tunable optical properties of Ag-ZnO hierarchical nanostructures by a microwave synthesis method. Mater. Chem. Phys..

[639] Saberon S.I., Maguyon-Detras M.C., Migo M.V.P., Marvin U., Herrera M.U., Manalo R.D. (2018). Microwave-assisted synthesis of zinc oxide nanoparticles on paper. Key Eng. Mater..

[640] Moloto N., Mpelane S., Sikhwivhilu L.M., Ray S.S. (2012). Optical and morphological properties of ZnO- and TiO_2_-derived nanostructures synthesized via a microwave-assisted hydrothermal method. Int. J. Photoenergy.

[641] Yanga L.Y., Donga S.Y., Suna J.H., Fenga J.L., Wua Q.H., Sun S.P. (2010). Microwave-assisted preparation, characterization and photocatalytic properties of a dumbbell-shaped ZnO photocatalyst. J. Hazard. Mater..

[642] Ahmed F., Kumar S., Arshi N., Anwar M.S., Koo B.H., Lee C.G. (2011). Rapid and cost effective synthesis of ZnO nanorods using microwave irradiation technique. Funct. Mater. Lett..

[643] Ahmed F., Kumar S., Arshi N., Anwar P.R. (2011). Growth and characterization of ZnO nanorods by microwave-assisted route: Green chemistry approach. Adv. Mater. Lett..

[644] Hamedani N.F., Mahjoub A.R., Khodadadi A.A., Mortazavi Y. (2011). Microwave assisted fast synthesis of various ZnO morphologies for selective detection of CO, CH_4_ and ethanol. Sens. Actuators B-Chem..

[645] Hamedani N.F., Farzaneh F. (2006). Synthesis of ZnO nanocrystals with hexagonal (wurtzite) structure in water using microwave irradiation. J. Sci. I. R. Iran..

[646] Mahpeykar S.M., Koohsorkhi J., Ghafoori-fard H. (2012). Ultra-fast microwave-assisted hydrothermal synthesis of long vertically aligned ZnO nanowires for dye-sensitized solar cell application. Nanotechnology.

[647] Vijayalakshmi K., Karthick K. (2012). Influence of annealing on the photoluminescence of nanocrystalline ZnO synthesized by microwave processing. Philos. Mag. Lett..

[648] Jaafar N.F., Najman A.M.M., Marfur A., Jusoh N.W.C. (2020). Strategies for the formation of oxygen vacancies in zinc oxide nanoparticles used for photocatalytic degradation of phenol under visible light irradiation. J. Photoch. Photobiol. A-Chem..

[649] Al-Gaashani R., Radiman S., Daud A.R., Tabet N., Al-Dourid Y. (2013). XPS and optical studies of different morphologies of ZnO nanostructures prepared by microwave methods. Ceram. Int..

[650] Al-Hazmi F., Abdel Aal N., Al-Ghamdi A.A., Alnowaiser F., Gafer Z.H., Al-Sehemi A.G., El-Tantawy F., Yakuphanoglu F. (2013). Facile green synthesis, optical and photocatalytic properties of zinc oxide nanosheets via microwave assisted hydrothermal technique. J. Electroceram..

[651] Papadaki D., Foteinis S., Mhlongo G.H., Nkosi S.S., Motaung D.E., Ray S.S., Tsoutsos T., Kiriakidis G. (2017). Life cycle assessment of facile microwave-assisted zinc oxide (ZnO) nanostructures. Sci. Total Environ..

[652] Li X., Sun P., Yang T., Zhao J., Wang Z., Wang W., Liu Y., Lu G., Du Y. (2013). Template-free microwave-assisted synthesis of ZnO hollow microspheres and their application in gas sensing. CrystEngComm.

[653] Sangari N.U., Devi S.C. (2013). Synthesis and characterization of nano ZnO rods via microwave assisted chemical precipitation method. J. Solid State Chem..

[654] Gu F., You D., Wang Z., Han D., Guo G. (2014). Improvement of gas-sensing property by defect engineering in microwave-assisted synthesized 3D ZnO nanostructures. Sens. Actuators B-Chem..

[655] Li X., Wang C., Zhou X., Liu J., Sun P., Lu G. (2016). Gas sensing properties of flower-like ZnO prepared by a microwave-assisted technique. RSC Adv..

[656] Li X., Zhou X., Liu Y., Sun P., Shimanoe K., Yamazoe N., Lu G. (2014). Microwave hydrothermal synthesis and gas sensing application of porous ZnO core–shell microstructures. RSC Adv..

[657] Shindea V.V., Dalavi D.S., Malic S.S., Hong C.K., Kim J.H., Patil P.S. (2014). Surfactant free microwave assisted synthesis of ZnO microspheres: Study of their antibacterial activity. Appl. Surf. Sci..

[658] Caglar Y., Gorgun K., Aksoy S. (2015). Effect of deposition parameters on the structural properties of ZnO nanopowders prepared by microwave-assisted hydrothermal synthesis. Spectrochim. Acta A.

[659] Al-Sabahi J., Bora T., Al-Abri M., Dutta J. (2016). Controlled defects of zinc oxide nanorods for efficient visible light photocatalytic degradation of phenol. Materials.

[660] Salah N., AL-Shawafi W.M., Alshahrie A., Baghdadi N., Soliman Y.M., Memic A. (2019). Size controlled, antimicrobial ZnO nanostructures produced by the microwave assisted route. Mater. Sci. Eng. C.

[661] Shingange K., Mhlongo G.H., Motaung D.E., Ntwaeaborwa O.M. (2016). Tailoring the sensing properties of microwave-assisted grown ZnO nanorods: Effect of irradiation time on luminescence and magnetic behavior. J. Alloys Compd..

[662] Kumar P.N., Sakthivel K., Balasubramanian V. (2017). Microwave assisted biosynthesis of rice shaped ZnO nanoparticles using Amorphophallus konjac tuber extract and its application in dye sensitized solar cells. Mater. Sci.-Pol..

[663] Tong L., Li W., Shen O., Rong H., Gong L. (2018). Microwave-assisted the facile synthesis and photocatalytic properties of rhombic ZnO microstructures. Mater. Lett..

[664] Nandi A., Nag P., Saha H., Majumdar S. (2018). Precursor dependent morphologies of microwave assisted ZnO nanostructures and their VOC detection properties. Mater. Today Proc..

[665] Wolska E., Sibera D., Witkowski B.S., Yatsunenko S.A., Pelech I., Narkiewicz U., Godlewski M. (2011). Photoluminescence and chromaticity properties of ZnO nanopowders made by a microwave hydrothermal method. Acta Phys. Pol. A.

[666] Fu L.H., Dong Y.Y., Ma M.G., Li S.M., Sun S.L., Sun R.C. (2013). Zn_5_(OH)_8_Cl_2_·H_2_O sheets formed using cellulose as matrix via microwave-assisted method and its transformation to ZnO. Mater. Lett..

[667] Somsri S., Sonwaew W., Rujiwatra A. (2016). Psidium guajava Linn extract mediated microwave synthesis and photocatalytic activities of ZnO nanoparticles. Mater. Lett..

[668] Vahidi A., Vaghari H., Najian Y., Najian M.N., Jafarizadeh-Malmiri H. (2018). Evaluation of three different green fabrication methods for the synthesis of crystalline ZnO nanoparticles using Pelargonium zonale leaf extract. Green Process. Synth..

[669] Dutta S., Jaiswal K.K., Verma R., Basavaraju D.M., Ramaswamy A.P. (2019). Green synthesis of zinc oxide catalyst under microwave irradiation using banana (Musa spp.) corm (rhizome) extract for biodiesel synthesis from fish waste lipid. Biocatal. Agric. Biotechnol..

[670] Thankachan R.M., Joy N., Abrahama J., Kalarikkal K., Thomas S., Oluwafemi O.S. (2017). Enhanced photocatalytic performance of ZnO nanostructures produced via a quick microwave assisted route for the degradation of rhodamine in aqueous solution. Mater. Res. Bull..

[671] Zhu L., Zheng Y., Hao T., Shi X., Chen Y., Ou-Yang J. (2009). Synthesis of hierarchical ZnO nanobelts via Zn(OH)F intermediate using ionic liquid-assistant microwave irradiation method. Mater. Lett..

[672] Malik L.A., Bashir A., Manzoor T., Pandith A.H. (2019). Microwave-assisted synthesis of glutathionecoated hollow zinc oxide for the removal of heavy metal ions from aqueous systems. RSC Adv..

[673] Sooksaen P., Chuankrerkkul N. (2016). Morphology-design and semiconducting characteristics of zinc oxide nanostructures under microwave irradiation. Integr. Ferroelectr..

[674] Singh A.K. (2010). Microwave synthesis, optical, structural and magnetic characterization of ZnO/Mn doped ZnO nanoparticles. J. Optoelectron. Adv. Mater..

[675] Hadzic B., Romcevic N., Romcevic N., Kuryliszyn-Kudelska I., Dobrowolski W., Narkiewicz U., Sibera D. (2016). Raman study of surface optical phonons in hydrothermally obtained ZnO(Mn) nanoparticles. Opt. Mater..

[676] Konicki W., Sibera D., Narkiewicz U. (2018). Adsorptive removal of cationic dye from aqueous solutions by ZnO/ZnMn2O4 nanocomposite. Separ. Sci. Technol..

[677] Konicki W., Sibera D., Narkiewicz U. (2017). Adsorption of acid red 88 anionic dye from aqueous solution onto ZnO/Mn2O4 nanocomposite: Equilibrium, kinetics, and thermodynamics. Pol. J. Environ. Stud..

[678] Limaye M.V., Singh S.B., Das R., Poddar P., Kulkarni S.K. (2011). Room temperature ferromagnetism in undoped and Fe doped ZnO nanorods: Microwave-assisted synthesis. J. Solid State Chem..

[679] Kwong T.L., Yung K.F. (2015). Surfactant-free microwave-assisted synthesis of Fe-doped ZnO nanostars as photocatalyst for degradation of tropaeolin O in water under visible light. J. Nanomater..

[680] Konicki W., Sibera D., Narkiewicz U. (2017). Removal of Rhodamine B from aqueous solution by ZnFe_2_O_4_ nanocomposite with magnetic separation performance. Pol. J. Chem. Technol..

[681] Sibera D., Jedrzejewski R., Mizeracki J., Presz A., Narkiewicz U., Lojkowski W. (2009). Synthesis and characterization of ZnO doped with Fe_2_O_3_—Hydrothermal synthesis and calcination process. Acta Phys. Pol. A.

[682] Guskos N., Typek J., Zolnierkiewicz G., Wardal K., Sibera D., Narkiewicz U. (2011). Magnetic resonance study of nanocrystalline ZnO nanopowders doped with Fe_2_O_3_ obtained by hydrothermal synthesis. Rev. Adv. Mater. Sci..

[683] Typek J., Wardal K., Zolnierkiewicz G., Guskos N., Narkiewicz U., Bonca J., Kruchinin S. (2015). Magnetic properties of Fe_2_O_3_/ZnO nanocomposites. Nanotechnology in the Security Systems.

[684] Kuryliszyn-Kudelska I., Hadzic B., Sibera D., Kilanski L., Romcevic N., Romcevic M., Narkiewicz U., Dobrowolski W. (2011). Dynamic magnetic properties of ZnO nanocrystals incorporating Fe. J. Alloys Compd..

[685] Konicki W., Sibera D., Mijowska E., Lendzion-Bieluń Z., Narkiewicz U. (2013). Equilibrium and kinetic studies on acid dye Acid Red 88 adsorption by magnetic ZnFe_2_O_4_ spinel ferrite nanoparticles. J. Colloid Interface Sci..

[686] Rezaei M., Habibi-Yangjeh A. (2013). Microwave-assisted preparation of Ce-doped ZnO nanostructures as an efficient photocatalyst. Mater. Lett..

[687] Shulga A., Butusov L.A., Chudinova G.K., Sheshko T.F. (2020). Microwave-assisted synthesis of cerium doped ZnO nanostructures and its optical properties. J. Phys. Conf. Ser..

[688] Hadžić B., Romčević N., Romčević M., Kuryliszyn-Kudelska I., Dobrowolski W., Trajić J., Timotijević D., Narkiewicz U., Sibera D. (2012). Surface optical phonons in ZnO(Co) nanoparticles: Raman study. J. Alloys Compd..

[689] Kuryliszyn-Kudelska I., Hadžić B., Sibera D., Romčević M., Romčević N., Narkiewicz U., Łojkowski W., Arciszewska M., Dobrowolski W. (2013). Magnetic properties of ZnO(Co) nanocrystals. J. Alloys Compd..

[690] Kuryliszyn-Kudelska I., Dobrowolski W., Arciszewska M., Romčević N., Romčević M., Hadžić B., Sibera D., Narkiewicz U., Lojkowski W. (2013). Transition metals in ZnO nanocrystals: Magnetic and structural properties. Sci. Sinter..

[691] Typek J., Guskos N., Zolnierkiewicz G., Sibera D., Narkiewicz U. (2017). Magnetic resonance study of Co-doped ZnO nanomaterials: A case of high doping. Rev. Adv. Mater. Sci..

[692] Li D., Huang J.F., Cao L.Y., Ou Yang H.B., Li J.Y., Yao C.Y. (2014). Microwave hydrothermal synthesis of K^+^ doped ZnO nanoparticles with enhanced photocatalytic properties under visible-light. Mater. Lett..

[693] Eliasa M., Amin M.K., Firoz S.H., Hossain M.A., Akter S., Hossain M.A., Uddin M.N., Siddiquey I.A. (2017). Microwave-assisted synthesis of Ce-doped ZnO/CNT composite with enhanced photo-catalytic activity. Ceram. Int..

[694] Rosowska J., Kaszewski J., Witkowski B., Wachnicki Ł., Godlewski M. (2016). The effect of synthesis pressure on properties of Eu-doped ZnO nanopowders prepared by microwave hydrothermal method. Acta Phys. Pol. A.

[695] Fang M., Tang C.M., Liu Z.W. (2018). Microwave-assisted hydrothermal synthesis of Cu-doped ZnO single crystal nanoparticles with modified photoluminescence and confirmed ferromagnetism. J. Electron. Mater..

[696] Rana A.H.S., Shahid A., Lee J.Y., Kim H.S. (2018). High-power microwave-assisted Ga doping, an effective method to tailor n-ZnO/p-Si heterostructure optoelectronic characteristics. Phys. Status Solidi A.

[697] Bernardo M.S., Villanueva P.G., Jardiel T., Calatayud D.G., Peiteado M., Caballero A.C. (2017). Ga-doped ZnO self-assembled nanostructures obtained by microwave-assisted hydrothermal synthesis: Effect on morphology and optical properties. J. Alloys Compd..

[698] Azqhandi M.H.A., Vasheghani F.B., Rajabi F.H., Keramati M. (2017). Synthesis of Cd doped ZnO/CNT nanocomposite by using microwave method: Photocatalytic behavior, adsorption and kinetic study. Results Phys..

[699] Li D., Huangn J.F., Cao L.Y., Li J.Y., Yang H.B.O., Yao C.Y. (2014). Microwave hydrothermal synthesis of Sr^2+^ doped ZnO crystallites with enhanced photocatalytic properties. Ceram. Int..

[700] Hu Y., Qian H., Liu Y., Du G., Zhang F., Wang L., Hu X. (2011). A microwave-assisted rapid route to synthesize ZnO/ZnS core–shell nanostructures via controllable surface sulfidation of ZnO nanorods. CrystEngComm.

[701] Ruiz Peralta M.D.L., Pal U., Sánchez Zeferino R. (2012). Photoluminescence (PL) quenching and enhanced photocatalytic activity of Au-decorated ZnO nanorods fabricated through microwave-assisted chemical synthesis. ACS Appl. Mater. Interfaces.

[702] Bazant P., Kuritka I., Hudecek O., Machovsky M., Mrlik M., Sedlacek T. (2013). Microwave-assisted synthesis of Ag/ZnO hybrid filler, preparation, and characterization of antibacterial poly(vinyl chloride) composites made from the same. Polym. Compos..

[703] Bazant P., Klofac J., Munster L., Kuritka I. (2016). Antibacterial powders for medical application prepared by microwave hydrothermal assisted synthesis. Nanosci. Nanotechnol..

[704] Liu H., Liu H., Yang J., Zhai H., Liu X., Jia H. (2019). Microwave-assisted one-pot synthesis of Ag decorated flower-like ZnO composites photocatalysts for dye degradation and NO removal. Ceram. Int..

[705] Saoud K., Rola Alsoubaihi R., Bensalah N., Bora T., Bertino M., Dutta J. (2015). Synthesis of supported silver nano-spheres on zinc oxide nanorods for visible light photocatalytic applications. Mater. Res. Bull..

[706] Motshekga S.C., Ray S.S., Onyango M.S., Momba M.N.B. (2013). Microwave-assisted synthesis, characterization and antibacterial activity of Ag/ZnO nanoparticles supported bentonite clay. J. Hazard. Mater..

[707] Liu X., Pan L., Lv T., Lu T., Zhu G., Sun Z., Sun C. (2011). Microwave-assisted synthesis of ZnO–graphene composite for photocatalytic reduction of Cr(VI). Catal. Sci. Technol..

[708] Lu T., Pan L., Li H., Zhu G., Lv T., Liu X., Sun Z., Chen T., Chua D.H.C. (2011). Microwave-assisted synthesis of graphene–ZnO nanocomposite for electrochemical supercapacitors. J. Alloys Compd..

[709] Lv T., Pan L., Liu X., Lu T., Zhu G., Sun Z. (2011). Enhanced photocatalytic degradation of methylene blue by ZnO-reduced graphene oxide composite synthesized via microwave-assisted reaction. J. Alloys Compd..

[710] Omar F.S., Ming H.N., Hafiz S.M., Ngee L.H. (2014). Microwave synthesis of zinc oxide/reduced graphene oxide hybrid for adsorption-photocatalysis application. Int. J. Photoenergy.

[711] Lellala K., Namratha K., Byrappa K. (2016). Microwave assisted synthesis and characterization of nanostructure zinc oxide-graphene oxide and photo degradation of brilliant blue. Mater. Today Proc..

[712] Sreejesh M., Dhanush S., Rossignol F., Nagaraja H.S. (2017). Microwave assisted synthesis of rGO/ZnO composites for non-enzymatic glucose sensing and supercapacitor applications. Ceram. Int..

[713] Romeiro F.C., Rodrigues M.A., Silva L.A.J., Catto A.C., Luis F., Silva L.F., Longo E., Nossol E., Lima R.C. (2017). rGO-ZnO nanocomposites for high electrocatalytic effect on water oxidation obtained by microwave-hydrothermal method. Appl. Surf. Sci..

[714] Liu X., Pan L., Lv T., Sun Z. (2013). Investigation of photocatalytic activities over ZnO–TiO_2_–reduced graphene oxide composites synthesized via microwave-assisted reaction. J. Colloid Interface Sci..

[715] Zhu G., Li X., Wang H., Zhang L. (2017). Microwave assisted synthesis of reduced graphene oxide incorporated MOF-derived ZnO composites for photocatalytic application. Catal. Commun..

[716] Długosz O., Szostak K., Banach M. (2020). Photocatalytic properties of zirconium oxide–zinc oxide nanoparticles synthesised using microwave irradiation. Appl. Nanosci..

[717] Obaidullah M., Takeshi Furusawa T., Siddiquey I.A., Bahadur N.M., Sato M., Suzuki N. (2018). A fast and facile microwave irradiation method for the synthesis of ZnO@ZrO_2_ core-shell nanocomposites and the investigation of their optical properties. Adv. Powder Technol..

[718] Yusof N.A.A., Zain N.M., Pauzi N. (2019). Synthesis of chitosan/zinc oxide nanoparticles stabilized by chitosan via microwave heating. Bull. Chem. React. Eng. Catal..

[719] Hoseinzadeha A., Habibi-Yangjeha A., Davari M. (2016). Antifungal activity of magnetically separable Fe_3_O_4_/ZnO/AgBr nanocomposites prepared by a facile microwave-assisted method. Prog. Nat. Sci. Mater. Int..

[720] Krithika S., Balavijayalakshmi J. (2019). Synthesis of molybdenum disulfide doped zinc oxide nanocomposites by microwave assisted method. Mater. Res. Express.

[721] Ahmada N., Umar A., Kumar R., Alam M. (2016). Microwave-assisted synthesis of ZnO doped CeO_2_ nanoparticles as potential scaffold for highly sensitive nitroaniline chemical sensor. Ceram. Int..

[722] Babitha K.B., Linsha V., Anas S., Mohamed A.P., Kiran M., Ananthakumar S. (2015). Microwave assisted aqueous synthesis of organosilane treated mesoporous Si@ZnO nano architectures as dual-functional, photocatalysts. J. Environ. Chem. Eng..

[723] Cho S., Kim S., Oh E., Seung-Ho Jung S.-H., Lee K.-H. (2009). Synthesis of hierarchical hexagonal zinc oxide/zinc aluminium hydroxide heterostructures through epitaxial growth using microwave irradiation. CrystEngComm.

[724] Arin J., Thongtem S., Thongtem T. (2013). Single-step synthesis of ZnO/TiO_2_ nanocomposites by microwave radiation and their photocatalytic activitie. Mater. Lett..

[725] Ashok C.H., Venkateswara Rao K. (2014). ZnO/TiO_2_ nanocomposite rods synthesized by microwave-assisted method for humidity sensor application. Superlattice Microst..

[726] Vijayalakshmi K., Karthick K. (2014). High quality ZnO/CuO nanocomposites synthesized by microwave assisted reaction. J. Mater. Sci. Mater. Electron..

[727] Vijayalakshmi K., Sivaraj D. (2015). Enhanced antibacterial activity of Cr doped ZnO nanorods synthesized using microwave processing. RSC Adv..

[728] Bhattia S., Surveb S., Shukla V.N. (2017). Structure and Magnetic Characteristics of Co & Cr-doped ZnO nanoparticles, synthesized by using Microwave method. Mater. Today Proc..

[729] Badhusha M.S.M. (2016). Microwave assisted synthesis of ZnO and Co doped ZnO nanoparticles and their antibacterial activity. Der Pharma. Chem..

[730] Karthik K., Dhanuskodi S., Gobinath C., Sivaramakrishnan S. (2015). Microwave-assisted synthesis of CdO-ZnO nanocomposite and its antibacterial activity against human pathogens. Spectrochim. Acta A.

[731] Wolska-Kornio E., Kaszewski J., Witkowski B.S., Wachnicki Ł., Godlewski M. (2016). The effect of annealing on properties of europium doped ZnO nanopowders obtained by a microwave hydrothermal method. Opt. Mater..

[732] Zhao J., Ge S., Pan D., Pan Y., Murugadoss V., Li R., Xie W., Lu Y., Wu T., Wujcik E.K. (2019). Microwave hydrothermal synthesis of In_2_O_3_-ZnO nanocomposites and their enhanced photoelectrochemical properties. J. Electrochem. Soc..

[733] Prakash T., Jayaprakash R., Espro C., Neri G., Ranjith E. (2014). Kumar Effect of Sn doping on microstructural and optical properties of ZnO nanoparticles synthesized by microwave irradiation method. J. Mater. Sci..

[734] Kashinath L., Namratha K., Byrappa K. (2015). Microwave assisted facile hydrothermal synthesis and characterization of zinc oxide flower grown on graphene oxide sheets for enhanced photodegradation of dyes. Appl. Surf. Sci..

[735] Karunakaran C., Rajeswari V., Gomathisankar P. (2011). Optical, electrical, photocatalytic, and bactericidal properties of microwave synthesized nanocrystalline Ag-ZnO and ZnO. Solid State Sci..

[736] Cao W., Chen L., Qi Z. (2015). Microwave-assisted synthesis of Ag/Ag_2_SO_4_/ZnO nanostructures for efficient visible-light-driven photocatalysis. J. Mol. Catal. A-Chem..

[737] Duan L., Wang P., Wei F., Yu X., Fan J., Xia H., Zhu P., Tian Y. (2014). Microwave-assisted fabrication and characterization of p-ZnO:(Ag,N) nanorods/n-Si photodetector. J. Alloys Compd..

[738] Shingange K., Tshabalala Z.P., Ntwaeaborwa O.M., Motaung D.E., Mhlongo G.H. (2016). Highly selective NH_3_ gas sensor based on Au loaded ZnO nanostructuresprepared using microwave-assisted method. J. Colloid Interface Sci..

[739] Zhang H., Chen W.-G., Li Y.-Q., Song Z.-H. (2018). Gas sensing performances of ZnO hierarchical structures for detecting dissolved gases in transformer oil: A mini review. Front. Chem..

[740] Bakshi M.S. (2016). How surfactants control crystal growth of nanomaterials. Cryst. Growth Des..

[741] Morsy S.M.I. (2014). Role of surfactants in nanotechnology and their applications. Int. J. Curr. Microbiol. Appl. Sci.

[742] Quintanilla-Carvajal M.X., Matiacevich S., Hernández-Sánchez H., Gutiérrez-López G.F. (2015). Role of surfactants and their applications in structured nanosized systems. Food Nanoscience and Nanotechnology.

[743] Heinz H., Pramanik C., Heinz O., Ding Y., Mishra R.K., Marchon D., Flatt R.J., Estrela-Lopis I., Llop J., Moya S. (2017). Nanoparticle decoration with surfactants: Molecular interactions, assembly, and applications. Surf. Sci. Rep..

[744] Chen Y., Renner P., Liang H. (2019). Dispersion of Nanoparticles in Lubricating Oil: A Critical Review. Lubricants.

[745] Watt J., Cheong S., Tilley R.D. (2013). How to control the shape of metal nanostructures in organic solution phase synthesis for plasmonics and catalysis. Nano Today.

[746] Wang J., Chen R., Xiang L., Komarneni S. (2018). Synthesis, properties and applications of ZnO nanomaterials with oxygen vacancies: A review. Ceram. Int..

[747] Demazeau G. (2007). Solvothermal reactions: An opening-up on the synthesis of novel materials or the development of new processes. High. Press. Res..

[748] Demazeau G. (2010). Solvothermal processes: Definition, key factors governing the involved chemical reactions and new trends. Z. Nat..

[749] Demazeau G. (2008). Solvothermal reactions: An original route for the synthesis of novel materials. J. Mater. Sci..

[750] Demazeau G. (2008). Solvothermal processes: New trends in materials chemistry. J. Phys. Conf. Ser..

[751] Inoue M., Somiya S. (2013). Solvothermal synthesis of metal oxides. Handbook of Advanced Ceramics.

[752] Lai J., Niu W., Luque R., Xu G. (2015). Solvothermal synthesis of metal nanocrystals and their applications. Nano Today.

[753] Anžlovar A., Kogej K., Orel Z.C., Žigon M. (2014). Impact of inorganic hydroxides on ZnO nanoparticle formation and morphology. Cryst. Growth Des..

[754] Garnweitner G., Niederberger M. (2008). Organic chemistry in inorganic nanomaterials synthesis. J. Mater. Chem..

[755] Šarić A., Štefanić G., Dražić G., Gotić M. (2015). Solvothermal synthesis of zinc oxide microspheres. J. Alloys Compd..

[756] Šarić A., Despotović I., Štefanić G., Dražić G. (2017). The influence of ethanolamines on the solvothermal synthesis of zinc oxide: A combined experimental and theoretical study. ChemistrySelect.

[757] Šarić A., Despotović I., Štefanić G. (2019). Solvothermal synthesis of zinc oxide nanoparticles: A combined experimental and theoretical study. J. Mol. Struct..

[758] Wojnarowicz J., Opalinska A., Chudoba T., Gierlotka S., Mukhovskyi R., Pietrzykowska E., Sobczak K., Lojkowski W. (2016). Effect of water content in ethylene glycol solvent on the size of ZnO nanoparticles prepared using microwave solvothermal synthesis. J. Nanomater..

[759] Hu X., Gong J., Zhang L., Yu J.C. (2008). Continuous size tuning of monodisperse ZnO colloidal nanocrystal clusters by a microwave-polyol process and their application for humidity sensing. Adv. Mater..

[760] Jamatia T., Skoda D., Urbanek P., Munster L., Sevcik J., Kuritka I. (2019). Microwave-assisted particle size-controlled synthesis of ZnO nanoparticles and its application in fabrication of PLED device. J. Phys. Conf. Ser..

[761] Zhu P., Zhang J., Wu Z., Zhang Z. (2008). Microwave-assisted synthesis of various ZnO hierarchical nanostructures: Effects of heating parameters of microwave oven. Cryst. Growth Des..

[762] Bilecka I., Elser P., Niederberger M. (2009). Kinetic and thermodynamic aspects in the microwave-assisted synthesis of ZnO nanoparticles in benzyl alcohol. ACS Nano.

[763] Bilecka I., Djerdj I., Niederberger M. (2008). One-minute synthesis of crystalline binary and ternary metal oxide nanoparticles. Chem. Commun..

[764] Canh T.D., Tuyen N.V., Long N.N. (2009). Influence of solvents on the growth of zinc oxide nanoparticles fabricated by microwave irradiation. VNUJ. Sci. Math. Phys..

[765] Kajbafvala A., Shayegh M.R., Mazloumi M., Zanganeh S., Lak A., Mohajerani M.S., Sadrnezhaad S.K. (2009). Nanostructure sword-like ZnO wires: Rapid synthesis and characterization through a microwave-assisted route. J. Alloys Compd..

[766] Garces H.F., Espinal A.E., Suib S.L. (2012). Tunable shape microwave synthesis of zinc oxide nanospheres and their desulfurization performance compared with nanorods and platelet-like morphologies for the removal of hydrogen sulfide. J. Phys. Chem. C.

[767] Zhang L., Zhu Y.J. (2009). ZnO micro- and nano-structures: Microwave-assisted solvothermal synthesis, morphology control and photocatalytic properties. Appl. Phys. A.

[768] Liu J.-S., Cao J.-M., Li Z.-Q., Ji G.-B., Zheng M.-B. (2007). A simple microwave-assisted decomposing route for synthesis of ZnO nanorods in the presence of PEG400. Mater. Lett..

[769] Bhatte K.D., Tambade P., Fujita S.I., Arai M., Bhanage B.M. (2010). Microwave-assisted additive free synthesis of nanocrystalline zinc oxide. Powder Technol..

[770] Brahma S., Jagannatha R.K., Shivashankar S. (2010). Rapid growth of nanotubes and nanorods of würtzite ZnO through microwave-irradiation of a metalorganic complex of zinc and a surfactant in solution. Bull. Mater. Sci..

[771] Brahma S., Shivashankar S.A. (2020). Microwave irradiation assisted rapid growth of ZnO nanorods over metal coated/electrically conducting substrate. Mater. Lett..

[772] Schneider J.J., Hoffmann R.C., Engstler J., Klyszcz A., Erdem E., Jakes P., Eichel R.A., Pitta-Bauermann L., Bill J. (2010). Synthesis, characterization, defect chemistry, and FET properties of microwave-derived nanoscaled zinc oxide. Chem. Mater..

[773] Ambrozic G., Orel Z.C., Zigon M. (2011). Mcirowave-assisted non-aqueous synthesis of ZnO Nanoparticles. Mater. Technol..

[774] Kuo K.T., Lin J.C. (2011). Synthesis of zinc oxide nanoparticles in microwave reactor for TFT fabrication. SID.

[775] Rai P., Kim S.G., Yu Y.T. (2012). Microwave assisted synthesis of flower-like ZnO and effect of annealing atmosphere on its photoluminescence property. J. Mater. Sci. Mater. Electron..

[776] Bhatte K.D., Sawant N.D., Watile R.A., Bhanage B.M. (2012). A rapid, one step microwave assisted synthesis of nanosize zinc oxide. Mater. Lett..

[777] Kajbafvala A., Samberg J.P., Ghorbani H., Kajbafvala E., Sadrnezhaad S.K. (2012). Effects of initial precursor and microwave irradiation on step-by-step synthesis of zinc oxide nano-architectures. Mater. Lett..

[778] Khoza P.B., Moloto M.J., Sikhwivhilu L.M. (2012). The effect of solvents, acetone, water, and ethanol, on the morphological and optical properties of ZnO nanoparticles prepared by microwave. J. Nanotechnol..

[779] Shen X., Sun J., Zhu G., Ji Z., Chen Z., Li N. (2013). Morphological syntheses of ZnO nanostructures under microwave irradiation. J. Mater. Sci..

[780] Zhao X., Qi L. (2012). Rapid microwave-assisted synthesis of hierarchical ZnO hollow spheres and their application in Cr(VI) removal. Nanotechnology.

[781] Mahmood M.A., Baruah S., Dutta J. (2011). Enhanced visible light photocatalysis by manganese doping or rapid crystallization with ZnO nanoparticles. Mater. Chem. Phys..

[782] Baruah S., Rafique R.F., Dutta J. (2008). Visible light photocatalysis by tailoring crystal defects in zinc oxide nanostructures. Nano Brief. Rep. Rev..

[783] Bu I.Y.Y. (2013). Rapid synthesis of ZnO nanostructures through microwave heating process. Ceram. Int..

[784] Garza M.D.L., Lópeza I., Aviñaa F., Gomez I. (2013). Microwave-assisted solvothermal synthesis of porous zinc oxide nanostructures. J. Ovonic. Res..

[785] Ma J., Liu J., Bao Y., Zhu Z., Liu H. (2013). Morphology-photocatalytic properties-growth mechanism for ZnO nanostructures via microwave-assisted hydrothermal synthesis. Cryst. Res. Technol..

[786] Ma J., Liu J., Bao Y., Zhu Z., Wang X., Zhang J. (2013). Synthesis of largescale uniform mulberry-like ZnO particles with microwave hydrothermal method and its antibacterial property. Ceram. Int..

[787] Krishnapriya R., Praneetha S., Murugan A.V. (2015). Energy-efficient, microwave-assisted hydro/solvothermal synthesis of hierarchical flowers and rice grain-like ZnO nanocrystals as photoanodes for high performance dye-sensitized solar cells. CrystEngComm.

[788] Pimentel A., Rodrigues J., Duarte P., Nunes D., Costa F.M., Monteiro T., Martins R., Fortunato E. (2015). Effect of solvents on ZnO nanostructures synthesized by solvothermal method assisted by microwave radiation: A photocatalytic study. J. Mater. Sci..

[789] Hoffmann R.C., Sanctis S., Erdem E., Weber S., Schneider J.J. (2016). Zinc diketonates as single source precursors for ZnO nanoparticles: Microwave-assisted synthesis, electrophoretic deposition and field-effect transistor device properties. J. Mater. Chem. C.

[790] Sekhar M.C., Ramana M.V. (2017). Instant synthesis of ZnO nanoparticles by microwave hydrothermal method. Int. J. Nanosci. Nanotechnol..

[791] Karthikeyan L., Akshaya M.V., Basu P.K. (2017). Microwave assisted synthesis of ZnO and Pd-ZnO nanospheres for UV photodetector. Sens. Actuators A-Phys..

[792] Iwamura T., Goto S., Sakaguchi M., Chujo Y. (2016). Synthesis of submicrometer zinc oxide particles and zinc oxide nanowires using microwave irradiation. Chem. Lett..

[793] Skoda D., Urbanek P., Sevcik J., Munster L., Antos J., Kuritka I. (2018). Microwave-assisted synthesis of colloidal ZnO nanocrystals and their utilization in improving polymer light emitting diodes efficiency. Mater. Sci. Eng. B.

[794] Bhatta L.K.G., Bhatta U.M., Venkatesh K. (2019). Facile microwave-assisted synthesis of zinc oxide and characterization. J. Sci. Ind. Res..

[795] Saloga P.E.J., Thünemann A.F. (2019). Microwave-assisted synthesis of ultrasmall zinc oxide nanoparticles. Langmuir.

[796] Sedlák J., Kuřitka I., Machovský M., Šuly P., Bažant P., Sedláček T. (2015). Zinc oxide nanoparticles with surface modified by degradation of capping polymers in situ during microwave synthesis. Adv. Powder Technol..

[797] De Peres M.L., de Avila Delucis R., Amico S.C., Gatto D.A. (2019). Zinc oxide nanoparticles from microwave-assisted solvothermal process: Photocatalytic performance and use for wood protection against xylophagous fungus. Nanomater. Nanotechnol..

[798] Wojnarowicz J., Kusnieruk S., Chudoba T., Gierlotka S., Lojkowski W., Knoff W., Lukasiewicz M.I., Witkowski B.S., Wolska A., Klepka M.T. (2015). Paramagnetism of cobalt-doped ZnO nanoparticles obtained by microwave solvothermal synthesis. Beilstein J. Nanotechnol..

[799] Wojnarowicz J., Kuśnieruk S., Chudoba T., Mizeracki J., Łojkowski W. (2015). Microwave solvothermal synthesis of Co-doped ZnO nanoparticles. Glass Ceram..

[800] Wojnarowicz J., Chudoba T., Gierlotka S., Sobczak K., Lojkowski W. (2018). Size control of cobalt-doped ZnO nanoparticles obtained in microwave solvothermal synthesis. Crystals.

[801] Skoda D., Urbanek P., Sevcik J., Munster L., Nadazdy V., Cullen D.A., Bazant P., Antos J., Kuritka I. (2018). Colloidal cobalt-doped ZnO nanoparticles by microwave-assisted synthesis and their utilization in thin composite layers with MEH-PPV as an electroluminescent material for polymer light emitting diodes. Org. Electron..

[802] Wojnarowicz J., Mukhovskyi R., Pietrzykowska E., Kusnieruk S., Mizeracki J., Lojkowski W. (2016). Microwave solvothermal synthesis and characterization of manganese-doped ZnO nanoparticles. Beilstein J. Nanotechnol..

[803] Wojnarowicz J., Omelchenko M., Szczytko J., Chudoba T., Gierlotka S., Majhofer A., Twardowski A., Lojkowski W. (2018). Structural and magnetic properties of Co-Mn codoped ZnO nanoparticles obtained by microwave solvothermal synthesis. Crystals.

[804] Tomaszewska-Grzeda A., Opalinska A., Grzanka E., Lojkowski W., Gedanken A., Godlewski M., Yatsunenko S., Osinin V., Story T. (2006). Magnetic properties of ZnMnO nanopowders solvothermally grown at low temperature from zinc and manganese acetate. Appl. Phys. Lett..

[805] Bhattacharyya S., Gedanken A. (2008). Microwave-assisted insertion of silver nanoparticles into 3-D mesoporous zinc oxide nanocomposites and nanorods. J. Phys. Chem. C.

[806] Wang L., Hu Q., Li Z., Guo J., Li Y. (2012). Microwave-assisted synthesis and photocatalytic performance of Ag-doped hierarchical ZnO architectures. Mater. Lett..

[807] Herring N.P., AbouZeid K., Mohamed M.B., Pinsk J., El-Shall M.S. (2011). Formation mechanisms of gold zinc oxide hexagonal nanopyramids by heterogeneous nucleation using microwave synthesis. Langmuir.

[808] Fidelus J., Piticescu R.R., Piticescu R.M., Lojkowski W., Giurgiu L. (2008). Solvothermal synthesis of Co-doped ZnO nanopowders. Z. Naturforsch..

[809] Lojkowski W., Gedanken A., Grzanka E., Opalinska A., Strachowski T., Pielaszek R., Tomaszewska-Grzeda A., Yatsunenko S., Godlewski M., Matysiak H. (2009). Solvothermal synthesis of nanocrystalline zinc oxide doped with Mn^2+^, Ni^2+^, Co^2+^ and Cr^3+^ ions. J. Nanopart. Res..

[810] Bilecka I., Luo L., Djerdj I., Rossel M.D., Jagodic M., Jaglicic Z., Masubuchi Y., Kikkawa S., Niederberger M. (2011). Microwave-assisted nonaqueous Sol-Gel chemistry for highly concentrated ZnO-based magnetic semiconductor nanocrystals. J. Phys. Chem. C.

[811] Hammarberg E., Prodi-Schwab A., Feldmann C. (2009). Microwave-assisted polyol synthesis of aluminium- and indium-doped ZnO nanocrystals. J. Colloid Interface Sci..

[812] Tuyen N.V., Canh T.D., Long N.N., Nghia N.X., Trinh B.N.Q., Shen Z.J. (2009). Synthesis of undoped and M-doped ZnO (M = Co, Mn) nanopowder in water using microwave irradiation. Phys. Conf. Ser..

[813] Tuyen N.V., Long N.N., Canh T.D. (2011). Synthesis and characteristics of single-crystal Ni-doped ZnO nanorods prepared by a microwave irradiation method. Surf. Sci. Nanotechnol..

[814] Liu Q., Zhu P., Li G., Guo Q., Shuai X., Sun R., Wong C. A microwave-assisted solvothermal process to synthesize Al-doped ZnO powders and its optical and electrical properties. Proceedings of the 17th International Conference on Electronic Packaging Technology (ICEPT).

[815] Jayathilake D.S.Y., Nirmal Peiris T.A., Sagu J.S., Potter D.B., Wijayantha K.G.U., Carmalt C.J., Southee D.J. (2017). Microwave-assisted synthesis and processing of Al-doped, Ga-doped, and Al, Ga codoped ZnO for the pursuit of optimal conductivity for transparent conducting film fabrication. ACS Sustain. Chem. Eng..

[816] Obaidullah M., Furusawa T., Siddiquey I.S., Sato M., Suzuki N. (2017). Synthesis of ZnO-Al_2_O_3_ core-shell nanocomposite materials by fast and facile microwave irradiation method and investigation of their optical properties. Adv. Powder Technol..

[817] Manoharan S.S., Arora S. (2009). Photoluminescent properties of Mg doped ZnO by microwave combustion and microwave polyol method. Mater. Sci. Eng. B.

[818] Liu K., Qin Y., Muhammad Y., Zhu Y., Tang R., Chen N., Shi H., Zhang H., Tong Z., Yu B. (2019). Effect of Fe_3_O_4_ content and microwave reaction time on the properties of Fe_3_O_4_/ZnO magnetic nanoparticles. J. Alloys Compd..

[819] Zhou M., Hu Y., Liu Y., Yang W., Qian H. (2012). Microwave-assisted route to fabricate coaxial ZnO/C/CdS nanocables with enhanced visible light-driven photocatalytic activity. CrystEngComm.

[820] Liu Y., Hu Y., Zhou M., Qian H., Hu X. (2012). Microwave-assisted non-aqueous route to deposit well-dispersed ZnO nanocrystals on reduced graphene oxide sheets with improved photoactivity for the decolorization of dyes under visible light. Appl. Catal. B.

[821] Hsueh Y.-H., Hsieh C.-T., Chiu S.-T., Tsai P.-H., Liu C.-Y., Ke W.-J. (2019). Antibacterial property of composites of reduced graphene oxide with nano-silver and zinc oxide nanoparticles synthesized using a microwave-assisted approach. Int. J. Mol. Sci..

[822] Guo Y., Wang H., He C., Qiu L., Cao X. (2009). Uniform carbon-coated ZnO nanorods: Microwave-assisted preparation, cytotoxicity, and photocatalytic activity. Langmuir.

[823] Moghaddam F.M., Saeidian H. (2007). Controlled microwave-assisted synthesis of ZnO nanopowder and its catalytic activity for O-acylation of alcohol and phenol. Mater. Sci. Eng. B.

[824] Rabieh S., Bagheri M., Heydari M., Badiei E. (2014). Microwave assisted synthesis of ZnO nanoparticles in ionic liquid [Bmim]cl and their photocatalytic investigation. Mater. Sci. Semicon. Proc..

[825] Brahma S., Shivashankar S.A. (2010). Microwave irradiation-assisted method for the deposition of adherent oxide films on semiconducting and dielectric substrates. Thin Solid Films.

[826] Brahma S., Shivashankar S.A. (2013). Surfactant free, non-aqueous method, for the deposition of ZnO nanoparticle thin films on Si(100) substrate with tunable ultraviolet (UV) emission. Curr. Nanosci..

[827] Brahma S., Huang J.-L., Liu C.-P., Kukreja L.M., Shivashankar S.A. (2013). Low temperature and rapid deposition of ZnO nanorods on Si(100) substrate with tunable optical emissions. Mater. Chem. Phys..

[828] Brahma S., Shivashankar S.A. (2014). Preparation of zinc oxide coatings by using newly designed metal–organic complexes of Zn: Effect of molecular structure of the precursor and surfactant over the crystallization, growth and luminescence. J. Alloys Compd..

[829] Brahma S., Lo K.-Y., Shivashankar S.A. (2015). Facile, low temperature (≤100 °C) growth of vertically aligned ZnO nanorods over an amorphous or disordered surface. Mater. Lett..

[830] Brahma S., Jaiswal P., Suresh K.S., Lo K.-Y., Suwas S., Shivashankar S.A. (2015). Effect of substrates and surfactants over the evolution of crystallographic texture of nanostructured ZnO thin films deposited through microwave irradiation. Thin Solid Films.

[831] Kumar V., Gohain M., Som S., Kumar V., Bezuindenhoudt B.C.B., Swart H.C. (2016). Microwave assisted synthesis of ZnO nanoparticles for lighting and dye removal application. Phys. B Condens. Matter..

[832] Sun L., Shao Q., Zhang Y., Jiang H., Ge S., Lou S., Lin J., Zhang J., Wu S., Dong M. (2020). N self-doped ZnO derived from microwave hydrothermal synthesized zeolitic imidazolate framework-8 toward enhanced photocatalytic degradation of methylene blue. J. Colloid Interface Sci..

[833] Varadhaseshan R., Meenakshi Sundar S., Prema C. (2014). On the preparation, structural and magnetic properties of ZnO: Co nanoparticles. Eur. Phys. J. Appl. Phys..

[834] Varadhaseshan R., Meenakshi Sundar S., Prema C. (2014). Effect of pH and particle size on the magnetic behavior of cobalt doped ZnO nanocrystals. Phys. Procedia.

[835] Guruvammal D., Selvaraj S., Meenakshi Sundar S. (2018). Structural, optical and magnetic properties of Co doped ZnO DMS nanoparticles by microwave irradiation method. J. Magn. Magn. Mater..

[836] Neupanea G.R., Kaphle A., Hari P. (2019). Microwave-assisted Fe-doped ZnO nanoparticles for enhancement of silicon solar cell efficiency. Sol. Energy Mater. Sol. Cells.

[837] Varadhaseshan R., Ravi S., Meenakshi Sundar S. (2018). Synthesis, structural and magnetic characterization of Mn^2+^ doped ZnO nanopowders. Int. J. Creat. Res. Thoughts.

[838] Xin Z., Li L., Zhang X., Zhang W. (2018). Microwave-assisted hydrothermal synthesis of chrysanthemum-like Ag/ZnO prismatic nanorods and their photocatalytic properties with multiple modes for dye degradation and hydrogen production. RSC Adv..

[839] Wojnarowicz J., Chudoba T., Majcher A., Łojkowski W., Cravotto G., Carnaroglio D. (2017). Microwaves applied to hydrothermal synthesis of nanoparticles. Microwave Chemistry.

[840] Yiamsawas D., Boonpavanitchakul K., Kangwansupamonkon W. (2009). Preparation of ZnO nanostructures by solvothermal method. J. Microsc Soc. Thail..

[841] Yuan F., Hu P., Yu L., Li S., Ke J. (2008). Deposition of ZnO on the surface of Al metal particles by esterification reaction under solvothermal conditions. J. Mater. Sci..

[842] Demir M.M., Muñoz-Espi R., Lieberwirtha I., Wegner G. (2006). Precipitation of monodisperse ZnO nanocrystalsvia acid-catalyzed esterification of zinc acetate. J. Mater. Chem..

[843] Gotic M., Music S. (2008). Synthesis of nanocrystalline iron oxide particles in the Iron(III) acetate/alcohol/acetic acid system. Eur. J. Inorg. Chem..

[844] Li Q., Li H., Wang R., Li G., Yang H., Chen R. (2013). Controllable microwave and ultrasonic wave combined synthesis of ZnO micro-/nanostructures in HEPES solution and their shape-dependent photocatalytic activities. J. Alloys Compd..

[845] Li D., Wang J., Wu X., Feng C., Li X. (2013). Ultraviolet-assisted synthesis of hourglass-like ZnO microstructure through an ultrasonic and microwave combined technology. Ultrason. Sonochem..

[846] Lagashetty A., Havanoor V., Basavaraja S., Balaji S.D., Venkataraman A. (2007). Microwave-assisted route for synthesis of nanosized metal oxides. Sci. Technol. Adv. Mater..

[847] Cao Y., Liu B., Huang R., Xia Z., Ge S. (2011). Flash synthesis of flower-like ZnO nanostructures by microwave-induced combustion process. Mater. Lett..

[848] Nehru L.C., Swaminathan V., Sanjeeviraja C. (2012). Rapid synthesis of nanocrystalline ZnO by a microwave-assisted combustion method. Powder Technol..

[849] Köseoğlu Y. (2014). A simple microwave-assisted combustion synthesis and structural, optical and magnetic characterization of ZnO nanoplatelets. Ceram. Int..

[850] Chandore V., Carpenter G., Sen R., Gupta N. (2013). Synthesis of nanocrystalline ZnO by Microwave Assisted Combustion method: An eco friendly and solvent free route. Int. J. Environ. Sci. Dev. Monit..

[851] Kooti M., Naghdi Sedeh A. (2013). Microwave-assisted combustion synthesis of ZnO nanoparticles. J. Chem..

[852] Assi N., Mohammadi A., Manuchehri Q.S., Walker R.B. (2014). Synthesis and characterization of ZnO nanoparticle synthesized by a microwave-assisted combustion method and catalytic activity for the removal of ortho-nitrophenol. Desalin. Water Treat..

[853] Manikandan A., Vijaya J.J., Ragupathi C., Kennedy L.J. (2014). Optical properties and dye-sensitized solar cell applications of ZnO nanostructures prepared by microwave combustion synthesis. J. Nanosci. Nanotechnol..

[854] Safeera T.A., Anila E.I. (2017). Synthesis and characterization of ZnO nanophosphor by microwave combustion technique. Int. J. Recent Innov. Eng. Res..

[855] Mallikarjunaswamy C., Lakshmi Ranganatha V., Ramu R., Nagaraju U.G. (2020). Facile microwave-assisted green synthesis of ZnO nanoparticles: Application to photodegradation, antibacterial and antioxidant. J. Mater. Sci. Mater. Electron..

[856] Jun T., Song K., Jung Y., Jeong S., Moon J. (2011). High-performance low-temperature solution-processable ZnO thin film transistors by microwave-assisted annealing. J. Mater. Chem..

[857] Huang H., Lv L., Hu Y., Lou Z., Hou Y., Teng F. (2017). Enhanced performance in inverted polymer solar cells employing microwave-annealed sol-gel ZnO as electron transport layers. Org. Electron..

[858] Peiris T.A.N., Sagu J.S., Yusof Y.H., Wijayantha K.G.U. (2015). Microwave-assisted low temperature fabrication of ZnO thin film electrodes for solar energy harvesting. Thin Solid Films.

[859] Azizi S., Mohamad R., Mahdavi Shahri M. (2017). Green microwave-assisted combustion synthesis of zinc oxide nanoparticles with citrullus colocynthis (L.) schrad: Characterization and biomedical applications. Molecules.

[860] Anas S., Rahul S., Babitha K.B., Mangalaraja R.V., Ananthakumar S. (2015). Microwave accelerated synthesis of zinc oxide nanoplates and theirenhanced photocatalytic activity under UV and solar illuminations. Appl. Surf. Sci..

[861] Chua X., Chena T., Zhanga W., Zhenga B., Shui H. (2009). Investigation on formaldehyde gas sensor with ZnO thick film prepared through microwave heating method. Sens. Actuators B-Chem..

[862] Alnarabiji M.S., Yahya N., Hamid S.B.A., Azizli K.A., Shafie A., Solemani H. (2014). Microwave synthesis of ZnO nanoparticles for enhanced oil recovery. Adv. Mater. Res..

[863] Assi N., Azar P.A., Tehrani M.S., Husain S.W., Darwish M., Pourmand S. (2017). Synthesis of ZnO-nanoparticles by microwave assisted sol-gel method and its role in photocatalytic degradation of food dye Tartrazine (Acid Yellow 23). Int. J. Nano Dimens..

[864] Xu W.G., Ma C.X., Lu S.X., Dai L., Zhang H.F. (2011). Microwave-assisted synthesis and photocatalytic activity of quantum-sized ZnO. Adv. Mater. Res..

[865] Bai Z., Xie C., Zhang S., Xu W., Xu J. (2011). Microwave sintering of ZnO nanopowders and characterization for gas sensing. Mater. Sci. Eng. B.

[866] Chaiyo P., Ngiwlai A., Thumthan O., Nutariya J., Sumran S., Pukird S. (2018). Electrical and gas sensing properties of ZnO nanoclusters by microwaves and carbon assisted. Mater. Today Proc..

[867] Solis-Pomar F., Jaramillo A., Lopez-Villareal J., Medina C., Rojas D., Mera A.C., Meléndrez M.F., Pérez-Tijerina E. (2016). Rapid synthesis and photocatalytic activity of ZnO nanowires obtained through microwave-assisted thermal decomposition. Ceram. Int..

[868] Zhou J., Wang Z., Wang L., Wu M., Ouyang S., Gu E. (2006). Synthesis of ZnO hexagonal tubes by a microwave heating method. Superlattice Microst..

[869] Al-Naser Q.A.H., Zhou J., Wang H., Liu G., Wang L. (2015). Synthesis, growth and characterization of ZnO microtubes using a traveling-wave mode microwave system. Mater. Res. Bull..

[870] Al-Naser Q.A.H., Zhou J., Liu G., Wang L. (2016). ZnO single crystal microtubes: Synthesis, growth mechanism, and geometric structure using direct microwave irradiation. Ceram. Int..

[871] Takahashi N. (2008). Simple and rapid synthesis of ZnO nano-fiber by means of a domestic microwave oven. Mater. Lett..

[872] Sakamato N., Ishizuka S., Wakiya N., Suzuki H. (2009). Shape controlled ZnO nanoparticle prepared by microwave irradiation method. J. Ceram. Soc. Jpn..

[873] Ansari A.R., Hussain S., Imran M., Al-Ghamdi A.A., Chandan M.R. (2018). Optical investigations of microwave induced synthesis of zinc oxide thin-film. Mater. Sci.-Pol..

[874] Qurashi A., Tabet N., Faiz M., Yamzaki T. (2009). Ultra-fast microwave synthesis of ZnO nanowires and their dynamic response toward hydrogen gas. Nanoscale Res. Lett..

[875] Cheng J., Guo R., Wang Q.-M. (2004). Zinc oxide single-crystal microtubes. Appl. Phys. Lett..

[876] Cheng H., Cheng J., Zhang Y., Wang Q.-M. (2007). Large-scale fabrication of ZnO micro-and nano-structures by microwave thermal evaporation deposition. J. Cryst. Growth.

[877] Wei G., Qin W., Ning L., Kim R., Wang G., Zhang D., Zhu P., Zheng K., Wang L. (2010). Synthesis of ZnO nanosheets by microwave thermal vapor method. J. Nanosci. Nanotechnol..

[878] Meléndrez M.F., Solis-Pomar F., Gutierrez-Lazos C.D., Flores P., Jaramillo A.F., Fundora A., Pérez-Tijerina E. (2016). A new synthesis route of ZnO nanonails via microwave plasma-assisted chemical vapor deposition. Ceram. Int..

[879] Raj R.T., Pajeevkumar K. (2012). Synthesis of ZnO nanostructures using domestic microwave oven based remote plasma deposition system. Nanosci. Nanotechnol..

[880] Subannajui K. (2016). Super-fast synthesis of ZnO nanowires by microwave air-plasma. Chem. Commun..

[881] Hattoria Y., Mukasa S., Toyota H., Inoue T., Nomura S. (2011). Continuous synthesis of magnesium-hydroxide, zinc-oxide, and silver nanoparticles by microwave plasma in water. Mater. Chem. Phys..

[882] Irzh A., Genish I., Klein L., Solovyov L.A., Gedanken A. (2010). Synthesis of ZnO and Zn nanoparticles in microwave plasma and their deposition on glass slides. Langmuir.

[883] Lee B.-J., Jo S.-I., Jeong G.-H. (2019). Synthesis of ZnO nanomaterials using low-cost compressed air as microwave plasma gas at atmospheric pressure. Nanomaterials.

[884] Yonezawa T., Hyono A., Sato S., Ariyada O. (2010). Preparation of Zinc Oxide nanoparticles by using microwave-induced plasma in liquid. Chem. Lett..

[885] Chen D., Ai S., Liang Z., Wei F. (2016). Preparation and photocatalytic properties of zinc oxide nanoparticles by microwave-assisted ball milling. Ceram. Int..

[886] Cho S., Shim D.S., Jung S.H., Oh E., Lee B.R., Lee K.H. (2009). Fabrication of ZnO nanoneedle arrays by direct microwave irradiation. Mater. Lett..

[887] Li H., Liu E.T., Chan F.Y.F., Lu Z., Chen R. (2011). Fabrication of ordered flower-like ZnO nanostructures by a microwave and ultrasonic combined technique and their enhanced photocatalytic activity. Mater. Lett..

[888] Liu Q., Liu E., Li J., Qiu Y., Chen R. (2020). Rapid ultrasonic-microwave assisted synthesis of spindle-like Ag/ZnO nanostructures and their enhanced visible-light photocatalytic and antibacterial activities. Catal. Today.

[889] Dou P., Tan F., Wang W., Sarreshteh A., Qiao X., Qiu X., Chen J. (2015). One-step microwave-assisted synthesis of Ag/ZnO/graphene nanocomposites with enhanced photocatalytic activity. J. Photoch. Photobiol. A.

[890] Selvakumari D., Abraham C.S., Subhashini P., Lakshminarayan N. (2016). Microwave synthesis of aluminium doped ZnO nanostructures and characterization. Funct. Nanostruct..

[891] Chang S.C., Li T.H., Liao Y.Z., Luo Y.K., Shen Y.L., Syu W.S., Chan H.T. Influence of applied power in microwave hydrogen plasma annealing on aluminum doped zinc oxide/tin doped indium oxide bilayer films for low emissivity application. Proceedings of the 13th International Microsystems, Packaging, Assembly and Circuits Technology Conference (IMPACT).

[892] Li Y., Chen L.L., Zhao F.X. (2018). Highly selective acetone sensor based on ternary Au/Fe_2_O_3_−ZnO synthesized via co-precipitation and microwave irradiation. Trans. Nonferrous Met. Soc. China.

[893] N’Konou K., Haris M., Lare Y., Baneto M., Napo K., Torchio P. (2016). Effect of barium doping on structural and optical properties of zinc oxide nanoparticles synthesized by microwave hydrothermal method. Phys. Status Solidi B.

[894] Mary J.A., Vijaya J.J., Kennedy L.J., Bououdina M. (2016). Microwave-assisted synthesis, characterization and antibacterial properties of Ce-Cu dual doped ZnO nanostructures. Optik.

[895] Rasouli S., Valefi M., Moeen S.J., Arabi A.M. (2011). Microwave-assisted gel combustion synthesis of ZnO-Co nano-pigments. J. Ceram. Process. Res..

[896] Hamdelou S., Guergouri K. (2016). Microstructure and electrical properties of Co-Doped ZnO varistors. J. Ceram. Sci. Tech..

[897] Yathisha R.O., Nayaka Y.A., Vidyasagar C.C. (2016). Microwave combustion synthesis of hexagonal prism shaped ZnO nanoparticles and effect of Cr on structural, optical and electrical properties of ZnO nanoparticles. Mater. Chem. Phys..

[898] Yang H., Zhang Q., Chen Y., Huang Y., Yang F., Lu Z. (2018). Ultrasonic-microwave synthesis of ZnO/BiOBr functionalized cotton fabrics with antibacterial and photocatalytic properties. Carbohydr. Polym..

[899] Zhang X.H., Xie S.Y., Jiang Z.Y., Zhang X., Tian Z.Q., Xie Z.X., Huang R.B., Zheng L.S. (2003). Rational design and fabrication of ZnO nanotubes from nanowire templates in a microwave plasma system. J. Phys. Chem. B.

[900] Tinacba E.J.C., Nuñez J.A., Tumlos R.B., Ramos H.J. (2017). ZnO/Zn and ZnO filmdeposited viamicrowave atmospheric plasma jet as photo-catalyst for Rhodamine 6G dye degradation. Thin Solid Films.

[901] Sherlya E.D., Vijaya J.J., Selvam N.C.S., Kennedy L.J. (2014). Microwave assisted combustion synthesis of coupled ZnO–ZrO_2_ nanoparticles and their role in the photocatalytic degradation of 2,4-dichlorophenol. Ceram. Int..

[902] Shahmirzaee M., Afarani M.S., Arabi A.M., Nejhad A.I. (2017). In situ crystallization of ZnAl_2_O_4_/ZnO nanocomposite on alumina granule for photocatalytic purification of wastewater. Res. Chem. Intermed..

[903] Shahmirzaee M., Afarani M.S., Nejhad A.I., Arabi A.M. (2019). Microwave-assisted combustion synthesis of ZnAl_2_O_4_ and ZnO nanostructure particles for photocatalytic wastewater treatment. Particul. Sci. Technol..

[904] Vidyasagara C.C., Hosamani G., Kariyajjanavar P., Yathishad R.O., Nayaka Y.A. (2018). One-pot microwave synthesis and effect of Cu^2+^ ions on structural properties of Cu-ZnO nano crystals. Mater. Today Proc..

[905] Ajamein H., Haghighi M. (2016). Influence of ambient gas on microwave-assisted combustion synthesis of CuO–ZnO–Al_2_O_3_ nanocatalyst used in fuel cell grade hydrogen production via methanol steam reforming. Ceram. Int..

[906] Prasad V., Simiyon G.G., Mammen A.E., Jayaprakash N. (2019). Microwave assisted synthesis, characterization and photo-catalytic study of Cu/ZnO nanocomposite. Rasayan J. Chem..

[907] Korake P.V., Kadam A.N., Garadkar K.M. (2014). Photocatalytic activity of Eu^3+^-doped ZnO nanorods synthesized via microwave assisted technique. J. Rare Earth.

[908] Rasouli S., Arabi A.-M., Naeimi A., Hashemi S.-M. (2018). Microwave-assisted combustion synthesis of ZnO: Eu nanoparticles: Effect of fuel types. J. Fluoresc..

[909] Nagao D., Fukushiman J., Hayashi Y., Takizawa H. (2015). Synthesis of homologous compounds Fe_2_O_3_(ZnO)m (m = 6, 8, 34) by various selective microwave heating conditions. Ceram. Int..

[910] Hata S., Taguchi K., Oshima K., Du Y., Shiraishi Y., Toshima N. (2019). Preparation of Ga-ZnO nanoparticles using microwave and ultrasonic irradiation, and the application of poly (3,4-ethylenedioxythiophene)-poly(styrenesulfonate) hybrid thermoelectric films. ChemistrySelect.

[911] Jantrasee S., Moontragoon P., Pinitsoontorn S., Ruttanapun C. (2019). Enhancing thermoelectric properties of nanostructure Ga-doped ZnO prepared by microwave-hydrothermal synthesizing with comparing to calculation results. Mater. Res. Express.

[912] Jun J.-H., Cho W.-J. (2019). Multi- and single-step in-situ microwave annealing as low-thermal-budget techniques for solutionprocessed indium–gallium–zinc oxide thin films. Semicond. Sci. Technol..

[913] Wu C.H., Kuo S.N., Chang K.M., Chen Y.M., Zhang Y.X., Xu N., Liu W.Y., Chin A. (2020). Reliability of atmosphere pressure-plasma enhanced chemical vapor deposition deposited indium gallium zinc oxide resistive random access memory device with microwave annealing. J. Nanosci. Nanotechnol..

[914] Köseoğlu Y. (2016). Fuel aided rapid synthesis and room temperature ferromagnetism of M_0.1_Co_0.1_Zn_0.8_O (M = Mn, Ni, Fe and Cu) DMS nanoparticles. Ceram. Int..

[915] Angel Ezhilarasi A., Judith Vijaya J., John Kennedy L., Vasanth M. (2016). Green synthesis of Mg doped zinc oxide nanoparticles using aloe vera plant extract and its characterization. J. Chem. Pharm. Sci..

[916] Al-Naser Q.A.H., Zhou J., Wang H., Liu G., Wang L. (2015). Synthesis and optical properties of MgO-doped ZnO microtubes using microwave heating. Opt. Mater..

[917] Abdullahi S.S., Köseog Y., Güner S., Kazan S., Kocaman B., Ndikilar C.E. (2015). Synthesis and characterization of Mn and Co codoped ZnO nanoparticles. Superlattices Microstruct..

[918] Gunnewiek R.F.K., Kiminami R.H.G.A. (2017). Two-step microwave sintering of nanostructured ZnO-based varistors. Ceram. Int..

[919] Dastafkan K., Kiani A., Obeydavi A., Rahimi M. (2018). Crystallization and solid solution attainment of samarium doped ZnO nanorods via a combined ultrasonic-microwave irradiation approach. Ultrason. Sonochem..

[920] Liang B., Feng Y., Zhang W. (2017). ZnO whiskers synthesized by rapid microwave reduction reaction. Int. J. Mater. Res..

[921] Feng J., Wang Y., Zou L., Li B., He X., Ren Y., Lv Y., Fan Z. (2015). Synthesis of magnetic ZnO/ZnFe_2_O_4_ by a microwave combustion method, and its high rate of adsorption of methylene blue. J. Colloid Interface Sci..

[922] Potirak P., Pecharapa W., Techitdheera W. (2014). Microwave-assisted synthesis of ZnO/MWCNT hybrid nanocomposites and their alcohol-sensing properties. J. Exp. Nanosci..

[923] Kumar R., Kumar Singh R., Vaz A.R., Moshkalev S.A. (2015). Microwave-assisted synthesis and deposition of a thin ZnO layer on microwave-exfoliated graphene: Optical and electrochemical evaluations. RSC Adv..

[924] Guo Y., Chang B., Wen B., Zhao C., Yin C., Zhou Y., Wang Y., Yang B., Zhang S. (2016). One-pot synthesis of graphene/zinc oxide by microwave irradiation with enhanced supercapacitor performance. RSC Adv..

[925] Kumar R., Kumar Singh R., Singh D.P., Savu R., Moshkalev S.A. (2016). Microwave heating time dependent synthesis of various dimensional graphene oxide supported hierarchical ZnO nanostructures and its photoluminescence studies. Mater. Des..

[926] Varma H.K., Ananthakumar S., Warrier K.G.K., Damodaran A.D. (1996). Synthesis of zinc oxide varistors through microwave-derived precursor. Ceram. Int..

[927] Kim H.W., Kwon Y.J., Mirzaei A., Kang S.Y., Choi M.S., Bang J.H., Kim S.S. (2017). Synthesis of zinc oxide semiconductors-graphene nanocomposites by microwave irradiation for application to gas sensors. Sens. Actuators B-Chem..

